# Mapping of lamin A- and progerin-interacting genome regions

**DOI:** 10.1007/s00412-012-0376-7

**Published:** 2012-05-19

**Authors:** Nard Kubben, Michiel Adriaens, Wouter Meuleman, Jan Willem Voncken, Bas van Steensel, Tom Misteli

**Affiliations:** 1 Genome Cell Biology Group, National Cancer Institute, National Institutes of Health, Bethesda, 20892 MD USA; 2 Department of Experimental Cardiology, AMC Medical Research, Meibergdreef 15, 1100DD Amsterdam, the Netherlands; 3 Division of Gene Regulation, Netherlands Cancer Institute, Amsterdam, the Netherlands; 4 Department of Molecular Genetics, Maastricht University, 6229 ER Maastricht, the Netherlands

## Abstract

Mutations in the A-type lamins A and C, two major components of the nuclear lamina, cause a large group of phenotypically diverse diseases collectively referred to as laminopathies. These conditions often involve defects in chromatin organization. However, it is unclear whether A-type lamins interact with chromatin in vivo and whether aberrant chromatin–lamin interactions contribute to disease. Here, we have used an unbiased approach to comparatively map genome-wide interactions of gene promoters with lamin A and progerin, the mutated lamin A isoform responsible for the premature aging disorder Hutchinson–Gilford progeria syndrome (HGPS) in mouse cardiac myoytes and embryonic fibroblasts. We find that lamin A-associated genes are predominantly transcriptionally silent and that loss of lamin association leads to the relocation of peripherally localized genes, but not necessarily to their activation. We demonstrate that progerin induces global changes in chromatin organization by enhancing interactions with a specific subset of genes in addition to the identified lamin A-associated genes. These observations demonstrate disease-related changes in higher order genome organization in HGPS and provide novel insights into the role of lamin–chromatin interactions in chromatin organization.

**Electronic supplementary material:**

The online version of this article (doi:10.1007/s00412-012-0376-7) contains supplementary material, which is available to authorized users.

## Introduction

The nuclear envelope (NE) defines the boundary between the nucleus and the cytoplasm. The NE is composed of an outer and an inner nuclear membrane (ONM and INM, respectively) interrupted by nuclear pore complexes (NPCs) and is lined by the lamina, a network of the intermediate filaments made up largely of the A-type lamins (lamin A, Δ10, C, and C2), encoded by the *LMNA* gene, and B-type lamins (lamin B1, B2 and B3), encoded by the *LMNB1* and *LMNB2* genes, respectively (Broers et al. 2006).

Mutations in A-type lamins cause a group of phenotypically diverse diseases, collectively referred to as laminopathies (Broers et al. 2004). They include several types of muscular dystrophies, lipodystrophies, cardiomyopathies, neurological disorders, and premature aging syndromes. In line with the well-established role of A-type lamins in maintaining higher order chromatin organization (Sullivan et al. 1999), laminopathy-associated point mutations in the lamin A/C gene (*LMNA*) often deregulate chromatin structure and organization. The most dramatic laminopathy-associated chromatin reorganization occurs in the premature aging disease Hutchinson–Gilford progeria syndrome (HGPS), where the disease-causing mutant lamin A isoform progerin causes loss of heterochromatin and reduces mono- and trimethylation of lysines 9 and 20 on histone 3 (Scaffidi and Misteli 2006; Goldman et al. 2004). Another progeria-related lamin A mutation, E145K, leads to alterations in pericentric chromatin, abnormal clustering of centromeres, and mislocalization of telomeres (Taimen et al. 2009). Loss of heterochromatin and mislocalization of HP1β also occur in LMNA mutation-mediated mandibuloacral dysplasia (Filesi et al. 2005).

Several lines of evidence suggest that A-type lamins interact directly with chromatin in vivo and that these interactions are affected by *LMNA* mutations. First, the structurally related B-type lamins directly bind histones H2A and H2B in vitro (Goldberg et al. 1999), and lamin B interacts with chromatin in vivo at discrete lamin-associated domains (LADs; de Wit and van Steensel 2009). These domains are characterized by a low gene activity and are demarcated by insulators (9). Second, amino acids 396–430 of the human lamin A/C tail bind core histones in vitro (Taniura et al. 1995). Third, the immunoglobulin domain of lamin A/C covalently binds 30-bp dimerized DNA fragments in vitro, and the FPLD-associated R483Q and W mutations lower this affinity (Stierlé et al. 2003). Based on these findings, it was hypothesized that direct interaction of A-type lamins with chromatin is important for chromatin organization and gene regulation (Dechat et al. 2008). While interactions of chromatin with lamin B have been mapped (Peric-Hupkes et al. 2010; Pickersgill et al. 2006), it is unknown which chromatin regions directly interact with A-type lamins in vivo and how loss or mutation of lamin A/C affects chromatin organization and gene expression.

To probe the role of the lamina in genome organization, gene expression, and its relevance to laminopathies, we have conducted unbiased, genome-wide mapping of gene promoters that interact with lamin A and/or progerin using a high-affinity pull-down technique (Kubben et al. 2010). We find that lamin A preferentially binds silent or lowly expressed genes. This association facilitates, but does not determine, the peripheral localization, and loss of the interaction is not sufficient for gene activation. Progerin increases lamina–gene interactions by interacting with a specific subset of silent or lowly expressed genes in addition to binding regular lamin A-associated genes. These data demonstrate a direct and distinct effect of lamin A and progerin on chromatin interaction and organization at the lamina.

## Materials and methods

### Cell lines and culture

An OST-P lentiviral expression plasmid was created by deleting amino acids 609–658 of the pCDH MCSNard One-STrEP tagged lamin A (OST-A) plasmid (Pegoraro et al. 2009). A lentiviral vector expressing shRNA directed against mouse lamin A/C (pSIHpuro-shRNA-mouseLMNA) was generated by annealing 5′gatccGAGCTTGACTTCCAGAAGAACATttcaagagaATGTTCTTCTGGAAGTCAAGCTCtttttg3′ and 5′aattcaaaaaGAGCTTGACTTCCAGAAGAACATtctcttgaaATGTTCTTCTGGAAGTCAAGCTCg 3′ oligos and subsequent ligation into *Bam*HI- and *Eco*RI-restricted pSIH H1 plasmid (System Biosciences, Mountain View, USA). Mouse cardiac myocyte NklTAg cells (Rybkin et al. 2003) and mouse embryonic fibroblasts (MEFs) of wild-type and LMNA knockout (LMNA^KO−/−^) embryos (Sullivan et al. 1999) expressing OST-A or OST-P at expression levels comparable to endogenous lamin A levels were created and cultured as described elsewhere (Kubben et al. 2010).

### Western blot

Cells were lysed in SDS-PAGE Laemmli loading buffer and further denatured by heating for 5 min at 95°C. Western blots and immunodetection were performed essentially as described elsewhere (Pegoraro et al. 2009). The primary antibodies used for immunodetection were α-lamin A/C (1:500 dilution, Sc6215; StCruz, Santa Cruz, CA, USA) and α-beta actin (1:5,000 dilution, A-5441; Sigma, St. Louis, MO, USA).

### Immunofluorescence microscopy

Immunofluorescence was essentially performed as described elsewhere (Pegoraro et al. 2009). Cells were grown on 0.1 % gelatin/PBS solution-coated multi-well glass slides (MP Biomedicals, Solon, OH, USA), and α-LMNA/C (1:500 dilution, Sc-7292; StCruz), α-LMNB1 (1:500 dilution, Sc-6217; StCruz), or Chromeo642-conjugated Strep-Tactin and α-StrepMAB (both diluted 1:250, IBATAGnology, Göttingen, Germany) were used as a primary antibodies.

### Chromatin immunoprecipitation

NklTAg and MEF cell lines expressing OST-A, OST-P or control vector constructs were formaldehyde fixed (1 %, 5 min), solubilized by sonication in the presence of a high percentage of detergents (1 % SDS, 1 % Trx-100) and diluted to contain equal concentrations of genomic DNA as described previously (Kubben et al. 2010). Samples were end-over-end rotated (overnight, 4°) with herring sperm DNA coated Strep-Tactin Matrix (IBA BioTagnology, Göttingen, Germany). Pelleted Strep-Tactin matrix was washed with low and high salt buffer (0.1 % SDS, 1 % Triton X-100, 2 mM EDTA, 20 mM Tris pH 8.1, 150 mM and 500 mM NaCl respectively), LiCl buffer (0.25 M LiCl, 1 % Igepal-CA630, 1 % deoxycholic acid, 1 mM EDTA, 10 mM Tris pH 8.1), OST stringent wash buffer (2 M NaCl, 2 % Trx-100, 500 mM LiCl, 0.1 % SDS, 1 % Sodium Deoxycholate, 20 mM Tris pH 8.1, 2 mM EDTA) and TE buffer (10 mM Tris, 1 mM EDTA pH 8.0). Precipitated protein–chromatin complexes were eluted at room temperature in fresh elution buffer (1 % SDS and 0.1 M NaHCO3). After de-cross-linking for 6 h at 65°C in the presence of 200 mM NaCl and 30 μg/ml RNAase A, DNA was purified using a Qiagen PCR cleanup kit (Qiagen, Hilden, Germany) and amplified according to Whole Genome Amplification kit instructions (Sigma, St. Louis, USA) for further analysis on promoter arrays. Amplified DNA fragments were hybridized to NimbleGen MM8 385 K Refseq promoter arrays, tiling a region of 2000 bp downstream to 500 bp upstream for transcription start sites of ∼19,000 Refseq genes, according to Electronic supplementary material (ESM) Fig. S3. Hybridization and data acquisition were performed in-house by NimbleGen according to standard procedures (Roche NimbleGen, Madison, WI, USA). To identify OST-A-bound gene promoters in NklTAg cell lines, we compared OST-A chromatin immunoprecipitated DNA vs. control chromatin immunoprecipitated DNA hybridized to the same arrays (ESM Fig. S3) using a within-array analysis approach as specified by NimbleGen (version 6.2; NimbleGen 2010; http://www.nimblegen.com/products/lit/NG_ChIP-chip_Guide_v6p2.pdf), requiring overlapping peaks between replicates in addition to a false discovery rate (FDR) of <20 % in each replicate, as successfully applied previously (Romano et al. 2010). Identical to the NklTAG cell line ChIP analysis in MEF cell lines, genes interacting with OST-A or OST-P were identified by a within-array analysis comparing the probe levels of OST-A vs. control chromatin immunoprecipitated DNA or OST-P vs. control chromatin immunoprecipitated DNA hybridized on the same array (ESM Fig. S3). To further test whether the identified OST-A and OST-P targets in MEFs preferentially bind to OST-A or OST-P or interact with equal frequencies to both lamins, we next applied an in-between-array analysis (FDR = 5 %) to directly compare the probe signals of OST-A chromatin immunoprecipitated DNA with OST-P chromatin immunoprecipitated DNA, which were hybridized on separate arrays (ESM Fig. S3). This analysis has been previously successfully applied in ChIP studies and is further described elsewhere (Peric-Hupkes and van Steensel 2010). Both analyses combined led to the identification of three subsets of targets in MEFs: (1) gene promoters that bind preferentially to OST-A (referred to as A targets; OST-A ChIP enriched over OST-P and control ChIP and OST-P ChIP not enriched over control ChIP); (2) gene promoters that bind to both OST-A and OST-P (A&P targets; OST-A and OST-P ChIP enriched over control ChIP, in which OST-A and OST-P ChIP have comparable levels); (3) or gene promoters that preferentially bind to OST-P (P targets; OST-P ChIP enriched over OST-A and control ChIP and OST-A ChIP not enriched over control ChIP).

Cluster analysis of the identified lamin targets was essentially performed as described previously (Peric-Hupkes and van Steensel 2010). To test whether lamin targets were clustered, we defined the clusters as two or more adjacent lamin-associated genes not interrupted by non-target genes. Next, unclustered (cluster size = 1 gene) and clustered (cluster size >1 gene) lamin targets for all chromosomes were compared by Fisher’s exact test to the randomly expected occurrences of gene clusters, determined in 10,000 random simulations. Gene ontology (GO) analysis was performed with the DAVID database, comparing gene sets against all annotated gene promoters represented on NimbleGen MM8 385K Refseq promoter arrays, using standard settings for functional annotation clustering and listing significant clusters by the most significant GO biological process classes (Dennis et al. 2003; da Huang et al. 2009). Conservation plots were generated with CEAS analysis (http://cistrome.org/ap/root; Ji et al. 2006), showing the level of conservation of the ChIP regions compared to the genomic background. Genomatix MatInspector software (Cartharius et al. 2005) combined with the Genomatix transcription factor motif database (www.genomatix.de) was used to identify enriched transcription factor motifs (TFMs) in target sites and target promoters.

### Fluorescent in situ hybridization

To produce probes for DNA fluorescent in situ hybridization (FISH), bacterial artificial chromosomes (BACs; BACPAC Resources Center, Oakland, CA, USA) were labeled by nick translations with dUTP conjugated with biotin or dioxygenin (Roche, Madison, WI, USA) using mouse BAC clones (Acpp, RP24-383K20; Sp100, RP24-235A6; OLFR681, RP24-324M2; OLFR1471, RP24-346K11; Eif2b, RP24-285P5; Fanca, RP24-157M4) as described (Meaburn and Misteli 2008). FISH was performed as described elsewhere (Meaburn and Misteli 2008), with the exception of altered denaturation conditions (5 min, 85°C) and the introduction of a 45-min (20°C) incubation step in a block buffer (3 % BSA/0.05 % Tween-20/4× SSC) diluted (1:50) lamin B antibody (Sc-6217; Santa Cruz Biotechnology, Santa Cruz, CA, USA) prior to incubation with appropriate secondary antibodies.

Using SoftWoRx 3.7.0 Release 13EL (Applied Precision), FISH signals were detected on an IX70 microscope (Olympus) controlled by a Deltavision System (Applied Precision, ×60 1.4 oil objective lens, auxiliary magnification of 1.5 and optical step size of 0.2 μm) and analyzed in the *z* section with the brightest signal intensity. The two brightest FISH signals were used for quantification. The frequency of cells with minimally one FISH signal within 500-nm distance of lamin B staining was quantified. Minimally, 150 nuclei were analyzed in duplicate per probe per cell line. Per probe quantification results were statistically tested between different cell lines using the chi-square test. SigmaStat 3.1 software was used for statistical analysis. All test *P* values <0.05 were considered significant.

### RNA isolation and expression microarray

RNA from the cardiac left ventricles of 5-day-old wild-type (WT) and LMNA^GT−/−^ mice (*N* = 2 each); a LMNA null mouse model (Kubben et al. 2011); and from MEFs infected with a OST-A, OST-P, or empty vector lentivirus (*N* = 2 each) were isolated with the RNAeasy minikit (Qiagen, Hilden, Germany) and hybridized to Nugo Mouse Affymetrix Moe430A expression arrays and Affymetrix Mouse Gene 1.0 ST arrays, respectively. Intensity values after hybridization were normalized to the median signal intensity of the array. For individual genes, differences in expression levels were statistically tested by one-way ANOVA. The expression profiles for all sets of targets were statistically analyzed in R using a two-sample Kolmogorov–Smirnov test between groups, comparing the profiles of the subsets of targets as defined above to one another and against the expression profile of non-targets. Values of *p* below 0.05 were considered significant. For verification of microarray expression data by PCR, cDNA was synthesized with the iScript™ cDNA synthesis kit (BioRad, Hercules, CA, USA). SYBR Green real-time quantitative PCR analysis was performed with primers specified in ESM Fig. S4c. Differences in real-time PCR quantification were determined with Student’s *t* test and at the 0.05 significance level.

## Results

### Identification of lamin A-associated genes

To map lamin A-interacting chromatin regions at a genome-wide scale, we used a recently described high-efficiency pull-down system in which lamin A tagged with the biotin-derived One-STrEP tag (OST-A) is stably expressed at endogenous levels (Kubben et al. 2010) in a murine cardiac myocyte NklTAg cell line (Rybkin et al. 2003). The OST-A fusion protein has previously been characterized in detail (Kubben et al. 2010) and co-localizes in NklTAg cells with endogenous lamin A and lamin B1 (ESM Fig. S1). To identify lamin A-associated genome regions, we performed chromatin immunoprecipitation (ChIP) using the Strep-Tactin matrix, an engineered streptavidin analogue for selective binding (*K*_d_ = 1 μM) to the OST tag (Junttila et al. 2005; ESM Fig. S2a). The recovered chromatin was hybridized in duplicate to the NimbleGen promoter tiling arrays covering the 2,500-bp promoter regions flanking the transcriptional start site of ∼19,000 annotated Refseq genes. Chromatin immunoprecipitated DNA from empty vector infected cells was used as a control (ESM Fig. S2a). Six hundred ninety-two lamin A-associated (OST-A) genes were identified based on enrichment in two independent experiments and statistical criteria (Romano et al. 2010; ESM Table S1; see “Materials and methods”). Visual inspection of the processed probe-level data for eight randomly chosen lamin A targets and four non-targets showed little variability between duplicates and confirmed an overall high enrichment of OST-A chromatin immunoprecipitated DNA vs. control chromatin immunoprecipitated DNA in lamin A targets, but not in non-targets.

**Fig. 1:**
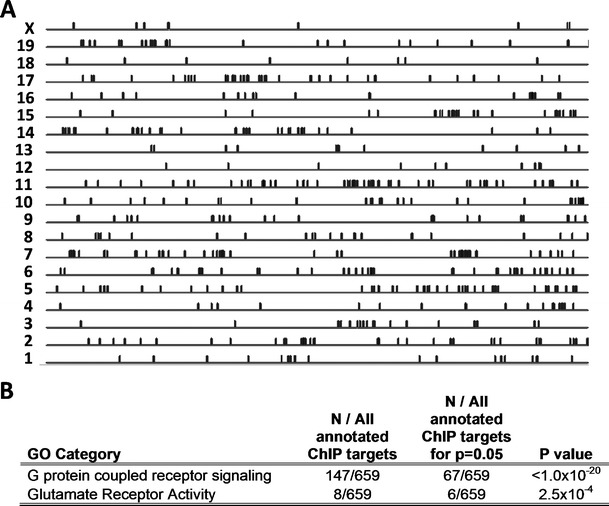
Genomic localization and characteristics of lamin-associated genes. **a** ChIP-chip targets of OST-A in NklTAg myocytes plotted according to their genomic location on a relative scale correcting for individual chromosome size differences. **b** Gene ontology (*GO*) analysis on identified lamin A targets. Per GO category, the number of lamin A-associated genes enriched in comparison to the total number of GO annotated genes is indicated, as well as the minimal amount of genes to reach significance (*p* = 0.05)

The 692 OST-A target genes localize to all chromosomes (Fig. 1a and ESM Fig. S5). Of the lamin A targets, 28 % localize in gene clusters (defined as two or more adjacent genes), which is fivefold higher (*p* < 1.0 × 10^−4^; Fisher’s exact test) than expected for random gene association (ESM Fig. S6a, b). GO analysis identified the enrichment of lamin A targets in the categories “G protein-coupled receptor signaling” (*N* = 147/659 annotated genes, *p* < 1.0 × 10^−20^; Fig. 1b), mainly consisting of olfactory receptors (OLFR) and VMNR (*N* = 104, *N* = 11 respectively), and “glutamate receptor activity” (*N* = 8/659, *p* = 2.5 × 10^−4^; Fig. 1b). Conservation plots did not reveal a significant sequence conservation of precipitated DNA fragments (ESM Fig. S7), arguing against the existence of a specific lamin A-binding sequence. Analysis of TFMs revealed 4 of the 181 known TFMs to be significantly enriched in OST-A-associated gene promoters in comparison to random promoter sequences (*p* < 0.05; ESM Table S2). These enriched TFMs consist of those for the HAML (*p* = 3.2 × 10^−4^), BPTF (*p* = 4.3 × 10^−4^), AIRE (*p* < 1.0 × 10^−4^), and ZF10 TF families (*p* = 3.2 × 10^−2^). These data demonstrate that A-type lamin-interacting gene promoters preferentially localize in genomic clusters, lack unique consensus lamin A-binding sequences, and are enriched for a small set of specific transcription factor binding motifs.

### Nuclear localization of lamin A-associated genes

**Fig. 2:**
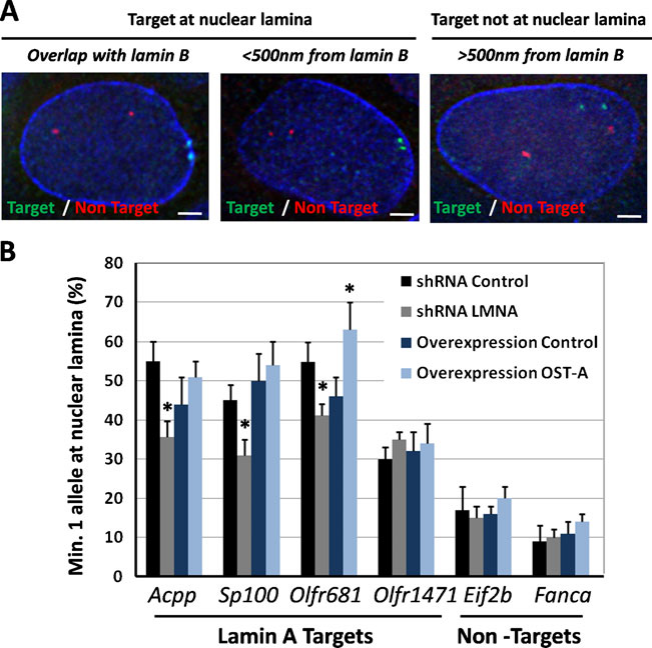
Subnuclear localization of lamin A-associated genes. **a** FISH analysis of one lamin A targets (*Sp100*, *green*) and one non-target (*Fanca*, *red*) gene in NklTAg cells. Targets are defined as FISH signals overlapping with or within 500 nm of lamin B staining (*blue*). The *white line* indicates a distance of 1,500 nM. **b** Quantification of FISH signals for four lamin A targets (*Acpp, Sp100, Olfr681*, and *Olfr1471*) and two non-targets (*Eif2b* and *Fanca*) in NklTAg cells infected with control shRNA, lamin A/C shRNA, OST-A, or a control vector. *Asterisks* indicate a significant (*p* < 0.05, *χ* ^2^ test) change in peripheral positioning in comparison to control infected cells

To assess the subnuclear localization of lamin A-associated genes and to validate the ChIP technique, the location of four randomly chosen lamin A targets and two non-targets were probed by DNA FISH in NklTAg cells. We measured the percentage of cells with at least one FISH signal within 500 nm of the nuclear periphery identified by lamin B, representing <5 % of the average nuclear diameter (10.5 ± 1.5 μm; Fig. 2a). For the randomly selected lamin A targets *Acpp*, *Sp100*, *Olfr681*, and *Olfr1471* located on chromosomes 9, 1, 7, and 19, respectively, at least one allele localized to the periphery in 55, 45, 55, and 30 %, respectively, of cells compared to 17 and 9 % for the non-targets *Eif2b* and *Fanca* (Fig. 2b). The difference between the peripheral localization of lamin A targets and non-targets is statistically significant at *p* < 0.001 in a *χ* ^2^ test.

Next, we set out to probe the effects of loss of A-type lamins on the subnuclear localization of lamin A-associated genes by knocking down lamin A/C in NklTAg cells using shRNA. As a control, the expression of lamin A/C shRNA resulted in over 90 % knockdown for both lamin A and C proteins (ESM Fig. S4b). Upon knockdown, the peripheral localization of the lamin A target loci *Acpp*, *Sp100*, and *OLFR681* decreased significantly to 35, 31, and 25 % (*χ* ^2^ test: *p* < 0.01; Fig. 2b). In contrast, the least peripherally localized lamin-associated *OLFR1471* locus did not change its position upon loss of lamin A (*χ* ^2^ test: *p* > 0.05; Fig. 2b). The non-targets *Eif2b* and *Fanca* retained their subnuclear, non-peripheral positions (*χ* ^2^ test: *p* > 0.05; Fig. 2b). On the other hand, the overexpression of OST-A at endogenous levels did not affect the localization of lamin A-associated genes, with the exception of the lamin A target *OLFR681* (from 46 to 63 %, *p* < 0.05, *χ* ^2^ test). These findings support a role for lamin A in retaining gene promoters at the nuclear periphery.

### Loss of lamin A association is not sufficient for gene activation

**Fig. 3:**
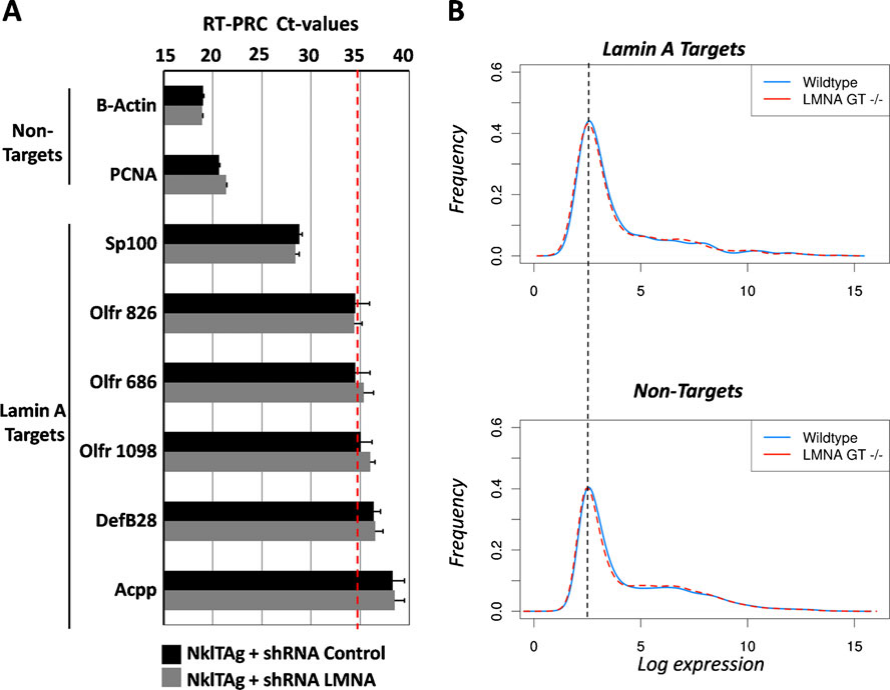
Expression profiles of lamin A-associated genes. **a** Real-time quantitative PCR on six lamin A targets (*Sp100*, *Olfr826*, *olfr686*, *olfr1098, Defb28*, and *Acpp*) and two non-targets (*B-Actin* and *Pcna*) in the presence and absence of lamin A/C shRNA in NklTAg myocytes. *C*_t_ values (±SD) were determined in six independent samples using equal amounts of starting material (100 ng cDNA) and corrected for the mRNA levels of the housekeeping gene GAPDH. The detection limit of quantitative PCR (*C*_t_ ∼ 35) is indicated with a *dotted line* (Akhunova et al. 2009). **b** Genome-wide RNA expression profiles (log_2_ values) of lamin A-associated genes and non-target genes in cardiac left ventricle of wild-type and LMNA^GT−/−^ mice, a functional knockout for A-type lamins (Kubben et al. 2011)

To test whether loss of lamina association is sufficient for the reactivation of silent genes, we first probed for the transcriptional activity of the lamin A-associated and peripherally located genes *Acpp*, *Sp100* (Fig. 2b), and several other randomly chosen lamin A targets (*Olfr826*, *Olfr686*, *Olfr1098*, *Defb28*; Fig. 3a). The expression levels were determined by qPCR analysis on NklTAg cardiac myocytes expressing control vs. lamin A/C shRNA (ESM Fig. S4b) and the microarray expression profiles of neonatal cardiac tissues of WT and LMNA^GT−/−^ mice, a LMNA null model based on gene trap technology (Kubben et al. 2011). These lamin A targets were mostly very lowly or not expressed (*C*_t_ = 28–38), and none changed expression upon loss of lamin A association in NklTAG cardiac mycoytes (Fig. 3a).

We extended these findings to the genome-wide level as the basal expression profiles of all annotated lamin A-interacting genes (*N* = 559) differed significantly from non-target genes and indicated overall lower expression levels (Δlog_2_ = −0.66, *p* < 0.05, two-sample Kolmogorov–Smirnov test; Fig. 3b). Among the 559 annotated lamin A targets, 175 genes (31 %) were expressed at levels below the 20th percentile within the total cardiac myocyte transcriptome (log_2_ < 2.57, which translates into >170 times lower expression than the housekeeping gene GAPDH), 313 genes (56 %) were expressed between the 20th and 80th percentiles (2.57 < log_2_ > 6.77), and 71 genes (13 %) were expressed above the 80th percentile (log_2_ > 6.77; ESM Table S3). Highly expressed lamin A interactors (log_2_ > 6.77, *N* = 71) were not enriched for any GO category. Loss of A-type lamins in LMNA^GT−/−^ cardiac tissues significantly changed the transcriptional profile of 1,136 genes (Kubben et al. 2011). The average expression fold change (FC) of the 559 lamin A-associated genes was similar to the FC observed in the total genome and indicated no preferential effect of loss of A-type lamins on the lamin A targets (OST-A targets: FC = 0.99 ± 0.08, *p* = 0.734; non-target: FC = 1.01 ± 0.11, *p* = 0.624, two-sample Kolmogorov–Smirnov test; Fig. 3a). Individual lamin A-bound genes that significantly changed expression upon loss of A-type lamins (*N* = 12) were mostly expressed between log_2_ expressions of 2.57 and 6.77 (20th to 80th percentiles, *N* = 8), but also included three low expressors (log_2_ < 2.57, 20th percentile) and *repin1*, which was expressed highly (log_2_ = 7.30; ESM Table S4). This group of 12 lamin A targets was not enriched for a particular GO category (*p* > 0.05; ESM Table S4). These findings not only support a role for lamin A in the nuclear organization of chromatin via capturing transcriptionally silent genes but also suggest that loss of this association is not sufficient for gene activation.

### Identification of lamin A- and progerin-associated genome regions

**Fig. 4:**
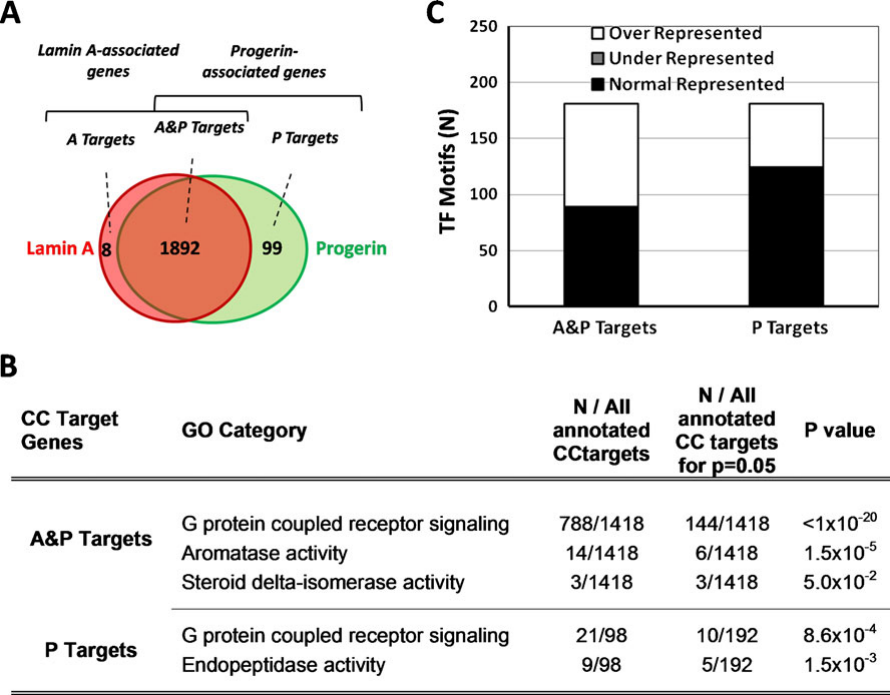
Characteristics of common and differential lamin A- and progerin-associated genes. **a** Venn diagram of lamin A- and progerin-interacting genes determined by ChIP-chip in MEF cells (ESM Fig. S2). **b** Gene ontology (*GO*) analysis on A&P and P targets indicating the GO class, the amount of genes (*N*) per total amount of annotated ChIP target genes, the minimal amount of targets needed for a significance level of 0.05, and the significance level. **c** Transcription factor motif analysis on all subclasses of lamin-associated genes for respectively over-, under-, and normal representation of 181 transcription factor motifs (*TFM*)

After characterizing lamin A–chromatin interactions in cardiac mycoytes, we next set out to probe for aberrant progerin–chromatin interactions in a separate set of experiments performed in MEFs. To identify genome regions which interact preferentially with lamin A, progerin, or both, we expressed OST-tagged lamin A (OST-A) or progerin (OST-P) at endogenous levels in MEFs (Kubben et al. 2010). OST-A and OST-P co-localize with endogenous A-type lamins and LMNB1 in MEFs (ESM Fig. S1). OST-P expression, in contrast to OST-A, leads to distortions of the nuclear lamina (ESM Figs. S1 and S4a) and global loss of LAP2 and HP1γ nuclear levels (Kubben et al. 2010) similar to the cellular phenotypes observed in HGPS patient cells (Scaffidi and Misteli 2006), further indicating the full functionality of OST-tagged lamin A and progerin in MEFs. Lamin A- and progerin-associated gene promoters were identified by a combined statistical analysis (see “Materials and methods”) comparing individual array signals from OST-A chromatin immunoprecipitated chromatin and OST-P chromatin immunoprecipitated chromatin with each other and chromatin immunoprecipitated chromatin from control vector infected cells (ESM Fig. S2b). We identified 1,900 lamin A-associated gene promoters and 1991 progerin-associated gene promoters (Fig. 4a and ESM Table S1). Lamin-associated genes identified in NklTAg and MEF cells significantly overlapped as 37 and 38 % of the genes identified at the nuclear lamina in NklTAg cells, associated with lamin A or progerin, respectively, in MEFs as well (*p* < 1 × 10^−4^, *χ* ^2^ test).

The vast majority (99.6 %, *N* = 1,892) of the identified lamin-associated genes in MEFs interacted with both lamin A and progerin and will further be referred to as A&P targets (Fig. 4a). With the exception of eight genes (A targets; ESM Table S5), all lamin A-associated genes were also bound by progerin. In contrast, a larger set of 99 genes (0.5 %) was found to preferentially interact with progerin, but not lamin A (P targets; ESM Table S6). Closer examination of the genomic localization reveals that 76 and 74 % of the lamin A- and progerin-associated genes, respectively, are located in clusters (2–31 adjacent targets), which is more frequent than expected for random gene sets (*p* < 1 × 10^−4^; ESM Fig. S6c–f), and both sets of interactors overlap significantly (*p* < 1 × 10^−15^; Fisher’s exact test).

A&P targets were significantly enriched in “G protein-coupled receptor signaling” (788 of 1,488 annotated genes, *p* < 1.0 × 10^−20^; Fig 4b), “aromatase activity” (14 of 1,488, *p* = 1.5 × 10^−5^; Fig 4b), and “steroid delta-isomerase activity” (3 of 1,488, *p* = 5.0 × 10^−2^) GO categories. P targets were to a lesser extent enriched for “G protein-coupled receptor signaling” (21 of 98 annotated genes, *p* = 8.6 × 10^−4^) and also enriched for the “endopeptidase activity” (9 of 98, *p* = 1.5 × 10^−3^) GO category. The enrichment of different GO families amongst P and A&P targets suggests distinct preferential association of various gene groups with progerin.

No consensus lamin binding sequence could be identified in P or A&P targets (ESM Fig. S7). TFM analysis indicated the enrichment of A&P-associated gene promoters for 92 of 181 TFMs (*p* < 0.05; Fig. 4c and ESM Table S2). The most highly enriched TFMs include those for the hepatocyte nuclear factor 6 (*p* < 1.0 × 10^−10^), the spermatide-specific transcription factor RUSH (*p* < 1.0 × 10^−10^), the keratinocyte differentiation required transcription factor Brn-5 (*p* < 1.0 × 10^−10^), and neuronal expressed Brn Pou domain TF families (BRNF, *p* < 1.0 × 10^−10^). In contrast, P targets were less strongly enriched (minimal *p* = 9.99 × 10^−7^) for fewer TFMs (*N* = 57). Of the 57 TFMs that occurred more frequently than in random promoter sequences, SF1 (*p* = 4.6 × 10^−2^) and craniofacial development involved GTF2IRD1 upstream control element (GUCE, *p* = 2.4 × 10^−2^) were found to be not enriched in A&P-associated gene promoters. Overall, these data show that lamin A and progerin interact differentially with chromatin and that progerin-associated genes possess distinct features, including their biological function and TFMs.

### Global gene expression profiles of lamin A- and progerin-associated genes

**Fig. 5:**
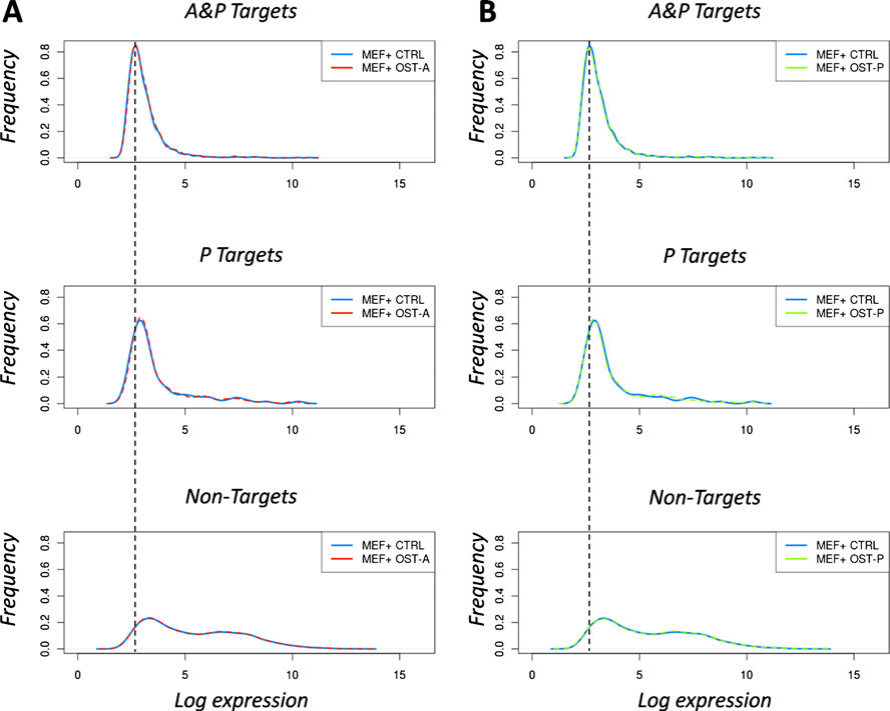
Expression profiles of lamina/progerin- and progerin-specific associated genes. **a** Genome-wide RNA expression profiles of A&P, P, and non-targets in MEFs expressing an empty control vector vs. OST-A. **b** Similar expression profiles in MEFs expressing an empty control vector vs. OST-P

To examine the expression behavior of lamina-associated genes in response to the overexpression of lamin A or progerin, we analyzed mRNA from MEFs transfected with OST-A, OST-P, or a control vector using expression arrays. The introduction of progerin changed the global transcriptional profile significantly (468 genes down, 702 genes up; *p* < 0.05, ANOVA), and these changes were different from the overexpression of wild-type lamin A (571 up, 517 down; 379 changed by both OST-A and OST-P). In agreement with previous studies using HGPS patient skin fibroblasts (Csoka et al. 2004), we found specific misregulation of genes involved in extracellular matrix organization and cell cycle control in cells expressing progerin. In MEFs infected with a control vector, the expression profiles of P and A&P targets were statistically different from non-target genes (*p* < 0.05, two-sample Kolmogorov–Smirnov test; Fig. 5b). Both A&P targets (Δlog_2_ = −2.2, *p* < 0.05, two-sample Kolmogorov–Smirnov test) and, to a lesser extent, P targets (Δlog_2_ = −1.7, *p* < 0.05, two-sample Kolmogorov–Smirnov test) were repressed compared to non-target genes. The mRNA levels of both classes of interactors differed significantly from each other as well (Δlog_2_ = −0.6, *p* < 0.05, two-sample Kolmogorov–Smirnov test). These basal expression levels of the subclasses did not change significantly upon OST-A or OST-P expression in WT MEFs (*p* > 0.05, two-sample Kolmogorov–Smirnov test; Fig. 5a, b), suggesting that the expression of lamin A or progerin is not sufficient to induce a global change in the gene activity of lamina-associated genes.

## Progerin-induced changes in gene expression of lamina-associated genes

Finally, to identify lamina-associated genes whose expression is specifically misregulated in HGPS, we searched for ChIP targets whose transcription was affected by progerin in MEFs. One hundred eighty-nine of the 1,999 genes associated with lamin A and/or progerin display significantly altered expressions upon the introduction of OST-A or OST-P into MEFs (Table 1). This group of interacting genes (*N* = 189) is particularly enriched for transcriptional regulators (*N* = 43, “regulation of transcription” GO category, *p* = 1.9 × 10^−5^) and includes ten zinc finger proteins (*MYM-type* *2 zinc finger*, *Zinc finger-64*, *Zinc finger-157*, *Zinc finger-187*, *Zinc finger-386*, *Zinc finger-397*, *Zinc finger-426*, *Zinc finger-568*, and *Zinc finger-760*); two members of the SWI/SNF complex (*Smarcc2* and *Smarca2*); two PPAR transcription factor-related proteins (*Ppara* and *Pparg1cb*); and well-described transcriptional regulators like *JunD* and *Retinoblastoma 1* (*Rb1*; Table 1).

**Table 1:** A-type lamin-associated genes that change expression upon introduction of lamin A or progerin

Entrez gene ID	Gene abbreviation	Target type	Expression WT+Ctrl	Expression WT+OST-A	Expression WT+OST-P	FC A/Ctrl	FC P/Ctrl	FC P/A	Expression change		Regulator of transcription?	HGPS candidate?
Lamin A only changes expression												
216742	A730024A03Rik	MEF A&P Common	2.58	2.92	2.77	1.27^a^	1.14	0.90	A↑	P-	No	No
18720	Pip5k1b	MEF A&P Common	3.95	4.29	3.94	1.26^a^	0.99	0.78	A↑	P-	No	No
232237	Fgd5	MEF P	3.22	3.52	3.19	1.23^a^	0.98	0.80^a^	A↑	P-	No	No
338337	Cog3	MEF A&P Common	2.15	2.44	2.49	1.22^a^	1.27	1.04	A↑	P-	No	No
110521	Hivep1	MEF A&P Common	2.78	3.06	2.89	1.21^a^	1.08	0.89	A↑	P-	Yes	No
382252	A830080D01Rik	MEF A&P Common	5.05	5.33	5.23	1.21^a^	1.13	0.93^a^	A↑	P-	No	No
56709	Dnajb12	MEF A&P Common	3	3.26	3.07	1.19^a^	1.05	0.88	A↑	P-	No	No
64103	Tnmd	MEF A&P Common	3.64	3.87	3.41	1.17^a^	0.85	0.73	A↑	P-	No	No
226747	Ahctf1	MEF A&P Common	2.86	3.07	2.88	1.15^a^	1.01	0.88	A↑	P-	Yes	No
68564	Nufip2	MEF A&P Common	3.57	3.78	3.58	1.15^a^	1.01	0.87^a^	A↑	P-	No	No
320743	A630005I04Rik	MEF P	2.8	3	2.82	1.14^a^	1.01	0.88	A↑	P-	No	No
12452	Ccng2	MEF A&P Common	3.05	3.23	3.11	1.13^a^	1.04	0.92	A↑	P-	No	No
69256	Zfp397	MEF A&P Common	3.85	4.03	3.91	1.13^a^	1.04	0.92	A↑	P-	Yes	No
17681	Msc	MEF A&P Common	2.72	2.9	2.81	1.13^a^	1.06	0.94^a^	A↑	P-	Yes	No
18763	Pkd1	MEF A&P Common	3.18	3.36	3.27	1.13^a^	1.06	0.94	A↑	P-	No	No
240034	BC029103	MEF A&P Common	2.64	2.81	2.76	1.12^a^	1.09	0.97	A↑	P-	Yes	No
78943	Ern1	MEF A&P Common	2.67	2.84	2.79	1.12^a^	1.09	0.97	A↑	P-	Yes	No
54353	Scap2	MEF A&P Common	2.29	2.45	2.44	1.11^a^	1.11	0.99	A↑	P-	No	No
20448	St6galnac4	MEF A&P Common	2.56	2.72	2.53	1.11^a^	0.98	0.88	A↑	P-	No	No
71721	1200015N20Rik	MEF A&P Common	2.39	2.55	2.4	1.11^a^	1.01	0.90	A↑	P-	No	No
209039	Tenc1	MEF A&P Common	2.28	2.43	2.36	1.10^a^	1.06	0.95	A↑	P-	No	No
54169	Myst4	MEF A&P Common	2.21	2.35	2.18	1.10^a^	0.98	0.89	A↑	P-	Yes	No
319468	Ppm1h	MEF A&P Common	2.56	2.7	2.58	1.10^a^	1.01	0.92	A↑	P-	No	No
66412	Arrdc4	MEF P	3.21	3.35	3.2	1.10^a^	0.99	0.90	A↑	P-	No	No
20927	Abcc8	MEF A&P Common	2.56	2.68	2.6	1.08^a^	1.03	0.95	A↑	P-	No	No
229584	Pogz	MEF A&P Common	3.3	3.42	3.17	1.08^a^	0.91	0.84	A↑	P-	Yes	No
269523	Vcp	MEF A&P Common	2.82	2.92	2.77	1.07^a^	0.97	0.90	A↑	P-	No	No
109815	H47	MEF A&P Common	2.62	2.71	2.74	1.06^a^	1.09	1.02	A↑	P-	No	No
21652	Phf1	MEF A&P Common	2.95	3.04	2.96	1.06^a^	1.01	0.95	A↑	P-	Yes	No
20361	Sema7a	MEF A&P Common	4.31	4.39	4.43	1.05^a^	1.09	1.03	A↑	P-	No	No
11416	Slc33a1	MEF A&P Common	2.82	2.89	2.72	1.04^a^	0.93	0.89^a^	A↑	P-	No	No
74320	Wdr33	MEF A&P Common	2.16	2.23	2.24	1.04^a^	1.06	1.01	A↑	P-	No	No
22722	Zfp64	MEF A&P Common	2.1	2.17	2.22	1.04^a^	1.09	1.04	A↑	P-	Yes	No
68094	Smarcc2	MEF A&P Common	2.75	2.82	2.71	1.04^a^	0.97	0.93	A↑	P-	Yes	No
17133	Maff	MEF A&P Common	2.99	3.05	2.98	1.04^a^	0.99	0.95	A↑	P-	Yes	No
14629	Gclc	MEF A&P Common	2.33	2.38	2.32	1.03^a^	0.99	0.96^a^	A↑	P-	Yes	No
230793	Ahdc1	MEF P	2.77	2.81	2.89	1.02^a^	1.09	1.06	A↑	P-	No	No
12487	Cd28	MEF A	3.38	3.42	3.45	1.02^a^	1.05	1.02	A↑	P-	No	No
14057	Sfxn1	MEF A&P Common	2.51	2.54	2.53	1.02^a^	1.01	0.99	A↑	P-	No	No
12837	Col8a1	MEF A&P Common	2.79	2.75	2.75	0.97^a^	0.97	1.00	A↓	P-	No	No
114674	Gtf2ird2	MEF A&P Common	3.74	3.69	3.65	0.96^a^	0.94	0.97	A↓	P-	Yes	No
56878	Rbms1	MEF P	2.25	2.19	2.17	0.95^a^	0.95	0.99	A↓	P-	No	No
71520	Grap	MEF A&P Common	3.16	3.09	3.1	0.95^a^	0.96	1.01	A↓	P-	No	No
76007	Zfp198	MEF A&P Common	3.31	3.22	3.38	0.93^a^	1.05	1.12	A↓	P-	Yes	No
269955	Rccd1	MEF A&P Common	4.42	4.32	4.49	0.93^a^	1.05	1.13	A↓	P-	No	No
18571	Pdcd6ip	MEF A&P Common	3.13	3.03	2.91	0.93^a^	0.86	0.92	A↓	P-	No	No
52428	Rhpn2	MEF A&P Common	4.24	4.13	4.19	0.92^a^	0.97	1.04	A↓	P-	No	No
227743	Mapkap1	MEF A&P Common	2.97	2.86	2.88	0.92^a^	0.94	1.01	A↓	P-	No	No
26885	Casp8ap2	MEF A&P Common	4.14	4.01	4.3	0.91^a^	1.12	1.22^a^	A↓	P-	Yes	No
245631	Mum1l1	MEF A&P Common	4.14	4.01	4.15	0.91^a^	1.01	1.10^a^	A↓	P-	No	No
226075	Glis3	MEF A&P Common	2.65	2.52	2.66	0.91^a^	1.01	1.10^a^	A↓	P-	Yes	No
78558	Htra3	MEF A&P Common	2.59	2.46	2.49	0.91^a^	0.93	1.02	A↓	P-	No	No
17354	Mllt10	MEF A&P Common	3.56	3.42	3.45	0.90^a^	0.93	1.02	A↓	P-	No	No
74243	2210009G21Rik	MEF A&P Common	2.05	1.86	1.98	0.87^a^	0.95	1.09	A↓	P-	No	No
218877	Sema3g	MEF A&P Common	2.25	2.05	2.06	0.87^a^	0.88	1.01	A↓	P-	No	No
18145	Npc1	MEF A&P Common	2.35	2.13	2.37	0.85^a^	1.01	1.18	A↓	P-	No	No
73102	3110004L20Rik	MEF P	10.3	10.06	10.14	0.84^a^	0.90	1.06	A↓	P-	No	No
212307	Mapre2	MEF A&P Common	3.77	3.53	3.68	0.84^a^	0.94	1.11^a^	A↓	P-	No	No
67448	Plxdc2	MEF A&P Common	3.41	2.69	3.14	0.60^a^	0.83	1.37	A↓	P-	No	No
Progerin only changes expression												
11730	Ang3	MEF A&P Common	5.14	5.22	5.87	1.05	1.66^a^	1.57	A-	P↑	No	
100201	Tmem64	MEF P	5.28	5.28	5.98	1.00	1.62^a^	1.62	A-	P↑	No	No
67770	5830433M19Rik	MEF A&P Common	2.76	2.94	3.13	1.13	1.29^a^	1.14	A-	P↑	No	No
68010	Bambi	MEF A&P Common	2.76	2.94	3.13	1.13	1.29^a^	1.14	A-	P↑	No	No
245007	Zbtb38	MEF P	8.76	8.83	9.08	1.04	1.25^a^	1.19	A-	P↑	Yes	No
240396	Rkhd2	MEF A&P Common	7.22	7.44	7.52	1.16	1.23^a^	1.06	A-	P↑	No	No
20623	Snrk	MEF A&P Common	3.43	3.73	3.71	1.23	1.21^a^	0.99	A-	P↑	No	No
382051	4833426J09Rik	MEF A&P Common	3.24	3.38	3.49	1.10	1.19^a^	1.08	A-	P↑	No	No
621080	AI429214	MEF P	6.27	6.25	6.5	0.98	1.17^a^	1.19	A-	P↑	No	No
432731	Zfp187	MEF A&P Common	2.55	2.68	2.78	1.09	1.17^a^	1.07	A-	P↑	Yes	No
233168	AI987944	MEF A&P Common	3.97	4.11	4.2	1.10	1.17^a^	1.06	A-	P↑	Yes	No
80860	D11Lgp1e	MEF A&P Common	3.97	4.11	4.2	1.10	1.17^a^	1.06	A-	P↑	No	No
17064	Cd93	MEF A&P Common	3.52	3.55	3.73	1.02	1.16^a^	1.13^a^	A-	P↑	No	Yes
80289	Lysmd3	MEF A&P Common	2.24	2.37	2.41	1.09	1.13^a^	1.03	A-	P↑	No	No
74196	2610511O17Rik	MEF A&P Common	4.14	4.23	4.3	1.06	1.12^a^	1.05	A-	P↑	No	No
13643	Efnb3	MEF A&P Common	2.89	3.09	3.05	1.14	1.12^a^	0.97	A-	P↑	No	No
67581	Tbc1d23	MEF A&P Common	3.13	3.4	3.27	1.20	1.10^a^	0.91	A-	P↑	No	No
80880	Ankrd47	MEF A&P Common	2.76	2.85	2.89	1.06	1.09^a^	1.03	A-	P↑	No	No
13349	Darc	MEF A&P Common	2.87	3.13	3	1.19	1.09^a^	0.91	A-	P↑	No	No
18806	Pld2	MEF A&P Common	3.84	3.9	3.96	1.04	1.09^a^	1.04	A-	P↑	No	No
67246	2810474O19Rik	MEF A&P Common	2.54	2.47	2.64	0.95	1.07^a^	1.13^a^	A-	P↑	No	Yes
18127	Nos3	MEF A&P Common	2.82	3.18	2.92	1.28	1.07^a^	0.84	A-	P↑	No	No
16640	Klra9	MEF A&P Common	2.42	2.46	2.49	1.02	1.05^a^	1.02	A-	P↑	No	No
13712	Elk1	MEF A&P Common	2.5	2.58	2.57	1.05	1.05^a^	0.99	A-	P↑	Yes	No
12494	Cd38	MEF A&P Common	2.4	2.55	2.46	1.10	1.04^a^	0.94	A-	P↑	No	No
243905	Zfp568	MEF A&P Common	3.4	3.54	3.46	1.10	1.04^a^	0.95	A-	P↑	Yes	No
28169	Agpat3	MEF A&P Common	3.84	3.9	3.9	1.04	1.04^a^	1.00	A-	P↑	No	No
78134	Gpr23	MEF A&P Common	2.23	2.22	2.28	0.99	1.04^a^	1.04	A-	P↑	No	No
102414	Clk3	MEF A&P Common	2.27	2.41	2.32	1.10	1.04^a^	0.94	A-	P↑	No	No
209497	AW547186	MEF A&P Common	2.18	2.33	2.23	1.10	1.04^a^	0.93	A-	P↑	No	No
21682	Tec	MEF A&P Common	2.95	3.01	2.93	1.04	0.99^a^	0.95	A-	P↓	No	No
27060	Tcirg1	MEF A&P Common	2.87	2.82	2.83	0.96	0.97^a^	1.01	A-	P↓	No	No
208718	4930429A22Rik	MEF A&P Common	2.92	2.95	2.87	1.02	0.97^a^	0.95^a^	A-	P↓	No	Yes
57444	Isg20	MEF A&P Common	2.88	2.9	2.83	1.01	0.97^a^	0.95^a^	A-	P↓	No	Yes
93691	Klf7	MEF A&P Common	3.84	3.76	3.78	0.94	0.96^a^	1.01	A-	P↓	Yes	No
71361	Amid	MEF A&P Common	3.24	3.21	3.18	0.97	0.96^a^	0.98	A-	P↓	No	No
78757	4921505C17Rik	MEF A&P Common	2.29	2.33	2.23	1.02	0.96^a^	0.93	A-	P↓	No	No
225339	E230022H04Rik	MEF A&P Common	2.57	2.48	2.51	0.93	0.96^a^	1.02	A-	P↓	No	No
230103	Npr2	MEF A&P Common	2.42	2.37	2.36	0.96	0.96^a^	0.99	A-	P↓	No	No
76295	Atp11b	MEF A&P Common	2.24	2.22	2.18	0.98	0.96^a^	0.97	A-	P↓	No	No
21969	Top1	MEF A&P Common	2.78	2.73	2.71	0.96	0.95^a^	0.99	A-	P↓	No	No
231201	AF366264	MEF A&P Common	2.63	2.66	2.56	1.02	0.95^a^	0.93	A-	P↓	No	No
13518	Dst	MEF A&P Common	2.34	2.36	2.27	1.01	0.95^a^	0.94^a^	A-	P↓	No	Yes
71361	Amid	MEF A&P Common	2.4	2.38	2.33	0.98	0.95^a^	0.97	A-	P↓	No	No
50754	Fbxw7	MEF A&P Common	2.68	2.72	2.61	1.02	0.95^a^	0.93	A-	P↓	No	No
99899	Ifi44	MEF A&P Common	2.41	2.44	2.34	1.02	0.95^a^	0.93^a^	A-	P↓	No	Yes
215335	Slc36a1	MEF A&P Common	3.32	3.37	3.24	1.03	0.95^a^	0.91	A-	P↓	No	No
654824	Ankrd37	MEF A&P Common	2.82	2.81	2.74	0.99	0.95^a^	0.95	A-	P↓	No	No
56220	Zfp386	MEF A&P Common	2.86	2.84	2.78	0.98	0.95^a^	0.96^a^	A-	P↓	Yes	Yes
67037	Pmf1	MEF A&P Common	2.48	2.55	2.4	1.04	0.95^a^	0.90	A-	P↓	Yes	No
70676	Gulp1	MEF A&P Common	2.44	2.36	2.36	0.94	0.95^a^	1.00	A-	P↓	No	No
22592	Ercc5	MEF A&P Common	2.96	3.06	2.87	1.07	0.94^a^	0.88^a^	A-	P↓	No	Yes
228357	Lrp4	MEF A&P Common	3.13	3.03	3.04	0.93	0.94^a^	1.01	A-	P↓	No	No
225164	Mib1	MEF A&P Common	2.67	2.63	2.58	0.97	0.94^a^	0.97	A-	P↓	No	No
67966	Zcchc10	MEF A&P Common	4.42	4.39	4.32	0.97	0.93^a^	0.95	A-	P↓	No	No
239337	Adamts12	MEF A&P Common	2.85	2.83	2.75	0.98	0.93^a^	0.95^a^	A-	P↓	No	Yes
67760	Slc38a2	MEF A&P Common	2.7	2.65	2.6	0.96	0.93^a^	0.97	A-	P↓	No	No
69274	Ctdspl	MEF A&P Common	2.48	2.45	2.38	0.97	0.93^a^	0.95	A-	P↓	No	No
170826	Ppargc1b	MEF A&P Common	3.71	3.69	3.6	0.98	0.93^a^	0.94^a^	A-	P↓	Yes	Yes
14911	Thumpd3	MEF A&P Common	3.34	3.31	3.23	0.97	0.93^a^	0.95	A-	P↓	No	No
20668	Sox13	MEF A&P Common	3.74	3.68	3.63	0.95	0.93^a^	0.97	A-	P↓	Yes	No
329015	1810013C15Rik	MEF A&P Common	2.84	2.81	2.72	0.97	0.92^a^	0.94	A-	P↓	No	No
14465	Gata6	MEF A&P Common	2.61	2.61	2.49		0.92^a^	0.92	A-	P↓	Yes	No
17122	Mxd4	MEF A&P Common	2.98	2.89	2.86	0.93	0.92^a^	0.98	A-	P↓	Yes	No
78896	1500015O10Rik	MEF A&P Common	2.56	2.58	2.44	1.01	0.92^a^	0.91	A-	P↓	No	No
217031	Tada2l	MEF A&P Common	2.44	2.34	2.32	0.93	0.92^a^	0.99	A-	P↓	Yes	No
15939	Ier5	MEF A&P Common	2.9	2.95	2.77	1.03	0.91^a^	0.88	A-	P↓	No	No
14609	Gja1	MEF A&P Common	2.58	2.49	2.45	0.93	0.91^a^	0.97	A-	P↓	No	No
224697	Adamts10	MEF A&P Common	2.74	2.67	2.61	0.95	0.91^a^	0.96	A-	P↓	No	No
13518	Dst	MEF A&P Common	2.39	2.41	2.26	1.01	0.91^a^	0.90	A-	P↓	No	No
15903	Id3	MEF A&P Common	3.88	3.9	3.72	1.01	0.90^a^	0.88	A-	P↓	Yes	No
320487	D930036F22Rik	MEF A&P Common	3.32	3.25	3.16	0.95	0.90^a^	0.94	A-	P↓	No	No
227541	Camk1d	MEF A&P Common	3.11	3.07	2.95	0.97	0.90^a^	0.92	A-	P↓	No	No
13614	Edn1	MEF A&P Common	2.76	2.75	2.6	0.99	0.90^a^	0.90^a^	A-	P↓	No	Yes
217588	Mbip	MEF A&P Common	3	2.89	2.84	0.92	0.90^a^	0.97	A-	P↓	No	No
170753	Gig1	MEF A&P Common	2.95	2.8	2.79	0.90	0.90^a^	0.99	A-	P↓	No	No
15900	Irf8	MEF A&P Common	3.31	3.16	3.14	0.90	0.89^a^	0.99	A-	P↓	Yes	No
72154	2610020C11Rik	MEF A&P Common	2.8	2.61	2.63	0.87	0.89^a^	1.01	A-	P↓	Yes	No
19645	Rb1	MEF A&P Common	3.93	3.83	3.76	0.93	0.89^a^	0.95	A-	P↓	Yes	No
14872	Gstt2	MEF A&P Common	3.07	3.14	2.89	1.04	0.88^a^	0.84	A-	P↓	No	No
213541	Ythdf2	MEF A&P Common	2.86	2.85	2.68	0.99	0.88^a^	0.89	A-	P↓	No	No
234736	Rfwd3	MEF A&P Common	2.67	2.52	2.49	0.90	0.88^a^	0.98	A-	P↓	No	No
71586	Ifih1	MEF A&P Common	3.27	3.24	3.09	0.97	0.88^a^	0.90	A-	P↓	No	No
66812	Ppcdc	MEF A&P Common	3.23	3.31	3.05	1.05	0.88^a^	0.84^a^	A-	P↓	No	Yes
15081	H3f3b	MEF A&P Common	3.21	3.13	3.02	0.94	0.88^a^	0.93	A-	P↓	No	No
108062	Cstf2	MEF A&P Common	3.23	3.19	3.04	0.97	0.88^a^	0.90	A-	P↓	No	No
235028	Zfp426	MEF A&P Common	3.13	3.16	2.94	1.02	0.88^a^	0.86	A-	P↓	Yes	No
101565	6330503K22Rik	MEF A&P Common	2.84	2.77	2.65	0.95	0.88^a^	0.92	A-	P↓	No	No
107746	Rapgef1	MEF A&P Common	4.7	4.46	4.51	0.84	0.88^a^	1.04	A-	P↓	No	No
22695	Zfp36	MEF A&P Common	6.21	6.07	6.01	0.90	0.87^a^	0.96	A-	P↓	No	No
73095	2900084M01Rik	MEF A&P Common	3.37	3.31	3.17	0.95	0.87^a^	0.91	A-	P↓	No	No
338365	Slc41a2	MEF A&P Common	5.47	5.41	5.25	0.95	0.86^a^	0.90	A-	P↓	No	No
66481	Rps21	MEF A&P Common	2.96	2.81	2.74	0.90	0.86^a^	0.95	A-	P↓	No	No
19013	Ppara	MEF A&P Common	3.22	3.11	2.99	0.92	0.85^a^	0.92	A-	P↓	Yes	No
17423	Ndst2	MEF A&P Common	2.86	2.88	2.63	1.01	0.85^a^	0.84	A-	P↓	No	No
252837	Ccrl1	MEF A&P Common	3.54	3.37	3.29	0.88	0.84^a^	0.95	A-	P↓	No	No
71041	Pcgf6	MEF A&P Common	3.27	3.26	3.02	0.99	0.84^a^	0.85	A-	P↓	Yes	No
13823	Epb4.1l3	MEF A&P Common	6	6.05	5.74	1.03	0.84^a^	0.81	A-	P↓	No	No
74153	Ube1l	MEF A&P Common	5.22	5.05	4.94	0.88	0.82^a^	0.93	A-	P↓	No	No
70676	Gulp1	MEF A&P Common	2.69	2.61	2.4	0.94	0.82^a^	0.86	A-	P↓	No	No
225160	Thoc1	MEF A&P Common	2.79	2.64	2.49	0.90	0.81^a^	0.90	A-	P↓	Yes	No
67155	Smarca2	MEF A&P Common	4.05	4.49	3.6	1.35	0.73^a^	0.54	A-	P↓	Yes	No
231986	AI591476	MEF P	7.24	7.02	6.53	0.85	0.61^a^	0.71	A-	P↓	Yes	No
Lamin A and progerin change expression												
233989	Hnrpul	MEF A&P Common	2.55	2.89	2.73	1.26^a^	1.13^a^	0.90	A↑	P↑	Yes	No
232989	Hnrpul1	MEF A&P Common	2.16	2.47	2.41	1.23^a^	1.19^a^	0.96^a^	A↑	P↑	Yes	No
170748	BC017612	MEF A&P Common	2.36	2.63	2.54	1.20^a^	1.13^a^	0.94	A↑	P↑	No	No
234699	BC022641	MEF A&P Common	2.99	3.23	3.11	1.18^a^	1.09^a^	0.92^a^	A↑↑	P↑	No	No
57439	1300007B12Rik	MEF A&P Common	3.08	3.31	3.5	1.17^a^	1.34^a^	1.14	A↑	P↑	No	No
67155	Smarca2	MEF A&P Common	7.3	7.49	7.55	1.14^a^	1.19^a^	1.04	A↑	P↑	Yes	No
387524	Znrf2	MEF A&P Common	2.45	2.61	2.56	1.11^a^	1.08^a^	0.97	A↑	P↑	No	No
67713	Dnajc19	MEF A&P Common	3.28	3.41	3.38	1.09^a^	1.07^a^	0.98	A↑	P↑	No	No
241447	Lass6	MEF A&P Common	3.52	3.64	3.47	1.08^a^	0.97^a^	0.89^a^	A↑	P↓	Yes	Yes
226517	Smg7	MEF A&P Common	3.35	3.42	3.27	1.04^a^	0.95^a^	0.90^a^	A↑	P↓	No	Yes
26410	Map3k8	MEF A&P Common	3.35	3.42	3.27	1.04^a^	0.95^a^	0.90^a^	A↑	P↓	No	Yes
16478	Jund1	MEF A&P Common	2.94	2.89	2.83	0.96^a^	0.93^a^	0.96^a^	A↓	P↓↓	Yes	Yes
71446	Wrb	MEF A&P Common	2.63	2.57	2.45	0.95^a^	0.88^a^	0.92	A↓	P↓	No	No
54216	Pcdh7	MEF A&P Common	3.32	3.25	3.17	0.95^a^	0.90^a^	0.95	A↓	P↓	No	No
15944	Irgm	MEF A&P Common	2.46	2.35	2.38	0.92^a^	0.95^a^	1.02^a^	A↓↓	P↓	No	No
16668	Krt1-18	MEF A&P Common	2.95	2.84	2.83	0.92^a^	0.92^a^	0.99	A↓	P↓	No	No
214290	Zcchc6	MEF A&P Common	3.21	3.09	3.06	0.92^a^	0.90^a^	0.98	A↓	P↓	No	No
94044	Bcl2l13	MEF A&P Common	8.77	8.63	8.54	0.90^a^	0.85^a^	0.94	A↓	P↓	No	No
77124	9130221H12Rik	MEF A&P Common	3.02	2.87	2.86	0.90^a^	0.90^a^	0.99	A↓	P↓	No	No
12322	Camk2a	MEF A&P Common	4.17	4	4.09	0.88^a^	0.95^a^	1.06	A↓	P↓	No	No
12322	Camk2a	MEF A&P Common	3.8	3.63	3.56	0.88^a^	0.85^a^	0.95	A↓	P↓	No	No
16004	Igf2r	MEF A&P Common	3.17	2.98	3.03	0.87^a^	0.91^a^	1.04	A↓	P↓	No	No
26412	Map4k2	MEF A&P Common	3.64	3.4	3.51	0.84^a^	0.91^a^	1.08	A↓	P↓	No	No
57814	Kcne4	MEF A&P Common	4.04	3.72	3.57	0.80^a^	0.72^a^	0.90	A↓	P↓	No	No
67333	Stk35	MEF A&P Common	3.34	2.97	2.98	0.77^a^	0.78^a^	1.01	A↓	P↓	No	No
243612	D630042P16Rik	MEF A&P Common	7.32	6.49	6.75	0.56^a^	0.67^a^	1.20	A↓	P↓	No	No
192187	Stab1	MEF A&P Common	5.77	4.61	5.06	0.44^a^	0.61^a^	1.37	A↓	P↓	No	No

^a^mRNA expression level linear fold changes differ significantly (*p* < 0.05, no fold change criterion; ANOVA) in MEF OST-A vs. empty control cells, in MEF OST-P vs. empty control cells, or in MEF OST-P vs. MEF OST-A. HGPS candidates are indicated (yes/no) based on significantly changing expression levels upon the introduction of OST-P as compared to both empty control cells and MEF OST-A expression levels. When both MEF OST-P and MEF OST-A expression levels changed significantly compared to empty control cells, only those genes in which expression changes were opposite or in similar direction, but more pronounced upon the introduction of OST-P, were selected as HGPS candidates. Genes involved in “regulation of transcription,” as based on the gene ontology annotation, are indicated within the second last column.

Sixteen targets out of the group of 189 lamin-interacting genes were considered bona fide candidates for HGPS as they all changed expression upon the introduction of progerin in comparison to MEFs expressing lamin A or empty vector constructs (Table 1). Of these candidates, 12 changed expression in response to progerin only (range *Cd93*, +16 %; *Ppcdc*, *−*12 %), 3 were down-regulated in response to progerin while lamin A increased their expression (*Lass6*: +8 % OST-A vs. *−*3 % OST-P; *Smg7*: *+*4 % OST-A vs. *−*5 % OST-P; *Map3k8*: *+*4 % OST-A vs. *−*5 % OST-P), and 1 decreased expression more upon the introduction of progerin as compared to lamin A (*Jund1*: *−*4 % OST-A vs. *−*7 % OST-P). The expression levels of 11 candidates remained below the 20th percentile of control MEF transcriptome gene expression levels (log_2_ < 3.31), while the other five candidates were only slightly more highly expressed (range = 3.52 < log_2_ > 3.69). Among the HGPS candidates, *Jun1d*, *Lag1 homolog ceramide synthethase* (*Lass6*), and *PPARγ coactivator 1B* (*Pparg1cb*) are all involved in transcriptional regulation (GO annotation; Table 1). Overall, these analyses identify lamina-associated genes that are specifically controlled by progerin.

## Discussion

Aberrant chromatin organization is a hallmark of many laminopathies, and disrupted lamin A–chromatin interactions have been suggested to contribute to the etiology of several laminopathies (Dechat et al. 2008). We here map in a genome-wide, unbiased fashion genes which interact with lamin A or with the disease-causing lamin A isoform progerin. We found that lamin A preferentially binds to peripherally localized, genomically clustered, and silent genes and that lamin A and progerin display differential chromatin interaction. Furthermore, we demonstrate that loss or gain of interaction with lamins changes the subnuclear position of several interacting genes, but per se is not sufficient to change their gene expression levels. We speculate that altered gene location may predispose genes for subsequent aberrant regulation.

Recent studies using the DamID method to map lamin-interacting genome regions have revealed the importance of lamina–chromatin interactions in chromatin organization and gene regulation by identifying sharply defined LADs across the genome (Guelen et al. 2008; Peric-Hupkes et al. 2010). LADs typically contain gene-poor, low-expressed regions and cell type specifically silenced gene clusters (Shevelyov et al. 2009). In agreement with these studies which were based on the analysis of chromatin interactions with lamin B, we here report, using an independent complementary approach, that lamin A-associated genes are generally silent or expressed at very low levels, genomically clustered, and preferentially localized at the nuclear periphery. Consistent with this, GO analysis reveals that lamin A-associated genes are enriched in gene groups such as sensory perception or glutamate-based neurotransmission, which are non-functional in the fibroblasts or cardiomyocytes analyzed here and hence expected to be transcriptionally silent (Lomvardas et al. 2006; Palmada and Centelles 1998). The enrichment of many tissue and cell type-specific transcription factor binding motifs (TFMs) in lamin A-associated gene promoters further supports this notion. Other enriched TFMs may contribute to the transcriptional repressive environment of the NE by maintaining condensed chromatin (Aoki et al. 1998).

We identified and compared lamin A- and progerin-associated gene promoters. Nearly all lamin A-associated genes (99.5 %) also interact with progerin. While we cannot completely rule out that the large overlap is due to the cross-linking of endogenous lamin A plus its associated promoter regions to OST-tagged progerin, two lines of evidence argue against this scenario. Firstly, we find that under similar experimental cross-linking conditions, OST-tagged progerin does not pull down NPC components despite the presence of lamin A, which is known to interact with NPCs (Kubben et al. 2010). Secondly, DamID experiments to map lamina–chromatin interactions performed in the absence of cross-linking using multiple nuclear lamina proteins (lamin B1 and emerin) show a large degree of overlap in the set of interacting genes (de Wit and van Steensel 2009; Peric-Hupkes et al. 2010).

We probed the relevance of lamin–chromatin interactions in the subnuclear positioning and regulation of gene expression in lamin A knockdown and overexpression systems. Despite a substantial relocalization of various lamin A-associated genes to the nuclear interior upon lamin A/C knockdown, these loci remain overall more frequently localized at the NE compared to non-targets. The reverse experimental approach by the overexpression of lamin A in cardiac myocytes demonstrates that elevated levels of lamin A can result in even further increased peripheral localization for some lamin A targets. Combined, these findings support a role for A-type lamins in facilitating the recruitment of silenced genes to the nuclear periphery. Interactions with additional nuclear lamina proteins, like emerin and lamin B, which are known chromatin interactors (de Wit and van Steensel 2009), likely contribute to this functional nuclear organization. These findings are in line with the observation that the overexpression of lamin A results in altered gene expression (Scaffidi and Misteli 2011).

A key finding is that while the localization of predominantly silent genes to the periphery is dependent on lamin A, dissociation from the lamina does not necessarily lead to their activation. We speculate that loss of lamina association is only one of multiple steps required for gene activation. Similar behavior has previously been observed for the CFTR locus whose relocation due to the activation and consequent internalization of neighboring genes by itself is insufficient for its activation (Zink et al. 2004; Sadoni et al. 2008). Furthermore, olfactory receptors, which were identified as the most prominent group of lamin A targets in our study, require a multistep process for their stochastic activation (Lomvardas et al. 2006). It is possible that dissociation from the nuclear periphery renders these genes “poised” and facilitates their activation by subsequent signals that may occur, e.g., during differentiation. Extending these studies to differentiation models in which additional factors support the gene activation of specific subsets of target genes will be important.

We identify a group of genes which attach to the lamina only in the presence of progerin. This class of genes is distinct from common lamin A and progerin targets by a number of characteristics: First, progerin targets are involved in different biological processes as compared to common targets, including the general active pathway of proteolysis, and are less enriched for TFMs enriched in promoters bound by both lamin A and progerin. Furthermore, genome-wide expression profile analysis shows that regardless of the presence of progerin, the basal expression levels of common targets are lower than for progerin target genes. These data indicate that transcriptional silencing at the nuclear lamina is strongest for common targets and suggest that progerin targets partially escape a repressive effect of the NE, possibly due to a loss of interaction with the lamina under normal conditions. Despite the fact that these progerin-sensitive genes remain lowly expressed and do not change expression dramatically upon the presence of progerin, additional steps might be required for gene activation in disease situations. A particular enrichment for transcriptional regulators amongst lamin interactors whose expression is differentially regulated by lamin A and progerin hints at a role of general transcriptional misregulation in HGPS, which is in line with previous findings indicating widespread misregulation (Csoka et al. 2004). Among the targets for HGPS, the transcription factor *JunD* is of particular interest as it has been previously linked to senescence-associated growth arrest (Meixner et al. 2010; Sheerin et al. 2002). Further studies will, however, be required to prove and characterize a role of JunD, as well as the other progerin targets identified here, in HGPS.

## Electronic supplementary material

Below is the link to the electronic supplementary material.

Figure S1 (JPEG 44 kb)

High resolution image (TIFF 13367 kb)

Figure S2 (JPEG 75 kb)

High resolution image (TIFF 12790 kb)

Figure S3 (JPEG 97 kb)

High resolution image (TIFF 17086 kb)

Figure S4 (JPEG 94 kb)

High resolution image (TIFF 14509 kb)

Figure S5 (JPEG 62 kb)

High resolution image (TIFF 9603 kb)

Figure S6 (JPEG 64 kb)

High resolution image (TIFF 11457 kb)

Figure S7 (JPEG 63 kb)

High resolution image (TIFF 11610 kb)

Table S1 (DOCX 325 kb)

## Abbreviations

AIRE: Autoimmune regulator
ANOVA: One-way analysis of variance
BAC: Bacterial artificial chromosome
BPTF: Bromeodomain and plant homeo-domain transcription factor
ChIP: Chromatin immunoprecipitation
FDR: False discovery rate
FPLD: Familial partial lipodystrophy
FC: Fold change
GT: Gene trap
GO: Gene ontology
GUCE: GTF2IRD1 upstream control element
HAML: Human acute myelogenous leukemia factor
HGPS: Hutchinson–Gilford progeria syndrome
INM: Inner nuclear membrane
IFNγ: Interferon gamma
LAD: Lamin-associated domain
LMNA^KO−/−^: LMNA knockout
MEF: Mouse embryonic fibroblast
NE: Nuclear envelope
NPC: Nuclear pore complex
OLFR: Olfactory receptor
OST-A: One-STrEP tagged lamin A
OST-P: One-STrEP tagged progerin
ONM: Outer nuclear membrane
SF1: Steroidogenic factor-1
TF: Transcription factor
TFM: Transcription factor motif
VMNR: Vomeronasal receptor
WT: Wild type
ZF10: Zinc finger 10
