# Supplementary figures and images for: Mapping of lamin A- and progerin-interacting genome regions
Source: Chromosoma. 2012 May 19;121(5):447–64. doi: 10.1007/s00412-012-0376-7 (PMC3443488; doi:10.1007/s00412-012-0376-7)

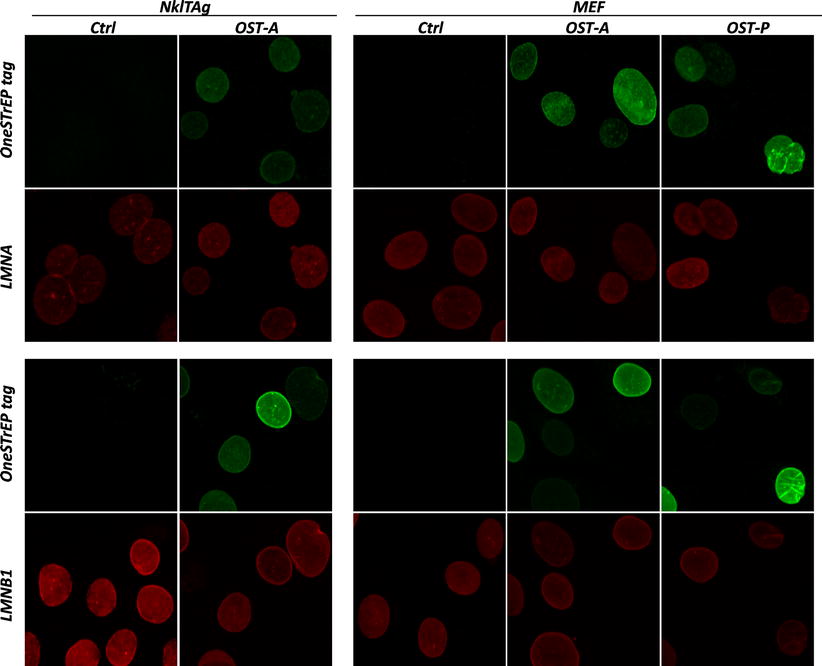

Supplement: Supplementary file 1 — (JPEG 44 kb) [file 412_2012_376_Fig6_ESM.jpg]

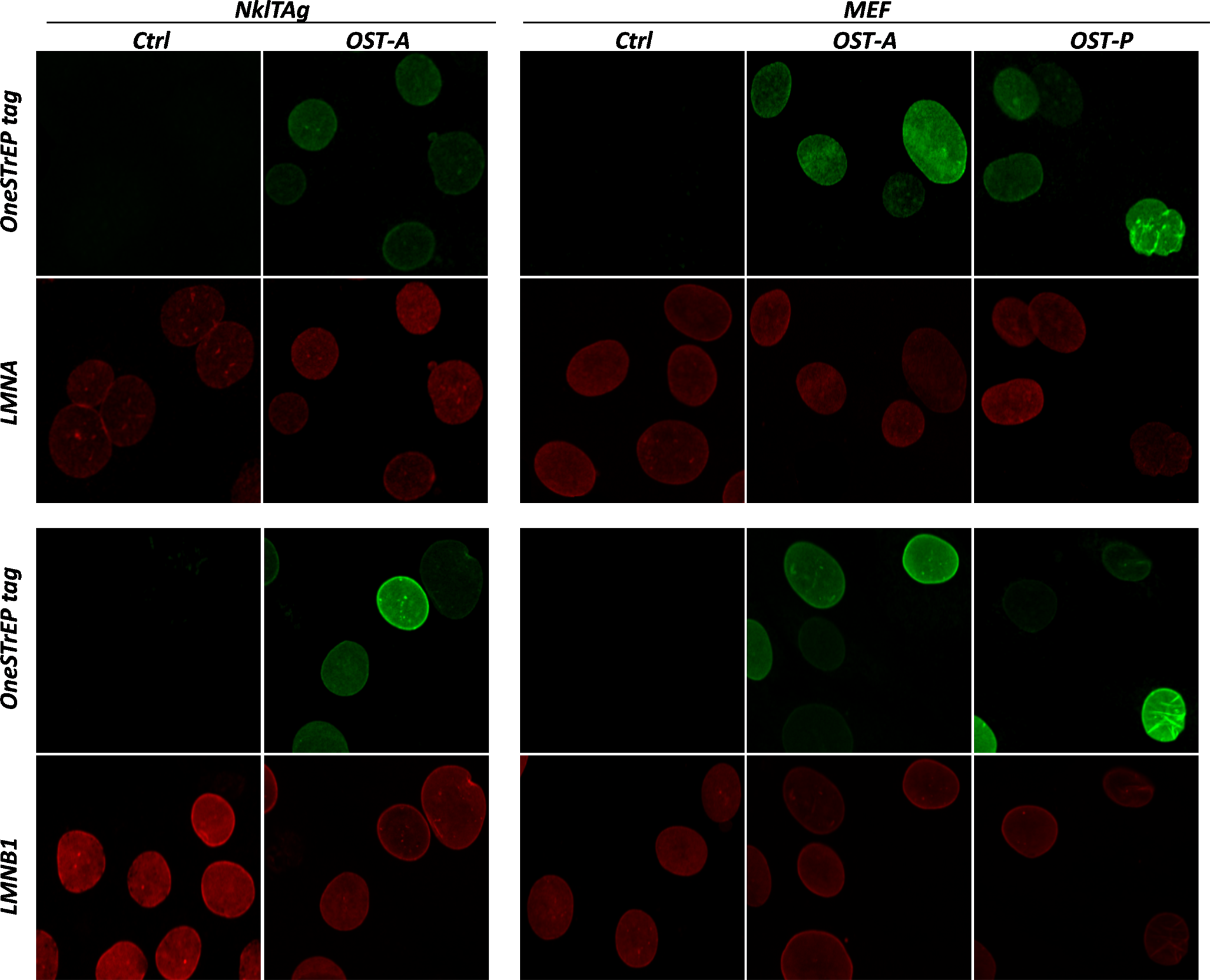

Supplement: Supplementary file 2 — High resolution image (TIFF 13367 kb) [file 412_2012_376_MOESM1_ESM.tif]

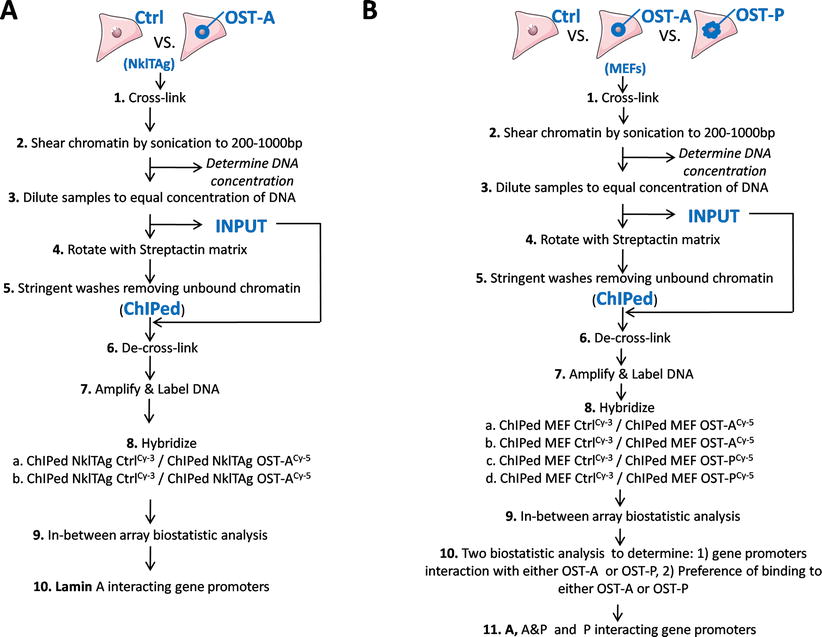

Supplement: Supplementary file 3 — (JPEG 75 kb) [file 412_2012_376_Fig7_ESM.jpg]

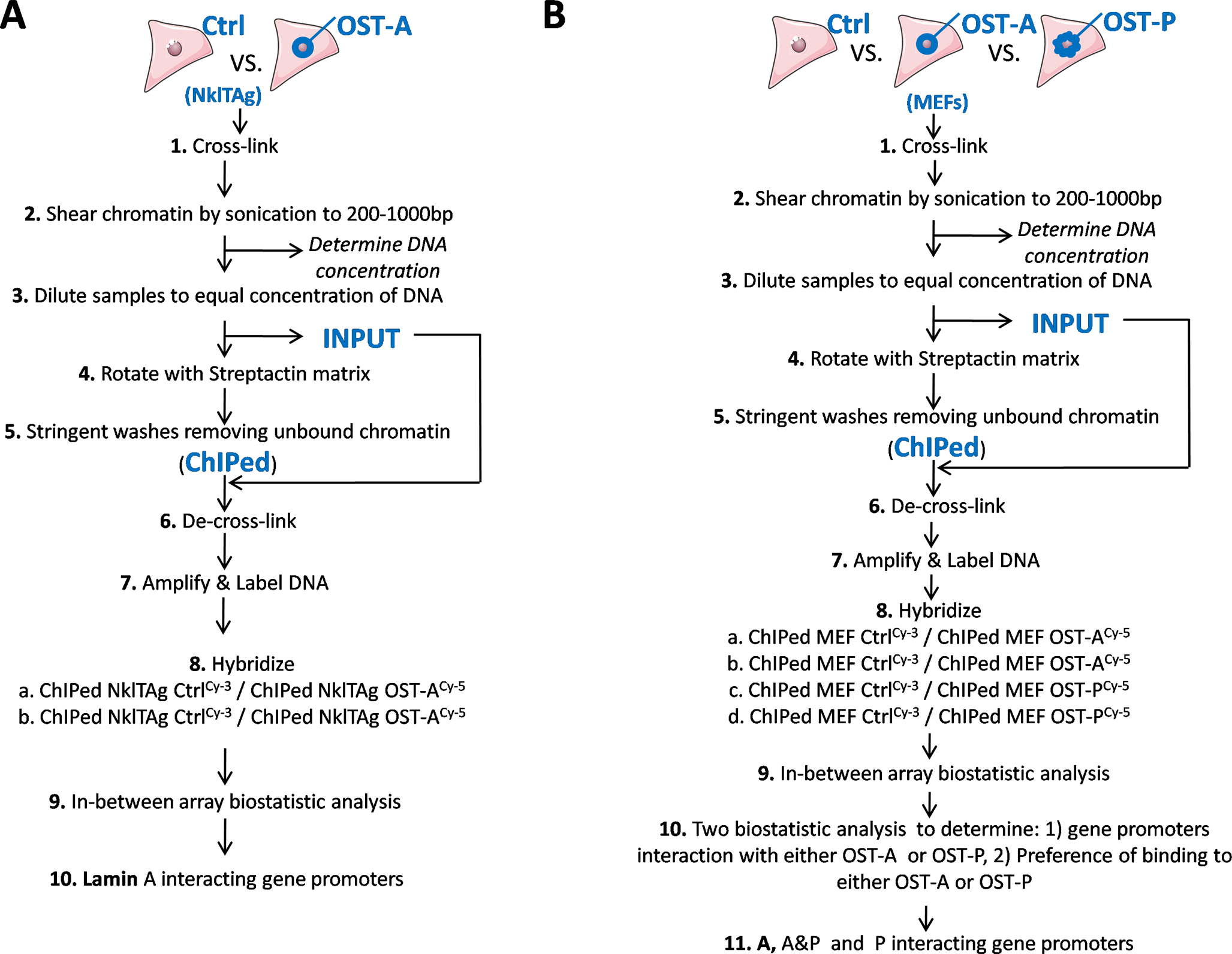

Supplement: Supplementary file 4 — High resolution image (TIFF 12790 kb) [file 412_2012_376_MOESM2_ESM.tif]

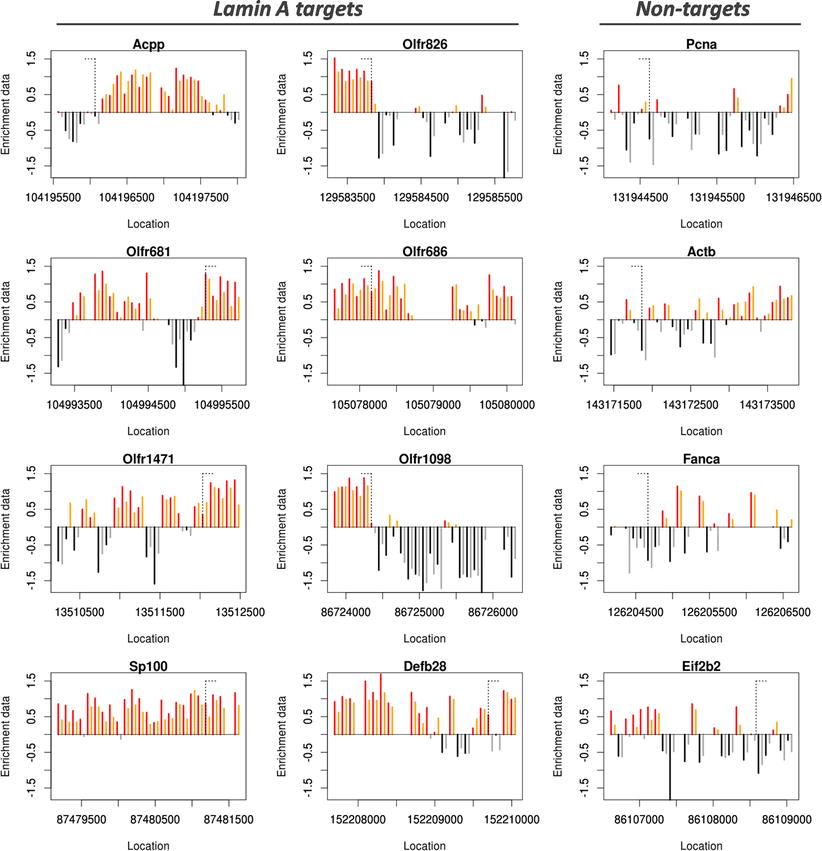

Supplement: Supplementary file 5 — (JPEG 97 kb) [file 412_2012_376_Fig8_ESM.jpg]

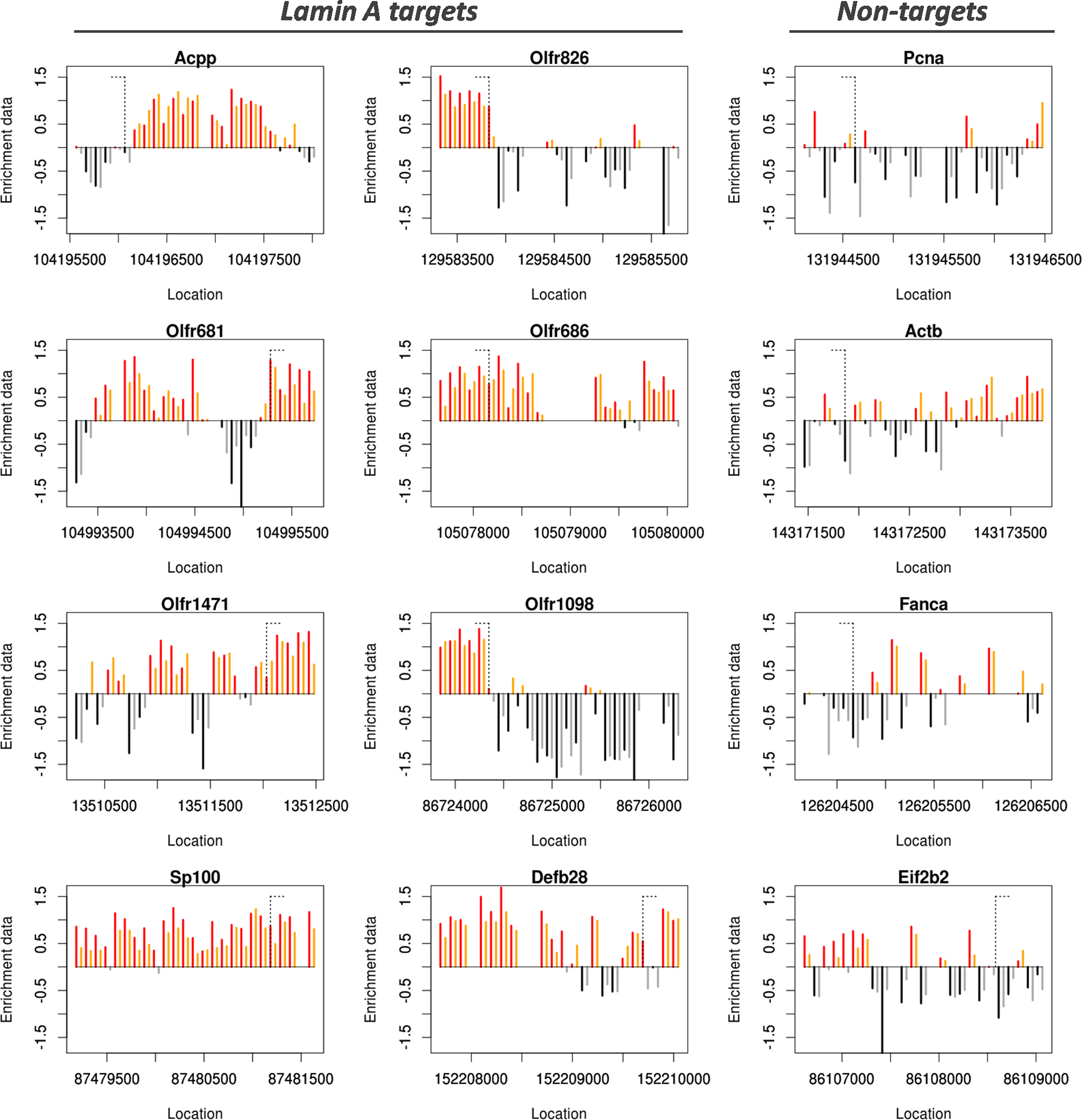

Supplement: Supplementary file 6 — High resolution image (TIFF 17086 kb) [file 412_2012_376_MOESM3_ESM.tif]

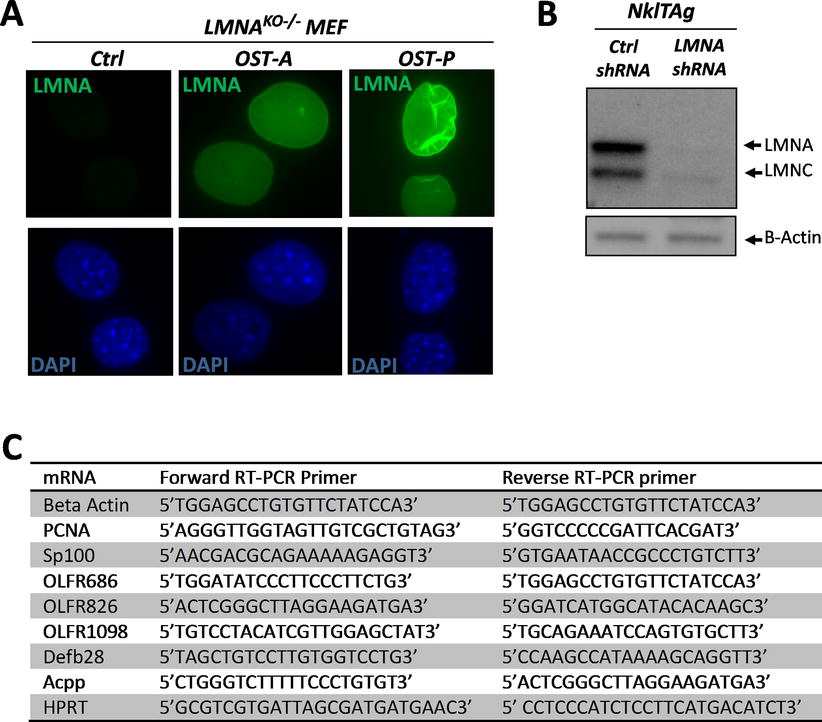

Supplement: Supplementary file 7 — (JPEG 94 kb) [file 412_2012_376_Fig9_ESM.jpg]

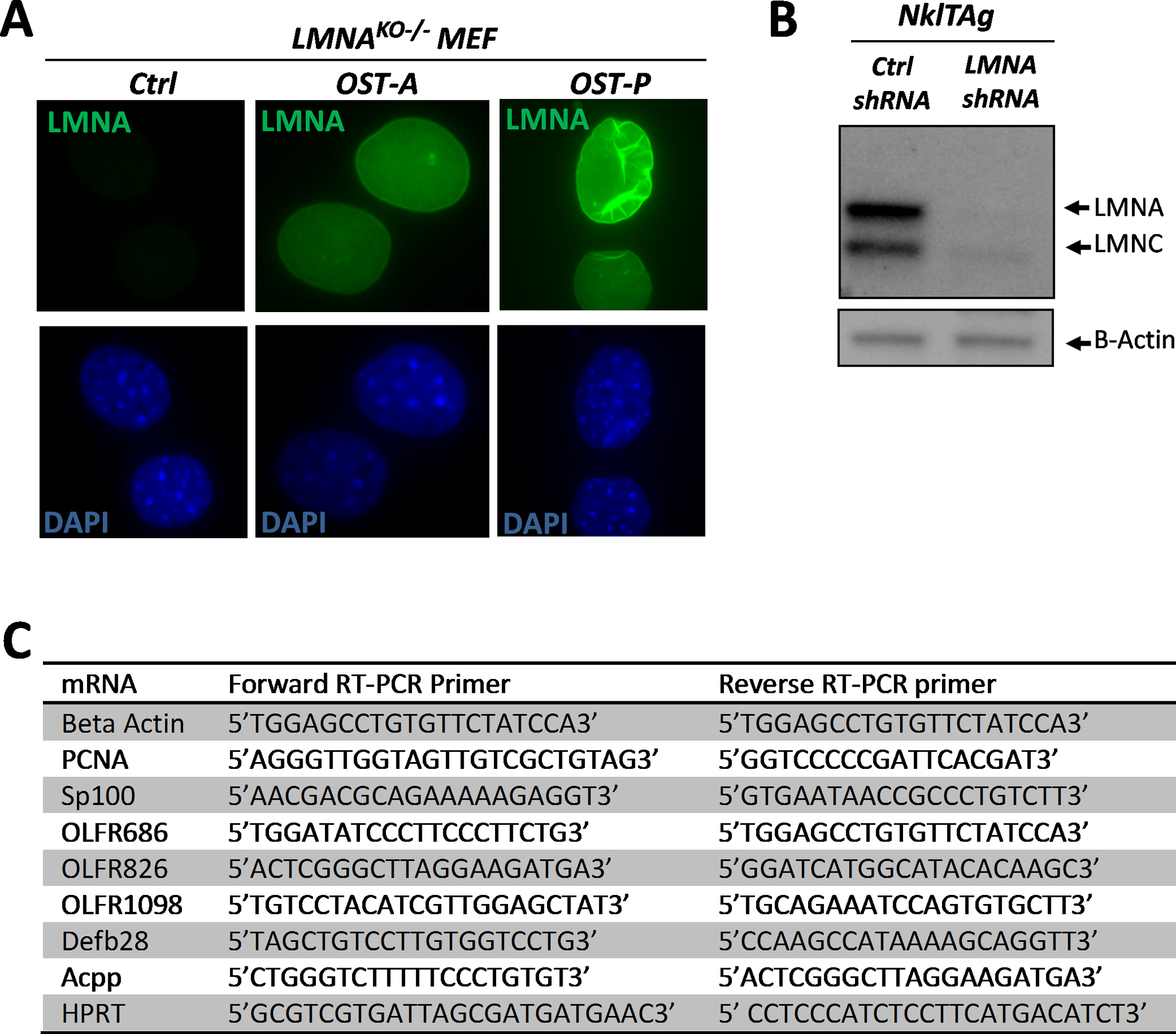

Supplement: Supplementary file 8 — High resolution image (TIFF 14509 kb) [file 412_2012_376_MOESM4_ESM.tif]

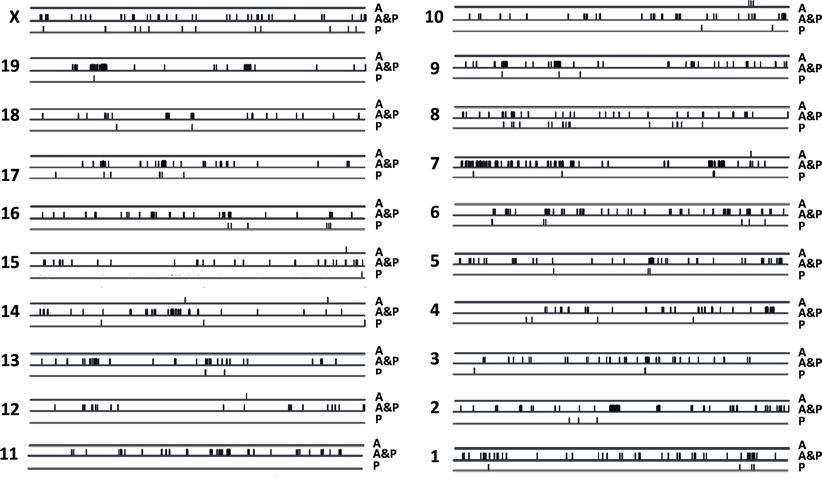

Supplement: Supplementary file 9 — (JPEG 62 kb) [file 412_2012_376_Fig10_ESM.jpg]

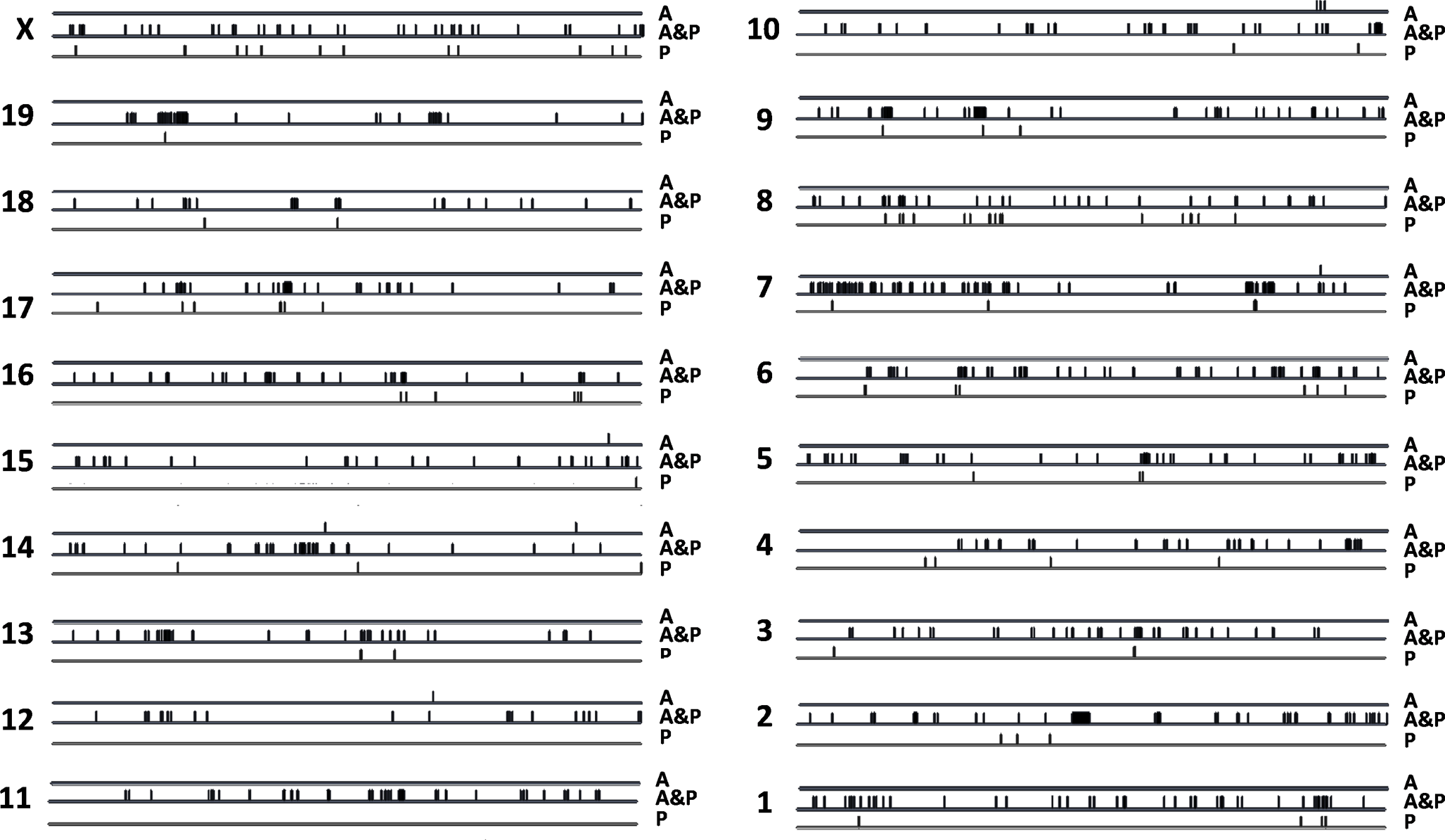

Supplement: Supplementary file 10 — High resolution image (TIFF 9603 kb) [file 412_2012_376_MOESM5_ESM.tif]

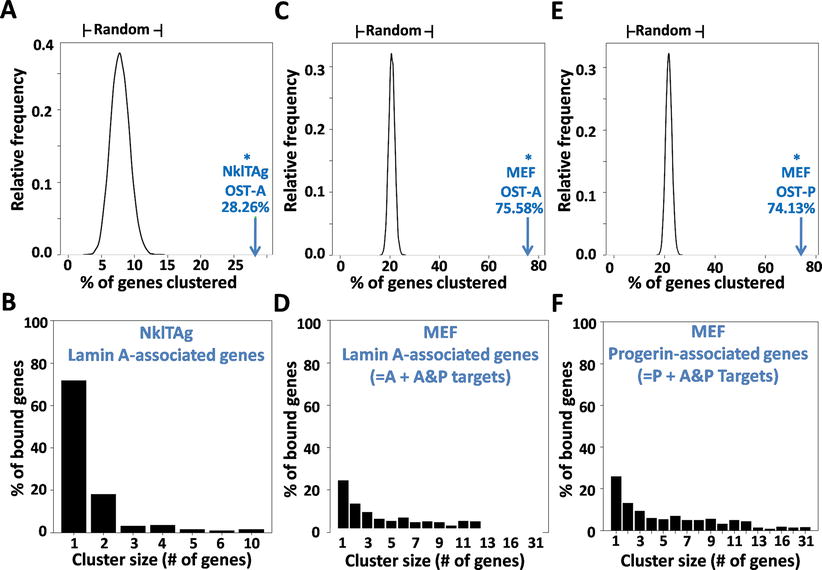

Supplement: Supplementary file 11 — (JPEG 64 kb) [file 412_2012_376_Fig11_ESM.jpg]

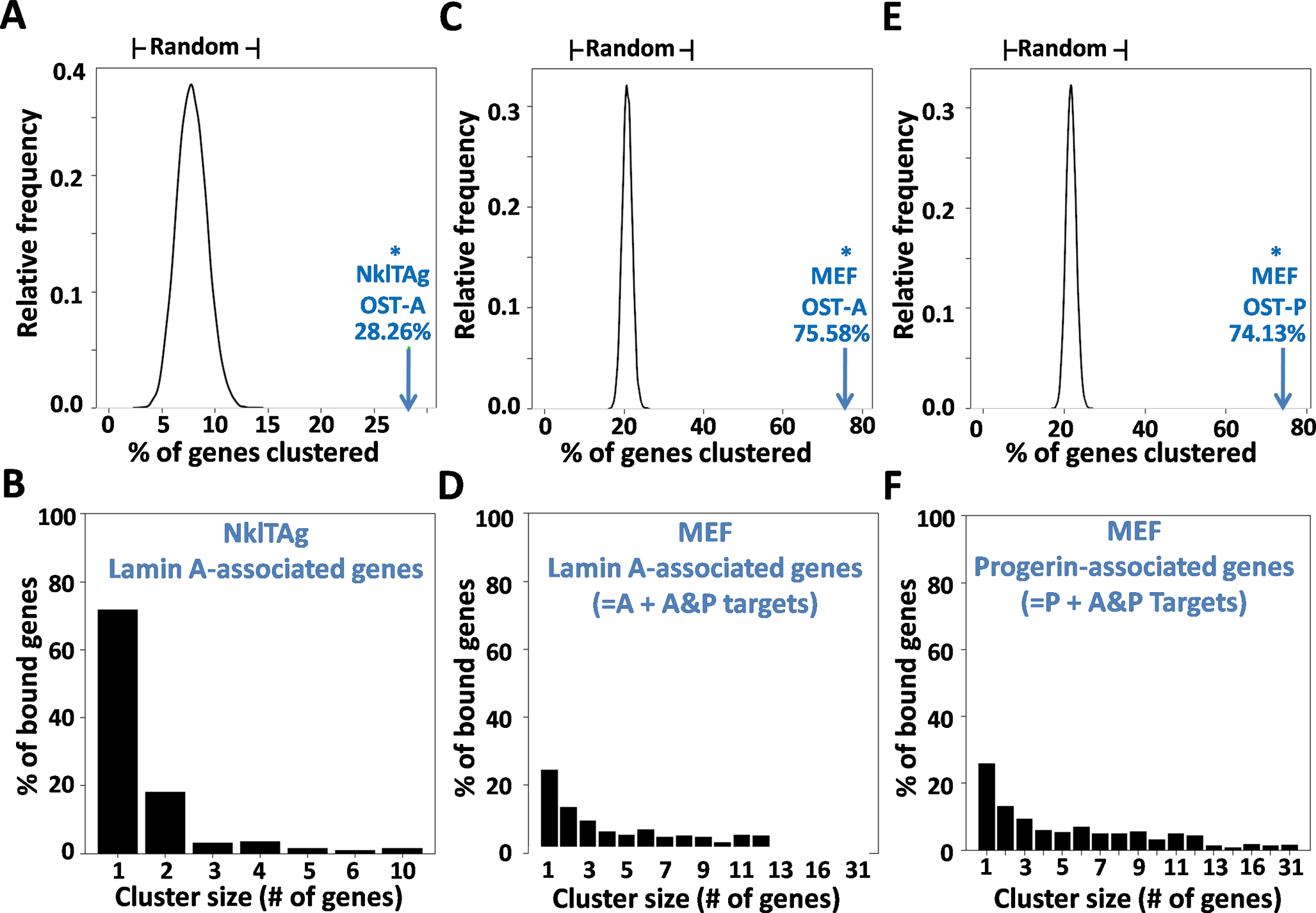

Supplement: Supplementary file 12 — High resolution image (TIFF 11457 kb) [file 412_2012_376_MOESM6_ESM.tif]

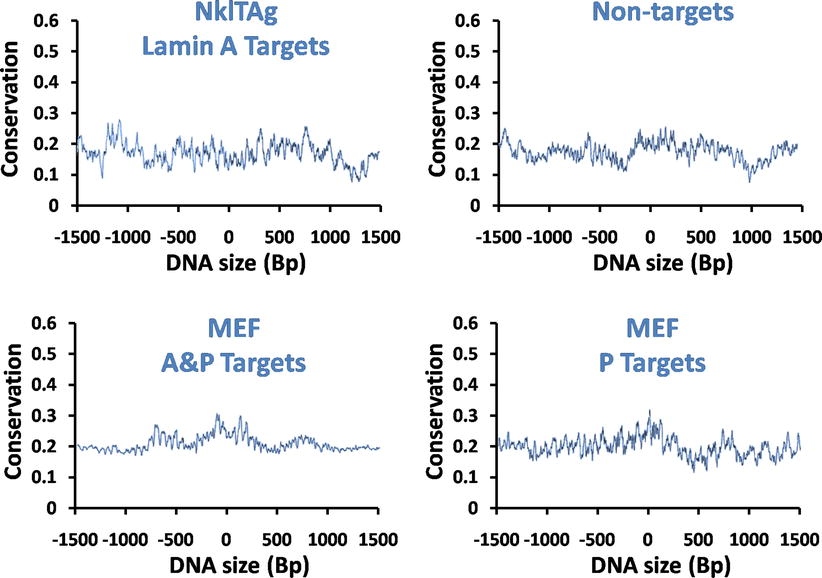

Supplement: Supplementary file 13 — (JPEG 63 kb) [file 412_2012_376_Fig12_ESM.jpg]

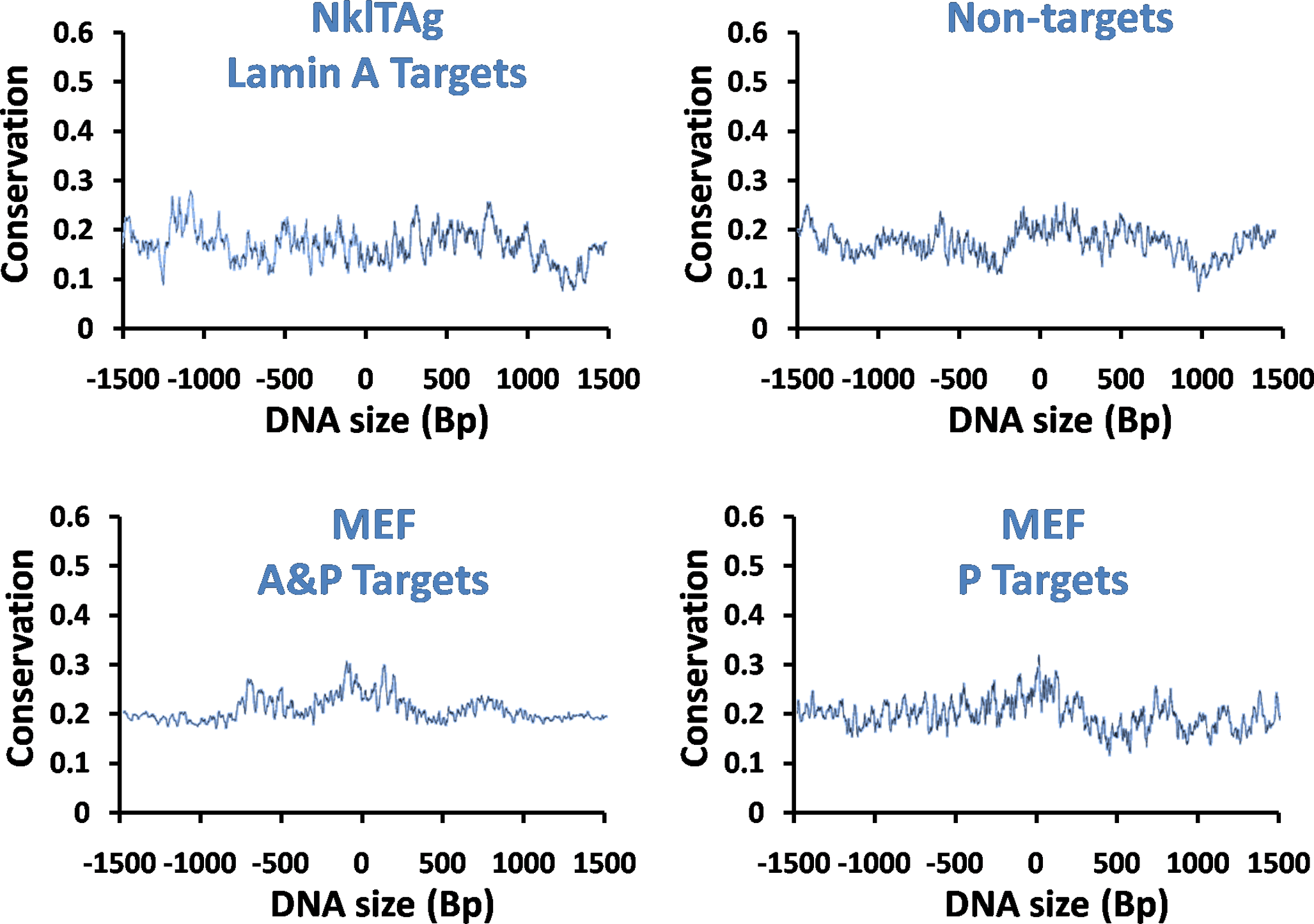

Supplement: Supplementary file 14 — High resolution image (TIFF 11610 kb) [file 412_2012_376_MOESM7_ESM.tif]
