# Supplementary material for: Mapping of lamin A- and progerin-interacting genome regions
Source: Chromosoma. 2012 May 19;121(5):447–64. doi: 10.1007/s00412-012-0376-7 (PMC3443488; doi:10.1007/s00412-012-0376-7)
Supplement: Supplementary file 15 — (DOCX 325 kb) [file 412_2012_376_MOESM8_ESM.docx]

**Table S1: ChIP data of all identified lamin A- and progerin-associated gene promoters**

| Entrez Gene ID | Gene Abbreviation | NklTAg Target type in NKLTAG | MEF Target type | Chrom | Start | End |
| --- | --- | --- | --- | --- | --- | --- |
| 98388 | Chst10 | NKLTAG A | NONE | chr1 | 38842206 | 38844706 |
| 71872 | Aox4 | NKLTAG A | NONE | chr1 | 58153015 | 58155515 |
| 21958 | Tnp1 | NKLTAG A | NONE | chr1 | 72948579 | 72951079 |
| 14767 | Nmur1 | NKLTAG A | NONE | chr1 | 88218812 | 88221312 |
| 20215 | Sag | NKLTAG A | NONE | chr1 | 89632850 | 89635350 |
| 11611 | Agxt | NKLTAG A | NONE | chr1 | 94963650 | 94966150 |
| 17248 | Mdm4 | NKLTAG A | NONE | chr1 | 134852394 | 134854894 |
| 29809 | Rabgap1l | NKLTAG A | NONE | chr1 | 162187748 | 162190248 |
| 17926 | Myoc | NKLTAG A | NONE | chr1 | 164473843 | 164476343 |
| 20344 | Selp | NKLTAG A | NONE | chr1 | 165950052 | 165952552 |
| 14194 | Fh1 | NKLTAG A | NONE | chr1 | 177461753 | 177464253 |
| 98256 | Kmo | NKLTAG A | NONE | chr1 | 177466867 | 177469367 |
| 22283 | Ush2a | NKLTAG A | NONE | chr1 | 189961821 | 189964321 |
| 17221 | Cd46 | NKLTAG A | NONE | chr1 | 196792472 | 196794972 |
| 56533 | Rgs17 | NKLTAG A | NONE | chr10 | 4422199 | 4424699 |
| 619323 | F730021E23Rik | NKLTAG A | NONE | chr10 | 20235444 | 20237944 |
| 22361 | Vnn1 | NKLTAG A | NONE | chr10 | 23571040 | 23573540 |
| 215854 | Taar5 | NKLTAG A | NONE | chr10 | 23658121 | 23660621 |
| 209517 | Taar7b | NKLTAG A | NONE | chr10 | 23687354 | 23689854 |
| 435206 | Taar7d | NKLTAG A | NONE | chr10 | 23714637 | 23717137 |
| 276742 | Taar7e | NKLTAG A | NONE | chr10 | 23725029 | 23727529 |
| 435207 | Taar7f | NKLTAG A | NONE | chr10 | 23736925 | 23739425 |
| 215859 | Taar8a | NKLTAG A | NONE | chr10 | 23763915 | 23766415 |
| 494546 | Taar8c | NKLTAG A | NONE | chr10 | 23790828 | 23793328 |
| 503558 | Taar9 | NKLTAG A | NONE | chr10 | 23798450 | 23800950 |
| 210757 | E430004N04Rik | NKLTAG A | NONE | chr10 | 28355810 | 28358310 |
| 72580 | 2700019D07Rik | NKLTAG A | NONE | chr10 | 33647707 | 33650207 |
| 215900 | A630077B13Rik | NKLTAG A | NONE | chr10 | 33816888 | 33819388 |
| 213402 | Armc2 | NKLTAG A | NONE | chr10 | 41664659 | 41667159 |
| 54198 | Snx3 | NKLTAG A | NONE | chr10 | 42188443 | 42190943 |
| 14806 | Grik2 | NKLTAG A | NONE | chr10 | 49471324 | 49473824 |
| 107449 | Unc5b | NKLTAG A | NONE | chr10 | 60226438 | 60228938 |
| 65971 | 1700021K02Rik | NKLTAG A | NONE | chr10 | 60565342 | 60567842 |
| 65971 | 1700021K02Rik | NKLTAG A | NONE | chr10 | 60568618 | 60571118 |
| 16694 | Krtap12-1 | NKLTAG A | NONE | chr10 | 77162301 | 77164801 |
| 59004 | Pias4 | NKLTAG A | NONE | chr10 | 80570552 | 80573052 |
| 103425 | Ncln\|Edg6 | NKLTAG A | NONE | chr10 | 80898985 | 80901485 |
| 13611 | Edg6 | NKLTAG A | NONE | chr10 | 80902766 | 80905266 |
| 74007 | Btbd11 | NKLTAG A | NONE | chr10 | 84815617 | 84818117 |
| 22027 | Hsp90b1\|Gnn | NKLTAG A | NONE | chr10 | 86133610 | 86137243 |
| 20728 | Spic | NKLTAG A | NONE | chr10 | 88112321 | 88114821 |
| 14421 | B4galnt1 | NKLTAG A | NONE | chr10 | 126566173 | 126568673 |
| 16325 | Inhbc | NKLTAG A | NONE | chr10 | 126772879 | 126775379 |
| 69917 | Obfc2b\|Rnf41 | NKLTAG A | NONE | chr10 | 127812245 | 127815105 |
| 18373 | Olfr9 | NKLTAG A | NONE | chr10 | 128390862 | 128393362 |
| 258861 | Olfr763 | NKLTAG A | NONE | chr10 | 128412235 | 128414735 |
| 258266 | Olfr247 | NKLTAG A | NONE | chr10 | 129378016 | 129380516 |
| 194974 | Sunc1\|RP23-219P13.3 | NKLTAG A | NONE | chr11 | 8946594 | 8950994 |
| 13195 | Ddc | NKLTAG A | NONE | chr11 | 11780157 | 11782657 |
| 216616 | Efemp1 | NKLTAG A | NONE | chr11 | 28765772 | 28768272 |
| 15122 | Hba-a1\|F830116E18Rik | NKLTAG A | NONE | chr11 | 32184963 | 32187463 |
| 16160 | Il12b | NKLTAG A | NONE | chr11 | 44241485 | 44243985 |
| 214105 | Sox30 | NKLTAG A | NONE | chr11 | 45821732 | 45824232 |
| 432552 | RP23-273O7.4 | NKLTAG A | NONE | chr11 | 46246152 | 46248652 |
| 347708 | Dppa1 | NKLTAG A | NONE | chr11 | 46472184 | 46474684 |
| 258464 | Olfr1384 | NKLTAG A | NONE | chr11 | 49355062 | 49357562 |
| 258461 | Olfr1381 | NKLTAG A | NONE | chr11 | 49393171 | 49395671 |
| 404336 | Olfr1380 | NKLTAG A | NONE | chr11 | 49405345 | 49407845 |
| 19127 | Prop1 | NKLTAG A | NONE | chr11 | 50796555 | 50799055 |
| 21408 | Zfp354a | NKLTAG A | NONE | chr11 | 50900679 | 50903179 |
| 21414 | Tcf7 | NKLTAG A | NONE | chr11 | 52125494 | 52127994 |
| 14799 | Gria1 | NKLTAG A | NONE | chr11 | 56853378 | 56855878 |
| 268417 | Zfp496\|4933439C10Rik | NKLTAG A | NONE | chr11 | 59321606 | 59324106 |
| 13855 | Epn2 | NKLTAG A | NONE | chr11 | 61395346 | 61397846 |
| 68460 | Dhrs7c | NKLTAG A | NONE | chr11 | 67612482 | 67614982 |
| 104709 | BB220380 | NKLTAG A | NONE | chr11 | 68317285 | 68319785 |
| 18626 | Per1 | NKLTAG A | NONE | chr11 | 68913143 | 68915643 |
| 108803 | 4933402P03Rik | NKLTAG A | NONE | chr11 | 69634135 | 69636635 |
| 56486 | Gabarap | NKLTAG A | NONE | chr11 | 69805564 | 69808064 |
| 17312 | Mgl1 | NKLTAG A | NONE | chr11 | 69980968 | 69983468 |
| 103844 | AI842396\|Kif1c | NKLTAG A | NONE | chr11 | 70515850 | 70519494 |
| 237831 | Slc13a5 | NKLTAG A | NONE | chr11 | 72082260 | 72084760 |
| 280408 | Rilp\|Scarf1 | NKLTAG A | NONE | chr11 | 75327735 | 75330235 |
| 18738 | Pitpna | NKLTAG A | NONE | chr11 | 75402302 | 75404802 |
| 19193 | Pipox | NKLTAG A | NONE | chr11 | 77709479 | 77711979 |
| 20500 | Slc13a2 | NKLTAG A | NONE | chr11 | 78237880 | 78240380 |
| 216991 | Centa2 | NKLTAG A | NONE | chr11 | 79968356 | 79970856 |
| 16869 | Lhx1 | NKLTAG A | NONE | chr11 | 84341229 | 84343729 |
| 77864 | Ypel2 | NKLTAG A | NONE | chr11 | 86787704 | 86790204 |
| 17523 | Mpo | NKLTAG A | NONE | chr11 | 87607775 | 87610275 |
| 380718 | Mks1 | NKLTAG A | NONE | chr11 | 87667441 | 87669941 |
| 544809 | LOC544809 | NKLTAG A | NONE | chr11 | 95514733 | 95517233 |
| 15416 | Hoxb8 | NKLTAG A | NONE | chr11 | 96095994 | 96098494 |
| 450219 | Gsdm3 | NKLTAG A | NONE | chr11 | 98440449 | 98442949 |
| 69664 | 2310043L02Rik\|LOC629873 | NKLTAG A | NONE | chr11 | 99396606 | 99399106 |
| 629873 | LOC629873 | NKLTAG A | NONE | chr11 | 99399267 | 99401767 |
| 16670 | Krt1-2 | NKLTAG A | NONE | chr11 | 99903705 | 99906205 |
| 20533 | Slc4a1 | NKLTAG A | NONE | chr11 | 102180705 | 102183205 |
| 56494 | Gosr2 | NKLTAG A | NONE | chr11 | 103513284 | 103515784 |
| 16518 | Kcnj2 | NKLTAG A | NONE | chr11 | 110880253 | 110882753 |
| 71203 | 4933434M16Rik | NKLTAG A | NONE | chr11 | 112639268 | 112641768 |
| 72014 | 1500005I02Rik\|Gpr142 | NKLTAG A | NONE | chr11 | 114611482 | 114615513 |
| 58222 | Rab37 | NKLTAG A | NONE | chr11 | 114968274 | 114970774 |
| 27029 | Sgsh\|Slc26a11 | NKLTAG A | NONE | chr11 | 119169646 | 119173592 |
| 209011 | Sirt7 | NKLTAG A | NONE | chr11 | 120440557 | 120443057 |
| 194655 | Tieg3 | NKLTAG A | NONE | chr12 | 25238352 | 25240852 |
| 217480 | Dgkb | NKLTAG A | NONE | chr12 | 38388970 | 38391470 |
| 18511 | Pax9 | NKLTAG A | NONE | chr12 | 57611650 | 57614150 |
| 238266 | Syt16 | NKLTAG A | NONE | chr12 | 74914598 | 74917098 |
| 214321 | Adam4b | NKLTAG A | NONE | chr12 | 82297730 | 82300230 |
| 217695 | Zfyve1 | NKLTAG A | NONE | chr12 | 84484142 | 84486642 |
| 19179 | Psmc1 | NKLTAG A | NONE | chr12 | 100509251 | 100511751 |
| 12401 | Serpina6 | NKLTAG A | NONE | chr12 | 104057621 | 104060121 |
| 20701 | Serpina1b | NKLTAG A | NONE | chr12 | 104138739 | 104141239 |
| 71907 | Serpina9 | NKLTAG A | NONE | chr12 | 104414202 | 104416702 |
| 105245 | Txndc5 | NKLTAG A | NONE | chr13 | 38535426 | 38537926 |
| 17152 | Mak | NKLTAG A | NONE | chr13 | 41080933 | 41083433 |
| 77117 | 6720457D02Rik | NKLTAG A | NONE | chr13 | 62567722 | 62570222 |
| 68271 | 4930441O14Rik | NKLTAG A | NONE | chr13 | 68241306 | 68243806 |
| 79401 | Spz1 | NKLTAG A | NONE | chr13 | 93676425 | 93678925 |
| 67295 | Rab3c | NKLTAG A | NONE | chr13 | 111400480 | 111402980 |
| 14945 | Gzmk | NKLTAG A | NONE | chr13 | 114301275 | 114303775 |
| 545007 | LOC545007 | NKLTAG A | NONE | chr14 | 4150026 | 4152526 |
| 544988 | LOC544988 | NKLTAG A | NONE | chr14 | 5895366 | 5897866 |
| 258387 | Olfr720 | NKLTAG A | NONE | chr14 | 12968847 | 12971347 |
| 68045 | 2700060E02Rik | NKLTAG A | NONE | chr14 | 18614019 | 18616519 |
| 59011 | Myoz1\|Synpo2l | NKLTAG A | NONE | chr14 | 19454148 | 19458858 |
| 73068 | Fut11 | NKLTAG A | NONE | chr14 | 19481519 | 19484019 |
| 18792 | Plau | NKLTAG A | NONE | chr14 | 19623258 | 19625758 |
| 435391 | LOC435391 | NKLTAG A | NONE | chr14 | 20502628 | 20505128 |
| 268723 | A830039N20Rik | NKLTAG A | NONE | chr14 | 22081695 | 22084195 |
| 77592 | 4931406H21Rik | NKLTAG A | NONE | chr14 | 24417987 | 24420487 |
| 218865 | Chdh | NKLTAG A | NONE | chr14 | 28848311 | 28852141 |
| 93725 | Ear10 | NKLTAG A | NONE | chr14 | 42802735 | 42805235 |
| 140806 | Il17e | NKLTAG A | NONE | chr14 | 53884979 | 53887479 |
| 140806 | Il17e\|Cmtm5 | NKLTAG A | NONE | chr14 | 53888678 | 53891178 |
| 67881 | 1810034K20Rik | NKLTAG A | NONE | chr14 | 54613985 | 54616485 |
| 12684 | Cideb\|Ltb4r2 | NKLTAG A | NONE | chr14 | 54713994 | 54716494 |
| 12684 | Cideb\|Ltb4r2\|Ltb4r1 | NKLTAG A | NONE | chr14 | 54716788 | 54720586 |
| 14939 | Gzmb | NKLTAG A | NONE | chr14 | 55215831 | 55218331 |
| 56471 | Stmn4 | NKLTAG A | NONE | chr14 | 65296484 | 65298984 |
| 93688 | Klhl1 | NKLTAG A | NONE | chr14 | 95401668 | 95404168 |
| 212085 | Trim52 | NKLTAG A | NONE | chr14 | 104989803 | 104992303 |
| 239283 | Oxgr1 | NKLTAG A | NONE | chr14 | 119177117 | 119179617 |
| 223254 | Farp1 | NKLTAG A | NONE | chr14 | 120235253 | 120237753 |
| 23880 | Fyb | NKLTAG A | NONE | chr15 | 6525085 | 6527585 |
| 14000 | Rnasen | NKLTAG A | NONE | chr15 | 12776614 | 12779114 |
| 239368 | BC030476 | NKLTAG A | NONE | chr15 | 34395900 | 34398400 |
| 64705 | Dpys | NKLTAG A | NONE | chr15 | 39687041 | 39689541 |
| 67552 | H2afy3 | NKLTAG A | NONE | chr15 | 62048569 | 62051069 |
| 13072 | Cyp11b2 | NKLTAG A | NONE | chr15 | 74682980 | 74685480 |
| 67038 | 2010109I03Rik | NKLTAG A | NONE | chr15 | 74708459 | 74710959 |
| 110454 | Ly6a | NKLTAG A | NONE | chr15 | 74824643 | 74827143 |
| 223646 | Naprt1 | NKLTAG A | NONE | chr15 | 75721236 | 75723736 |
| 75475 | Oplah | NKLTAG A | NONE | chr15 | 76134000 | 76136500 |
| 328561 | 9130218O11Rik | NKLTAG A | NONE | chr15 | 77422851 | 77425351 |
| 19293 | Pvalb | NKLTAG A | NONE | chr15 | 78033106 | 78035606 |
| 17972 | Ncf4 | NKLTAG A | NONE | chr15 | 78070088 | 78072588 |
| 16185 | Il2rb | NKLTAG A | NONE | chr15 | 78321777 | 78324277 |
| 14958 | H1f0 | NKLTAG A | NONE | chr15 | 78853466 | 78855966 |
| 14958 | H1f0\|Gcat | NKLTAG A | NONE | chr15 | 78856174 | 78858674 |
| 16520 | Kcnj4 | NKLTAG A | NONE | chr15 | 79336240 | 79338740 |
| 67130 | Ndufa6 | NKLTAG A | NONE | chr15 | 82181506 | 82184006 |
| 56448 | Cyp2d22 | NKLTAG A | NONE | chr15 | 82207426 | 82209926 |
| 68607 | Serhl | NKLTAG A | NONE | chr15 | 82925980 | 82928480 |
| 72026 | Trmu | NKLTAG A | NONE | chr15 | 85705092 | 85707592 |
| 402773 | D030018L15Rik | NKLTAG A | NONE | chr15 | 95907087 | 95909587 |
| 18642 | Pfkm | NKLTAG A | NONE | chr15 | 97938154 | 97940654 |
| 223887 | BC032281 | NKLTAG A | NONE | chr15 | 98691943 | 98694443 |
| 11830 | Aqp5 | NKLTAG A | NONE | chr15 | 99416813 | 99419313 |
| 11831 | Aqp6 | NKLTAG A | NONE | chr15 | 99427433 | 99429933 |
| 20273 | Scn8a | NKLTAG A | NONE | chr15 | 100762358 | 100764858 |
| 66322 | 1700011A15Rik | NKLTAG A | NONE | chr15 | 101273807 | 101276307 |
| 16679 | Krt2-10 | NKLTAG A | NONE | chr15 | 101299511 | 101302011 |
| 16682 | Krt2-4 | NKLTAG A | NONE | chr15 | 101752245 | 101754745 |
| 332131 | 2310030B04Rik | NKLTAG A | NONE | chr15 | 101781758 | 101784258 |
| 67824 | 1110025F24Rik | NKLTAG A | NONE | chr16 | 4633787 | 4636287 |
| 74482 | Ifitm7\|2900011O08Rik | NKLTAG A | NONE | chr16 | 13897699 | 13900738 |
| 67203 | Nde1 | NKLTAG A | NONE | chr16 | 14081052 | 14083552 |
| 224129 | Adcy5 | NKLTAG A | NONE | chr16 | 35072623 | 35075123 |
| 239845 | Gpr156 | NKLTAG A | NONE | chr16 | 37833734 | 37836234 |
| 64082 | Popdc2 | NKLTAG A | NONE | chr16 | 38279483 | 38281983 |
| 224405 | Cyyr1 | NKLTAG A | NONE | chr16 | 85439069 | 85441569 |
| 170651 | Krtap16-1\|Krtap16-5 | NKLTAG A | NONE | chr16 | 88762628 | 88765128 |
| 77918 | Krtap16-5 | NKLTAG A | NONE | chr16 | 88766349 | 88768849 |
| 16704 | Krtap8-2 | NKLTAG A | NONE | chr16 | 88784808 | 88787308 |
| 109857 | Cbr3 | NKLTAG A | NONE | chr16 | 93569758 | 93572258 |
| 56173 | Cldn14 | NKLTAG A | NONE | chr16 | 93896877 | 93899377 |
| 77106 | Gpr178 | NKLTAG A | NONE | chr17 | 6378412 | 6380912 |
| 72536 | Tagap | NKLTAG A | NONE | chr17 | 7762193 | 7764693 |
| 320111 | 9630019K15Rik | NKLTAG A | NONE | chr17 | 8176579 | 8179079 |
| 71682 | Wdr27\|1600012H06Rik | NKLTAG A | NONE | chr17 | 14677759 | 14681700 |
| 245847 | Amdhd2 | NKLTAG A | NONE | chr17 | 23890859 | 23893359 |
| 79044 | Mrps34\|Nme3 | NKLTAG A | NONE | chr17 | 24620719 | 24624605 |
| 17229 | Mcpt6 | NKLTAG A | NONE | chr17 | 25091932 | 25094432 |
| 14755 | Pigq\|LOC621239 | NKLTAG A | NONE | chr17 | 25669062 | 25671562 |
| 21784 | Tff1 | NKLTAG A | NONE | chr17 | 30891534 | 30894034 |
| 22092 | Tsga2 | NKLTAG A | NONE | chr17 | 31005566 | 31008066 |
| 18771 | Pknox1 | NKLTAG A | NONE | chr17 | 31310405 | 31312905 |
| 320997 | 4732474A20Rik | NKLTAG A | NONE | chr17 | 32184857 | 32187357 |
| 70101 | Cyp4f16 | NKLTAG A | NONE | chr17 | 32269862 | 32272362 |
| 240063 | BC052046 | NKLTAG A | NONE | chr17 | 32548284 | 32550784 |
| 54218 | B3galt4 | NKLTAG A | NONE | chr17 | 33561433 | 33563933 |
| 547431 | Btnl2 | NKLTAG A | NONE | chr17 | 33960402 | 33962902 |
| 54197 | Rnf5 | NKLTAG A | NONE | chr17 | 34211117 | 34213617 |
| 114654 | Ly6g6d\|Ly6g6e | NKLTAG A | NONE | chr17 | 34682020 | 34685497 |
| 21926 | Tnf\|Lta | NKLTAG A | NONE | chr17 | 34809548 | 34812048 |
| 16992 | Lta | NKLTAG A | NONE | chr17 | 34812735 | 34815235 |
| 22024 | Crisp2 | NKLTAG A | NONE | chr17 | 40257074 | 40259574 |
| 75564 | 1700027N10Rik | NKLTAG A | NONE | chr17 | 45607279 | 45609779 |
| 74094 | Tjap1 | NKLTAG A | NONE | chr17 | 45746094 | 45748594 |
| 107971 | Frs3 | NKLTAG A | NONE | chr17 | 47156397 | 47158897 |
| 76441 | Daam2 | NKLTAG A | NONE | chr17 | 49029420 | 49031920 |
| 211468 | Kcnh8 | NKLTAG A | NONE | chr17 | 52065691 | 52068191 |
| 50498 | Ebi3 | NKLTAG A | NONE | chr17 | 55543915 | 55546415 |
| 70785 | Dennd1c | NKLTAG A | NONE | chr17 | 56763263 | 56765763 |
| 21948 | Tnfsf7 | NKLTAG A | NONE | chr17 | 56834610 | 56837110 |
| 68617 | 1110012J17Rik | NKLTAG A | NONE | chr17 | 66290355 | 66292855 |
| 17929 | Myom1 | NKLTAG A | NONE | chr17 | 70922377 | 70924877 |
| 22436 | Xdh | NKLTAG A | NONE | chr17 | 73854581 | 73857081 |
| 94224 | Srd5a2 | NKLTAG A | NONE | chr17 | 73952314 | 73954814 |
| 50766 | Crim1 | NKLTAG A | NONE | chr17 | 78100125 | 78102625 |
| 77868 | Six3os1\|Six3 | NKLTAG A | NONE | chr17 | 85526191 | 85529659 |
| 93885 | Pcdhb14 | NKLTAG A | NONE | chr18 | 37571630 | 37574130 |
| 67365 | Hdhd1a | NKLTAG A | NONE | chr18 | 50693361 | 50695861 |
| 54445 | Unc93b1 | NKLTAG A | NONE | chr19 | 3933218 | 3935718 |
| 11632 | Aip\|Tmem134 | NKLTAG A | NONE | chr19 | 4123986 | 4127827 |
| 19265 | Ptprcap | NKLTAG A | NONE | chr19 | 4152645 | 4155145 |
| 52028 | Bbs1\|Dpp3 | NKLTAG A | NONE | chr19 | 4906112 | 4908612 |
| 240514 | Ccdc85b\|Fibp | NKLTAG A | NONE | chr19 | 5457049 | 5461193 |
| 12507 | Cd5\|A430093F15Rik | NKLTAG A | NONE | chr19 | 10805519 | 10808491 |
| 258677 | Olfr76 | NKLTAG A | NONE | chr19 | 12187219 | 12189719 |
| 226025 | Trpm3 | NKLTAG A | NONE | chr19 | 22204213 | 22206713 |
| 13380 | Dkk1 | NKLTAG A | NONE | chr19 | 30614993 | 30617493 |
| 19091 | Prkg1 | NKLTAG A | NONE | chr19 | 31729867 | 31732367 |
| 226144 | Spfh1 | NKLTAG A | NONE | chr19 | 44122336 | 44124836 |
| 70806 | D19Ertd652e\|BC063749 | NKLTAG A | NONE | chr19 | 47972092 | 47974592 |
| 67507 | 1700019N19Rik | NKLTAG A | NONE | chr19 | 58847164 | 58849664 |
| 73442 | Hspa12a | NKLTAG A | NONE | chr19 | 58913695 | 58916195 |
| 17533 | Mrc1 | NKLTAG A | NONE | chr2 | 14145167 | 14147667 |
| 68231 | 1700113O17Rik | NKLTAG A | NONE | chr2 | 17914200 | 17916700 |
| 74295 | 1700092C17Rik | NKLTAG A | NONE | chr2 | 22605630 | 22608130 |
| 227648 | AU024582 | NKLTAG A | NONE | chr2 | 26249855 | 26252355 |
| 11350 | Abl1 | NKLTAG A | NONE | chr2 | 31579990 | 31582490 |
| 21375 | Tbr1 | NKLTAG A | NONE | chr2 | 61603291 | 61605791 |
| 545428 | 2610301F02Rik | NKLTAG A | NONE | chr2 | 76970975 | 76973475 |
| 18573 | Pde1a | NKLTAG A | NONE | chr2 | 79708738 | 79711238 |
| 20378 | Frzb | NKLTAG A | NONE | chr2 | 80247735 | 80250235 |
| 258571 | Olfr1033 | NKLTAG A | NONE | chr2 | 85819478 | 85821978 |
| 18347 | Olfr48 | NKLTAG A | NONE | chr2 | 89645310 | 89647810 |
| 320600 | A930104D05Rik | NKLTAG A | NONE | chr2 | 117967110 | 117969610 |
| 74176 | Tgm5 | NKLTAG A | NONE | chr2 | 120776700 | 120779200 |
| 18798 | Plcb4 | NKLTAG A | NONE | chr2 | 135431270 | 135433770 |
| 20614 | Snap25 | NKLTAG A | NONE | chr2 | 136402943 | 136405443 |
| 58214 | Cst10 | NKLTAG A | NONE | chr2 | 149094689 | 149097189 |
| 442835 | 9230002F21Rik | NKLTAG A | NONE | chr2 | 152181079 | 152183579 |
| 67701 | Wfdc2 | NKLTAG A | NONE | chr2 | 164251920 | 164254420 |
| 277345 | Wfdc16 | NKLTAG A | NONE | chr2 | 164329507 | 164332007 |
| 629754 | LOC629754\|LOC629756 | NKLTAG A | NONE | chr2 | 164345250 | 164347750 |
| 381405 | Gm1008 | NKLTAG A | NONE | chr2 | 165052824 | 165055324 |
| 228880 | N/A\|Prkcbp1 | NKLTAG A | NONE | chr2 | 165587655 | 165590155 |
| 20459 | Ptk6 | NKLTAG A | NONE | chr2 | 181131696 | 181134196 |
| 18389 | Oprl1 | NKLTAG A | NONE | chr2 | 181642449 | 181644949 |
| 17116 | Mab21l1 | NKLTAG A | NONE | chr3 | 55868453 | 55870953 |
| 23937 | Mab21l2\|Lrba\|LOC667024 | NKLTAG A | NONE | chr3 | 86633710 | 86636210 |
| 229499 | Fcrl1 | NKLTAG A | NONE | chr3 | 87460374 | 87462886 |
| 23897 | Hax1 | NKLTAG A | NONE | chr3 | 90084113 | 90086613 |
| 73730 | 1110008K04Rik | NKLTAG A | NONE | chr3 | 92936189 | 92938689 |
| 545548 | LOC545548 | NKLTAG A | NONE | chr3 | 93011133 | 93013633 |
| 69269 | Scnm1\|Lysmd1 | NKLTAG A | NONE | chr3 | 95217513 | 95221392 |
| 59020 | Pdzk1 | NKLTAG A | NONE | chr3 | 96913530 | 96916030 |
| 77578 | Bcl9 | NKLTAG A | NONE | chr3 | 97301412 | 97303912 |
| 18072 | Nhlh2 | NKLTAG A | NONE | chr3 | 102137215 | 102139715 |
| 258482 | Olfr266 | NKLTAG A | NONE | chr3 | 106950113 | 106952613 |
| 13171 | Dbt | NKLTAG A | NONE | chr3 | 116503144 | 116505644 |
| 109676 | Ank2 | NKLTAG A | NONE | chr3 | 126989974 | 126992474 |
| 211556 | BC002199 | NKLTAG A | NONE | chr3 | 127800386 | 127802886 |
| 229927 | Clca4 | NKLTAG A | NONE | chr3 | 144786188 | 144788688 |
| 242253 | Wdr63 | NKLTAG A | NONE | chr3 | 146044922 | 146047422 |
| 242285 | Rdhe2 | NKLTAG A | NONE | chr4 | 3946310 | 3948810 |
| 76299 | Txndc4\|Invs | NKLTAG A | NONE | chr4 | 48298922 | 48302658 |
| 545648 | LOC545648\|Ifnz | NKLTAG A | NONE | chr4 | 88251361 | 88253861 |
| 21687 | Tek | NKLTAG A | NONE | chr4 | 94229317 | 94231817 |
| 100102 | Pcsk9 | NKLTAG A | NONE | chr4 | 105974461 | 105976961 |
| 230777 | Hcrtr1 | NKLTAG A | NONE | chr4 | 129640966 | 129643466 |
| 74772 | Atp13a2 | NKLTAG A | NONE | chr4 | 140257003 | 140259503 |
| 100198 | H6pd | NKLTAG A | NONE | chr4 | 148852323 | 148854823 |
| 433844 | LOC433844 | NKLTAG A | NONE | chr5 | 10237868 | 10240368 |
| 15234 | Hgf | NKLTAG A | NONE | chr5 | 16063373 | 16065873 |
| 70935 | Speer4f | NKLTAG A | NONE | chr5 | 16985945 | 16988445 |
| 101023 | Zfp513 | NKLTAG A | NONE | chr5 | 31478314 | 31480814 |
| 15476 | Hs3st1 | NKLTAG A | NONE | chr5 | 40043224 | 40045724 |
| 80334 | Kcnip4 | NKLTAG A | NONE | chr5 | 48797508 | 48800008 |
| 433886 | LOC433886 | NKLTAG A | NONE | chr5 | 52663389 | 52665889 |
| 12425 | Cckar | NKLTAG A | NONE | chr5 | 53995850 | 53998350 |
| 54403 | Slc4a4 | NKLTAG A | NONE | chr5 | 90007307 | 90009807 |
| 104443 | Npffr2 | NKLTAG A | NONE | chr5 | 90600627 | 90603127 |
| 73246 | Rassf6 | NKLTAG A | NONE | chr5 | 91715167 | 91717667 |
| 57349 | Cxcl7 | NKLTAG A | NONE | chr5 | 91841717 | 91844217 |
| 57349 | Cxcl7\|Cxcl4 | NKLTAG A | NONE | chr5 | 91845712 | 91848212 |
| 11745 | Anxa3 | NKLTAG A | NONE | chr5 | 97031716 | 97034216 |
| 72145 | Wdfy3\|LOC666515\|LOC666527 | NKLTAG A | NONE | chr5 | 102309723 | 102312223 |
| 231532 | Arhgap24 | NKLTAG A | NONE | chr5 | 102719692 | 102722347 |
| 13602 | Sparcl1 | NKLTAG A | NONE | chr5 | 104353506 | 104356006 |
| 13406 | Dmp1 | NKLTAG A | NONE | chr5 | 104440929 | 104443429 |
| 15891 | Ibsp | NKLTAG A | NONE | chr5 | 104537599 | 104540099 |
| 231549 | Lrrc8d | NKLTAG A | NONE | chr5 | 105932780 | 105935280 |
| 73318 | 1700013N18Rik | NKLTAG A | NONE | chr5 | 108068554 | 108071054 |
| 231605 | Galnt9 | NKLTAG A | NONE | chr5 | 110853786 | 110856286 |
| 231633 | Tmem119 | NKLTAG A | NONE | chr5 | 114060857 | 114063357 |
| 231655 | Oasl1 | NKLTAG A | NONE | chr5 | 115182238 | 115184738 |
| 18778 | Pla2g1b | NKLTAG A | NONE | chr5 | 115725264 | 115727764 |
| 231672 | Fbxw8 | NKLTAG A | NONE | chr5 | 118415915 | 118418415 |
| 252972 | Tpcn1\|Iqcd | NKLTAG A | NONE | chr5 | 120847639 | 120851230 |
| 15445 | Hpd | NKLTAG A | NONE | chr5 | 123442805 | 123445305 |
| 231727 | B3gnt4\|N/A | NKLTAG A | NONE | chr5 | 123769077 | 123771577 |
| 68184 | Denr | NKLTAG A | NONE | chr5 | 124165892 | 124168392 |
| 21871 | Atp6v0a2 | NKLTAG A | NONE | chr5 | 124999188 | 125001688 |
| 56743 | Lat2 | NKLTAG A | NONE | chr5 | 134899150 | 134901650 |
| 243300 | 6430598A04Rik | NKLTAG A | NONE | chr5 | 137969286 | 137971786 |
| 59031 | Chst12 | NKLTAG A | NONE | chr5 | 140756101 | 140758601 |
| 231871 | E330036I19Rik | NKLTAG A | NONE | chr5 | 143726562 | 143729062 |
| 66066 | Gng11 | NKLTAG A | NONE | chr6 | 3951986 | 3954486 |
| 18979 | Pon1 | NKLTAG A | NONE | chr6 | 5143324 | 5145824 |
| 12057 | Opn1sw | NKLTAG A | NONE | chr6 | 29329990 | 29332490 |
| 232670 | Tspan33 | NKLTAG A | NONE | chr6 | 29642265 | 29644765 |
| 20899 | Stra8 | NKLTAG A | NONE | chr6 | 34849111 | 34851611 |
| 319472 | 9330158H04Rik | NKLTAG A | NONE | chr6 | 36317503 | 36320003 |
| 243764 | Chrm2 | NKLTAG A | NONE | chr6 | 36451361 | 36453861 |
| 387514 | Tas2r143 | NKLTAG A | NONE | chr6 | 42327844 | 42330344 |
| 387512 | Tas2r135 | NKLTAG A | NONE | chr6 | 42333135 | 42335635 |
| 387353 | Tas2r126 | NKLTAG A | NONE | chr6 | 42362141 | 42364641 |
| 258989 | Olfr457 | NKLTAG A | NONE | chr6 | 42401283 | 42403783 |
| 58909 | D430015B01Rik | NKLTAG A | NONE | chr6 | 58953580 | 58956080 |
| 21336 | Tacr1 | NKLTAG A | NONE | chr6 | 82366132 | 82368632 |
| 75305 | Ankrd53 | NKLTAG A | NONE | chr6 | 83726301 | 83728801 |
| 12154 | Bmp10 | NKLTAG A | NONE | chr6 | 87392673 | 87395173 |
| 72103 | 2010301N04Rik\|E230015B07Rik | NKLTAG A | NONE | chr6 | 87636070 | 87639788 |
| 232217 | 4933427D06Rik | NKLTAG A | NONE | chr6 | 89059820 | 89062320 |
| 113855 | V1rb7 | NKLTAG A | NONE | chr6 | 89677843 | 89680343 |
| 113848 | V1ra6 | NKLTAG A | NONE | chr6 | 89810541 | 89813041 |
| 113856 | V1rb8 | NKLTAG A | NONE | chr6 | 89939674 | 89942174 |
| 113846 | V1ra4 | NKLTAG A | NONE | chr6 | 89985391 | 89987891 |
| 113845 | V1ra3 | NKLTAG A | NONE | chr6 | 90001845 | 90004345 |
| 113853 | V1rb3 | NKLTAG A | NONE | chr6 | 90189344 | 90191844 |
| 71699 | Slc41a3 | NKLTAG A | NONE | chr6 | 90582719 | 90585219 |
| 232227 | Iqsec1 | NKLTAG A | NONE | chr6 | 90627292 | 90629792 |
| 259302 | Srgap3 | NKLTAG A | NONE | chr6 | 112685018 | 112687518 |
| 22750 | Zfp9 | NKLTAG A | NONE | chr6 | 118444377 | 118446877 |
| 14790 | Grcc10 | NKLTAG A | NONE | chr6 | 124706198 | 124708698 |
| 54137 | Acrbp\|N/A | NKLTAG A | NONE | chr6 | 125013593 | 125016093 |
| 22158 | Tulp3 | NKLTAG A | NONE | chr6 | 128320913 | 128323413 |
| 58179 | Klrc3 | NKLTAG A | NONE | chr6 | 129608481 | 129610981 |
| 16642 | Klrc2 | NKLTAG A | NONE | chr6 | 129625882 | 129628382 |
| 16631 | Klra13\|Klra12\|Klra23 | NKLTAG A | NONE | chr6 | 130271626 | 130277194 |
| 16634 | Klra3 | NKLTAG A | NONE | chr6 | 130302723 | 130305223 |
| 93746 | Gprc5d | NKLTAG A | NONE | chr6 | 135083476 | 135085976 |
| 69187 | 1810033M07Rik | NKLTAG A | NONE | chr6 | 136885876 | 136888376 |
| 114875 | Plcz1\|Capza3 | NKLTAG A | NONE | chr6 | 140001962 | 140005823 |
| 108096 | Slco1a5 | NKLTAG A | NONE | chr6 | 142279672 | 142282172 |
| 20678 | Sox5\|N/A | NKLTAG A | NONE | chr6 | 144166139 | 144168639 |
| 71323 | Rassf8 | NKLTAG A | NONE | chr6 | 145763541 | 145766041 |
| 232836 | Galp | NKLTAG A | NONE | chr7 | 5798176 | 5800676 |
| 22776 | Zim1 | NKLTAG A | NONE | chr7 | 6300018 | 6302518 |
| 232855 | BC023179 | NKLTAG A | NONE | chr7 | 6813540 | 6816040 |
| 246791 | Obox3 | NKLTAG A | NONE | chr7 | 14575161 | 14577661 |
| 546024 | Crxos1 | NKLTAG A | NONE | chr7 | 15052645 | 15055145 |
| 14809 | Grik5 | NKLTAG A | NONE | chr7 | 24780558 | 24783058 |
| 13389 | Dll3 | NKLTAG A | NONE | chr7 | 28010044 | 28012544 |
| 57296 | Psmd8 | NKLTAG A | NONE | chr7 | 28888791 | 28891291 |
| 320225 | A230107C01Rik | NKLTAG A | NONE | chr7 | 28922243 | 28924743 |
| 74927 | 4930479M11Rik | NKLTAG A | NONE | chr7 | 30379844 | 30382344 |
| 11944 | Atp4a | NKLTAG A | NONE | chr7 | 30419111 | 30421611 |
| 233107 | Kctd15\|LOC668467 | NKLTAG A | NONE | chr7 | 34361101 | 34363601 |
| 208111 | C330019L16Rik | NKLTAG A | NONE | chr7 | 42415241 | 42417741 |
| 12489 | Cd33 | NKLTAG A | NONE | chr7 | 43400643 | 43403143 |
| 74400 | 4933405K07Rik | NKLTAG A | NONE | chr7 | 43473266 | 43475766 |
| 19144 | Klk6 | NKLTAG A | NONE | chr7 | 43691491 | 43693991 |
| 14814 | Grin2d | NKLTAG A | NONE | chr7 | 45734109 | 45736609 |
| 117589 | Asb7 | NKLTAG A | NONE | chr7 | 66557740 | 66560240 |
| 244049 | Gm489 | NKLTAG A | NONE | chr7 | 72179608 | 72182108 |
| 244058 | Rgma | NKLTAG A | NONE | chr7 | 73247019 | 73249519 |
| 402747 | D630004N19Rik | NKLTAG A | NONE | chr7 | 100421207 | 100423707 |
| 69710 | Centd2 | NKLTAG A | NONE | chr7 | 101249942 | 101252442 |
| 233578 | Olfr553 | NKLTAG A | NONE | chr7 | 102488308 | 102490808 |
| 94088 | Trim6 | NKLTAG A | NONE | chr7 | 104090636 | 104093136 |
| 209387 | AI451617 | NKLTAG A | NONE | chr7 | 104381146 | 104383646 |
| 625321 | LOC625321 | NKLTAG A | NONE | chr7 | 104408682 | 104411182 |
| 258817 | Olfr655 | NKLTAG A | NONE | chr7 | 104470500 | 104473000 |
| 259061 | Olfr668 | NKLTAG A | NONE | chr7 | 104799083 | 104801583 |
| 259063 | Olfr691 | NKLTAG A | NONE | chr7 | 105211035 | 105213535 |
| 101602 | AI467606 | NKLTAG A | NONE | chr7 | 126880583 | 126883083 |
| 381924 | Itgad | NKLTAG A | NONE | chr7 | 127963114 | 127965614 |
| 12945 | Dmbt1 | NKLTAG A | NONE | chr7 | 130821223 | 130823723 |
| 75770 | Brsk2 | NKLTAG A | NONE | chr7 | 141757140 | 141759975 |
| 14128 | Fcer2a | NKLTAG A | NONE | chr8 | 3693674 | 3696174 |
| 75528 | 1700018L24Rik | NKLTAG A | NONE | chr8 | 11838492 | 11840992 |
| 619290 | A230072I06Rik | NKLTAG A | NONE | chr8 | 12276790 | 12279290 |
| 14068 | F7 | NKLTAG A | NONE | chr8 | 13024022 | 13026522 |
| 234094 | Arhgef10 | NKLTAG A | NONE | chr8 | 14926876 | 14929376 |
| 80286 | Tusc3 | NKLTAG A | NONE | chr8 | 40654076 | 40656576 |
| 53318 | Pdlim3 | NKLTAG A | NONE | chr8 | 47382301 | 47384801 |
| 330776 | LOC330776\|Aadat | NKLTAG A | NONE | chr8 | 63394871 | 63398765 |
| 77113 | Klhl2 | NKLTAG A | NONE | chr8 | 67671557 | 67674057 |
| 434320 | LOC434320 | NKLTAG A | NONE | chr8 | 70293507 | 70296007 |
| 76813 | Armc6\|Sfrs14 | NKLTAG A | NONE | chr8 | 73161221 | 73163721 |
| 66462 | 2810428I15Rik\|Uba52 | NKLTAG A | NONE | chr8 | 73435228 | 73437728 |
| 22186 | Uba52 | NKLTAG A | NONE | chr8 | 73438856 | 73441356 |
| 244550 | 5832418A03Rik | NKLTAG A | NONE | chr8 | 87014093 | 87016593 |
| 244551 | Nanos3 | NKLTAG A | NONE | chr8 | 87066157 | 87068657 |
| 257632 | Card15 | NKLTAG A | NONE | chr8 | 91535451 | 91537951 |
| 76527 | 2010004A03Rik | NKLTAG A | NONE | chr8 | 113692049 | 113694549 |
| 56453 | Mbtps1\|LOC668258 | NKLTAG A | NONE | chr8 | 122444204 | 122446704 |
| 170732 | Trhr2 | NKLTAG A | NONE | chr8 | 125246336 | 125248836 |
| 270109 | E330039K12Rik | NKLTAG A | NONE | chr8 | 128725440 | 128727940 |
| 110350 | Dync2h1 | NKLTAG A | NONE | chr9 | 7176546 | 7179046 |
| 235040 | Atg4d | NKLTAG A | NONE | chr9 | 21013700 | 21016200 |
| 54698 | Crtam | NKLTAG A | NONE | chr9 | 40755155 | 40757655 |
| 21683 | Tecta | NKLTAG A | NONE | chr9 | 42150424 | 42152924 |
| 76509 | 1600029D21Rik | NKLTAG A | NONE | chr9 | 50244754 | 50247254 |
| 330941 | AI593442 | NKLTAG A | NONE | chr9 | 52438697 | 52441197 |
| 320563 | Islr2 | NKLTAG A | NONE | chr9 | 57999352 | 58001852 |
| 12047 | Bcl2a1d\|Bcl2a1a | NKLTAG A | NONE | chr9 | 88529243 | 88531743 |
| 235534 | Acpl2 | NKLTAG A | NONE | chr9 | 96698274 | 96700774 |
| 56492 | Cldn18 | NKLTAG A | NONE | chr9 | 99518867 | 99521367 |
| 378954 | 3000002C10Rik\|Nme6 | NKLTAG A | NONE | chr9 | 109688199 | 109690699 |
| 74498 | Gorasp1\|ttc21a | NKLTAG A | NONE | chr9 | 119784303 | 119788256 |
| 546166 | LOC546166 | NKLTAG A | NONE | chr9 | 119984527 | 119987027 |
| 434446 | LOC434446 | NKLTAG A | NONE | chr9 | 121684719 | 121687219 |
| 75199 | Rhox2 | NKLTAG A | NONE | chrX | 33674436 | 33676936 |
| 621852 | LOC621852 | NKLTAG A | NONE | chrX | 33967141 | 33969641 |
| 320707 | Atp2b3 | NKLTAG A | NONE | chrX | 69753805 | 69756305 |
| 382243 | LOC382243 | NKLTAG A | NONE | chrX | 144933316 | 144935816 |
| 12169 | Bmx | NKLTAG A | NONE | chrX | 159601798 | 159604298 |
| 66816 | Thap2\|Psrc2 | NONE | MEF A | chr10 | 114787068 | 114790545 |
| 619332 | 4933416C03Rik | NONE | MEF A | chr10 | 115517489 | 115519989 |
| 77397 | 9530003J23Rik | NONE | MEF A | chr10 | 116642216 | 116644716 |
| 75801 | Six6os1 | NONE | MEF A | chr12 | 73836103 | 73838603 |
| 16475 | Jub | NONE | MEF A | chr14 | 53531121 | 53533621 |
| 13618 | Ednrb | NONE | MEF A | chr14 | 102728875 | 102731375 |
| 70069 | H1fnt | NONE | MEF A | chr15 | 98084813 | 98087313 |
| 209776 | Gpr139 | NONE | MEF A | chr7 | 118975023 | 118977523 |
| 18387 | Oprk1 | NONE | MEF A&P | chr1 | 5576573 | 5579073 |
| 240690 | St18 | NONE | MEF A&P | chr1 | 6718131 | 6720631 |
| 71096 | Sntg1 | NONE | MEF A&P | chr1 | 9283952 | 9286452 |
| 59014 | Rrs1\|Adhfe1 | NONE | MEF A&P | chr1 | 9531293 | 9533793 |
| 29819 | Stau2 | NONE | MEF A&P | chr1 | 16360109 | 16362609 |
| 83691 | Crispld1 | NONE | MEF A&P | chr1 | 17710635 | 17713135 |
| 574082 | Defb49 | NONE | MEF A&P | chr1 | 18208278 | 18210778 |
| 77673 | Defb41\|Defb17 | NONE | MEF A&P | chr1 | 18249794 | 18252352 |
| 226896 | Tcfap2d | NONE | MEF A&P | chr1 | 19086237 | 19088737 |
| 433278 | BC099381\|Ndg1 | NONE | MEF A&P | chr1 | 21333064 | 21335564 |
| 12839 | Col9a1 | NONE | MEF A&P | chr1 | 24129974 | 24132474 |
| 210933 | Bai3 | NONE | MEF A&P | chr1 | 25773753 | 25776253 |
| 210940 | 4931408C20Rik | NKLTAG A | MEF A&P | chr1 | 26631506 | 26634006 |
| 210962 | Gm597 | NONE | MEF A&P | chr1 | 28724298 | 28726798 |
| 70988 | 4931428L18Rik\|4930521A18Rik | NONE | MEF A&P | chr1 | 31165384 | 31169157 |
| 442837 | C230029F24Rik | NONE | MEF A&P | chr1 | 49187160 | 49189660 |
| 241076 | C030018G13Rik | NONE | MEF A&P | chr1 | 66399621 | 66402121 |
| 66722 | Spag16 | NONE | MEF A&P | chr1 | 69758195 | 69760695 |
| 381287 | A530032D15Rik\|5830484A20Rik\|A530040E14Rik | NKLTAG A | MEF A&P | chr1 | 84988378 | 84991336 |
| 620078 | LOC620078\|C130026I21Rik | NKLTAG A | MEF A&P | chr1 | 85149092 | 85151592 |
| 109032 | 5830484A20Rik\|MGC118608\|LOC434484 | NKLTAG A | MEF A&P | chr1 | 87429475 | 87432373 |
| 20684 | Sp100 | NKLTAG A | MEF A&P | chr1 | 87479184 | 87481684 |
| 394436 | Ugt1a1 | NONE | MEF A&P | chr1 | 90041129 | 90043629 |
| 171382 | Trpm8 | NKLTAG A | MEF A&P | chr1 | 90132452 | 90134952 |
| 20452 | St8sia4\|N/A | NONE | MEF A&P | chr1 | 97497500 | 97500000 |
| 74441 | Slco6c1 | NONE | MEF A&P | chr1 | 98958209 | 98960709 |
| 23836 | Cdh20 | NONE | MEF A&P | chr1 | 106594365 | 106596865 |
| 71869 | Serpinb12 | NONE | MEF A&P | chr1 | 108759995 | 108762495 |
| 241196 | Serpinb13 | NONE | MEF A&P | chr1 | 108806530 | 108809030 |
| 20248 | Serpinb3a | NONE | MEF A&P | chr1 | 108879350 | 108881850 |
| 394252 | Serpinb3d | NONE | MEF A&P | chr1 | 108910527 | 108913027 |
| 383548 | Serpinb3b | NONE | MEF A&P | chr1 | 108988161 | 108990661 |
| 381286 | Serpinb3c | NONE | MEF A&P | chr1 | 109104061 | 109106561 |
| 66957 | Serpinb11 | NONE | MEF A&P | chr1 | 109187860 | 109190360 |
| 241197 | Serpinb10 | NONE | MEF A&P | chr1 | 109354549 | 109357049 |
| 17167 | Marco | NONE | MEF A&P | chr1 | 122332130 | 122334630 |
| 269109 | Dpp10\|LOC637749 | NONE | MEF A&P | chr1 | 125870368 | 125875098 |
| 210417 | D130067I03Rik | NONE | MEF A&P | chr1 | 131099019 | 131101519 |
| 13137 | Daf2 | NONE | MEF A&P | chr1 | 132250055 | 132252555 |
| 22789 | Zp3r | NONE | MEF A&P | chr1 | 132456641 | 132459141 |
| 12269 | C4bp | NONE | MEF A&P | chr1 | 132488645 | 132491145 |
| 64435 | Fcamr | NONE | MEF A&P | chr1 | 132626447 | 132628947 |
| 69169 | Faim3 | NONE | MEF A&P | chr1 | 132691322 | 132693822 |
| 19264 | Ptprc | NONE | MEF A&P | chr1 | 139991177 | 139993677 |
| 170788 | Crb1 | NKLTAG A | MEF A&P | chr1 | 141192952 | 141195452 |
| 14060 | F13b | NONE | MEF A&P | chr1 | 141316137 | 141318637 |
| 50702 | Cfhr1 | NONE | MEF A&P | chr1 | 141376133 | 141378633 |
| 214403 | LOC214403 | NONE | MEF A&P | chr1 | 141597147 | 141599647 |
| 545366 | BC026782 | NONE | MEF A&P | chr1 | 141674541 | 141677041 |
| 64214 | Rgs18 | NONE | MEF A&P | chr1 | 146537139 | 146539639 |
| 215378 | B830045N13Rik | NONE | MEF A&P | chr1 | 148255907 | 148258407 |
| 240843 | 6430517E21Rik | NONE | MEF A&P | chr1 | 160192503 | 160195003 |
| 11899 | Astn1 | NONE | MEF A&P | chr1 | 160197032 | 160199532 |
| 240894 | 4831428F09Rik | NONE | MEF A&P | chr1 | 168518020 | 168520520 |
| 545378 | Sh2d1b2 | NONE | MEF A&P | chr1 | 172067545 | 172070045 |
| 80915 | Dusp12 | NONE | MEF A&P | chr1 | 172721715 | 172724215 |
| 493583 | Itlnb\|Itlna | NONE | MEF A&P | chr1 | 173371459 | 173373968 |
| 18106 | Cd244 | NONE | MEF A&P | chr1 | 173393873 | 173396373 |
| 17085 | Ly9 | NONE | MEF A&P | chr1 | 173443449 | 173445949 |
| 75345 | Slamf7 | NONE | MEF A&P | chr1 | 173489212 | 173491712 |
| 12506 | Cd48 | NONE | MEF A&P | chr1 | 173516729 | 173519229 |
| 27218 | Slamf1 | NONE | MEF A&P | chr1 | 173601831 | 173604331 |
| 12523 | Cd84 | NONE | MEF A&P | chr1 | 173675278 | 173677778 |
| 30925 | Slamf6 | NONE | MEF A&P | chr1 | 173752211 | 173754711 |
| 258759 | Olfr1408 | NONE | MEF A&P | chr1 | 174967390 | 174969890 |
| 258758 | Olfr1406 | NONE | MEF A&P | chr1 | 175020607 | 175023107 |
| 258880 | Olfr218 | NONE | MEF A&P | chr1 | 175038032 | 175040532 |
| 258881 | Olfr1404 | NONE | MEF A&P | chr1 | 175050327 | 175052827 |
| 13349 | Darc | NONE | MEF A&P | chr1 | 175169619 | 175172119 |
| 319836 | E430029J22Rik | NONE | MEF A&P | chr1 | 175435449 | 175437949 |
| 258302 | Olfr420 | NKLTAG A | MEF A&P | chr1 | 175993449 | 175995949 |
| 258238 | Olfr417 | NONE | MEF A&P | chr1 | 176203593 | 176206093 |
| 258709 | Olfr415 | NONE | MEF A&P | chr1 | 176225745 | 176228245 |
| 258756 | Olfr414 | NONE | MEF A&P | chr1 | 176265104 | 176267604 |
| 54418 | Fmn2 | NONE | MEF A&P | chr1 | 176323846 | 176326346 |
| 545388 | B020018G12Rik | NONE | MEF A&P | chr1 | 177815289 | 177817789 |
| 110957 | D1Pas1 | NONE | MEF A&P | chr1 | 188666203 | 188668703 |
| 213783 | Plekhg1 | NONE | MEF A&P | chr10 | 6473374 | 6475874 |
| 215772 | 9130014G24Rik | NONE | MEF A&P | chr10 | 10119529 | 10122029 |
| 14816 | Grm1 | NKLTAG A | MEF A&P | chr10 | 10772046 | 10774546 |
| 215821 | D10Bwg1379e | NONE | MEF A&P | chr10 | 18432674 | 18435174 |
| 237300 | 4.9E+23 | NONE | MEF A&P | chr10 | 18475709 | 18478209 |
| 353169 | Slc2a12 | NONE | MEF A&P | chr10 | 22332546 | 22335046 |
| 67719 | 2310057J18Rik | NONE | MEF A&P | chr10 | 28675219 | 28677719 |
| 67412 | N/A\|6330407J23Rik | NONE | MEF A&P | chr10 | 28831254 | 28833754 |
| 78977 | Popdc3 | NONE | MEF A&P | chr10 | 44975719 | 44978219 |
| 14727 | Gp49a | NONE | MEF A&P | chr10 | 51167093 | 51169593 |
| 14728 | Lilrb4 | NONE | MEF A&P | chr10 | 51177389 | 51179889 |
| 210198 | Gprc6a | NONE | MEF A&P | chr10 | 51319373 | 51321873 |
| 320995 | Rfxdc1 | NONE | MEF A&P | chr10 | 51371932 | 51374432 |
| 19886 | Ros1 | NONE | MEF A&P | chr10 | 51883159 | 51885659 |
| 22639 | Zfa | NONE | MEF A&P | chr10 | 52233654 | 52236154 |
| 215085 | Slc35f1 | NONE | MEF A&P | chr10 | 52377019 | 52379519 |
| 73390 | 1700060H10Rik | NONE | MEF A&P | chr10 | 55793331 | 55795831 |
| 544696 | D630037F22Rik | NONE | MEF A&P | chr10 | 55850106 | 55852606 |
| 18768 | Pkib | NONE | MEF A&P | chr10 | 57332283 | 57334783 |
| 18768 | Pkib | NONE | MEF A&P | chr10 | 57337395 | 57339895 |
| 12140 | Fabp7 | NONE | MEF A&P | chr10 | 57471335 | 57473835 |
| 57319 | Smpdl3a | NONE | MEF A&P | chr10 | 57481014 | 57483514 |
| 245263 | LOC245263 | NONE | MEF A&P | chr10 | 57631517 | 57634017 |
| 11994 | Pcdh15 | NONE | MEF A&P | chr10 | 73665152 | 73667652 |
| 544710 | LOC544710 | NONE | MEF A&P | chr10 | 77237042 | 77239542 |
| 75188 | 1700009J07Rik | NONE | MEF A&P | chr10 | 77294556 | 77297056 |
| 404334 | Olfr1355 | NONE | MEF A&P | chr10 | 78280302 | 78282802 |
| 259044 | Olfr1353 | NONE | MEF A&P | chr10 | 78370779 | 78373279 |
| 259074 | Olfr1352 | NONE | MEF A&P | chr10 | 78384920 | 78387420 |
| 259042 | Olfr1351 | NONE | MEF A&P | chr10 | 78418452 | 78420952 |
| 18357 | Olfr57 | NKLTAG A | MEF A&P | chr10 | 78435926 | 78438426 |
| 216144 | AJ543404 | NKLTAG A | MEF A&P | chr10 | 78648903 | 78651403 |
| 408062 | BC062115 | NONE | MEF A&P | chr10 | 81486271 | 81488771 |
| 237411 | 4932415D10Rik | NONE | MEF A&P | chr10 | 81714577 | 81717077 |
| 432480 | Gm1553 | NONE | MEF A&P | chr10 | 81921917 | 81924417 |
| 18478 | Pah | NONE | MEF A&P | chr10 | 86949767 | 86952267 |
| 110312 | Pmch | NONE | MEF A&P | chr10 | 87518873 | 87521373 |
| 216225 | Slc5a8 | NONE | MEF A&P | chr10 | 88313835 | 88316335 |
| 320091 | Tmem16d | NONE | MEF A&P | chr10 | 88687089 | 88689589 |
| 71832 | Csl | NKLTAG A | MEF A&P | chr10 | 99188846 | 99191346 |
| 67569 | Mgat4c | NKLTAG A | MEF A&P | chr10 | 101802124 | 101804624 |
| 103098 | Slc6a15 | NONE | MEF A&P | chr10 | 102795530 | 102798030 |
| 353025 | Caps2 | NONE | MEF A&P | chr10 | 111565730 | 111568230 |
| 19279 | Ptprr | NKLTAG A | MEF A&P | chr10 | 115420508 | 115423008 |
| 17105 | Lyzs | NONE | MEF A&P | chr10 | 116685874 | 116688374 |
| 50929 | Il22\|Iltifb | NONE | MEF A&P | chr10 | 117605934 | 117608434 |
| 116849 | Iltifb | NONE | MEF A&P | chr10 | 117697531 | 117700031 |
| 15978 | Ifng | NONE | MEF A&P | chr10 | 117842039 | 117844539 |
| 19683 | Rdh16 | NONE | MEF A&P | chr10 | 127202102 | 127204602 |
| 380674 | AY053573 | NONE | MEF A&P | chr10 | 127225232 | 127227732 |
| 258760 | Olfr767 | NONE | MEF A&P | chr10 | 128482410 | 128484910 |
| 257667 | Olfr769 | NONE | MEF A&P | chr10 | 128514872 | 128517372 |
| 258862 | Olfr770 | NONE | MEF A&P | chr10 | 128536215 | 128538715 |
| 258540 | Olfr771 | NONE | MEF A&P | chr10 | 128563431 | 128565931 |
| 257666 | Olfr772 | NONE | MEF A&P | chr10 | 128577468 | 128579968 |
| 257664 | Olfr773-ps1 | NONE | MEF A&P | chr10 | 128589868 | 128592368 |
| 258232 | Olfr774 | NONE | MEF A&P | chr10 | 128639099 | 128641599 |
| 258539 | Olfr775\|Olfr1518 | NONE | MEF A&P | chr10 | 128651484 | 128653984 |
| 404321 | Olfr776 | NONE | MEF A&P | chr10 | 128661911 | 128664411 |
| 258537 | Olfr777 | NONE | MEF A&P | chr10 | 128671770 | 128674270 |
| 258723 | Olfr781 | NONE | MEF A&P | chr10 | 128733831 | 128736331 |
| 257985 | Olfr782 | NONE | MEF A&P | chr10 | 128751513 | 128754013 |
| 258724 | Olfr784 | NKLTAG A | MEF A&P | chr10 | 128788583 | 128791083 |
| 258542 | Olfr786 | NKLTAG A | MEF A&P | chr10 | 128837762 | 128840262 |
| 258069 | Olfr787 | NONE | MEF A&P | chr10 | 128863626 | 128866126 |
| 258544 | Olfr788 | NONE | MEF A&P | chr10 | 128873642 | 128876142 |
| 258935 | Olfr790 | NONE | MEF A&P | chr10 | 128901834 | 128904334 |
| 258932 | Olfr791 | NONE | MEF A&P | chr10 | 128927177 | 128929677 |
| 258150 | Olfr792 | NONE | MEF A&P | chr10 | 128941487 | 128943987 |
| 258375 | Olfr794 | NONE | MEF A&P | chr10 | 128971605 | 128974105 |
| 258933 | Olfr796 | NKLTAG A | MEF A&P | chr10 | 129010928 | 129013428 |
| 258549 | Olfr798 | NONE | MEF A&P | chr10 | 129028508 | 129031008 |
| 258929 | Olfr799 | NONE | MEF A&P | chr10 | 129048078 | 129050578 |
| 258541 | Olfr800 | NONE | MEF A&P | chr10 | 129060756 | 129063256 |
| 258282 | Olfr801 | NONE | MEF A&P | chr10 | 129072966 | 129075466 |
| 258934 | Olfr802 | NONE | MEF A&P | chr10 | 129085186 | 129087686 |
| 258547 | Olfr803 | NONE | MEF A&P | chr10 | 129094488 | 129096988 |
| 258068 | Olfr804 | NKLTAG A | MEF A&P | chr10 | 129105828 | 129108328 |
| 258548 | Olfr805 | NONE | MEF A&P | chr10 | 129125991 | 129128491 |
| 258546 | Olfr806 | NONE | MEF A&P | chr10 | 129141364 | 129143864 |
| 258931 | Olfr807 | NONE | MEF A&P | chr10 | 129157897 | 129160397 |
| 258930 | Olfr808 | NONE | MEF A&P | chr10 | 129168446 | 129170946 |
| 258321 | Olfr809 | NKLTAG A | MEF A&P | chr10 | 129176819 | 129179319 |
| 258543 | Olfr810 | NONE | MEF A&P | chr10 | 129194036 | 129196536 |
| 258545 | Olfr811 | NONE | MEF A&P | chr10 | 129204972 | 129207472 |
| 258791 | Olfr812 | NONE | MEF A&P | chr10 | 129245489 | 129247989 |
| 258252 | Olfr813 | NKLTAG A | MEF A&P | chr10 | 129257468 | 129259968 |
| 259165 | Olfr814 | NONE | MEF A&P | chr10 | 129277204 | 129279704 |
| 258665 | Olfr815 | NONE | MEF A&P | chr10 | 129305175 | 129307675 |
| 258667 | Olfr816 | NONE | MEF A&P | chr10 | 129314725 | 129317225 |
| 258773 | Olfr818 | NONE | MEF A&P | chr10 | 129348509 | 129351009 |
| 258670 | Olfr820 | NONE | MEF A&P | chr10 | 129418311 | 129420811 |
| 258772 | Olfr821 | NONE | MEF A&P | chr10 | 129434576 | 129437076 |
| 258666 | Olfr822 | NONE | MEF A&P | chr10 | 129475360 | 129477860 |
| 258668 | Olfr823 | NKLTAG A | MEF A&P | chr10 | 129515237 | 129517737 |
| 258669 | Olfr824 | NONE | MEF A&P | chr10 | 129529504 | 129532004 |
| 258672 | Olfr825\|LOC623635 | NKLTAG A | MEF A&P | chr10 | 129565773 | 129568273 |
| 258671 | Olfr826 | NKLTAG A | MEF A&P | chr10 | 129583327 | 129585827 |
| 258297 | Olfr827 | NKLTAG A | MEF A&P | chr10 | 129613577 | 129616077 |
| 11923 | Neurod4 | NKLTAG A | MEF A&P | chr10 | 129682675 | 129685175 |
| 67596 | 5830405N20Rik | NONE | MEF A&P | chr10 | 129748237 | 129750737 |
| 211739 | BC027127 | NONE | MEF A&P | chr11 | 16155774 | 16158274 |
| 56193 | Plek\|1500041B16Rik | NKLTAG A | MEF A&P | chr11 | 16949936 | 16952436 |
| 237694 | 4932414J04Rik | NKLTAG A | MEF A&P | chr11 | 21410683 | 21413183 |
| 216644 | D130052B06Rik | NKLTAG A | MEF A&P | chr11 | 33497301 | 33499801 |
| 16822 | Lcp2 | NONE | MEF A&P | chr11 | 33945200 | 33947700 |
| 574403 | MGC99845 | NONE | MEF A&P | chr11 | 34212821 | 34215321 |
| 94176 | Dock2 | NONE | MEF A&P | chr11 | 34414036 | 34416536 |
| 237730 | AI595406 | NONE | MEF A&P | chr11 | 35641497 | 35643997 |
| 14406 | Gabrg2 | NKLTAG A | MEF A&P | chr11 | 41843242 | 41846101 |
| 14399 | Gabra6 | NONE | MEF A&P | chr11 | 42163931 | 42166431 |
| 258463 | Olfr1393 | NKLTAG A | MEF A&P | chr11 | 49121572 | 49124072 |
| 258462 | Olfr1392 | NONE | MEF A&P | chr11 | 49134745 | 49137245 |
| 18307 | Olfr10 | NONE | MEF A&P | chr11 | 49158970 | 49161470 |
| 259068 | Olfr1390 | NKLTAG A | MEF A&P | chr11 | 49181956 | 49184456 |
| 259069 | Olfr1389 | NONE | MEF A&P | chr11 | 49271900 | 49274400 |
| 258459 | Olfr1388 | NKLTAG A | MEF A&P | chr11 | 49285275 | 49287775 |
| 258465 | Olfr1387 | NKLTAG A | MEF A&P | chr11 | 49301103 | 49303603 |
| 257888 | Olfr1386 | NKLTAG A | MEF A&P | chr11 | 49311575 | 49314075 |
| 258027 | Olfr1385 | NKLTAG A | MEF A&P | chr11 | 49335957 | 49338457 |
| 404337 | Olfr1383 | NKLTAG A | MEF A&P | chr11 | 49365147 | 49367647 |
| 237758 | Zfp454 | NONE | MEF A&P | chr11 | 50730366 | 50732866 |
| 258912 | Olfr1378 | NONE | MEF A&P | chr11 | 50810442 | 50812942 |
| 258913 | Olfr1377 | NONE | MEF A&P | chr11 | 50826125 | 50828625 |
| 18351 | Olfr51 | NONE | MEF A&P | chr11 | 50848396 | 50850896 |
| 211472 | Olfr1373 | NKLTAG A | MEF A&P | chr11 | 51988451 | 51990951 |
| 276865 | Olfr1371 | NKLTAG A | MEF A&P | chr11 | 52056910 | 52059410 |
| 69864 | 1810065E05Rik | NONE | MEF A&P | chr11 | 58235303 | 58237803 |
| 403200 | 4930504O13Rik\|Olfr30 | NONE | MEF A&P | chr11 | 58268618 | 58271118 |
| 18329 | Olfr30 | NONE | MEF A&P | chr11 | 58271642 | 58274142 |
| 257932 | Olfr332 | NONE | MEF A&P | chr11 | 58306448 | 58308948 |
| 258179 | Olfr331 | NKLTAG A | MEF A&P | chr11 | 58318249 | 58320749 |
| 258879 | Olfr330 | NKLTAG A | MEF A&P | chr11 | 58345679 | 58348179 |
| 259148 | Olfr329 | NKLTAG A | MEF A&P | chr11 | 58359151 | 58361651 |
| 258495 | Olfr328 | NKLTAG A | MEF A&P | chr11 | 58367932 | 58370432 |
| 258261 | Olfr325 | NKLTAG A | MEF A&P | chr11 | 58395058 | 58397558 |
| 257892 | Olfr324 | NONE | MEF A&P | chr11 | 58411586 | 58414086 |
| 78354 | 2210407C18Rik | NONE | MEF A&P | chr11 | 58429165 | 58431665 |
| 258373 | Olfr323 | NKLTAG A | MEF A&P | chr11 | 58441739 | 58444239 |
| 258493 | Olfr319 | NONE | MEF A&P | chr11 | 58515897 | 58518397 |
| 258494 | Olfr318 | NONE | MEF A&P | chr11 | 58536741 | 58539241 |
| 258064 | Olfr316 | NONE | MEF A&P | chr11 | 58571861 | 58574361 |
| 258531 | Olfr315 | NONE | MEF A&P | chr11 | 58592323 | 58594823 |
| 258529 | Olfr313 | NONE | MEF A&P | chr11 | 58631204 | 58633704 |
| 258065 | Olfr312 | NONE | MEF A&P | chr11 | 58645350 | 58647850 |
| 258530 | Olfr311 | NONE | MEF A&P | chr11 | 58655310 | 58657810 |
| 17882 | N/A\|Myh2 | NONE | MEF A&P | chr11 | 66985413 | 66987914 |
| 17879 | Myh1 | NONE | MEF A&P | chr11 | 67014308 | 67016808 |
| 17884 | Myh4 | NONE | MEF A&P | chr11 | 67052223 | 67054723 |
| 17885 | Myh8 | NONE | MEF A&P | chr11 | 67091318 | 67093818 |
| 19674 | Rcvrn | NONE | MEF A&P | chr11 | 67501491 | 67503991 |
| 93896 | Glp2r | NKLTAG A | MEF A&P | chr11 | 67586848 | 67589348 |
| 24070 | Mpdu1\|Cd68 | NKLTAG A | MEF A&P | chr11 | 69478337 | 69480837 |
| 12514 | Cd68 | NONE | MEF A&P | chr11 | 69481757 | 69484257 |
| 327956 | Vmo1 | NONE | MEF A&P | chr11 | 70330311 | 70332811 |
| 216871 | C730027E14Rik | NONE | MEF A&P | chr11 | 70333403 | 70335903 |
| 195046 | Nalp1 | NONE | MEF A&P | chr11 | 70958061 | 70960561 |
| 216881 | BC030477 | NONE | MEF A&P | chr11 | 71564897 | 71567397 |
| 258924 | Olfr376 | NKLTAG A | MEF A&P | chr11 | 73188945 | 73191445 |
| 258923 | Olfr1 | NONE | MEF A&P | chr11 | 73211715 | 73214215 |
| 259026 | Olfr378 | NONE | MEF A&P | chr11 | 73241676 | 73244176 |
| 259027 | Olfr380 | NONE | MEF A&P | chr11 | 73269905 | 73272405 |
| 259024 | Olfr381 | NONE | MEF A&P | chr11 | 73302517 | 73305017 |
| 258435 | Olfr382 | NONE | MEF A&P | chr11 | 73332892 | 73335392 |
| 259025 | Olfr385 | NONE | MEF A&P | chr11 | 73405431 | 73407931 |
| 193053 | Olfr386 | NONE | MEF A&P | chr11 | 73416776 | 73419276 |
| 259011 | Olfr389 | NONE | MEF A&P | chr11 | 73593020 | 73595520 |
| 258344 | Olfr390 | NKLTAG A | MEF A&P | chr11 | 73601134 | 73603634 |
| 259008 | Olfr392 | NONE | MEF A&P | chr11 | 73630775 | 73633275 |
| 258185 | Olfr255\|Olfr393 | NONE | MEF A&P | chr11 | 73663812 | 73666318 |
| 259007 | Olfr395 | NONE | MEF A&P | chr11 | 73723185 | 73725685 |
| 18321 | Olfr23 | NONE | MEF A&P | chr11 | 73754442 | 73756942 |
| 258343 | Olfr397 | NONE | MEF A&P | chr11 | 73778804 | 73781304 |
| 259005 | Olfr139 | NONE | MEF A&P | chr11 | 73860967 | 73863467 |
| 258701 | Olfr401 | NKLTAG A | MEF A&P | chr11 | 73935485 | 73937985 |
| 258703 | Olfr402 | NKLTAG A | MEF A&P | chr11 | 73969350 | 73971850 |
| 404316 | Olfr403 | NONE | MEF A&P | chr11 | 74009699 | 74012199 |
| 258706 | Olfr43 | NONE | MEF A&P | chr11 | 74022909 | 74025409 |
| 258181 | Olfr406 | NONE | MEF A&P | chr11 | 74083585 | 74086085 |
| 18359 | Olfr59 | NONE | MEF A&P | chr11 | 74102842 | 74105342 |
| 258702 | Olfr410 | NKLTAG A | MEF A&P | chr11 | 74150924 | 74153424 |
| 11418 | Accn1 | NONE | MEF A&P | chr11 | 80787309 | 80789809 |
| 11418 | Accn1 | NONE | MEF A&P | chr11 | 80968598 | 80971098 |
| 237880 | 1700071K01Rik | NONE | MEF A&P | chr11 | 81389234 | 81391734 |
| 276950 | Slfn8 | NONE | MEF A&P | chr11 | 82836505 | 82839005 |
| 20555 | Slfn1 | NONE | MEF A&P | chr11 | 82931039 | 82933539 |
| 20558 | Slfn4 | NONE | MEF A&P | chr11 | 82989380 | 82991880 |
| 20557 | Slfn3 | NONE | MEF A&P | chr11 | 83005524 | 83008024 |
| 214604 | 4932411E22Rik | NONE | MEF A&P | chr11 | 89234538 | 89237038 |
| 76758 | Gsdm2 | NONE | MEF A&P | chr11 | 98460852 | 98463352 |
| 70810 | 4631426H08Rik | NONE | MEF A&P | chr11 | 99138531 | 99141031 |
| 16675 | Krt1-c29 | NONE | MEF A&P | chr11 | 99166684 | 99169184 |
| 70831 | 4733401H21Rik | NONE | MEF A&P | chr11 | 99722009 | 99724509 |
| 71888 | 2310015J09Rik | NKLTAG A | MEF A&P | chr11 | 99831802 | 99834302 |
| 380730 | Gm884 | NONE | MEF A&P | chr11 | 103430329 | 103432829 |
| 140723 | Cacng5 | NONE | MEF A&P | chr11 | 107726952 | 107729452 |
| 76380 | Ccdc46 | NONE | MEF A&P | chr11 | 108286106 | 108288606 |
| 76380 | Ccdc46 | NONE | MEF A&P | chr11 | 108496707 | 108499207 |
| 16517 | Kcnj16 | NKLTAG A | MEF A&P | chr11 | 110833676 | 110836176 |
| 319819 | 4932435O22Rik | NKLTAG A | MEF A&P | chr11 | 114264450 | 114266950 |
| 140497 | Cd300d | NONE | MEF A&P | chr11 | 114817470 | 114819970 |
| 382551 | Clm3 | NKLTAG A | MEF A&P | chr11 | 114863885 | 114866385 |
| 217306 | Cd300e | NKLTAG A | MEF A&P | chr11 | 114877626 | 114880126 |
| 328059 | Slc7a15 | NONE | MEF A&P | chr12 | 8624574 | 8627074 |
| 245297 | LOC245297 | NONE | MEF A&P | chr12 | 18155529 | 18158029 |
| 236366 | 5730507C01Rik | NONE | MEF A&P | chr12 | 18677209 | 18679709 |
| 382686 | LOC382686 | NONE | MEF A&P | chr12 | 21167254 | 21169754 |
| 211914 | Ddef2 | NONE | MEF A&P | chr12 | 21356245 | 21358745 |
| 432637 | LOC432637 | NONE | MEF A&P | chr12 | 22452724 | 22455224 |
| 69732 | 2410018L13Rik | NONE | MEF A&P | chr12 | 23102900 | 23105400 |
| 636791 | LOC636791 | NONE | MEF A&P | chr12 | 27746401 | 27748901 |
| 17933 | Myt1l | NONE | MEF A&P | chr12 | 30112660 | 30115160 |
| 70948 | Wdr20 | NONE | MEF A&P | chr12 | 66142121 | 66144621 |
| 78257 | 4921529O18Rik | NONE | MEF A&P | chr12 | 73358770 | 73361270 |
| 193322 | Oog1 | NONE | MEF A&P | chr12 | 88493889 | 88496389 |
| 544881 | LOC544881 | NONE | MEF A&P | chr12 | 88505970 | 88508470 |
| 435337 | LOC435337 | NONE | MEF A&P | chr12 | 88787252 | 88789752 |
| 18191 | Nrxn3 | NONE | MEF A&P | chr12 | 89464877 | 89467377 |
| 625794 | LOC625794 | NONE | MEF A&P | chr12 | 93289101 | 93291601 |
| 271036 | 4932415G16Rik | NONE | MEF A&P | chr12 | 101803731 | 101806231 |
| 75483 | Cox8c | NKLTAG A | MEF A&P | chr12 | 103298355 | 103300855 |
| 20704 | Serpina1e | NKLTAG A | MEF A&P | chr12 | 104357447 | 104359947 |
| 27378 | Tcl1b3 | NONE | MEF A&P | chr12 | 105590092 | 105592592 |
| 27380 | Tcl1b4 | NONE | MEF A&P | chr12 | 105601471 | 105603971 |
| 238406 | Adam6 | NONE | MEF A&P | chr12 | 113989715 | 113992215 |
| 544903 | LOC544903\|Ighg | NONE | MEF A&P | chr12 | 114306978 | 114309478 |
| 217944 | Rapgef5 | NONE | MEF A&P | chr12 | 117956487 | 117958987 |
| 432720 | Akr1c19 | NONE | MEF A&P | chr13 | 4230985 | 4233485 |
| 320581 | 4833405L16Rik | NONE | MEF A&P | chr13 | 8950108 | 8952608 |
| 26421 | Mrpplf3\|Plf\|Plf2 | NONE | MEF A&P | chr13 | 12854640 | 12857202 |
| 107849 | Mrpplf4 | NONE | MEF A&P | chr13 | 13070436 | 13072936 |
| 218030 | Pou6f2 | NONE | MEF A&P | chr13 | 18388969 | 18391469 |
| 218038 | Amph | NONE | MEF A&P | chr13 | 18953835 | 18956335 |
| 27052 | Aoah | NONE | MEF A&P | chr13 | 20799583 | 20802083 |
| 18341 | Olfr42 | NONE | MEF A&P | chr13 | 21138241 | 21140741 |
| 258527 | Olfr1368 | NONE | MEF A&P | chr13 | 21150020 | 21152520 |
| 258280 | Olfr1366 | NONE | MEF A&P | chr13 | 21544923 | 21547423 |
| 404335 | Olfr1365 | NONE | MEF A&P | chr13 | 21562985 | 21565485 |
| 258533 | Olfr1364 | NONE | MEF A&P | chr13 | 21581419 | 21583919 |
| 218066 | Olfr11 | NONE | MEF A&P | chr13 | 21646486 | 21648986 |
| 258536 | Olfr1360 | NONE | MEF A&P | chr13 | 21681907 | 21684407 |
| 252912 | V1rh17 | NONE | MEF A&P | chr13 | 22093342 | 22095842 |
| 252906 | V1rh2 | NONE | MEF A&P | chr13 | 22109630 | 22112130 |
| 252908 | V1ri8 | NONE | MEF A&P | chr13 | 22152490 | 22154990 |
| 252907 | V1ri1 | NONE | MEF A&P | chr13 | 22195013 | 22197513 |
| 171259 | V1ri9 | NONE | MEF A&P | chr13 | 22226785 | 22229285 |
| 171257 | V1ri6 | NONE | MEF A&P | chr13 | 22283826 | 22286326 |
| 171278 | V1rh21 | NONE | MEF A&P | chr13 | 22333375 | 22335875 |
| 171254 | V1ri3 | NONE | MEF A&P | chr13 | 22359810 | 22362310 |
| 171247 | V1rh4 | NONE | MEF A&P | chr13 | 22388002 | 22390502 |
| 171246 | V1rh3 | NONE | MEF A&P | chr13 | 22400493 | 22402993 |
| 171245 | V1rh1 | NONE | MEF A&P | chr13 | 22446736 | 22449236 |
| 171255 | V1ri4 | NONE | MEF A&P | chr13 | 22480082 | 22482582 |
| 171258 | V1ri7 | NONE | MEF A&P | chr13 | 22509210 | 22511710 |
| 171270 | V1rh11 | NONE | MEF A&P | chr13 | 22529515 | 22532015 |
| 171251 | V1rh8 | NKLTAG A | MEF A&P | chr13 | 22599895 | 22602395 |
| 171250 | V1rh7 | NONE | MEF A&P | chr13 | 22628000 | 22630500 |
| 432736 | RP23-75H1.7 | NONE | MEF A&P | chr13 | 22813483 | 22815983 |
| 171269 | V1rh10 | NONE | MEF A&P | chr13 | 22835079 | 22837579 |
| 171277 | V1rh20 | NONE | MEF A&P | chr13 | 22859460 | 22861960 |
| 171275 | V1rh18 | NONE | MEF A&P | chr13 | 22891126 | 22893626 |
| 171249 | V1rh6 | NONE | MEF A&P | chr13 | 23016713 | 23019213 |
| 171253 | V1ri2 | NONE | MEF A&P | chr13 | 23081256 | 23083756 |
| 171279 | V1ri10 | NONE | MEF A&P | chr13 | 23104613 | 23107113 |
| 171273 | V1rh14 | NONE | MEF A&P | chr13 | 23121695 | 23124195 |
| 171256 | V1ri5 | NONE | MEF A&P | chr13 | 23141949 | 23144449 |
| 171272 | V1rh13 | NONE | MEF A&P | chr13 | 23168107 | 23170607 |
| 171271 | V1rh12 | NKLTAG A | MEF A&P | chr13 | 23191489 | 23193989 |
| 171274 | V1rh16 | NONE | MEF A&P | chr13 | 23240006 | 23242506 |
| 319848 | 9130214H05Rik | NONE | MEF A&P | chr13 | 23921972 | 23924472 |
| 18814 | Plfr | NONE | MEF A&P | chr13 | 27723702 | 27726202 |
| 67505 | Prlpo | NONE | MEF A&P | chr13 | 27787769 | 27790269 |
| 218194 | Phactr1 | NONE | MEF A&P | chr13 | 42718552 | 42721052 |
| 380882 | Gm906 | NONE | MEF A&P | chr13 | 50262305 | 50264805 |
| 328250 | Gm806 | NONE | MEF A&P | chr13 | 50477803 | 50480303 |
| 380845 | Gm904 | NONE | MEF A&P | chr13 | 50653724 | 50656224 |
| 20745 | Spock1 | NONE | MEF A&P | chr13 | 57917398 | 57919898 |
| 26898 | Ctsj | NONE | MEF A&P | chr13 | 61015039 | 61017539 |
| 104002 | Ctsq | NONE | MEF A&P | chr13 | 61049720 | 61052220 |
| 56835 | Ctsr | NONE | MEF A&P | chr13 | 61173311 | 61175811 |
| 64139 | Ctsm | NONE | MEF A&P | chr13 | 61550919 | 61553419 |
| 117066 | Cts3 | NONE | MEF A&P | chr13 | 61579188 | 61581688 |
| 630579 | LOC630579 | NKLTAG A | MEF A&P | chr13 | 62137511 | 62140011 |
| 71508 | 8430426H19Rik | NKLTAG A | MEF A&P | chr13 | 62475935 | 62478435 |
| 435366 | LOC435366 | NKLTAG A | MEF A&P | chr13 | 62786229 | 62788729 |
| 258816 | Olfr466 | NONE | MEF A&P | chr13 | 65165016 | 65167516 |
| 97895 | Nalp4f\|LOC235779 | NONE | MEF A&P | chr13 | 65220215 | 65222739 |
| 382770 | C330014B19Rik | NONE | MEF A&P | chr13 | 66685953 | 66688453 |
| 76803 | 2410141K09Rik | NONE | MEF A&P | chr13 | 66725198 | 66727698 |
| 212569 | 6820416H06Rik | NKLTAG A | MEF A&P | chr13 | 68310918 | 68313418 |
| 328280 | Rslcan24 | NONE | MEF A&P | chr13 | 68329832 | 68332332 |
| 210044 | Adcy2 | NONE | MEF A&P | chr13 | 69466158 | 69468658 |
| 74338 | Slc6a19 | NONE | MEF A&P | chr13 | 74166197 | 74168697 |
| 18548 | Pcsk1 | NONE | MEF A&P | chr13 | 75553988 | 75556488 |
| 66479 | 1700029F12Rik | NONE | MEF A&P | chr13 | 98134528 | 98137028 |
| 27220 | Cart | NKLTAG A | MEF A&P | chr13 | 101000392 | 101002892 |
| 17948 | Birc1b | NONE | MEF A&P | chr13 | 101301849 | 101304349 |
| 53880 | Birc1g\|Birc1f | NONE | MEF A&P | chr13 | 101416373 | 101419945 |
| 66763 | 4933425L06Rik | NONE | MEF A&P | chr13 | 106200503 | 106203003 |
| 67304 | 3110070M22Rik\|LOC633640 | NKLTAG A | MEF A&P | chr13 | 120604911 | 120609256 |
| 320333 | D830030K20Rik | NKLTAG A | MEF A&P | chr14 | 3436645 | 3439145 |
| 544988 | LOC544988\|LOC554327\|LOC545007 | NKLTAG A | MEF A&P | chr14 | 3729656 | 3732156 |
| 108978 | 4930555G01Rik | NKLTAG A | MEF A&P | chr14 | 4605709 | 4608209 |
| 75438 | 1700001E04Rik | NKLTAG A | MEF A&P | chr14 | 4951171 | 4953671 |
| 545013 | LOC545013 | NKLTAG A | MEF A&P | chr14 | 6122304 | 6124804 |
| 545015 | 2610042L04Rik | NKLTAG A | MEF A&P | chr14 | 6343213 | 6345713 |
| 545015 | 2610042L04Rik | NKLTAG A | MEF A&P | chr14 | 6358475 | 6360975 |
| 218763 | Lrrc3b\|B230110C06Rik | NONE | MEF A&P | chr14 | 14229421 | 14234140 |
| 432825 | LOC432825 | NKLTAG A | MEF A&P | chr14 | 18392783 | 18395283 |
| 83997 | Slmap | NONE | MEF A&P | chr14 | 25366424 | 25368924 |
| 239036 | 4930596D02Rik | NONE | MEF A&P | chr14 | 34640967 | 34643467 |
| 218921 | 4930474N05Rik\|N/A | NONE | MEF A&P | chr14 | 34922453 | 34924953 |
| 20390 | Sftpd | NONE | MEF A&P | chr14 | 40093376 | 40095876 |
| 73415 | 1700049E17Rik | NKLTAG A | MEF A&P | chr14 | 40479923 | 40482423 |
| 75518 | 1700024B05Rik | NKLTAG A | MEF A&P | chr14 | 40616225 | 40618725 |
| 71826 | 1700001F09Rik | NKLTAG A | MEF A&P | chr14 | 42227178 | 42229678 |
| 13587 | Ear2\|Ear12 | NONE | MEF A&P | chr14 | 42980520 | 42983253 |
| 385138 | BC061237 | NKLTAG A | MEF A&P | chr14 | 43419994 | 43422494 |
| 218993 | Gm534 | NONE | MEF A&P | chr14 | 47803320 | 47805820 |
| 67419 | 3632451O06Rik | NONE | MEF A&P | chr14 | 48704779 | 48707279 |
| 258487 | Olfr722 | NONE | MEF A&P | chr14 | 48823392 | 48825892 |
| 258314 | Olfr725 | NONE | MEF A&P | chr14 | 48956852 | 48959352 |
| 258313 | Olfr726 | NONE | MEF A&P | chr14 | 49006130 | 49008630 |
| 258316 | Olfr727 | NONE | MEF A&P | chr14 | 49046529 | 49049029 |
| 258039 | Olfr728 | NONE | MEF A&P | chr14 | 49062088 | 49064588 |
| 258275 | Olfr729 | NONE | MEF A&P | chr14 | 49070323 | 49072823 |
| 258486 | Olfr730 | NONE | MEF A&P | chr14 | 49108669 | 49111169 |
| 258360 | Olfr731 | NONE | MEF A&P | chr14 | 49160334 | 49162834 |
| 258659 | Olfr732 | NONE | MEF A&P | chr14 | 49203702 | 49206202 |
| 258657 | Olfr733 | NONE | MEF A&P | chr14 | 49220758 | 49223258 |
| 258658 | Olfr734 | NONE | MEF A&P | chr14 | 49242284 | 49244784 |
| 257909 | Olfr735 | NONE | MEF A&P | chr14 | 49267891 | 49270391 |
| 239081 | Tlr11 | NONE | MEF A&P | chr14 | 49280452 | 49282952 |
| 258662 | Olfr738 | NONE | MEF A&P | chr14 | 49333496 | 49335996 |
| 258663 | Olfr739 | NONE | MEF A&P | chr14 | 49344471 | 49346971 |
| 258661 | Olfr740 | NONE | MEF A&P | chr14 | 49373004 | 49375504 |
| 258233 | Olfr741 | NONE | MEF A&P | chr14 | 49405410 | 49407910 |
| 258422 | Olfr742 | NONE | MEF A&P | chr14 | 49435156 | 49437656 |
| 257884 | Olfr744 | NKLTAG A | MEF A&P | chr14 | 49538174 | 49540674 |
| 258296 | Olfr745 | NONE | MEF A&P | chr14 | 49562233 | 49564733 |
| 258295 | Olfr746 | NONE | MEF A&P | chr14 | 49573189 | 49575689 |
| 258264 | Olfr747 | NONE | MEF A&P | chr14 | 49603083 | 49605583 |
| 258113 | Olfr748 | NONE | MEF A&P | chr14 | 49630282 | 49632782 |
| 56858 | Olfr749 | NONE | MEF A&P | chr14 | 49658645 | 49661145 |
| 638695 | LOC638695 | NONE | MEF A&P | chr14 | 50281706 | 50284206 |
| 22302 | V2r11\|V2r10 | NONE | MEF A&P | chr14 | 50371912 | 50374413 |
| 434459 | LOC434459 | NKLTAG A | MEF A&P | chr14 | 50472740 | 50475240 |
| 545047 | MGC118309 | NONE | MEF A&P | chr14 | 50638583 | 50641083 |
| 278676 | AY358078 | NONE | MEF A&P | chr14 | 50719996 | 50722496 |
| 258424 | Olfr1512 | NONE | MEF A&P | chr14 | 51294502 | 51297002 |
| 258268 | Olfr1511 | NONE | MEF A&P | chr14 | 51312222 | 51314722 |
| 258423 | Olfr1510 | NONE | MEF A&P | chr14 | 51332321 | 51334821 |
| 57271 | Olfr1509 | NONE | MEF A&P | chr14 | 51370365 | 51372865 |
| 57270 | Olfr1508 | NONE | MEF A&P | chr14 | 51385458 | 51387958 |
| 57269 | Olfr1507 | NONE | MEF A&P | chr14 | 51412413 | 51414913 |
| 320898 | A430107P09Rik | NKLTAG A | MEF A&P | chr14 | 51840393 | 51842893 |
| 320734 | C920008G01Rik | NONE | MEF A&P | chr14 | 51940365 | 51942865 |
| 279882 | A130082M07Rik | NKLTAG A | MEF A&P | chr14 | 52250482 | 52252982 |
| 17225 | Mcpt2 | NONE | MEF A&P | chr14 | 54994193 | 54996693 |
| 17227 | Mcpt4 | NONE | MEF A&P | chr14 | 55015881 | 55018381 |
| 13035 | Ctsg | NONE | MEF A&P | chr14 | 55056145 | 55058645 |
| 14942 | Gzme | NONE | MEF A&P | chr14 | 55074202 | 55076702 |
| 14944 | Gzmg | NONE | MEF A&P | chr14 | 55113150 | 55115650 |
| 30054 | Rnf17 | NONE | MEF A&P | chr14 | 55354807 | 55357307 |
| 67932 | 1700129C05Rik | NONE | MEF A&P | chr14 | 58096503 | 58099003 |
| 380907 | LOC380907 | NONE | MEF A&P | chr14 | 58132359 | 58134859 |
| 219131 | Phf11 | NKLTAG A | MEF A&P | chr14 | 58251126 | 58253626 |
| 628416 | LOC628416 | NONE | MEF A&P | chr14 | 66268323 | 66270823 |
| 380924 | Olfm4 | NONE | MEF A&P | chr14 | 78732456 | 78734956 |
| 66732 | 4921530L21Rik | NONE | MEF A&P | chr14 | 94762399 | 94764899 |
| 105439 | 9630044O09Rik | NONE | MEF A&P | chr14 | 102533921 | 102536421 |
| 76965 | Slitrk1 | NKLTAG A | MEF A&P | chr14 | 107795590 | 107798090 |
| 239318 | Plcxd3 | NONE | MEF A&P | chr15 | 4323490 | 4325990 |
| 12274 | C6 | NONE | MEF A&P | chr15 | 4675211 | 4677711 |
| 268780 | AU040377 | NONE | MEF A&P | chr15 | 7345019 | 7347519 |
| 105887 | Ugt3a1 | NONE | MEF A&P | chr15 | 9217229 | 9219729 |
| 223337 | Ugt3a2 | NONE | MEF A&P | chr15 | 9265770 | 9268270 |
| 75568 | Capsl | NONE | MEF A&P | chr15 | 9378752 | 9381252 |
| 19116 | Prlr | NONE | MEF A&P | chr15 | 10120379 | 10122879 |
| 12563 | Cdh6 | NONE | MEF A&P | chr15 | 13036102 | 13038602 |
| 215654 | Cdh12 | NONE | MEF A&P | chr15 | 21054176 | 21056676 |
| 223413 | 9230109A22Rik | NONE | MEF A&P | chr15 | 25093384 | 25095884 |
| 67498 | Kcnv1 | NONE | MEF A&P | chr15 | 44944401 | 44946901 |
| 239435 | Aard | NONE | MEF A&P | chr15 | 51868180 | 51870680 |
| 239436 | Slc30a8 | NONE | MEF A&P | chr15 | 52123635 | 52126135 |
| 320679 | Samd12 | NONE | MEF A&P | chr15 | 53732019 | 53734519 |
| 210463 | BC026439 | NONE | MEF A&P | chr15 | 57307208 | 57309708 |
| 331063 | AI987692 | NKLTAG A | MEF A&P | chr15 | 63674342 | 63676842 |
| 270328 | 9930109F21Rik | NONE | MEF A&P | chr15 | 63707724 | 63710224 |
| 213068 | Tmem71 | NONE | MEF A&P | chr15 | 66390215 | 66392715 |
| 76486 | Ly6k | NONE | MEF A&P | chr15 | 74626721 | 74629221 |
| 380997 | 9030605E09Rik | NONE | MEF A&P | chr15 | 82380863 | 82383363 |
| 223706 | BC018285 | NONE | MEF A&P | chr15 | 82448173 | 82450673 |
| 68444 | Cyp2d13 | NONE | MEF A&P | chr15 | 82469291 | 82471791 |
| 54526 | Syt10 | NONE | MEF A&P | chr15 | 89669036 | 89671536 |
| 239611 | Muc19\|Smgc | NONE | MEF A&P | chr15 | 91664092 | 91666594 |
| 239611 | Muc19 | NONE | MEF A&P | chr15 | 91732170 | 91734670 |
| 12805 | Cntn1 | NONE | MEF A&P | chr15 | 91954371 | 91956871 |
| 223838 | Adamts20 | NONE | MEF A&P | chr15 | 94231938 | 94234438 |
| 54003 | Nell2 | NONE | MEF A&P | chr15 | 95355932 | 95358432 |
| 257943 | Olfr285\|Olfr257 | NONE | MEF A&P | chr15 | 98141082 | 98143582 |
| 258278 | Olfr284 | NKLTAG A | MEF A&P | chr15 | 98168473 | 98170973 |
| 259038 | Olfr283 | NONE | MEF A&P | chr15 | 98206642 | 98209142 |
| 106051 | AW493845 | NONE | MEF A&P | chr15 | 100646058 | 100648558 |
| 109052 | Krtcap1 | NKLTAG A | MEF A&P | chr15 | 101401438 | 101403938 |
| 406223 | Kb14 | NONE | MEF A&P | chr15 | 101455722 | 101458222 |
| 16688 | Krt2-6b | NONE | MEF A&P | chr15 | 101507757 | 101510257 |
| 16687 | Krt2-6a | NONE | MEF A&P | chr15 | 101521839 | 101524339 |
| 223915 | BC067067 | NONE | MEF A&P | chr15 | 101629866 | 101632366 |
| 406220 | 4732484G22Rik | NONE | MEF A&P | chr15 | 101697152 | 101699652 |
| 14663 | Glycam1 | NONE | MEF A&P | chr15 | 103392612 | 103395112 |
| 74763 | 1200013P24Rik | NONE | MEF A&P | chr16 | 3797910 | 3800410 |
| 268859 | A2bp1 | NONE | MEF A&P | chr16 | 6983155 | 6985655 |
| 68172 | 4930517K11Rik | NKLTAG A | MEF A&P | chr16 | 10081805 | 10084305 |
| 224014 | Fgd4 | NKLTAG A | MEF A&P | chr16 | 16417279 | 16419779 |
| 74066 | 4933404G15Rik | NONE | MEF A&P | chr16 | 16631159 | 16633659 |
| 258443 | Olfr164 | NONE | MEF A&P | chr16 | 19199804 | 19202304 |
| 258458 | Olfr165 | NONE | MEF A&P | chr16 | 19321080 | 19323580 |
| 259071 | Olfr166 | NONE | MEF A&P | chr16 | 19398402 | 19400902 |
| 258937 | Olfr167 | NONE | MEF A&P | chr16 | 19428697 | 19431197 |
| 258354 | Olfr168 | NONE | MEF A&P | chr16 | 19443981 | 19446481 |
| 258959 | Olfr170 | NONE | MEF A&P | chr16 | 19519729 | 19522229 |
| 258960 | Olfr171 | NONE | MEF A&P | chr16 | 19538164 | 19540664 |
| 239789 | Gm606 | NONE | MEF A&P | chr16 | 26904707 | 26907207 |
| 14167 | Fgf12 | NONE | MEF A&P | chr16 | 28672449 | 28674949 |
| 268878 | Atp13a5 | NONE | MEF A&P | chr16 | 29297979 | 29300479 |
| 245308 | Zdhhc19 | NKLTAG A | MEF A&P | chr16 | 32414027 | 32416527 |
| 16646 | Kpna1 | NONE | MEF A&P | chr16 | 35901109 | 35903609 |
| 209294 | Csta | NONE | MEF A&P | chr16 | 36049990 | 36052490 |
| 268885 | Stfa2l1 | NONE | MEF A&P | chr16 | 36074009 | 36076509 |
| 76770 | 2010005H15Rik | NONE | MEF A&P | chr16 | 36138706 | 36141206 |
| 20861 | Stfa1 | NKLTAG A | MEF A&P | chr16 | 36253071 | 36255571 |
| 20863 | Stfa3 | NONE | MEF A&P | chr16 | 36374131 | 36376631 |
| 12374 | Casr | NONE | MEF A&P | chr16 | 36480873 | 36483373 |
| 12524 | Cd86 | NONE | MEF A&P | chr16 | 36584790 | 36587290 |
| 106347 | Ildr1 | NONE | MEF A&P | chr16 | 36611276 | 36613776 |
| 57738 | Slc15a2 | NONE | MEF A&P | chr16 | 36703701 | 36706201 |
| 207227 | Stxbp5l | NONE | MEF A&P | chr16 | 37303662 | 37306162 |
| 320874 | D930030D11Rik | NONE | MEF A&P | chr16 | 41124303 | 41126803 |
| 268890 | Lsamp | NONE | MEF A&P | chr16 | 41450673 | 41453173 |
| 208169 | Slc9a10 | NONE | MEF A&P | chr16 | 45452640 | 45455140 |
| 14525 | Gcet2 | NKLTAG A | MEF A&P | chr16 | 45527754 | 45530254 |
| 17450 | Morc1 | NONE | MEF A&P | chr16 | 48348862 | 48351362 |
| 52575 | Rg9mtd1 | NONE | MEF A&P | chr16 | 55962227 | 55964727 |
| 69457 | 2310005G13Rik | NONE | MEF A&P | chr16 | 56995797 | 56998297 |
| 66497 | 2610528E23Rik | NONE | MEF A&P | chr16 | 57527489 | 57529989 |
| 259002 | Olfr173 | NONE | MEF A&P | chr16 | 58739570 | 58742070 |
| 258999 | Olfr178 | NONE | MEF A&P | chr16 | 58831974 | 58834474 |
| 258178 | Olfr180 | NONE | MEF A&P | chr16 | 58858395 | 58860895 |
| 259001 | Olfr181 | NONE | MEF A&P | chr16 | 58868325 | 58870825 |
| 258478 | Olfr183 | NONE | MEF A&P | chr16 | 58939942 | 58942442 |
| 258318 | Olfr186 | NONE | MEF A&P | chr16 | 58969661 | 58972161 |
| 258319 | Olfr187 | NONE | MEF A&P | chr16 | 58978491 | 58980991 |
| 258392 | Olfr190 | NKLTAG A | MEF A&P | chr16 | 59016834 | 59019334 |
| 258035 | Olfr191 | NONE | MEF A&P | chr16 | 59028237 | 59030737 |
| 404309 | Olfr192 | NKLTAG A | MEF A&P | chr16 | 59040746 | 59043246 |
| 257972 | Olfr193 | NONE | MEF A&P | chr16 | 59052364 | 59054864 |
| 433031 | Olfr194 | NONE | MEF A&P | chr16 | 59061824 | 59064324 |
| 258775 | Olfr196 | NKLTAG A | MEF A&P | chr16 | 59109897 | 59112397 |
| 258477 | Olfr197 | NONE | MEF A&P | chr16 | 59128237 | 59130737 |
| 258036 | Olfr198 | NONE | MEF A&P | chr16 | 59144180 | 59146680 |
| 404310 | Olfr199 | NONE | MEF A&P | chr16 | 59158367 | 59160867 |
| 258996 | Olfr201 | NONE | MEF A&P | chr16 | 59211421 | 59213921 |
| 258997 | Olfr202 | NONE | MEF A&P | chr16 | 59226251 | 59228751 |
| 258479 | Olfr203 | NONE | MEF A&P | chr16 | 59243410 | 59245910 |
| 258994 | Olfr204 | NONE | MEF A&P | chr16 | 59257161 | 59259661 |
| 257881 | Olfr205 | NONE | MEF A&P | chr16 | 59271263 | 59273763 |
| 258993 | Olfr206 | NONE | MEF A&P | chr16 | 59287455 | 59289955 |
| 257973 | Olfr207\|Olfr208 | NONE | MEF A&P | chr16 | 59303972 | 59306472 |
| 224318 | Speer2 | NONE | MEF A&P | chr16 | 69743821 | 69746321 |
| 19146 | Prss7 | NONE | MEF A&P | chr16 | 78973184 | 78975689 |
| 268905 | Krtap13-1 | NONE | MEF A&P | chr16 | 88615720 | 88618220 |
| 16699 | Krtap13 | NKLTAG A | MEF A&P | chr16 | 88639987 | 88642487 |
| 23927 | Krtap14\|Krtap15 | NKLTAG A | MEF A&P | chr16 | 88714491 | 88718367 |
| 71369 | Krtap16-10 | NONE | MEF A&P | chr16 | 88851232 | 88853732 |
| 16700 | Krtap6-1 | NKLTAG A | MEF A&P | chr16 | 88918557 | 88921057 |
| 170653 | Krtap16-3\|Krtap16-8 | NKLTAG A | MEF A&P | chr16 | 88936128 | 88938740 |
| 73716 | Krtap21-1\|Krtap16-7 | NKLTAG A | MEF A&P | chr16 | 89292127 | 89294663 |
| 16701 | Krtap6-2 | NKLTAG A | MEF A&P | chr16 | 89308470 | 89310970 |
| 16522 | Kcnj6 | NONE | MEF A&P | chr16 | 95105994 | 95108494 |
| 21647 | Tcte3\|9030025P20Rik\|N/A | NONE | MEF A&P | chr17 | 14713221 | 14715721 |
| 21647 | Tcte3\|9030025P20Rik\|N/A | NONE | MEF A&P | chr17 | 14744574 | 14748522 |
| 14294 | Fprl1 | NONE | MEF A&P | chr17 | 17673075 | 17675575 |
| 14291 | Fpr-rs4 | NONE | MEF A&P | chr17 | 17724350 | 17726850 |
| 321021 | Fpr-rs7 | NONE | MEF A&P | chr17 | 19818423 | 19820923 |
| 321020 | Fpr-rs6 | NONE | MEF A&P | chr17 | 19887294 | 19889794 |
| 171228 | V1re5 | NONE | MEF A&P | chr17 | 20204995 | 20207495 |
| 14290 | Fpr-rs3 | NONE | MEF A&P | chr17 | 20329074 | 20331574 |
| 171225 | V1re2 | NONE | MEF A&P | chr17 | 20390204 | 20392704 |
| 171229 | V1re6 | NONE | MEF A&P | chr17 | 20437796 | 20440296 |
| 171226 | V1re3 | NONE | MEF A&P | chr17 | 20481698 | 20484198 |
| 171224 | V1re1 | NONE | MEF A&P | chr17 | 20517191 | 20519691 |
| 171231 | V1re8 | NONE | MEF A&P | chr17 | 20549247 | 20551747 |
| 171230 | V1re7 | NONE | MEF A&P | chr17 | 20594848 | 20597348 |
| 171232 | V1rf1 | NONE | MEF A&P | chr17 | 20931522 | 20934022 |
| 171234 | V1rf3 | NONE | MEF A&P | chr17 | 21016713 | 21019213 |
| 210853 | 6.7E+21 | NKLTAG A | MEF A&P | chr17 | 21870177 | 21872677 |
| 11307 | Abcg1 | NONE | MEF A&P | chr17 | 30775798 | 30778298 |
| 328795 | Ubash3a | NKLTAG A | MEF A&P | chr17 | 30937124 | 30939624 |
| 240069 | Morc2b | NONE | MEF A&P | chr17 | 32876175 | 32878675 |
| 69542 | 2300002M23Rik | NONE | MEF A&P | chr17 | 35173537 | 35176037 |
| 14985 | H2-M10.1 | NONE | MEF A&P | chr17 | 35933703 | 35936203 |
| 24108 | Ubd | NKLTAG A | MEF A&P | chr17 | 36799614 | 36802114 |
| 258831 | Olfr101 | NONE | MEF A&P | chr17 | 36907973 | 36910473 |
| 258218 | Olfr102\|Olfr100 | NONE | MEF A&P | chr17 | 36921935 | 36924435 |
| 258830 | Olfr103 | NONE | MEF A&P | chr17 | 36944783 | 36947283 |
| 257893 | Olfr105 | NONE | MEF A&P | chr17 | 36988559 | 36991059 |
| 257925 | Olfr106 | NONE | MEF A&P | chr17 | 37000582 | 37003082 |
| 258504 | Olfr107 | NONE | MEF A&P | chr17 | 37011602 | 37014102 |
| 258832 | Olfr109 | NONE | MEF A&P | chr17 | 37072260 | 37074760 |
| 14990 | H2-M2 | NONE | MEF A&P | chr17 | 37091082 | 37093582 |
| 258325 | Olfr110 | NONE | MEF A&P | chr17 | 37104705 | 37107205 |
| 545205 | Olfr111 | NONE | MEF A&P | chr17 | 37136031 | 37138531 |
| 258096 | Olfr112 | NKLTAG A | MEF A&P | chr17 | 37177002 | 37179502 |
| 258284 | Olfr114 | NONE | MEF A&P | chr17 | 37197904 | 37200404 |
| 257908 | Olfr115 | NONE | MEF A&P | chr17 | 37218308 | 37220808 |
| 258625 | Olfr116 | NONE | MEF A&P | chr17 | 37232186 | 37234686 |
| 258263 | Olfr117 | NONE | MEF A&P | chr17 | 37267884 | 37270384 |
| 404308 | Olfr118 | NONE | MEF A&P | chr17 | 37278077 | 37280577 |
| 258095 | Olfr119 | NONE | MEF A&P | chr17 | 37306724 | 37309224 |
| 258624 | Olfr120 | NONE | MEF A&P | chr17 | 37332051 | 37334551 |
| 258622 | Olfr121 | NONE | MEF A&P | chr17 | 37354918 | 37357418 |
| 258285 | Olfr122 | NONE | MEF A&P | chr17 | 37377680 | 37380180 |
| 258623 | Olfr123 | NONE | MEF A&P | chr17 | 37401498 | 37403998 |
| 259064 | Olfr124 | NONE | MEF A&P | chr17 | 37411199 | 37413699 |
| 258287 | Olfr125 | NONE | MEF A&P | chr17 | 37441053 | 37443553 |
| 258892 | Olfr126 | NONE | MEF A&P | chr17 | 37456646 | 37459146 |
| 258374 | Olfr127 | NONE | MEF A&P | chr17 | 37509600 | 37512100 |
| 383243 | Olfr128 | NONE | MEF A&P | chr17 | 37529620 | 37532120 |
| 258094 | Olfr761 | NONE | MEF A&P | chr17 | 37560575 | 37563075 |
| 258324 | Olfr129 | NONE | MEF A&P | chr17 | 37663144 | 37665644 |
| 258480 | Olfr130 | NONE | MEF A&P | chr17 | 37673225 | 37675725 |
| 258867 | Olfr131 | NONE | MEF A&P | chr17 | 37690529 | 37693029 |
| 257889 | Olfr132 | NONE | MEF A&P | chr17 | 37738743 | 37741243 |
| 258828 | Olfr133 | NONE | MEF A&P | chr17 | 37754642 | 37757142 |
| 258829 | Olfr134 | NKLTAG A | MEF A&P | chr17 | 37781138 | 37783638 |
| 258329 | Olfr135 | NONE | MEF A&P | chr17 | 37814299 | 37816799 |
| 170648 | Olfr138 | NONE | MEF A&P | chr17 | 37880825 | 37883325 |
| 258481 | Olfr137 | NKLTAG A | MEF A&P | chr17 | 37913012 | 37915512 |
| 258803 | Olfr136 | NONE | MEF A&P | chr17 | 37941211 | 37943711 |
| 619517 | Esp1 | NONE | MEF A&P | chr17 | 40173812 | 40176312 |
| 435529 | Gpr111 | NONE | MEF A&P | chr17 | 42205163 | 42207663 |
| 70530 | Lrfn2 | NONE | MEF A&P | chr17 | 48395945 | 48398445 |
| 634802 | LOC634802 | NONE | MEF A&P | chr17 | 48913044 | 48915544 |
| 56738 | Mocs1 | NONE | MEF A&P | chr17 | 48915807 | 48918307 |
| 328839 | A130033B22 | NONE | MEF A&P | chr17 | 51926194 | 51928694 |
| 211496 | 4932415M13Rik | NONE | MEF A&P | chr17 | 53179437 | 53181937 |
| 69083 | Sult1c2 | NONE | MEF A&P | chr17 | 53310387 | 53312887 |
| 20888 | Sult1c1 | NONE | MEF A&P | chr17 | 53455060 | 53457560 |
| 240119 | St6gal2 | NONE | MEF A&P | chr17 | 54919735 | 54922235 |
| 52614 | Emr4 | NONE | MEF A&P | chr17 | 55341121 | 55343621 |
| 13733 | Emr1 | NONE | MEF A&P | chr17 | 57042025 | 57044525 |
| 14158 | Fert2 | NONE | MEF A&P | chr17 | 63579483 | 63581983 |
| 381113 | Cdkl4\|F420015M19Rik | NONE | MEF A&P | chr17 | 80471660 | 80474160 |
| 71828 | Gtf2a1lf | NKLTAG A | MEF A&P | chr17 | 88575030 | 88577530 |
| 16867 | Lhcgr | NONE | MEF A&P | chr17 | 88699802 | 88702302 |
| 14309 | Fshr | NONE | MEF A&P | chr17 | 89108501 | 89111001 |
| 545238 | LOC545238 | NKLTAG A | MEF A&P | chr18 | 3469627 | 3472127 |
| 225192 | Hrh4 | NKLTAG A | MEF A&P | chr18 | 13148158 | 13150658 |
| 11829 | Aqp4 | NONE | MEF A&P | chr18 | 15546171 | 15548671 |
| 13505 | Dsc1 | NONE | MEF A&P | chr18 | 20257190 | 20259690 |
| 225256 | Dsg1b | NONE | MEF A&P | chr18 | 20517844 | 20520344 |
| 16769 | Dsg4 | NONE | MEF A&P | chr18 | 20577184 | 20579684 |
| 13512 | Dsg3 | NONE | MEF A&P | chr18 | 20651283 | 20653783 |
| 17288 | Mep1b | NONE | MEF A&P | chr18 | 21213353 | 21215853 |
| 70950 | 4921528I01Rik | NONE | MEF A&P | chr18 | 22313906 | 22316406 |
| 116731 | Pcdha1 | NONE | MEF A&P | chr18 | 37054258 | 37056758 |
| 353234 | Pcdha2 | NONE | MEF A&P | chr18 | 37063291 | 37065791 |
| 192163 | Pcdha3 | NONE | MEF A&P | chr18 | 37070180 | 37072680 |
| 12936 | Pcdha4 | NONE | MEF A&P | chr18 | 37076662 | 37079162 |
| 12941 | Pcdha5 | NONE | MEF A&P | chr18 | 37084413 | 37086913 |
| 12939 | Pcdha7 | NONE | MEF A&P | chr18 | 37097897 | 37100397 |
| 353235 | Pcdha8 | NONE | MEF A&P | chr18 | 37116440 | 37118940 |
| 192161 | Pcdha9 | NONE | MEF A&P | chr18 | 37121853 | 37124353 |
| 12943 | Pcdha10 | NONE | MEF A&P | chr18 | 37129293 | 37131793 |
| 12942 | Pcdha11\|Pcdha12 | NONE | MEF A&P | chr18 | 37144203 | 37146703 |
| 93872 | Pcdhb1 | NONE | MEF A&P | chr18 | 37388971 | 37391471 |
| 93873 | Pcdhb2 | NONE | MEF A&P | chr18 | 37418949 | 37421449 |
| 93874 | Pcdhb3 | NONE | MEF A&P | chr18 | 37424956 | 37427456 |
| 93875 | Pcdhb4 | NONE | MEF A&P | chr18 | 37431437 | 37433937 |
| 93876 | Pcdhb5 | NONE | MEF A&P | chr18 | 37444364 | 37446864 |
| 93877 | Pcdhb6 | NONE | MEF A&P | chr18 | 37458001 | 37460501 |
| 93878 | Pcdhb7 | NONE | MEF A&P | chr18 | 37465675 | 37468175 |
| 93879 | Pcdhb8 | NONE | MEF A&P | chr18 | 37479244 | 37481744 |
| 93880 | Pcdhb9 | NONE | MEF A&P | chr18 | 37524828 | 37527328 |
| 93881 | Pcdhb10 | NONE | MEF A&P | chr18 | 37535846 | 37538346 |
| 93883 | Pcdhb12 | NONE | MEF A&P | chr18 | 37559776 | 37562276 |
| 93884 | Pcdhb13 | NKLTAG A | MEF A&P | chr18 | 37566544 | 37569044 |
| 93711 | Pcdhga3 | NONE | MEF A&P | chr18 | 37798308 | 37800808 |
| 93700 | Pcdhgb2 | NONE | MEF A&P | chr18 | 37813832 | 37816332 |
| 93714 | Pcdhga6 | NONE | MEF A&P | chr18 | 37831202 | 37833702 |
| 20730 | Spink3 | NONE | MEF A&P | chr18 | 43862028 | 43864528 |
| 117158 | Scgb3a2 | NONE | MEF A&P | chr18 | 43887657 | 43890157 |
| 225443 | Gm94 | NONE | MEF A&P | chr18 | 43917708 | 43920208 |
| 433180 | LOC433180 | NONE | MEF A&P | chr18 | 44194723 | 44197223 |
| 78242 | 9230117E20Rik | NONE | MEF A&P | chr18 | 44227953 | 44230453 |
| 77424 | 9530002K18Rik | NONE | MEF A&P | chr18 | 44299904 | 44302404 |
| 18169 | Npy6r | NONE | MEF A&P | chr18 | 44393457 | 44395957 |
| 58916 | N/A\|Myot | NONE | MEF A&P | chr18 | 44457404 | 44459904 |
| 225583 | A730017C20Rik | NKLTAG A | MEF A&P | chr18 | 59185877 | 59188377 |
| 225594 | LOC225594 | NKLTAG A | MEF A&P | chr18 | 60398141 | 60400641 |
| 207269 | BC023105 | NONE | MEF A&P | chr18 | 60562663 | 60565163 |
| 20451 | St8sia3 | NONE | MEF A&P | chr18 | 64388756 | 64391256 |
| 17202 | Mc4r | NONE | MEF A&P | chr18 | 66985341 | 66987841 |
| 13176 | Dcc | NONE | MEF A&P | chr18 | 72475447 | 72477947 |
| 225724 | Mapk4 | NONE | MEF A&P | chr18 | 74189798 | 74192298 |
| 14427 | Galr1 | NKLTAG A | MEF A&P | chr18 | 82540729 | 82543229 |
| 225825 | Cd226 | NONE | MEF A&P | chr18 | 89329619 | 89332119 |
| 319609 | 9330132A10Rik | NONE | MEF A&P | chr18 | 89430922 | 89433422 |
| 207151 | Slc22a19 | NONE | MEF A&P | chr19 | 7777798 | 7780298 |
| 236149 | BC014805 | NONE | MEF A&P | chr19 | 7869212 | 7871712 |
| 434674 | LOC434674 | NKLTAG A | MEF A&P | chr19 | 8198527 | 8201027 |
| 236293 | D630002G06Rik | NONE | MEF A&P | chr19 | 8285384 | 8287884 |
| 319800 | C730048C13Rik | NKLTAG A | MEF A&P | chr19 | 8471650 | 8474150 |
| 18399 | Slc22a6 | NONE | MEF A&P | chr19 | 8683085 | 8685585 |
| 545279 | E530011F12Rik | NONE | MEF A&P | chr19 | 11059795 | 11062295 |
| 64381 | Ms4a8a | NONE | MEF A&P | chr19 | 11147573 | 11150073 |
| 69416 | 1700025F22Rik | NKLTAG A | MEF A&P | chr19 | 11231865 | 11234365 |
| 269063 | Ms4a5 | NONE | MEF A&P | chr19 | 11350418 | 11352918 |
| 109225 | Ms4a7 | NONE | MEF A&P | chr19 | 11402643 | 11405161 |
| 60361 | N/A\|Ms4a4b | NONE | MEF A&P | chr19 | 11508645 | 11511145 |
| 66607 | Ms4a4d | NONE | MEF A&P | chr19 | 11601893 | 11604393 |
| 225922 | Tmem122 | NONE | MEF A&P | chr19 | 11727104 | 11729604 |
| 225923 | Gm97 | NKLTAG A | MEF A&P | chr19 | 11762099 | 11764599 |
| 258227 | Olfr1418 | NKLTAG A | MEF A&P | chr19 | 11922496 | 11924996 |
| 258676 | Olfr1424 | NONE | MEF A&P | chr19 | 12126295 | 12128795 |
| 258409 | Olfr1431 | NONE | MEF A&P | chr19 | 12274612 | 12277112 |
| 258683 | Olfr262 | NKLTAG A | MEF A&P | chr19 | 12308204 | 12310704 |
| 258681 | Olfr232 | NONE | MEF A&P | chr19 | 12333276 | 12335776 |
| 258682 | Olfr1436 | NONE | MEF A&P | chr19 | 12365675 | 12368175 |
| 258117 | Olfr1437 | NONE | MEF A&P | chr19 | 12389370 | 12391870 |
| 107146 | Glyat | NONE | MEF A&P | chr19 | 12698352 | 12700852 |
| 258697 | Olfr1444 | NONE | MEF A&P | chr19 | 12926795 | 12929295 |
| 258698 | Olfr1447 | NONE | MEF A&P | chr19 | 12968297 | 12970797 |
| 258696 | Olfr1448 | NONE | MEF A&P | chr19 | 12986826 | 12989326 |
| 258300 | Olfr1449 | NONE | MEF A&P | chr19 | 12999758 | 13002258 |
| 258700 | Olfr1451 | NONE | MEF A&P | chr19 | 13064006 | 13066506 |
| 258695 | Olfr1453 | NONE | MEF A&P | chr19 | 13094846 | 13097346 |
| 258687 | Olfr1454 | NONE | MEF A&P | chr19 | 13128431 | 13130931 |
| 258568 | Olfr1457 | NONE | MEF A&P | chr19 | 13162165 | 13164665 |
| 258684 | Olfr1459 | NONE | MEF A&P | chr19 | 13213176 | 13215676 |
| 258688 | Olfr1462 | NONE | MEF A&P | chr19 | 13255687 | 13258187 |
| 258120 | Olfr1463 | NONE | MEF A&P | chr19 | 13299270 | 13301770 |
| 258121 | Olfr1465 | NONE | MEF A&P | chr19 | 13380802 | 13383302 |
| 258689 | Olfr1466 | NONE | MEF A&P | chr19 | 13406778 | 13409278 |
| 258690 | Olfr1469 | NONE | MEF A&P | chr19 | 13475589 | 13478089 |
| 258231 | Olfr1471 | NKLTAG A | MEF A&P | chr19 | 13510032 | 13512532 |
| 258685 | Olfr1472 | NONE | MEF A&P | chr19 | 13521034 | 13523534 |
| 258123 | Olfr1474 | NKLTAG A | MEF A&P | chr19 | 13535990 | 13538490 |
| 258298 | Olfr1475 | NKLTAG A | MEF A&P | chr19 | 13546715 | 13549215 |
| 258691 | Olfr1477 | NONE | MEF A&P | chr19 | 13567363 | 13569863 |
| 404339 | Olfr1480 | NONE | MEF A&P | chr19 | 13594693 | 13597193 |
| 258288 | Olfr1484 | NKLTAG A | MEF A&P | chr19 | 13650324 | 13652824 |
| 258629 | Olfr1487 | NONE | MEF A&P | chr19 | 13684182 | 13686682 |
| 258628 | Olfr1489 | NONE | MEF A&P | chr19 | 13698131 | 13700631 |
| 258098 | Olfr1490 | NONE | MEF A&P | chr19 | 13719464 | 13721964 |
| 258342 | Olfr1491 | NONE | MEF A&P | chr19 | 13769847 | 13772347 |
| 258341 | Olfr1495 | NONE | MEF A&P | chr19 | 13833362 | 13835862 |
| 258991 | Olfr1496 | NONE | MEF A&P | chr19 | 13845632 | 13848132 |
| 258736 | Olfr1497 | NONE | MEF A&P | chr19 | 13862128 | 13864628 |
| 258792 | Olfr1499 | NONE | MEF A&P | chr19 | 13882107 | 13884607 |
| 258097 | Olfr1500 | NKLTAG A | MEF A&P | chr19 | 13894913 | 13897413 |
| 258626 | Olfr1501 | NONE | MEF A&P | chr19 | 13905690 | 13908190 |
| 258793 | Olfr1502\|N/A | NONE | MEF A&P | chr19 | 13926813 | 13929313 |
| 258627 | Olfr1504 | NKLTAG A | MEF A&P | chr19 | 13954727 | 13957227 |
| 258151 | Olfr1505 | NONE | MEF A&P | chr19 | 13984040 | 13986540 |
| 225998 | Rorb | NONE | MEF A&P | chr19 | 19067640 | 19070140 |
| 18719 | Pip5k1a | NONE | MEF A&P | chr19 | 24510282 | 24512782 |
| 381236 | AI747699 | NKLTAG A | MEF A&P | chr19 | 33625135 | 33627635 |
| 67717 | Lipf | NONE | MEF A&P | chr19 | 34025244 | 34027744 |
| 329056 | A830019P07Rik | NONE | MEF A&P | chr19 | 35989222 | 35991722 |
| 72303 | Cyp2c65 | NONE | MEF A&P | chr19 | 39124058 | 39126558 |
| 69888 | Cyp2c66 | NONE | MEF A&P | chr19 | 39176941 | 39179441 |
| 13095 | Cyp2c29 | NONE | MEF A&P | chr19 | 39338414 | 39340914 |
| 13097 | Cyp2c38 | NONE | MEF A&P | chr19 | 39515886 | 39518386 |
| 13098 | Cyp2c39 | NONE | MEF A&P | chr19 | 39562181 | 39564681 |
| 545288 | C730004C24Rik | NONE | MEF A&P | chr19 | 39701853 | 39704353 |
| 433247 | 9030012A22Rik | NKLTAG A | MEF A&P | chr19 | 39793865 | 39796365 |
| 13099 | Cyp2c40 | NONE | MEF A&P | chr19 | 39865539 | 39868039 |
| 13096 | Cyp2c37 | NONE | MEF A&P | chr19 | 40043750 | 40046250 |
| 404195 | Cyp2c54 | NONE | MEF A&P | chr19 | 40126622 | 40129122 |
| 107141 | Cyp2c50 | NONE | MEF A&P | chr19 | 40141006 | 40143506 |
| 226105 | Cyp2c70 | NONE | MEF A&P | chr19 | 40240082 | 40242582 |
| 17060 | Blnk | NKLTAG A | MEF A&P | chr19 | 41047346 | 41049846 |
| 16333 | Ins1 | NKLTAG A | MEF A&P | chr19 | 52315761 | 52318261 |
| 71653 | 4930506M07Rik | NONE | MEF A&P | chr19 | 59128819 | 59131319 |
| 414758 | 5830428H23Rik | NKLTAG A | MEF A&P | chr19 | 61194953 | 61197453 |
| 547091 | LOC547091 | NONE | MEF A&P | chr19 | 61204526 | 61207026 |
| 209630 | Frmd4a | NONE | MEF A&P | chr2 | 4068143 | 4070643 |
| 18761 | Prkcq | NONE | MEF A&P | chr2 | 11088234 | 11090734 |
| 18232 | Nxph2 | NONE | MEF A&P | chr2 | 23141254 | 23143754 |
| 140483 | Hnmt | NONE | MEF A&P | chr2 | 23870885 | 23873385 |
| 215257 | Il1f9 | NONE | MEF A&P | chr2 | 24007167 | 24009667 |
| 54448 | Il1f6 | NONE | MEF A&P | chr2 | 24035425 | 24037925 |
| 258949 | Olfr338 | NONE | MEF A&P | chr2 | 36196786 | 36199286 |
| 258951 | Olfr339 | NONE | MEF A&P | chr2 | 36241408 | 36243908 |
| 258953 | Olfr340 | NONE | MEF A&P | chr2 | 36272595 | 36275095 |
| 258952 | Olfr341 | NONE | MEF A&P | chr2 | 36301637 | 36304137 |
| 258950 | Olfr342 | NONE | MEF A&P | chr2 | 36347422 | 36349922 |
| 258621 | Olfr344 | NONE | MEF A&P | chr2 | 36388608 | 36391108 |
| 258947 | Olfr345 | NONE | MEF A&P | chr2 | 36460049 | 36462549 |
| 258940 | Olfr346 | NONE | MEF A&P | chr2 | 36508012 | 36510512 |
| 258945 | Olfr347 | NONE | MEF A&P | chr2 | 36554331 | 36556831 |
| 258946 | Olfr348 | NONE | MEF A&P | chr2 | 36606535 | 36609035 |
| 18350 | Olfr50 | NONE | MEF A&P | chr2 | 36613246 | 36615746 |
| 18328 | Olfr3 | NONE | MEF A&P | chr2 | 36634599 | 36637099 |
| 258620 | Olfr350 | NONE | MEF A&P | chr2 | 36670056 | 36672556 |
| 258944 | Olfr351 | NONE | MEF A&P | chr2 | 36681855 | 36684355 |
| 258942 | Olfr352 | NONE | MEF A&P | chr2 | 36689576 | 36692076 |
| 258943 | Olfr353 | NONE | MEF A&P | chr2 | 36712355 | 36714855 |
| 258618 | Olfr355 | NONE | MEF A&P | chr2 | 36749621 | 36752121 |
| 258617 | Olfr356 | NONE | MEF A&P | chr2 | 36757129 | 36759629 |
| 227789 | Olfr358 | NONE | MEF A&P | chr2 | 36827121 | 36829621 |
| 258615 | Olfr360 | NKLTAG A | MEF A&P | chr2 | 36888315 | 36890815 |
| 258365 | Olfr361 | NONE | MEF A&P | chr2 | 36907255 | 36909755 |
| 259053 | Olfr362 | NONE | MEF A&P | chr2 | 36927157 | 36929657 |
| 258656 | Olfr365 | NONE | MEF A&P | chr2 | 37021251 | 37023751 |
| 236509 | Olfr366 | NONE | MEF A&P | chr2 | 37039499 | 37041999 |
| 94217 | Lrp1b | NONE | MEF A&P | chr2 | 42475107 | 42477607 |
| 70789 | Kynu | NONE | MEF A&P | chr2 | 43375337 | 43377837 |
| 16519 | Kcnj3 | NKLTAG A | MEF A&P | chr2 | 55250541 | 55253041 |
| 241391 | Galnt5 | NONE | MEF A&P | chr2 | 57811269 | 57813769 |
| 75721 | 4932414N04Rik | NONE | MEF A&P | chr2 | 68456722 | 68459222 |
| 22138 | Ttn | NONE | MEF A&P | chr2 | 76782886 | 76785386 |
| 241528 | Lrrc55 | NONE | MEF A&P | chr2 | 84997038 | 84999538 |
| 257951 | Olfr987 | NONE | MEF A&P | chr2 | 85132235 | 85134735 |
| 258166 | Olfr988 | NONE | MEF A&P | chr2 | 85154263 | 85156763 |
| 258865 | Olfr992 | NONE | MEF A&P | chr2 | 85200870 | 85203370 |
| 258425 | Olfr994 | NONE | MEF A&P | chr2 | 85231166 | 85233666 |
| 258428 | Olfr998 | NONE | MEF A&P | chr2 | 85389380 | 85391880 |
| 257899 | Olfr1000 | NKLTAG A | MEF A&P | chr2 | 85409247 | 85411747 |
| 258566 | Olfr1002 | NONE | MEF A&P | chr2 | 85448658 | 85451158 |
| 27216 | Olfr154 | NONE | MEF A&P | chr2 | 85464771 | 85467271 |
| 258563 | Olfr1006 | NONE | MEF A&P | chr2 | 85475512 | 85478012 |
| 258866 | Olfr1008 | NONE | MEF A&P | chr2 | 85488269 | 85490769 |
| 258255 | Olfr1010 | NONE | MEF A&P | chr2 | 85552201 | 85554701 |
| 258561 | Olfr1012 | NONE | MEF A&P | chr2 | 85560713 | 85563213 |
| 258757 | Olfr1013 | NONE | MEF A&P | chr2 | 85568641 | 85571141 |
| 258562 | Olfr1014 | NONE | MEF A&P | chr2 | 85575424 | 85577924 |
| 258564 | Olfr1015 | NONE | MEF A&P | chr2 | 85584351 | 85586851 |
| 258579 | Olfr1018 | NONE | MEF A&P | chr2 | 85621811 | 85624311 |
| 259017 | Olfr1019 | NONE | MEF A&P | chr2 | 85642128 | 85644628 |
| 258573 | Olfr1020 | NONE | MEF A&P | chr2 | 85648292 | 85650792 |
| 258582 | Olfr1022 | NONE | MEF A&P | chr2 | 85667432 | 85669932 |
| 258580 | Olfr1023 | NONE | MEF A&P | chr2 | 85685640 | 85688140 |
| 257900 | Olfr1024 | NONE | MEF A&P | chr2 | 85705391 | 85707891 |
| 257936 | Olfr1028 | NONE | MEF A&P | chr2 | 85749903 | 85752403 |
| 258581 | Olfr1030 | NONE | MEF A&P | chr2 | 85782680 | 85785180 |
| 258216 | Olfr1034 | NONE | MEF A&P | chr2 | 85845322 | 85847822 |
| 258245 | Olfr1036 | NONE | MEF A&P | chr2 | 85873580 | 85876080 |
| 259151 | Olfr1037 | NONE | MEF A&P | chr2 | 85886114 | 85888614 |
| 259015 | Olfr1038 | NONE | MEF A&P | chr2 | 85920763 | 85923263 |
| 258578 | Olfr1517 | NONE | MEF A&P | chr2 | 85932000 | 85934500 |
| 404323 | Olfr1040 | NONE | MEF A&P | chr2 | 85947071 | 85949571 |
| 257941 | Olfr1042\|Olfr1043 | NONE | MEF A&P | chr2 | 85960707 | 85963207 |
| 258570 | Olfr1043 | NONE | MEF A&P | chr2 | 85963286 | 85965786 |
| 259013 | Olfr1044 | NONE | MEF A&P | chr2 | 85972154 | 85974654 |
| 18352 | Olfr52 | NONE | MEF A&P | chr2 | 85982448 | 85984948 |
| 259019 | Olfr1045 | NKLTAG A | MEF A&P | chr2 | 85999089 | 86001589 |
| 258575 | Olfr1046 | NONE | MEF A&P | chr2 | 86018047 | 86020547 |
| 259018 | Olfr1049 | NONE | MEF A&P | chr2 | 86056030 | 86058530 |
| 404324 | Olfr1051 | NONE | MEF A&P | chr2 | 86076824 | 86079324 |
| 259020 | Olfr1056 | NONE | MEF A&P | chr2 | 86156719 | 86159219 |
| 404325 | Olfr1057 | NKLTAG A | MEF A&P | chr2 | 86175749 | 86178249 |
| 258386 | Olfr1058 | NONE | MEF A&P | chr2 | 86186755 | 86189255 |
| 258234 | Olfr1061 | NONE | MEF A&P | chr2 | 86214389 | 86216889 |
| 259082 | Olfr1062 | NKLTAG A | MEF A&P | chr2 | 86224013 | 86226513 |
| 258403 | Olfr1065 | NONE | MEF A&P | chr2 | 86246319 | 86248819 |
| 257880 | Olfr1066 | NONE | MEF A&P | chr2 | 86256608 | 86259108 |
| 258400 | Olfr228 | NONE | MEF A&P | chr2 | 86284079 | 86286579 |
| 258402 | Olfr1079 | NONE | MEF A&P | chr2 | 86339252 | 86341752 |
| 258404 | Olfr1080 | NONE | MEF A&P | chr2 | 86354461 | 86356961 |
| 404473 | Olfr1082 | NONE | MEF A&P | chr2 | 86395165 | 86397665 |
| 404326 | Olfr1083 | NONE | MEF A&P | chr2 | 86407908 | 86410408 |
| 258235 | Olfr1084 | NONE | MEF A&P | chr2 | 86440045 | 86442545 |
| 258583 | Olfr1085 | NONE | MEF A&P | chr2 | 86458795 | 86461295 |
| 258585 | Olfr1086 | NONE | MEF A&P | chr2 | 86477670 | 86480170 |
| 258843 | Olfr1087 | NONE | MEF A&P | chr2 | 86491312 | 86493812 |
| 257933 | Olfr1089 | NONE | MEF A&P | chr2 | 86533949 | 86536449 |
| 258844 | Olfr1090 | NONE | MEF A&P | chr2 | 86555075 | 86557575 |
| 258363 | Olfr1093 | NONE | MEF A&P | chr2 | 86584570 | 86587070 |
| 258725 | Olfr1095 | NONE | MEF A&P | chr2 | 86652035 | 86654535 |
| 258840 | Olfr1097 | NKLTAG A | MEF A&P | chr2 | 86691512 | 86694012 |
| 258842 | Olfr1098 | NKLTAG A | MEF A&P | chr2 | 86723847 | 86726347 |
| 258764 | Olfr1099 | NONE | MEF A&P | chr2 | 86759795 | 86762295 |
| 258587 | Olfr1100 | NONE | MEF A&P | chr2 | 86779133 | 86781633 |
| 258584 | Olfr1101 | NONE | MEF A&P | chr2 | 86789513 | 86792013 |
| 228228 | Olfr1102 | NONE | MEF A&P | chr2 | 86800809 | 86803309 |
| 258763 | Olfr1104 | NONE | MEF A&P | chr2 | 86822881 | 86825381 |
| 258747 | Olfr1106 | NONE | MEF A&P | chr2 | 86849573 | 86852073 |
| 258841 | Olfr1107 | NONE | MEF A&P | chr2 | 86872471 | 86874971 |
| 258762 | Olfr1109 | NONE | MEF A&P | chr2 | 86893734 | 86896234 |
| 258766 | Olfr259 | NONE | MEF A&P | chr2 | 86908724 | 86911224 |
| 258765 | Olfr1110 | NONE | MEF A&P | chr2 | 86936658 | 86939158 |
| 258586 | Olfr1111 | NONE | MEF A&P | chr2 | 86950998 | 86953498 |
| 258655 | Olfr1112 | NONE | MEF A&P | chr2 | 86990527 | 86993027 |
| 404327 | Olfr1113 | NONE | MEF A&P | chr2 | 87011732 | 87014232 |
| 258294 | Olfr1115 | NONE | MEF A&P | chr2 | 87050777 | 87053277 |
| 257875 | Olfr1116 | NONE | MEF A&P | chr2 | 87067684 | 87070184 |
| 259031 | Olfr1120 | NONE | MEF A&P | chr2 | 87156284 | 87158784 |
| 258347 | Olfr1123 | NONE | MEF A&P | chr2 | 87216888 | 87219388 |
| 259030 | Olfr1125 | NONE | MEF A&P | chr2 | 87233327 | 87235827 |
| 347712 | Pramel7 | NONE | MEF A&P | chr2 | 87292757 | 87295257 |
| 347711 | Pramel6 | NONE | MEF A&P | chr2 | 87307296 | 87309796 |
| 258346 | Olfr1128 | NONE | MEF A&P | chr2 | 87345881 | 87348381 |
| 258111 | Olfr1129 | NONE | MEF A&P | chr2 | 87373924 | 87376424 |
| 258835 | Olfr1130 | NONE | MEF A&P | chr2 | 87406228 | 87408728 |
| 258348 | Olfr1133 | NONE | MEF A&P | chr2 | 87446460 | 87448960 |
| 259032 | Olfr1134 | NONE | MEF A&P | chr2 | 87457258 | 87459758 |
| 258653 | Olfr1136 | NONE | MEF A&P | chr2 | 87494219 | 87496719 |
| 258101 | Olfr1137 | NONE | MEF A&P | chr2 | 87512243 | 87514743 |
| 258632 | Olfr1138 | NONE | MEF A&P | chr2 | 87538661 | 87541161 |
| 258635 | Olfr1140 | NONE | MEF A&P | chr2 | 87545036 | 87547536 |
| 258640 | Olfr152 | NONE | MEF A&P | chr2 | 87581374 | 87583874 |
| 258290 | Olfr1143 | NONE | MEF A&P | chr2 | 87601229 | 87603729 |
| 258220 | Olfr1148 | NONE | MEF A&P | chr2 | 87631879 | 87634379 |
| 258103 | Olfr1152 | NONE | MEF A&P | chr2 | 87666831 | 87669331 |
| 258641 | Olfr1154 | NONE | MEF A&P | chr2 | 87704013 | 87706513 |
| 258636 | Olfr1155 | NONE | MEF A&P | chr2 | 87743965 | 87746465 |
| 258814 | Olfr1156 | NONE | MEF A&P | chr2 | 87750570 | 87753070 |
| 117005 | Olfr74 | NONE | MEF A&P | chr2 | 87775002 | 87777502 |
| 258639 | Olfr1158 | NONE | MEF A&P | chr2 | 87788951 | 87791451 |
| 258643 | Olfr1160 | NONE | MEF A&P | chr2 | 87807115 | 87809615 |
| 117004 | Olfr73 | NONE | MEF A&P | chr2 | 87835476 | 87837976 |
| 258105 | Olfr1162 | NONE | MEF A&P | chr2 | 87850961 | 87853461 |
| 258638 | Olfr1163 | NONE | MEF A&P | chr2 | 87871719 | 87874219 |
| 258634 | Olfr1164 | NONE | MEF A&P | chr2 | 87894273 | 87896773 |
| 258642 | Olfr1165 | NONE | MEF A&P | chr2 | 87902324 | 87904824 |
| 258644 | Olfr1166 | NONE | MEF A&P | chr2 | 87925332 | 87927832 |
| 258291 | Olfr1167 | NONE | MEF A&P | chr2 | 87950356 | 87952856 |
| 258524 | Olfr1168 | NONE | MEF A&P | chr2 | 87983717 | 87986217 |
| 258525 | Olfr1170 | NONE | MEF A&P | chr2 | 88025369 | 88027869 |
| 404329 | Olfr1173 | NONE | MEF A&P | chr2 | 88075386 | 88077886 |
| 258767 | Olfr1176 | NONE | MEF A&P | chr2 | 88138405 | 88140905 |
| 258203 | Olfr1178 | NONE | MEF A&P | chr2 | 88190087 | 88192587 |
| 258919 | Olfr1179 | NONE | MEF A&P | chr2 | 88203271 | 88205771 |
| 258920 | Olfr1180 | NONE | MEF A&P | chr2 | 88212995 | 88215495 |
| 258167 | Olfr1182 | NONE | MEF A&P | chr2 | 88247275 | 88249775 |
| 258522 | Olfr1183 | NONE | MEF A&P | chr2 | 88260180 | 88262680 |
| 258820 | Olfr1184 | NONE | MEF A&P | chr2 | 88285572 | 88288072 |
| 258523 | Olfr1186 | NONE | MEF A&P | chr2 | 88324423 | 88326923 |
| 258921 | Olfr1188 | NONE | MEF A&P | chr2 | 88358309 | 88360809 |
| 329460 | Olfr1193 | NONE | MEF A&P | chr2 | 88476695 | 88479195 |
| 258748 | Olfr1195 | NONE | MEF A&P | chr2 | 88484069 | 88486569 |
| 258456 | Olfr1196 | NONE | MEF A&P | chr2 | 88501687 | 88504187 |
| 433449 | Olfr1197 | NKLTAG A | MEF A&P | chr2 | 88529936 | 88532436 |
| 404330 | Olfr1198 | NONE | MEF A&P | chr2 | 88547225 | 88549725 |
| 258450 | Olfr1199 | NONE | MEF A&P | chr2 | 88557012 | 88559512 |
| 257887 | Olfr1200 | NONE | MEF A&P | chr2 | 88568652 | 88571152 |
| 258454 | Olfr1202 | NONE | MEF A&P | chr2 | 88616011 | 88618511 |
| 258898 | Olfr1203 | NONE | MEF A&P | chr2 | 88629957 | 88632457 |
| 258455 | Olfr1204 | NONE | MEF A&P | chr2 | 88650790 | 88653290 |
| 258896 | Olfr1206 | NONE | MEF A&P | chr2 | 88663445 | 88665945 |
| 258774 | Olfr1208 | NONE | MEF A&P | chr2 | 88697934 | 88700434 |
| 258453 | Olfr1209 | NONE | MEF A&P | chr2 | 88710730 | 88713230 |
| 258025 | Olfr1211 | NONE | MEF A&P | chr2 | 88730652 | 88733152 |
| 258241 | Olfr1212 | NONE | MEF A&P | chr2 | 88757306 | 88759806 |
| 258900 | Olfr1213 | NONE | MEF A&P | chr2 | 88774228 | 88776728 |
| 258899 | Olfr1214 | NONE | MEF A&P | chr2 | 88788539 | 88791039 |
| 258451 | Olfr1215 | NONE | MEF A&P | chr2 | 88802625 | 88805125 |
| 258903 | Olfr1217 | NONE | MEF A&P | chr2 | 88824340 | 88826840 |
| 258901 | Olfr1219 | NONE | MEF A&P | chr2 | 88875428 | 88877928 |
| 258902 | Olfr1220 | NONE | MEF A&P | chr2 | 88898264 | 88900764 |
| 258904 | Olfr1221 | NONE | MEF A&P | chr2 | 88912849 | 88915349 |
| 258177 | Olfr1222 | NONE | MEF A&P | chr2 | 88926068 | 88928568 |
| 258894 | Olfr1223 | NONE | MEF A&P | chr2 | 88945360 | 88947860 |
| 258969 | Olfr1226 | NONE | MEF A&P | chr2 | 88994371 | 88996871 |
| 258973 | Olfr1228 | NONE | MEF A&P | chr2 | 89050031 | 89052531 |
| 257921 | Olfr1229 | NONE | MEF A&P | chr2 | 89083470 | 89085970 |
| 258785 | Olfr1230 | NONE | MEF A&P | chr2 | 89097607 | 89100107 |
| 258446 | Olfr1231 | NONE | MEF A&P | chr2 | 89103929 | 89106429 |
| 258320 | Olfr1232 | NONE | MEF A&P | chr2 | 89126517 | 89129017 |
| 258974 | Olfr1233 | NONE | MEF A&P | chr2 | 89140639 | 89143139 |
| 258975 | Olfr1234 | NONE | MEF A&P | chr2 | 89163766 | 89166266 |
| 258786 | Olfr1238 | NONE | MEF A&P | chr2 | 89207416 | 89209916 |
| 258804 | Olfr1240 | NONE | MEF A&P | chr2 | 89240616 | 89243116 |
| 258447 | Olfr1241 | NONE | MEF A&P | chr2 | 89283472 | 89285972 |
| 258970 | Olfr1242 | NONE | MEF A&P | chr2 | 89294649 | 89297149 |
| 258971 | Olfr1243 | NONE | MEF A&P | chr2 | 89328747 | 89331247 |
| 258784 | Olfr1245 | NONE | MEF A&P | chr2 | 89376063 | 89378563 |
| 258788 | Olfr1246 | NONE | MEF A&P | chr2 | 89391452 | 89393952 |
| 258787 | Olfr1248 | NONE | MEF A&P | chr2 | 89418529 | 89421029 |
| 257984 | Olfr1249 | NONE | MEF A&P | chr2 | 89431235 | 89433735 |
| 258967 | Olfr1250 | NONE | MEF A&P | chr2 | 89457778 | 89460278 |
| 259145 | Olfr1251 | NONE | MEF A&P | chr2 | 89468223 | 89470723 |
| 404331 | Olfr1252 | NONE | MEF A&P | chr2 | 89522448 | 89524948 |
| 258468 | Olfr1254 | NONE | MEF A&P | chr2 | 89589689 | 89592189 |
| 258979 | Olfr1255 | NONE | MEF A&P | chr2 | 89615166 | 89617666 |
| 258985 | Olfr1256 | NONE | MEF A&P | chr2 | 89636282 | 89638782 |
| 258984 | Olfr1257 | NONE | MEF A&P | chr2 | 89679666 | 89682166 |
| 258980 | Olfr1258 | NONE | MEF A&P | chr2 | 89728649 | 89731149 |
| 258338 | Olfr1259 | NONE | MEF A&P | chr2 | 89744452 | 89746952 |
| 258466 | Olfr1261 | NONE | MEF A&P | chr2 | 89792233 | 89794733 |
| 258976 | Olfr1262 | NONE | MEF A&P | chr2 | 89801246 | 89803746 |
| 258790 | Olfr1263 | NONE | MEF A&P | chr2 | 89813770 | 89816270 |
| 258340 | Olfr1265 | NONE | MEF A&P | chr2 | 89835759 | 89838259 |
| 258339 | Olfr1269 | NONE | MEF A&P | chr2 | 89919935 | 89922435 |
| 18331 | Olfr32 | NONE | MEF A&P | chr2 | 89939476 | 89941976 |
| 258987 | Olfr1270 | NONE | MEF A&P | chr2 | 89950343 | 89952843 |
| 257665 | Olfr1506 | NKLTAG A | MEF A&P | chr2 | 90022835 | 90025335 |
| 406186 | Olfr142 | NONE | MEF A&P | chr2 | 90053325 | 90055825 |
| 258789 | Olfr1271 | NONE | MEF A&P | chr2 | 90066767 | 90069267 |
| 258982 | Olfr1272 | NONE | MEF A&P | chr2 | 90082912 | 90085412 |
| 258977 | Olfr1273 | NONE | MEF A&P | chr2 | 90097198 | 90099698 |
| 668629 | LOC668629\|Olfr1274 | NONE | MEF A&P | chr2 | 90199501 | 90202001 |
| 269328 | Muc15 | NONE | MEF A&P | chr2 | 110520446 | 110522946 |
| 67575 | 4930430A15Rik\|Olfr1275 | NONE | MEF A&P | chr2 | 111029937 | 111034630 |
| 258390 | Olfr1276 | NKLTAG A | MEF A&P | chr2 | 111055955 | 111058455 |
| 258389 | Olfr1278 | NONE | MEF A&P | chr2 | 111091108 | 111093608 |
| 258388 | Olfr1279 | NONE | MEF A&P | chr2 | 111105039 | 111107539 |
| 258910 | Olfr1280 | NONE | MEF A&P | chr2 | 111114319 | 111116819 |
| 257979 | Olfr1281 | NKLTAG A | MEF A&P | chr2 | 111127259 | 111129759 |
| 258909 | Olfr1282 | NONE | MEF A&P | chr2 | 111136415 | 111138915 |
| 228443 | Olfr1283 | NONE | MEF A&P | chr2 | 111167472 | 111169972 |
| 258379 | Olfr1284 | NONE | MEF A&P | chr2 | 111177840 | 111180340 |
| 277562 | Olfr1286 | NONE | MEF A&P | chr2 | 111221288 | 111223788 |
| 257935 | Olfr1287 | NONE | MEF A&P | chr2 | 111247980 | 111250480 |
| 257662 | GA_x5J8B7W4T2P-84764-83847\|Olfr1290 | NONE | MEF A&P | chr2 | 111290474 | 111292974 |
| 258887 | Olfr1294 | NONE | MEF A&P | chr2 | 111338626 | 111341126 |
| 258398 | Olfr1295 | NKLTAG A | MEF A&P | chr2 | 111365781 | 111368281 |
| 258890 | Olfr1297 | NONE | MEF A&P | chr2 | 111422411 | 111424911 |
| 258888 | Olfr1298 | NONE | MEF A&P | chr2 | 111446334 | 111448834 |
| 258886 | Olfr1299 | NKLTAG A | MEF A&P | chr2 | 111463079 | 111465579 |
| 258889 | Olfr1301 | NONE | MEF A&P | chr2 | 111553089 | 111555589 |
| 258891 | Olfr1302 | NONE | MEF A&P | chr2 | 111579151 | 111581651 |
| 258397 | Olfr1303 | NONE | MEF A&P | chr2 | 111615063 | 111617563 |
| 258023 | Olfr1306 | NONE | MEF A&P | chr2 | 111713267 | 111715767 |
| 257956 | Olfr1307 | NONE | MEF A&P | chr2 | 111745793 | 111748293 |
| 258258 | Olfr1308\|N/A | NONE | MEF A&P | chr2 | 111761410 | 111763910 |
| 258439 | Olfr1309 | NONE | MEF A&P | chr2 | 111784435 | 111786935 |
| 258441 | Olfr1310 | NONE | MEF A&P | chr2 | 111809523 | 111812023 |
| 258271 | Olfr1311 | NONE | MEF A&P | chr2 | 111822191 | 111824691 |
| 258359 | Olfr1312 | NONE | MEF A&P | chr2 | 111843369 | 111845869 |
| 258257 | Olfr1313 | NONE | MEF A&P | chr2 | 111872920 | 111875420 |
| 258442 | Olfr1314 | NONE | MEF A&P | chr2 | 111893038 | 111895538 |
| 258737 | Olfr1316 | NONE | MEF A&P | chr2 | 111931154 | 111933654 |
| 16176 | Il1b\|9430083I22 | NONE | MEF A&P | chr2 | 129059337 | 129061837 |
| 16176 | Il1b\|9430083I22 | NONE | MEF A&P | chr2 | 129062061 | 129064561 |
| 18610 | Pdyn | NONE | MEF A&P | chr2 | 129390774 | 129393274 |
| 241636 | Tgm6 | NONE | MEF A&P | chr2 | 129812725 | 129815225 |
| 192140 | Tmc2 | NKLTAG A | MEF A&P | chr2 | 129884634 | 129887134 |
| 76161 | 6330527O06Rik | NONE | MEF A&P | chr2 | 135747367 | 135749867 |
| 241656 | Pak7 | NONE | MEF A&P | chr2 | 136078879 | 136081379 |
| 228677 | C130053K05Rik | NONE | MEF A&P | chr2 | 139183360 | 139185860 |
| 545471 | LOC545471 | NKLTAG A | MEF A&P | chr2 | 150176004 | 150178504 |
| 13011 | Cst7 | NONE | MEF A&P | chr2 | 150259874 | 150262374 |
| 381393 | 4921509C19Rik | NONE | MEF A&P | chr2 | 151167092 | 151169592 |
| 386649 | Nsfl1c | NONE | MEF A&P | chr2 | 151183748 | 151186248 |
| 654457 | Defb26 | NKLTAG A | MEF A&P | chr2 | 152202633 | 152205133 |
| 545475 | Defb28 | NKLTAG A | MEF A&P | chr2 | 152207695 | 152210195 |
| 76407 | Spag4l\|Bpil1 | NONE | MEF A&P | chr2 | 153562025 | 153567013 |
| 378700 | Rya3 | NONE | MEF A&P | chr2 | 153607670 | 153610170 |
| 71425 | 5430413K10Rik | NONE | MEF A&P | chr2 | 153947319 | 153949819 |
| 433492 | LOC433492 | NONE | MEF A&P | chr2 | 153996684 | 153999184 |
| 192201 | Wfdc15 | NONE | MEF A&P | chr2 | 163912601 | 163915101 |
| 53878 | Svs2 | NONE | MEF A&P | chr2 | 163929206 | 163931706 |
| 20941 | Svp2 | NONE | MEF A&P | chr2 | 163969248 | 163971748 |
| 20945 | Svs6 | NONE | MEF A&P | chr2 | 164006187 | 164008687 |
| 433501 | LOC433501 | NONE | MEF A&P | chr2 | 164213409 | 164215909 |
| 228911 | Tshz2 | NONE | MEF A&P | chr2 | 169322880 | 169325380 |
| 446211 | AY702102 | NONE | MEF A&P | chr2 | 169653892 | 169656392 |
| 67637 | 4930470P17Rik | NONE | MEF A&P | chr2 | 170292722 | 170295222 |
| 76829 | Dok5 | NONE | MEF A&P | chr2 | 170421144 | 170423644 |
| 228942 | Cbln4 | NONE | MEF A&P | chr2 | 171734171 | 171736671 |
| 76958 | 2210418O10Rik | NKLTAG A | MEF A&P | chr2 | 175396971 | 175399471 |
| 76958 | 2210418O10Rik | NKLTAG A | MEF A&P | chr2 | 176500911 | 176503411 |
| 433520 | LOC433520 | NKLTAG A | MEF A&P | chr2 | 177475633 | 177478133 |
| 74189 | Phactr3 | NONE | MEF A&P | chr2 | 178102756 | 178105256 |
| 68175 | 4930591A17Rik | NONE | MEF A&P | chr2 | 179342343 | 179344843 |
| 17932 | Myt1 | NONE | MEF A&P | chr2 | 181694447 | 181696947 |
| 71710 | Lrrcc1 | NONE | MEF A&P | chr3 | 14508531 | 14511031 |
| 320832 | Sirpb1 | NONE | MEF A&P | chr3 | 15402671 | 15405248 |
| 192167 | Nlgn1 | NONE | MEF A&P | chr3 | 26522482 | 26524982 |
| 70862 | Spata16 | NONE | MEF A&P | chr3 | 26826703 | 26829239 |
| 385263 | Gm1527 | NONE | MEF A&P | chr3 | 29081689 | 29084189 |
| 58869 | Pex2 | NONE | MEF A&P | chr3 | 33274078 | 33276578 |
| 229214 | Gpr103 | NONE | MEF A&P | chr3 | 36413324 | 36415824 |
| 21744 | Tenr | NONE | MEF A&P | chr3 | 37253217 | 37255717 |
| 242022 | Frem2 | NONE | MEF A&P | chr3 | 53744513 | 53747013 |
| 329641 | 6030405A18Rik | NONE | MEF A&P | chr3 | 55003316 | 55005816 |
| 56544 | V2R2 | NONE | MEF A&P | chr3 | 64167570 | 64170070 |
| 319210 | 4930518C23Rik | NONE | MEF A&P | chr3 | 64744913 | 64747413 |
| 229389 | Gm414 | NONE | MEF A&P | chr3 | 70093541 | 70096041 |
| 12038 | Bche | NONE | MEF A&P | chr3 | 73793668 | 73796168 |
| 213234 | BC050789 | NONE | MEF A&P | chr3 | 75229149 | 75231649 |
| 67931 | Serpini2 | NONE | MEF A&P | chr3 | 75355504 | 75358004 |
| 381489 | Lgr7 | NONE | MEF A&P | chr3 | 79823221 | 79825721 |
| 60596 | Gucy1a3 | NONE | MEF A&P | chr3 | 82230500 | 82233000 |
| 79235 | Lrat | NONE | MEF A&P | chr3 | 82989401 | 82991901 |
| 14161 | Fga | NONE | MEF A&P | chr3 | 83110080 | 83112580 |
| 12095 | Bglap-rs1 | NONE | MEF A&P | chr3 | 88455139 | 88457639 |
| 20755 | Sprr2a | NKLTAG A | MEF A&P | chr3 | 92299237 | 92301737 |
| 69611 | Sprrl7 | NONE | MEF A&P | chr3 | 92772113 | 92774613 |
| 66195 | 1110058A15Rik | NONE | MEF A&P | chr3 | 92837241 | 92839741 |
| 229571 | LOC229571 | NONE | MEF A&P | chr3 | 93152225 | 93154725 |
| 229574 | Flg2 | NONE | MEF A&P | chr3 | 93280675 | 93283175 |
| 20129 | Rptn | NONE | MEF A&P | chr3 | 93477102 | 93479602 |
| 399673 | Tdpoz2 | NONE | MEF A&P | chr3 | 93737567 | 93740067 |
| 207213 | Tdpoz1 | NONE | MEF A&P | chr3 | 93761172 | 93763672 |
| 399675 | Tdpoz4 | NONE | MEF A&P | chr3 | 93879801 | 93882301 |
| 399674 | Tdpoz3 | NONE | MEF A&P | chr3 | 93909423 | 93911923 |
| 399676 | Tdpoz5 | NKLTAG A | MEF A&P | chr3 | 94046218 | 94048718 |
| 258272 | Olfr1402 | NONE | MEF A&P | chr3 | 97496084 | 97498584 |
| 15495 | Hsd3b4 | NONE | MEF A&P | chr3 | 98595399 | 98597899 |
| 15496 | Hsd3b5 | NONE | MEF A&P | chr3 | 98774058 | 98776558 |
| 15493 | Hsd3b2 | NONE | MEF A&P | chr3 | 98853104 | 98855604 |
| 15494 | Hsd3b3 | NONE | MEF A&P | chr3 | 98890971 | 98893471 |
| 15497 | Hsd3b6 | NONE | MEF A&P | chr3 | 98942998 | 98945498 |
| 56543 | Kcnd3 | NONE | MEF A&P | chr3 | 105584871 | 105587371 |
| 76420 | 1700027A23Rik | NONE | MEF A&P | chr3 | 106142202 | 106144702 |
| 81600 | Chia | NONE | MEF A&P | chr3 | 106239437 | 106241937 |
| 12655 | Chi3l3 | NONE | MEF A&P | chr3 | 106295077 | 106297577 |
| 104183 | Chi3l4 | NONE | MEF A&P | chr3 | 106347020 | 106349520 |
| 229688 | BC051070 | NONE | MEF A&P | chr3 | 106533738 | 106536238 |
| 320181 | Fndc7 | NONE | MEF A&P | chr3 | 109017426 | 109019926 |
| 11723 | Amy2 | NONE | MEF A&P | chr3 | 113386255 | 113388755 |
| 75769 | 4833424O15Rik | NONE | MEF A&P | chr3 | 117565603 | 117568103 |
| 64580 | Ndst4 | NONE | MEF A&P | chr3 | 125427701 | 125430201 |
| 13645 | Egf | NONE | MEF A&P | chr3 | 129746838 | 129749338 |
| 77018 | Col25a1 | NONE | MEF A&P | chr3 | 130168032 | 130170532 |
| 18598 | Pdha2 | NONE | MEF A&P | chr3 | 141149242 | 141151742 |
| 56376 | Pdlim5 | NONE | MEF A&P | chr3 | 142328736 | 142331236 |
| 65956 | Ccl21c\|Ccl21b\|Ccl21a | NONE | MEF A&P | chr4 | 42013880 | 42016385 |
| 108816 | 4933409K07Rik\|LOC665845 | NONE | MEF A&P | chr4 | 42222351 | 42224851 |
| 18829 | Ccl21b | NONE | MEF A&P | chr4 | 42795251 | 42797751 |
| 230085 | N28178 | NKLTAG A | MEF A&P | chr4 | 42936350 | 42938850 |
| 634450 | LOC634450 | NKLTAG A | MEF A&P | chr4 | 46594243 | 46596743 |
| 272031 | E130309F12Rik | NONE | MEF A&P | chr4 | 49078561 | 49081061 |
| 209186 | C730036D15Rik | NONE | MEF A&P | chr4 | 49428751 | 49431251 |
| 230163 | Aldob | NONE | MEF A&P | chr4 | 49570083 | 49572583 |
| 68274 | 4930547C10Rik | NONE | MEF A&P | chr4 | 52615373 | 52617873 |
| 258821 | Olfr273 | NONE | MEF A&P | chr4 | 52877111 | 52879611 |
| 258836 | Olfr272 | NONE | MEF A&P | chr4 | 52932392 | 52934892 |
| 17841 | Mup2\|Mup1 | NKLTAG A | MEF A&P | chr4 | 60233399 | 60235899 |
| 17841 | Mup2\|Mup1 | NKLTAG A | MEF A&P | chr4 | 60475813 | 60478313 |
| 17844 | Mup5 | NONE | MEF A&P | chr4 | 61321041 | 61323541 |
| 381530 | MGC107671 | NONE | MEF A&P | chr4 | 61539978 | 61542478 |
| 17842 | Mup3 | NONE | MEF A&P | chr4 | 61573173 | 61575673 |
| 381531 | bM64F17.1 | NONE | MEF A&P | chr4 | 61636724 | 61639224 |
| 433719 | RP23-289L21.1 | NONE | MEF A&P | chr4 | 72890933 | 72893433 |
| 381590 | C87499 | NONE | MEF A&P | chr4 | 88104916 | 88107416 |
| 15965 | Ifna2 | NKLTAG A | MEF A&P | chr4 | 88154510 | 88157010 |
| 319247 | 9530080O11Rik\|Hook1 | NONE | MEF A&P | chr4 | 95457396 | 95461673 |
| 13109 | Cyp2j5 | NONE | MEF A&P | chr4 | 96155595 | 96158095 |
| 230500 | BC020077 | NONE | MEF A&P | chr4 | 99373976 | 99376476 |
| 16847 | Lepr | NONE | MEF A&P | chr4 | 101213370 | 101215870 |
| 16847 | Lepr | NONE | MEF A&P | chr4 | 101223961 | 101226461 |
| 545662 | B020004J07Rik | NONE | MEF A&P | chr4 | 101341454 | 101343954 |
| 242574 | C130073F10Rik | NONE | MEF A&P | chr4 | 101391221 | 101393721 |
| 329918 | A030013N09Rik | NONE | MEF A&P | chr4 | 111931417 | 111933917 |
| 230613 | A030001H23Rik | NONE | MEF A&P | chr4 | 112272257 | 112274757 |
| 230623 | A630098G03Rik | NONE | MEF A&P | chr4 | 113659315 | 113661815 |
| 13118 | BC060945 | NONE | MEF A&P | chr4 | 114907555 | 114910055 |
| 13119 | Cyp4a14 | NONE | MEF A&P | chr4 | 114993564 | 114996064 |
| 13117 | Cyp4a10\|BC013476 | NONE | MEF A&P | chr4 | 115014232 | 115016745 |
| 21846 | Tie1 | NONE | MEF A&P | chr4 | 117987493 | 117989993 |
| 66451 | 2610528J11Rik | NONE | MEF A&P | chr4 | 118023206 | 118025706 |
| 258852 | Olfr1341 | NONE | MEF A&P | chr4 | 118205340 | 118207840 |
| 258259 | Olfr1338 | NONE | MEF A&P | chr4 | 118251968 | 118254468 |
| 258306 | Olfr1337 | NKLTAG A | MEF A&P | chr4 | 118280015 | 118282515 |
| 435804 | Olfr1335 | NONE | MEF A&P | chr4 | 118307294 | 118309794 |
| 258265 | Olfr1333 | NONE | MEF A&P | chr4 | 118327870 | 118330370 |
| 258159 | Olfr1331 | NONE | MEF A&P | chr4 | 118364714 | 118367214 |
| 258214 | Olfr1329 | NKLTAG A | MEF A&P | chr4 | 118414897 | 118417397 |
| 404333 | Olfr1328 | NKLTAG A | MEF A&P | chr4 | 118432272 | 118434772 |
| 77432 | 9530002B09Rik | NONE | MEF A&P | chr4 | 122189612 | 122192112 |
| 69317 | Hmgb4 | NONE | MEF A&P | chr4 | 127762697 | 127765197 |
| 67663 | 4930549C01Rik | NKLTAG A | MEF A&P | chr4 | 135880512 | 135883012 |
| 242737 | Oog4 | NONE | MEF A&P | chr4 | 142716489 | 142718989 |
| 545693 | MGC118150 | NONE | MEF A&P | chr4 | 142776029 | 142778529 |
| 329986 | BC080695 | NONE | MEF A&P | chr4 | 142832153 | 142834653 |
| 194227 | MGC91194 | NKLTAG A | MEF A&P | chr4 | 143054048 | 143056548 |
| 381569 | MGC91195 | NKLTAG A | MEF A&P | chr4 | 143091287 | 143093787 |
| 194225 | E330017N17 | NONE | MEF A&P | chr4 | 143111183 | 143113683 |
| 347710 | Pramel5 | NONE | MEF A&P | chr4 | 143323812 | 143326312 |
| 347709 | Pramel4 | NKLTAG A | MEF A&P | chr4 | 143330384 | 143332884 |
| 100012 | Oog3 | NONE | MEF A&P | chr4 | 143428823 | 143431323 |
| 381570 | Oog2 | NONE | MEF A&P | chr4 | 143455415 | 143457915 |
| 433779 | MGC118154 | NONE | MEF A&P | chr4 | 143622650 | 143625150 |
| 381572 | 9430007A20Rik | NONE | MEF A&P | chr4 | 143784508 | 143787008 |
| 433801 | LOC433801 | NKLTAG A | MEF A&P | chr4 | 144881804 | 144884304 |
| 70005 | 1700029I01Rik | NONE | MEF A&P | chr4 | 145054201 | 145056701 |
| 627585 | LOC627585 | NKLTAG A | MEF A&P | chr4 | 145502615 | 145505115 |
| 19715 | Rex2 | NKLTAG A | MEF A&P | chr4 | 145894305 | 145896805 |
| 195531 | LOC195531 | NONE | MEF A&P | chr4 | 146342485 | 146344985 |
| 433804 | N/A\|LOC433804 | NKLTAG A | MEF A&P | chr4 | 146385802 | 146388302 |
| 112422 | 2610305D13Rik | NONE | MEF A&P | chr4 | 146458729 | 146461229 |
| 171266 | V1rg10 | NKLTAG A | MEF A&P | chr5 | 3006564 | 3009064 |
| 19941 | Rpl26 | NONE | MEF A&P | chr5 | 3240395 | 3242895 |
| 70920 | 4921511H03Rik | NONE | MEF A&P | chr5 | 7308169 | 7310669 |
| 109552 | Sri | NONE | MEF A&P | chr5 | 8050083 | 8052583 |
| 231014 | 9330182L06Rik | NONE | MEF A&P | chr5 | 9270165 | 9272665 |
| 667990 | LOC667990\|MGC129481 | NONE | MEF A&P | chr5 | 11598961 | 11601461 |
| 71026 | Speer3 | NONE | MEF A&P | chr5 | 13795637 | 13798137 |
| 381714 | MGC58177 | NKLTAG A | MEF A&P | chr5 | 14920395 | 14922895 |
| 360220 | Speer4d | NKLTAG A | MEF A&P | chr5 | 15128922 | 15131422 |
| 75657 | Speer4a | NONE | MEF A&P | chr5 | 26377841 | 26380341 |
| 381622 | 5031410I06Rik | NKLTAG A | MEF A&P | chr5 | 26435601 | 26438101 |
| 13483 | Dpp6 | NONE | MEF A&P | chr5 | 27146143 | 27148643 |
| 73526 | Speer4b | NONE | MEF A&P | chr5 | 27831729 | 27834229 |
| 15563 | Htr5a | NONE | MEF A&P | chr5 | 28170733 | 28173233 |
| 242939 | Cpz | NONE | MEF A&P | chr5 | 35841896 | 35844396 |
| 76071 | Jakmip1 | NONE | MEF A&P | chr5 | 37337164 | 37339664 |
| 19126 | Prom1\|N/A\|LOC624120 | NKLTAG A | MEF A&P | chr5 | 44386689 | 44389189 |
| 19126 | Prom1\|N/A\|LOC624120 | NKLTAG A | MEF A&P | chr5 | 44389429 | 44391929 |
| 14380 | G6pd2 | NONE | MEF A&P | chr5 | 62095120 | 62097620 |
| 14395 | Gabra2 | NONE | MEF A&P | chr5 | 71374484 | 71376984 |
| 545762 | AU018829 | NONE | MEF A&P | chr5 | 76478389 | 76480889 |
| 243083 | Tmprss11f | NONE | MEF A&P | chr5 | 87631107 | 87633607 |
| 231396 | Ugt2b36 | NONE | MEF A&P | chr5 | 88166616 | 88169116 |
| 22238 | Ugt2b5 | NONE | MEF A&P | chr5 | 88214379 | 88216879 |
| 112417 | Ugt2b37 | NONE | MEF A&P | chr5 | 88328849 | 88331349 |
| 72094 | Ugt2a3 | NONE | MEF A&P | chr5 | 88411237 | 88413737 |
| 100559 | Ugt2b38 | NONE | MEF A&P | chr5 | 88498249 | 88500749 |
| 552899 | Ugt2a2 | NONE | MEF A&P | chr5 | 88556319 | 88558819 |
| 94215 | Ugt2a1 | NONE | MEF A&P | chr5 | 88560807 | 88563307 |
| 20860 | Sult1e1 | NONE | MEF A&P | chr5 | 88665655 | 88668155 |
| 12993 | Csng | NONE | MEF A&P | chr5 | 88847127 | 88849627 |
| 12992 | Csnd | NONE | MEF A&P | chr5 | 88880715 | 88883215 |
| 73779 | 4930432K09Rik | NONE | MEF A&P | chr5 | 88898257 | 88900757 |
| 654494 | LOC654494 | NONE | MEF A&P | chr5 | 88922919 | 88925419 |
| 70977 | 4931407G18Rik | NONE | MEF A&P | chr5 | 89052011 | 89054511 |
| 20601 | Smr3\|Smr1 | NONE | MEF A&P | chr5 | 89075109 | 89077609 |
| 20600 | Smr2 | NONE | MEF A&P | chr5 | 89159116 | 89161616 |
| 17830 | Muc10 | NONE | MEF A&P | chr5 | 89389872 | 89392372 |
| 11698 | Ambn | NONE | MEF A&P | chr5 | 89528571 | 89531071 |
| 13801 | Enam | NONE | MEF A&P | chr5 | 89560535 | 89563035 |
| 11657 | Alb1 | NONE | MEF A&P | chr5 | 91534100 | 91536600 |
| 280662 | Afm | NONE | MEF A&P | chr5 | 91592162 | 91594662 |
| 19023 | Ppef2 | NONE | MEF A&P | chr5 | 93330978 | 93333478 |
| 381724 | BC061212 | NONE | MEF A&P | chr5 | 95145250 | 95147750 |
| 433920 | E330014E10Rik\|D5Ertd577e | NKLTAG A | MEF A&P | chr5 | 95695107 | 95697607 |
| 626391 | LOC626391 | NONE | MEF A&P | chr5 | 105099872 | 105102372 |
| 17472 | Mpa2 | NONE | MEF A&P | chr5 | 105379346 | 105381846 |
| 626578 | LOC626578 | NKLTAG A | MEF A&P | chr5 | 105479335 | 105481835 |
| 67366 | 1810008K16Rik | NONE | MEF A&P | chr5 | 109035165 | 109037665 |
| 22307 | V2r16 | NONE | MEF A&P | chr5 | 109246273 | 109248773 |
| 269693 | Ccdc60 | NKLTAG A | MEF A&P | chr5 | 116549484 | 116551984 |
| 57749 | Piwil1 | NONE | MEF A&P | chr5 | 129048953 | 129051453 |
| 381673 | A330070K13Rik | NONE | MEF A&P | chr5 | 130668834 | 130671334 |
| 68929 | Mospd3 | NONE | MEF A&P | chr5 | 137830328 | 137832828 |
| 545812 | LOC545812 | NKLTAG A | MEF A&P | chr5 | 138101046 | 138103546 |
| 13113 | Cyp3a13 | NONE | MEF A&P | chr5 | 138150905 | 138153405 |
| 118446 | Gje1 | NONE | MEF A&P | chr5 | 138192247 | 138194747 |
| 12007 | Azgp1 | NONE | MEF A&P | chr5 | 138209347 | 138211847 |
| 622474 | 4931427P12Rik\|LOC545814 | NKLTAG A | MEF A&P | chr5 | 138265011 | 138267511 |
| 76658 | 1700123K08Rik | NKLTAG A | MEF A&P | chr5 | 138793982 | 138796482 |
| 108723 | Card11 | NKLTAG A | MEF A&P | chr5 | 141252466 | 141254966 |
| 330222 | Sdk1 | NONE | MEF A&P | chr5 | 142107260 | 142109760 |
| 53324 | Nptx2 | NONE | MEF A&P | chr5 | 144797429 | 144799929 |
| 13114 | Cyp3a16 | NKLTAG A | MEF A&P | chr5 | 145722721 | 145725221 |
| 53973 | Cyp3a41 | NONE | MEF A&P | chr5 | 146023870 | 146026370 |
| 337924 | Cyp3a44 | NONE | MEF A&P | chr5 | 146109608 | 146112108 |
| 13112 | Cyp3a11 | NONE | MEF A&P | chr5 | 146183588 | 146186088 |
| 56388 | Cyp3a25 | NKLTAG A | MEF A&P | chr5 | 146313342 | 146315842 |
| 69282 | 1700001J03Rik | NKLTAG A | MEF A&P | chr5 | 146489038 | 146491538 |
| 245880 | Wasf3 | NONE | MEF A&P | chr5 | 146687239 | 146689739 |
| 14738 | Gpr12 | NONE | MEF A&P | chr5 | 146894253 | 146897588 |
| 74068 | Asz1 | NONE | MEF A&P | chr6 | 18058555 | 18061055 |
| 30785 | Cttnbp2 | NONE | MEF A&P | chr6 | 18464325 | 18466825 |
| 75196 | 4930532L20Rik | NONE | MEF A&P | chr6 | 18814318 | 18816818 |
| 386611 | Rnf133 | NONE | MEF A&P | chr6 | 23599807 | 23602307 |
| 387347 | Tas2r118 | NONE | MEF A&P | chr6 | 23919562 | 23922062 |
| 55961 | Slc13a1 | NONE | MEF A&P | chr6 | 24117594 | 24120094 |
| 74468 | Hyal5 | NONE | MEF A&P | chr6 | 24806006 | 24808506 |
| 14763 | Gpr37 | NONE | MEF A&P | chr6 | 25640239 | 25642739 |
| 14823 | Grm8 | NONE | MEF A&P | chr6 | 28075705 | 28078205 |
| 436523 | LOC436523 | NONE | MEF A&P | chr6 | 41319995 | 41322495 |
| 114228 | Prss1 | NONE | MEF A&P | chr6 | 41386549 | 41389049 |
| 18716 | Pip | NONE | MEF A&P | chr6 | 41775160 | 41777660 |
| 84543 | Sval2 | NONE | MEF A&P | chr6 | 41787945 | 41790445 |
| 71578 | Sval1 | NONE | MEF A&P | chr6 | 41879234 | 41881734 |
| 353148 | Tas2r139 | NONE | MEF A&P | chr6 | 42068542 | 42071042 |
| 258016 | Olfr453 | NKLTAG A | MEF A&P | chr6 | 42671645 | 42674145 |
| 258988 | Olfr38 | NONE | MEF A&P | chr6 | 42689660 | 42692160 |
| 258207 | Olfr452 | NONE | MEF A&P | chr6 | 42717647 | 42720147 |
| 258437 | Olfr450 | NONE | MEF A&P | chr6 | 42745079 | 42747579 |
| 259067 | Olfr449 | NONE | MEF A&P | chr6 | 42765489 | 42767989 |
| 258270 | Olfr448 | NONE | MEF A&P | chr6 | 42824059 | 42826559 |
| 258990 | Olfr447 | NONE | MEF A&P | chr6 | 42839131 | 42841631 |
| 258292 | Olfr446 | NONE | MEF A&P | chr6 | 42854839 | 42857339 |
| 258650 | Olfr444 | NONE | MEF A&P | chr6 | 42883106 | 42885606 |
| 258649 | Olfr441 | NKLTAG A | MEF A&P | chr6 | 43043350 | 43045850 |
| 258648 | Olfr438 | NKLTAG A | MEF A&P | chr6 | 43080913 | 43083413 |
| 258366 | Olfr434 | NKLTAG A | MEF A&P | chr6 | 43144521 | 43147021 |
| 66797 | Cntnap2 | NONE | MEF A&P | chr6 | 44987694 | 44990194 |
| 231932 | Gimap7 | NONE | MEF A&P | chr6 | 48646206 | 48648706 |
| 76507 | Abp1 | NONE | MEF A&P | chr6 | 48822839 | 48825339 |
| 109648 | Npy | NONE | MEF A&P | chr6 | 49750314 | 49752814 |
| 11922 | Neurod6 | NONE | MEF A&P | chr6 | 55610147 | 55612647 |
| 171194 | V1rc21 | NONE | MEF A&P | chr6 | 56884090 | 56886590 |
| 171192 | V1rc19 | NONE | MEF A&P | chr6 | 56912919 | 56915419 |
| 171193 | V1rc20 | NONE | MEF A&P | chr6 | 56929932 | 56932432 |
| 171205 | V1rc32 | NONE | MEF A&P | chr6 | 56959941 | 56962441 |
| 171203 | V1rc30 | NONE | MEF A&P | chr6 | 56998519 | 57001019 |
| 113858 | V1rc1 | NONE | MEF A&P | chr6 | 57041002 | 57043502 |
| 113860 | V1rc3 | NONE | MEF A&P | chr6 | 57064930 | 57067430 |
| 113861 | V1rc4 | NONE | MEF A&P | chr6 | 57116084 | 57118584 |
| 113864 | V1rc7 | NONE | MEF A&P | chr6 | 57161016 | 57163516 |
| 113863 | V1rc6 | NONE | MEF A&P | chr6 | 57185726 | 57188226 |
| 171202 | V1rc29 | NONE | MEF A&P | chr6 | 57252713 | 57255213 |
| 171189 | V1rc16 | NONE | MEF A&P | chr6 | 57290456 | 57292956 |
| 171199 | V1rc26 | NONE | MEF A&P | chr6 | 57319645 | 57322145 |
| 171200 | V1rc27 | NONE | MEF A&P | chr6 | 57332041 | 57334541 |
| 171196 | V1rc23 | NONE | MEF A&P | chr6 | 57830068 | 57832568 |
| 171191 | V1rc18 | NONE | MEF A&P | chr6 | 57885609 | 57888109 |
| 113865 | V1rc8 | NONE | MEF A&P | chr6 | 57908380 | 57910880 |
| 171190 | V1rc17 | NONE | MEF A&P | chr6 | 57938280 | 57940780 |
| 171206 | V1rc33 | NONE | MEF A&P | chr6 | 58145095 | 58147595 |
| 171198 | V1rc25 | NONE | MEF A&P | chr6 | 58192751 | 58195251 |
| 113859 | V1rc2 | NONE | MEF A&P | chr6 | 58234874 | 58237374 |
| 171195 | V1rc22 | NONE | MEF A&P | chr6 | 58364923 | 58367423 |
| 26357 | Abcg2 | NKLTAG A | MEF A&P | chr6 | 58524249 | 58526749 |
| 171188 | V1rc15 | NONE | MEF A&P | chr6 | 66488786 | 66491286 |
| 171185 | V1rc12 | NONE | MEF A&P | chr6 | 66608762 | 66611262 |
| 171184 | V1rc11 | NONE | MEF A&P | chr6 | 66645967 | 66648467 |
| 171183 | V1rc10 | NONE | MEF A&P | chr6 | 66658969 | 66661469 |
| 171186 | V1rc13 | NONE | MEF A&P | chr6 | 66706208 | 66708708 |
| 546213 | Gm1420\|N/A | NKLTAG A | MEF A&P | chr6 | 69578178 | 69580678 |
| 66740 | 4931417E11Rik | NKLTAG A | MEF A&P | chr6 | 73398676 | 73401176 |
| 30053 | Reg3d\|Reg3a | NONE | MEF A&P | chr6 | 78307872 | 78310717 |
| 19693 | Reg2 | NONE | MEF A&P | chr6 | 78332663 | 78335163 |
| 19692 | Reg1 | NONE | MEF A&P | chr6 | 78353491 | 78355991 |
| 19695 | Reg3g | NONE | MEF A&P | chr6 | 78397883 | 78400383 |
| 243499 | Lrrtm4 | NONE | MEF A&P | chr6 | 79946556 | 79949056 |
| 93674 | Cml3 | NKLTAG A | MEF A&P | chr6 | 85727634 | 85730134 |
| 93674 | Cml3 | NONE | MEF A&P | chr6 | 85730902 | 85733402 |
| 93673 | Cml2 | NONE | MEF A&P | chr6 | 85834292 | 85836792 |
| 320701 | C130034I18Rik | NONE | MEF A&P | chr6 | 97025374 | 97027874 |
| 232288 | Frmd4b | NONE | MEF A&P | chr6 | 97582544 | 97585044 |
| 207157 | 0610005I04 | NONE | MEF A&P | chr6 | 101738346 | 101740846 |
| 18488 | Cntn3 | NONE | MEF A&P | chr6 | 102430265 | 102432765 |
| 269784 | Cntn4 | NONE | MEF A&P | chr6 | 105641548 | 105644048 |
| 12391 | Cav3 | NKLTAG A | MEF A&P | chr6 | 112423277 | 112425777 |
| 11689 | Alox5 | NONE | MEF A&P | chr6 | 116426297 | 116428797 |
| 258019 | Olfr212 | NONE | MEF A&P | chr6 | 116470134 | 116472634 |
| 258020 | Olfr213 | NONE | MEF A&P | chr6 | 116504073 | 116506573 |
| 74043 | Pex26 | NONE | MEF A&P | chr6 | 121147285 | 121149785 |
| 243621 | Iqsec3 | NONE | MEF A&P | chr6 | 121438797 | 121441297 |
| 232345 | A2m | NONE | MEF A&P | chr6 | 121591573 | 121594073 |
| 17836 | Mug1 | NONE | MEF A&P | chr6 | 121802159 | 121804659 |
| 381806 | LOC381806 | NONE | MEF A&P | chr6 | 122100246 | 122102746 |
| 269799 | Clec4a1 | NONE | MEF A&P | chr6 | 122885476 | 122887976 |
| 73149 | Clec4a3 | NONE | MEF A&P | chr6 | 122916451 | 122918951 |
| 474145 | Dcir2 | NONE | MEF A&P | chr6 | 122953985 | 122956485 |
| 69810 | Clec4b | NONE | MEF A&P | chr6 | 123013624 | 123016124 |
| 26888 | Clec4a2 | NONE | MEF A&P | chr6 | 123087010 | 123089510 |
| 381809 | F830043G12Rik | NONE | MEF A&P | chr6 | 123136641 | 123139141 |
| 56620 | Clec4n | NONE | MEF A&P | chr6 | 123193492 | 123195992 |
| 17474 | Clec4d | NONE | MEF A&P | chr6 | 123225735 | 123228235 |
| 56619 | Clec4e | NONE | MEF A&P | chr6 | 123254990 | 123257490 |
| 56552 | V2r1b | NONE | MEF A&P | chr6 | 123988376 | 123990876 |
| 232400 | BC048546 | NONE | MEF A&P | chr6 | 128546725 | 128549225 |
| 667622 | LOC667622\|Clec2h | NONE | MEF A&P | chr6 | 128626004 | 128628504 |
| 17059 | Klrb1c | NONE | MEF A&P | chr6 | 128753699 | 128756199 |
| 387355 | Tas2r130 | NONE | MEF A&P | chr6 | 131596023 | 131598523 |
| 387342 | Tas2r107 | NKLTAG A | MEF A&P | chr6 | 131625277 | 131627777 |
| 387341 | Tas2r106 | NONE | MEF A&P | chr6 | 131644079 | 131646579 |
| 387340 | Tas2r104\|Tas2r105\|Tas2r114 | NONE | MEF A&P | chr6 | 131650937 | 131655156 |
| 387346 | Tas2r114 | NONE | MEF A&P | chr6 | 131655256 | 131657756 |
| 114600 | MP4 | NONE | MEF A&P | chr6 | 132082883 | 132085383 |
| 381833 | Prb1 | NONE | MEF A&P | chr6 | 132175714 | 132178214 |
| 381832 | BC061494\|Prpmp5 | NONE | MEF A&P | chr6 | 132279930 | 132282436 |
| 19131 | Prh1 | NONE | MEF A&P | chr6 | 132533534 | 132536034 |
| 83380 | Prp2 | NONE | MEF A&P | chr6 | 132559638 | 132562138 |
| 387348 | Tas2r120 | NONE | MEF A&P | chr6 | 132620649 | 132623149 |
| 353325 | Tas2r115 | NONE | MEF A&P | chr6 | 132703179 | 132705679 |
| 387339 | Tas2r102 | NONE | MEF A&P | chr6 | 132725823 | 132728323 |
| 353165 | Tas2r136 | NONE | MEF A&P | chr6 | 132743355 | 132745855 |
| 353166 | Tas2r117 | NONE | MEF A&P | chr6 | 132766593 | 132769093 |
| 112408 | Tas2r116 | NONE | MEF A&P | chr6 | 132819130 | 132821630 |
| 387344 | Tas2r110 | NONE | MEF A&P | chr6 | 132831700 | 132834200 |
| 387345 | Tas2r113 | NONE | MEF A&P | chr6 | 132856703 | 132859203 |
| 387352 | Tas2r125 | NONE | MEF A&P | chr6 | 132873343 | 132875843 |
| 387354 | Tas2r129 | NONE | MEF A&P | chr6 | 132914794 | 132917294 |
| 387356 | Tas2r131 | NONE | MEF A&P | chr6 | 132923037 | 132925537 |
| 387343 | Tas2r109 | NONE | MEF A&P | chr6 | 132946158 | 132948658 |
| 387616 | Tas2r140 | NONE | MEF A&P | chr6 | 133020986 | 133023486 |
| 277898 | 9830102E05Rik | NKLTAG A | MEF A&P | chr6 | 138036894 | 138039394 |
| 109593 | Lmo3 | NKLTAG A | MEF A&P | chr6 | 138545664 | 138548164 |
| 50540 | Igbp1b | NONE | MEF A&P | chr6 | 138622140 | 138624640 |
| 28248 | Slco1a1 | NONE | MEF A&P | chr6 | 141908700 | 141911200 |
| 321015 | 5330439B14Rik | NONE | MEF A&P | chr6 | 142569816 | 142572316 |
| 330450 | Mlstd1 | NONE | MEF A&P | chr6 | 148002585 | 148005085 |
| 18733 | Lilrb3 | NONE | MEF A&P | chr7 | 3322969 | 3325469 |
| 18722 | Pira1 | NONE | MEF A&P | chr7 | 3342457 | 3344957 |
| 18733 | Lilrb3\|Pira2 | NONE | MEF A&P | chr7 | 3447638 | 3450138 |
| 18729 | Pira6\|Pira3 | NONE | MEF A&P | chr7 | 3518062 | 3520572 |
| 664887 | LOC664887\|Pira6 | NONE | MEF A&P | chr7 | 3875275 | 3877775 |
| 17086 | N/A\|Ncr1 | NONE | MEF A&P | chr7 | 3938810 | 3941310 |
| 81014 | V1rd4\|LOC665163 | NONE | MEF A&P | chr7 | 5015732 | 5018232 |
| 81016 | V1rd2\|V1rd8 | NONE | MEF A&P | chr7 | 5275690 | 5278908 |
| 404285 | V1rd11 | NONE | MEF A&P | chr7 | 5487129 | 5489629 |
| 81013 | V1rd6 | NONE | MEF A&P | chr7 | 5613597 | 5616097 |
| 258917 | Olfr1336 | NONE | MEF A&P | chr7 | 6062595 | 6065095 |
| 258918 | Olfr1346 | NONE | MEF A&P | chr7 | 6076196 | 6078696 |
| 18349 | Olfr5 | NONE | MEF A&P | chr7 | 6084739 | 6087239 |
| 258383 | Olfr1347 | NONE | MEF A&P | chr7 | 6092457 | 6094957 |
| 258915 | Olfr1348 | NONE | MEF A&P | chr7 | 6105809 | 6108309 |
| 269862 | Olfr1349 | NKLTAG A | MEF A&P | chr7 | 6119012 | 6121512 |
| 258384 | Olfr1350 | NONE | MEF A&P | chr7 | 6172078 | 6174578 |
| 20871 | Aurkc | NKLTAG A | MEF A&P | chr7 | 6597385 | 6599885 |
| 22306 | V2r15 | NKLTAG A | MEF A&P | chr7 | 6941117 | 6943617 |
| 22305 | V2r14 | NONE | MEF A&P | chr7 | 7597143 | 7599643 |
| 381838 | Gm1961 | NKLTAG A | MEF A&P | chr7 | 8199682 | 8202182 |
| 252904 | V1re9 | NONE | MEF A&P | chr7 | 9455880 | 9458380 |
| 171262 | V1rl1 | NONE | MEF A&P | chr7 | 9506479 | 9508979 |
| 210045 | Nalp4b | NONE | MEF A&P | chr7 | 9560685 | 9563185 |
| 252910 | V1re13 | NONE | MEF A&P | chr7 | 9623931 | 9626431 |
| 245109 | Gm397 | NONE | MEF A&P | chr7 | 9878637 | 9881137 |
| 252905 | V1rg1 | NONE | MEF A&P | chr7 | 10575883 | 10578383 |
| 628898 | LOC628898\|V1rg5 | NONE | MEF A&P | chr7 | 10750638 | 10753138 |
| 171241 | V1rg6 | NONE | MEF A&P | chr7 | 10784206 | 10786706 |
| 171239 | V1rg4 | NONE | MEF A&P | chr7 | 10836649 | 10839149 |
| 171242 | V1rg7 | NONE | MEF A&P | chr7 | 11056327 | 11058827 |
| 171238 | V1rg3 | NONE | MEF A&P | chr7 | 11096828 | 11099328 |
| 171244 | V1rg9 | NONE | MEF A&P | chr7 | 11166043 | 11168543 |
| 171243 | V1rg8 | NONE | MEF A&P | chr7 | 11227492 | 11229992 |
| 171267 | V1rg11 | NONE | MEF A&P | chr7 | 11268128 | 11270628 |
| 252909 | V1rj3 | NONE | MEF A&P | chr7 | 11984894 | 11987394 |
| 171261 | V1rk1 | NONE | MEF A&P | chr7 | 12032037 | 12034537 |
| 171260 | V1rj2 | NONE | MEF A&P | chr7 | 12117517 | 12120017 |
| 20865 | Sult2a2 | NONE | MEF A&P | chr7 | 12631718 | 12634218 |
| 76971 | 2810007J24Rik | NONE | MEF A&P | chr7 | 13346266 | 13348766 |
| 627280 | B430211C08Rik\|LOC666335 | NONE | MEF A&P | chr7 | 13522746 | 13525246 |
| 194588 | MGC117731 | NONE | MEF A&P | chr7 | 13557887 | 13560387 |
| 246792 | Obox2 | NKLTAG A | MEF A&P | chr7 | 14295024 | 14297524 |
| 71468 | Obox1 | NKLTAG A | MEF A&P | chr7 | 14493054 | 14495554 |
| 252829 | Obox5 | NONE | MEF A&P | chr7 | 14914591 | 14917091 |
| 67084 | Ceacam14 | NONE | MEF A&P | chr7 | 16969203 | 16971703 |
| 381852 | LOC381852 | NONE | MEF A&P | chr7 | 17028288 | 17030788 |
| 66996 | Ceacam11 | NONE | MEF A&P | chr7 | 17128645 | 17131145 |
| 69785 | Ceacam13 | NONE | MEF A&P | chr7 | 17166410 | 17168919 |
| 67315 | Ceacam12 | NONE | MEF A&P | chr7 | 17222448 | 17224948 |
| 232925 | Gm580 | NONE | MEF A&P | chr7 | 17333015 | 17335515 |
| 26438 | Psg18 | NONE | MEF A&P | chr7 | 17512897 | 17515397 |
| 114871 | Psg28 | NONE | MEF A&P | chr7 | 17590063 | 17592563 |
| 56868 | Psg23 | NONE | MEF A&P | chr7 | 17774523 | 17777023 |
| 72242 | Psg21 | NONE | MEF A&P | chr7 | 17814744 | 17817244 |
| 26439 | Psg19 | NONE | MEF A&P | chr7 | 17956532 | 17959032 |
| 404290 | V1rd21 | NONE | MEF A&P | chr7 | 19297110 | 19299610 |
| 81011 | V1rd14 | NKLTAG A | MEF A&P | chr7 | 22118990 | 22121490 |
| 381936 | LOC381936 | NKLTAG A | MEF A&P | chr7 | 22427970 | 22430470 |
| 446099 | Nalp4e | NONE | MEF A&P | chr7 | 23008038 | 23010538 |
| 81012 | V1rd7 | NONE | MEF A&P | chr7 | 23338737 | 23341237 |
| 81010 | V1rd9 | NONE | MEF A&P | chr7 | 23365075 | 23367575 |
| 404291 | V1rd22 | NONE | MEF A&P | chr7 | 23460670 | 23463170 |
| 384572 | V1rd12 | NONE | MEF A&P | chr7 | 23574709 | 23577209 |
| 232959 | V1rd13 | NONE | MEF A&P | chr7 | 23600288 | 23602788 |
| 404286 | V1rd17 | NONE | MEF A&P | chr7 | 23635145 | 23637645 |
| 232962 | V1rd16 | NONE | MEF A&P | chr7 | 23659173 | 23661673 |
| 404289 | V1rd20 | NONE | MEF A&P | chr7 | 23690871 | 23693371 |
| 404288 | V1rd19\|V1rd18 | NONE | MEF A&P | chr7 | 23709870 | 23712370 |
| 209824 | V1rd15 | NONE | MEF A&P | chr7 | 23761533 | 23764033 |
| 13094 | Cyp2b9 | NONE | MEF A&P | chr7 | 25880168 | 25882668 |
| 13086 | Cyp2a4 | NONE | MEF A&P | chr7 | 26013951 | 26016451 |
| 330490 | Nalp9c | NONE | MEF A&P | chr7 | 26102498 | 26104998 |
| 243880 | Nalp4a | NONE | MEF A&P | chr7 | 26141872 | 26144372 |
| 171265 | V1re12 | NONE | MEF A&P | chr7 | 26320338 | 26322838 |
| 13087 | Cyp2a5\|Cyp2a4 | NONE | MEF A&P | chr7 | 26542100 | 26544600 |
| 442797 | 6330444E15Rik | NONE | MEF A&P | chr7 | 29285634 | 29288134 |
| 76718 | 1700067C01Rik\|LOC668241 | NONE | MEF A&P | chr7 | 29435256 | 29437756 |
| 57426 | Apbh | NONE | MEF A&P | chr7 | 31000076 | 31002576 |
| 381970 | Abpe | NONE | MEF A&P | chr7 | 31009520 | 31012020 |
| 494519 | Abpd | NONE | MEF A&P | chr7 | 33074582 | 33077082 |
| 233090 | Abpz | NONE | MEF A&P | chr7 | 33447555 | 33450055 |
| 110187 | Abpg | NONE | MEF A&P | chr7 | 33653231 | 33655741 |
| 66161 | Pop4 | NONE | MEF A&P | chr7 | 37979936 | 37982436 |
| 434171 | LOC434171 | NONE | MEF A&P | chr7 | 38236781 | 38239281 |
| 330513 | 4921507A12 | NKLTAG A | MEF A&P | chr7 | 39186864 | 39189364 |
| 243944 | 4930433I11Rik | NONE | MEF A&P | chr7 | 40758707 | 40761207 |
| 233164 | 4930549O06 | NONE | MEF A&P | chr7 | 40803871 | 40806371 |
| 434172 | LOC434172 | NONE | MEF A&P | chr7 | 41055479 | 41057979 |
| 75835 | 1700108E19Rik | NKLTAG A | MEF A&P | chr7 | 41439131 | 41441631 |
| 434179 | LOC434179 | NONE | MEF A&P | chr7 | 42465371 | 42467871 |
| 435970 | LOC435970 | NKLTAG A | MEF A&P | chr7 | 43043518 | 43046018 |
| 76999 | 1700127D06Rik | NKLTAG A | MEF A&P | chr7 | 43811053 | 43813553 |
| 13648 | Klk1b9 | NONE | MEF A&P | chr7 | 43842102 | 43844602 |
| 16613 | Klk1b11 | NONE | MEF A&P | chr7 | 43861920 | 43864420 |
| 16618 | Klk1b26\|Egfbp2 | NONE | MEF A&P | chr7 | 43878719 | 43881238 |
| 16616 | Klk1b21 | NKLTAG A | MEF A&P | chr7 | 43968331 | 43970831 |
| 16615 | Klk1b16 | NONE | MEF A&P | chr7 | 44002808 | 44005308 |
| 16617 | Klk1b24 | NONE | MEF A&P | chr7 | 44054304 | 44056804 |
| 18050 | Klk1b3 | NONE | MEF A&P | chr7 | 44064271 | 44066771 |
| 18048 | Klk1b4 | NONE | MEF A&P | chr7 | 44073480 | 44075980 |
| 16622 | Klk1b5 | NONE | MEF A&P | chr7 | 44082500 | 44085000 |
| 638411 | LOC638411\|Klk1 | NONE | MEF A&P | chr7 | 44091478 | 44093978 |
| 233221 | Mrgpra1 | NONE | MEF A&P | chr7 | 47221762 | 47224262 |
| 235712 | Mrgpra2 | NONE | MEF A&P | chr7 | 47357078 | 47359578 |
| 404235 | Mrgpra5 | NONE | MEF A&P | chr7 | 47423757 | 47426257 |
| 233222 | Mrgpra3 | NONE | MEF A&P | chr7 | 47468893 | 47471393 |
| 235854 | Mrgpra4\|N/A | NONE | MEF A&P | chr7 | 47849818 | 47852318 |
| 404242 | Mrgprx1 | NONE | MEF A&P | chr7 | 47895123 | 47897623 |
| 233230 | Mrgprb4 | NONE | MEF A&P | chr7 | 48066700 | 48069200 |
| 404240 | Mrgprb8 | NONE | MEF A&P | chr7 | 48254604 | 48257104 |
| 233231 | Mrgprb1 | NONE | MEF A&P | chr7 | 48323817 | 48326317 |
| 243978 | Mrgprx2 | NONE | MEF A&P | chr7 | 48366722 | 48369222 |
| 243979 | Mrgprb2 | NONE | MEF A&P | chr7 | 48425608 | 48428108 |
| 404238 | Mrgprb3 | NONE | MEF A&P | chr7 | 48511323 | 48513823 |
| 243996 | 4933405O20Rik | NONE | MEF A&P | chr7 | 50465208 | 50467708 |
| 20646 | Snrpn\|Snurf | NONE | MEF A&P | chr7 | 59883554 | 59886074 |
| 17984 | Ndn | NONE | MEF A&P | chr7 | 62225297 | 62227797 |
| 22652 | Mkrn3 | NONE | MEF A&P | chr7 | 62298589 | 62301089 |
| 258410 | Olfr291 | NONE | MEF A&P | chr7 | 84730549 | 84733049 |
| 258411 | Olfr290 | NONE | MEF A&P | chr7 | 84789965 | 84792465 |
| 233437 | F830104D24Rik | NONE | MEF A&P | chr7 | 84883412 | 84885912 |
| 258196 | Olfr309 | NONE | MEF A&P | chr7 | 86182372 | 86184872 |
| 258614 | Olfr308 | NONE | MEF A&P | chr7 | 86197211 | 86199711 |
| 258610 | Olfr307 | NONE | MEF A&P | chr7 | 86211655 | 86214155 |
| 258609 | Olfr305 | NONE | MEF A&P | chr7 | 86239596 | 86242096 |
| 258089 | Olfr304 | NONE | MEF A&P | chr7 | 86261919 | 86264419 |
| 257958 | Olfr301 | NONE | MEF A&P | chr7 | 86286124 | 86288624 |
| 257929 | Olfr299 | NONE | MEF A&P | chr7 | 86339173 | 86341673 |
| 257905 | Olfr298 | NONE | MEF A&P | chr7 | 86364810 | 86367310 |
| 258611 | Olfr297 | NONE | MEF A&P | chr7 | 86400519 | 86403019 |
| 258850 | Olfr295 | NONE | MEF A&P | chr7 | 86459037 | 86461537 |
| 257904 | Olfr294 | NONE | MEF A&P | chr7 | 86491904 | 86494404 |
| 257906 | Olfr293 | NONE | MEF A&P | chr7 | 86537424 | 86539924 |
| 258613 | Olfr292 | NONE | MEF A&P | chr7 | 86568218 | 86570718 |
| 258750 | Olfr551 | NONE | MEF A&P | chr7 | 102462062 | 102464562 |
| 259106 | Olfr552 | NONE | MEF A&P | chr7 | 102476176 | 102478676 |
| 18332 | Olfr33 | NONE | MEF A&P | chr7 | 102587732 | 102590232 |
| 259116 | Olfr559 | NONE | MEF A&P | chr7 | 102597809 | 102600309 |
| 259117 | Olfr560 | NONE | MEF A&P | chr7 | 102627248 | 102629748 |
| 170639 | Olfr78 | NONE | MEF A&P | chr7 | 102632716 | 102635216 |
| 259096 | Olfr561 | NONE | MEF A&P | chr7 | 102646346 | 102648846 |
| 258356 | Olfr564 | NONE | MEF A&P | chr7 | 102675300 | 102677800 |
| 258168 | Olfr566 | NONE | MEF A&P | chr7 | 102730592 | 102733092 |
| 259092 | Olfr569 | NKLTAG A | MEF A&P | chr7 | 102761472 | 102763972 |
| 259114 | Olfr570 | NONE | MEF A&P | chr7 | 102772189 | 102774689 |
| 259089 | Olfr571 | NONE | MEF A&P | chr7 | 102783158 | 102785658 |
| 258357 | Olfr574 | NONE | MEF A&P | chr7 | 102820287 | 102822787 |
| 259118 | Olfr575 | NONE | MEF A&P | chr7 | 102828941 | 102831441 |
| 258248 | Olfr576 | NONE | MEF A&P | chr7 | 102836922 | 102839422 |
| 259113 | Olfr577 | NONE | MEF A&P | chr7 | 102847311 | 102849811 |
| 259119 | Olfr578 | NONE | MEF A&P | chr7 | 102858483 | 102860983 |
| 259055 | Olfr582 | NONE | MEF A&P | chr7 | 102913301 | 102915801 |
| 258752 | Olfr583 | NONE | MEF A&P | chr7 | 102923120 | 102925620 |
| 259056 | Olfr584 | NKLTAG A | MEF A&P | chr7 | 102957340 | 102959840 |
| 259091 | Olfr585 | NONE | MEF A&P | chr7 | 102969563 | 102972063 |
| 259115 | Olfr586 | NONE | MEF A&P | chr7 | 102996103 | 102998603 |
| 259054 | Olfr589 | NKLTAG A | MEF A&P | chr7 | 103029066 | 103031566 |
| 258139 | Olfr591 | NONE | MEF A&P | chr7 | 103046956 | 103049456 |
| 258378 | Olfr593 | NONE | MEF A&P | chr7 | 103083682 | 103086182 |
| 258246 | Olfr594 | NONE | MEF A&P | chr7 | 103091540 | 103094040 |
| 436004 | Usp17\|Dub2a | NKLTAG A | MEF A&P | chr7 | 103125826 | 103130722 |
| 258135 | Olfr597 | NONE | MEF A&P | chr7 | 103192233 | 103194733 |
| 257975 | Olfr598 | NONE | MEF A&P | chr7 | 103200308 | 103202808 |
| 258726 | Olfr599 | NONE | MEF A&P | chr7 | 103209876 | 103212376 |
| 259048 | Olfr600 | NONE | MEF A&P | chr7 | 103220247 | 103222747 |
| 259073 | Olfr604 | NONE | MEF A&P | chr7 | 103257321 | 103259821 |
| 13532 | Dub2 | NKLTAG A | MEF A&P | chr7 | 103288516 | 103291016 |
| 258156 | Olfr605 | NONE | MEF A&P | chr7 | 103316442 | 103318942 |
| 259098 | Olfr606 | NKLTAG A | MEF A&P | chr7 | 103323159 | 103325659 |
| 258722 | Olfr611 | NONE | MEF A&P | chr7 | 103391703 | 103394203 |
| 259084 | Olfr615 | NONE | MEF A&P | chr7 | 103432299 | 103434799 |
| 258838 | Olfr617 | NONE | MEF A&P | chr7 | 103455844 | 103458344 |
| 259080 | Olfr619 | NONE | MEF A&P | chr7 | 103475476 | 103477976 |
| 258808 | Olfr620 | NONE | MEF A&P | chr7 | 103485672 | 103488172 |
| 259087 | Olfr622 | NONE | MEF A&P | chr7 | 103513459 | 103515959 |
| 259126 | Olfr623 | NONE | MEF A&P | chr7 | 103534569 | 103537069 |
| 258818 | Olfr629 | NONE | MEF A&P | chr7 | 103614559 | 103617059 |
| 259123 | Olfr632 | NONE | MEF A&P | chr7 | 103809202 | 103811702 |
| 259122 | Olfr635 | NKLTAG A | MEF A&P | chr7 | 103850996 | 103853496 |
| 259124 | Olfr638 | NONE | MEF A&P | chr7 | 103875079 | 103877579 |
| 259088 | Olfr639 | NONE | MEF A&P | chr7 | 103886021 | 103888521 |
| 258819 | Olfr640 | NONE | MEF A&P | chr7 | 103895637 | 103898137 |
| 258326 | Olfr642 | NKLTAG A | MEF A&P | chr7 | 103923673 | 103926173 |
| 259081 | Olfr643 | NONE | MEF A&P | chr7 | 103932921 | 103935421 |
| 259125 | Olfr644 | NONE | MEF A&P | chr7 | 103942350 | 103944850 |
| 258247 | Olfr645 | NONE | MEF A&P | chr7 | 103958399 | 103960899 |
| 259058 | Olfr646 | NONE | MEF A&P | chr7 | 103978101 | 103980601 |
| 244178 | Ubqln3 | NONE | MEF A&P | chr7 | 104016593 | 104019093 |
| 258755 | Olfr672 | NONE | MEF A&P | chr7 | 104870223 | 104872723 |
| 258753 | Olfr678 | NONE | MEF A&P | chr7 | 104941289 | 104943789 |
| 259046 | Olfr679 | NONE | MEF A&P | chr7 | 104957538 | 104960038 |
| 404318 | Olfr681 | NKLTAG A | MEF A&P | chr7 | 104993279 | 104995779 |
| 244187 | Olfr684 | NONE | MEF A&P | chr7 | 105031001 | 105033501 |
| 258160 | Olfr685 | NONE | MEF A&P | chr7 | 105054632 | 105057132 |
| 259072 | Olfr686 | NKLTAG A | MEF A&P | chr7 | 105077662 | 105080162 |
| 258591 | Olfr695 | NONE | MEF A&P | chr7 | 106507828 | 106510328 |
| 258592 | Olfr697 | NONE | MEF A&P | chr7 | 106535081 | 106537581 |
| 258590 | Olfr702 | NONE | MEF A&P | chr7 | 106617673 | 106620173 |
| 257902 | Olfr704 | NONE | MEF A&P | chr7 | 106656630 | 106659130 |
| 258350 | Olfr706 | NONE | MEF A&P | chr7 | 106679964 | 106682464 |
| 258173 | Olfr708 | NKLTAG A | MEF A&P | chr7 | 106705848 | 106708348 |
| 233670 | Olfr6 | NONE | MEF A&P | chr7 | 106750083 | 106752583 |
| 18317 | Olfr2 | NKLTAG A | MEF A&P | chr7 | 106795007 | 106797507 |
| 259036 | Olfr713 | NONE | MEF A&P | chr7 | 106827784 | 106830284 |
| 258776 | Olfr715 | NONE | MEF A&P | chr7 | 106920068 | 106922568 |
| 258597 | Olfr716 | NONE | MEF A&P | chr7 | 106936494 | 106938994 |
| 76858 | Nalp14 | NONE | MEF A&P | chr7 | 106956166 | 106958666 |
| 434225 | LOC434225 | NONE | MEF A&P | chr7 | 107073958 | 107076458 |
| 258418 | Olfr469 | NONE | MEF A&P | chr7 | 107614144 | 107616644 |
| 258417 | Olfr470 | NONE | MEF A&P | chr7 | 107636408 | 107638908 |
| 258770 | Olfr472 | NONE | MEF A&P | chr7 | 107691895 | 107694395 |
| 258771 | Olfr473 | NONE | MEF A&P | chr7 | 107722698 | 107725198 |
| 258488 | Olfr474 | NONE | MEF A&P | chr7 | 107743819 | 107746319 |
| 258926 | Olfr476 | NONE | MEF A&P | chr7 | 107756575 | 107759075 |
| 258928 | Olfr477 | NONE | MEF A&P | chr7 | 107779543 | 107782043 |
| 257891 | Olfr479 | NONE | MEF A&P | chr7 | 107844160 | 107846660 |
| 258728 | Olfr482 | NONE | MEF A&P | chr7 | 107886245 | 107888745 |
| 258492 | Olfr484 | NONE | MEF A&P | chr7 | 107915938 | 107918438 |
| 258041 | Olfr485 | NONE | MEF A&P | chr7 | 107950548 | 107953048 |
| 258489 | Olfr486 | NONE | MEF A&P | chr7 | 107963419 | 107965919 |
| 258042 | Olfr487 | NONE | MEF A&P | chr7 | 108003204 | 108005704 |
| 258727 | Olfr488 | NONE | MEF A&P | chr7 | 108046813 | 108049313 |
| 258491 | Olfr490 | NONE | MEF A&P | chr7 | 108077801 | 108080301 |
| 258490 | Olfr492 | NONE | MEF A&P | chr7 | 108114351 | 108116851 |
| 258307 | Olfr493 | NONE | MEF A&P | chr7 | 108137656 | 108140156 |
| 258732 | Olfr494 | NONE | MEF A&P | chr7 | 108156668 | 108159168 |
| 258361 | Olfr495 | NONE | MEF A&P | chr7 | 108184298 | 108186798 |
| 258733 | Olfr497 | NONE | MEF A&P | chr7 | 108211749 | 108214249 |
| 258304 | Olfr498 | NONE | MEF A&P | chr7 | 108254502 | 108257002 |
| 258734 | Olfr502 | NONE | MEF A&P | chr7 | 108314625 | 108317125 |
| 258163 | Olfr504 | NONE | MEF A&P | chr7 | 108356470 | 108358970 |
| 258215 | Olfr506 | NONE | MEF A&P | chr7 | 108401485 | 108403985 |
| 258738 | Olfr507 | NONE | MEF A&P | chr7 | 108410990 | 108413490 |
| 258769 | Olfr508 | NONE | MEF A&P | chr7 | 108419170 | 108421670 |
| 258369 | Olfr509 | NONE | MEF A&P | chr7 | 108437251 | 108439751 |
| 258308 | Olfr510 | NONE | MEF A&P | chr7 | 108456594 | 108459094 |
| 258719 | Olfr512 | NONE | MEF A&P | chr7 | 108502531 | 108505031 |
| 258718 | Olfr513 | NONE | MEF A&P | chr7 | 108544034 | 108546534 |
| 258721 | Olfr514 | NONE | MEF A&P | chr7 | 108616674 | 108619174 |
| 258720 | Olfr516 | NONE | MEF A&P | chr7 | 108636685 | 108639185 |
| 258136 | Olfr517 | NONE | MEF A&P | chr7 | 108659829 | 108662329 |
| 277935 | Olfr519 | NONE | MEF A&P | chr7 | 108685082 | 108687582 |
| 320360 | Ric3 | NONE | MEF A&P | chr7 | 108874001 | 108876501 |
| 244209 | Cyp2r1 | NONE | MEF A&P | chr7 | 114353653 | 114356153 |
| 67133 | Gp2 | NONE | MEF A&P | chr7 | 119249921 | 119252421 |
| 272428 | C730027J19Rik\|N/A | NONE | MEF A&P | chr7 | 119315452 | 119317952 |
| 233799 | Acsm2 | NONE | MEF A&P | chr7 | 119350880 | 119353380 |
| 20216 | Acsm3 | NONE | MEF A&P | chr7 | 119550071 | 119552571 |
| 320631 | Abca15 | NKLTAG A | MEF A&P | chr7 | 120117832 | 120120332 |
| 54376 | Cacng3 | NKLTAG A | MEF A&P | chr7 | 122460921 | 122463421 |
| 71208 | 4933440M02Rik | NKLTAG A | MEF A&P | chr7 | 125140420 | 125142920 |
| 70571 | Tcerg1l | NONE | MEF A&P | chr7 | 138235573 | 138238073 |
| 258958 | Olfr525 | NONE | MEF A&P | chr7 | 140172020 | 140174520 |
| 18361 | Olfr60 | NONE | MEF A&P | chr7 | 140196807 | 140199307 |
| 258512 | Olfr530 | NONE | MEF A&P | chr7 | 140224428 | 140226928 |
| 258955 | Olfr531 | NKLTAG A | MEF A&P | chr7 | 140251864 | 140254364 |
| 259028 | Olfr532 | NONE | MEF A&P | chr7 | 140270591 | 140273091 |
| 258956 | Olfr535 | NONE | MEF A&P | chr7 | 140341959 | 140344459 |
| 258513 | Olfr536 | NONE | MEF A&P | chr7 | 140358138 | 140360638 |
| 258201 | Olfr538 | NONE | MEF A&P | chr7 | 140423474 | 140425974 |
| 18345 | Olfr46 | NONE | MEF A&P | chr7 | 140459487 | 140461987 |
| 18362 | Olfr61 | NONE | MEF A&P | chr7 | 140487022 | 140489522 |
| 258962 | Olfr53 | NONE | MEF A&P | chr7 | 140501300 | 140503800 |
| 258963 | Olfr539 | NONE | MEF A&P | chr7 | 140516608 | 140519108 |
| 18344 | Olfr45 | NONE | MEF A&P | chr7 | 140540226 | 140542726 |
| 258964 | Olfr541 | NONE | MEF A&P | chr7 | 140553572 | 140556072 |
| 170786 | Cd209a | NONE | MEF A&P | chr8 | 3748430 | 3750930 |
| 170779 | Cd209d | NONE | MEF A&P | chr8 | 3877999 | 3880499 |
| 69165 | Cd209b | NONE | MEF A&P | chr8 | 3926298 | 3928814 |
| 20494 | Slc10a2 | NONE | MEF A&P | chr8 | 5104704 | 5107204 |
| 244281 | N/A\|C230040D10Rik | NONE | MEF A&P | chr8 | 10151882 | 10154382 |
| 231201 | AF366264 | NONE | MEF A&P | chr8 | 13837856 | 13840356 |
| 353320 | Defb37 | NONE | MEF A&P | chr8 | 18990532 | 18993032 |
| 360214 | Defb39 | NONE | MEF A&P | chr8 | 19064287 | 19066787 |
| 77674 | Defb12 | NONE | MEF A&P | chr8 | 19114310 | 19116810 |
| 360211 | Defb34 | NONE | MEF A&P | chr8 | 19121728 | 19124228 |
| 546038 | Spag11c/h | NONE | MEF A&P | chr8 | 19138735 | 19141269 |
| 78128 | Spag11 | NONE | MEF A&P | chr8 | 19155863 | 19158363 |
| 244332 | Defb14 | NONE | MEF A&P | chr8 | 19192333 | 19194833 |
| 116746 | Defb6 | NONE | MEF A&P | chr8 | 19223454 | 19225954 |
| 574081 | Defb53 | NONE | MEF A&P | chr8 | 19237892 | 19240392 |
| 27358 | Defb3 | NONE | MEF A&P | chr8 | 19291340 | 19293840 |
| 503556 | LOC503556 | NONE | MEF A&P | chr8 | 20085819 | 20088319 |
| 574083 | Defb51 | NONE | MEF A&P | chr8 | 20112838 | 20115338 |
| 626415 | 4930467E23Rik | NKLTAG A | MEF A&P | chr8 | 20377085 | 20379585 |
| 436177 | LOC436177 | NKLTAG A | MEF A&P | chr8 | 20424615 | 20427115 |
| 381598 | 2610005L07Rik\|A430108E01Rik | NKLTAG A | MEF A&P | chr8 | 20438598 | 20441192 |
| 66298 | 2010016B13Rik | NONE | MEF A&P | chr8 | 22518295 | 22520795 |
| 13216 | Defa1\|Defcr23 | NONE | MEF A&P | chr8 | 22547819 | 22550332 |
| 382059 | Defcr22 | NONE | MEF A&P | chr8 | 22655028 | 22657528 |
| 13237 | Defcr3 | NONE | MEF A&P | chr8 | 22780194 | 22782694 |
| 13239 | Defcr5 | NONE | MEF A&P | chr8 | 22790190 | 22792690 |
| 68009 | Defcr20\|Defcr4 | NONE | MEF A&P | chr8 | 22972800 | 22975345 |
| 382000 | AY761184 | NONE | MEF A&P | chr8 | 23168692 | 23171192 |
| 13240 | Defcr6\|Defcr24 | NONE | MEF A&P | chr8 | 23198081 | 23200581 |
| 13214 | Defb1 | NONE | MEF A&P | chr8 | 23240143 | 23242643 |
| 387334 | Defb50 | NONE | MEF A&P | chr8 | 23287083 | 23289583 |
| 13215 | Defb2 | NONE | MEF A&P | chr8 | 23303470 | 23305970 |
| 246085 | Defb10 | NONE | MEF A&P | chr8 | 23322445 | 23324945 |
| 246082 | Defb15 | NONE | MEF A&P | chr8 | 23397756 | 23400256 |
| 246084 | Defb35 | NONE | MEF A&P | chr8 | 23401896 | 23404396 |
| 246083 | Defb13 | NONE | MEF A&P | chr8 | 23410335 | 23412835 |
| 67929 | Ccdc70 | NONE | MEF A&P | chr8 | 23434140 | 23436640 |
| 234129 | Tpte\|N/A | NONE | MEF A&P | chr8 | 23746985 | 23749485 |
| 11497 | Adam3 | NONE | MEF A&P | chr8 | 26190870 | 26193370 |
| 541463 | Tex24 | NONE | MEF A&P | chr8 | 28808329 | 28810829 |
| 70952 | 4921537P18Rik | NONE | MEF A&P | chr8 | 28911605 | 28914105 |
| 382003 | Gm1698 | NONE | MEF A&P | chr8 | 29068286 | 29070786 |
| 244431 | Sgcz | NONE | MEF A&P | chr8 | 39421074 | 39423574 |
| 384806 | LOC384806 | NONE | MEF A&P | chr8 | 42291671 | 42294171 |
| 384813 | LOC384813 | NONE | MEF A&P | chr8 | 45125502 | 45128002 |
| 252866 | Adam34 | NONE | MEF A&P | chr8 | 45163882 | 45166382 |
| 109821 | F11 | NONE | MEF A&P | chr8 | 46760348 | 46762848 |
| 234267 | Gpm6a | NONE | MEF A&P | chr8 | 56451540 | 56454040 |
| 244486 | Adam29 | NONE | MEF A&P | chr8 | 58798590 | 58801090 |
| 270049 | 4930431L04Rik\|BC030500 | NKLTAG A | MEF A&P | chr8 | 61801896 | 61805769 |
| 12725 | Clcn3\|B230317F23Rik | NKLTAG A | MEF A&P | chr8 | 63873578 | 63877442 |
| 234329 | Trim60\|Trim61 | NONE | MEF A&P | chr8 | 67947055 | 67949566 |
| 258532 | Olfr373\|Olfr372 | NKLTAG A | MEF A&P | chr8 | 74984670 | 74987170 |
| 258532 | Olfr373 | NKLTAG A | MEF A&P | chr8 | 75026750 | 75029250 |
| 12745 | Clgn | NONE | MEF A&P | chr8 | 86277995 | 86280495 |
| 330820 | 4933402J07Rik | NONE | MEF A&P | chr8 | 90452011 | 90454511 |
| 104158 | Ces3 | NONE | MEF A&P | chr8 | 96086407 | 96088907 |
| 67935 | Ces7 | NONE | MEF A&P | chr8 | 96424336 | 96426836 |
| 12564 | Cdh8\|A330008L17Rik | NONE | MEF A&P | chr8 | 102303782 | 102307586 |
| 234671 | Ces2 | NONE | MEF A&P | chr8 | 107734214 | 107736714 |
| 72361 | 2210023G05Rik | NONE | MEF A&P | chr8 | 107848868 | 107851368 |
| 13909 | LOC13909 | NONE | MEF A&P | chr8 | 107970898 | 107973398 |
| 56523 | Pmfbp1 | NONE | MEF A&P | chr8 | 112381155 | 112383655 |
| 12308 | Calb2 | NONE | MEF A&P | chr8 | 113056835 | 113059335 |
| 244653 | Hydin | NONE | MEF A&P | chr8 | 113154105 | 113156605 |
| 170571 | Cntnap4 | NONE | MEF A&P | chr8 | 115454019 | 115456519 |
| 16525 | Kcnk1 | NONE | MEF A&P | chr8 | 128879259 | 128881759 |
| 74703 | Ccdc7\|1700008F21Rik | NONE | MEF A&P | chr8 | 131951063 | 131953723 |
| 14802 | Gria4 | NONE | MEF A&P | chr9 | 4795642 | 4798142 |
| 17384 | Mmp10 | NKLTAG A | MEF A&P | chr9 | 7488824 | 7491324 |
| 22068 | Trpc6 | NONE | MEF A&P | chr9 | 8542710 | 8545210 |
| 18667 | Pgr | NONE | MEF A&P | chr9 | 8860302 | 8862802 |
| 234964 | Ccdc67 | NONE | MEF A&P | chr9 | 15362049 | 15364549 |
| 244701 | Mtnr1b | NKLTAG A | MEF A&P | chr9 | 15624353 | 15626853 |
| 72560 | Naalad2 | NONE | MEF A&P | chr9 | 18135830 | 18138330 |
| 18322 | Olfr24 | NKLTAG A | MEF A&P | chr9 | 18505535 | 18508035 |
| 258598 | Olfr828 | NONE | MEF A&P | chr9 | 18566194 | 18568694 |
| 259070 | Olfr829 | NONE | MEF A&P | chr9 | 18605028 | 18607528 |
| 258559 | Olfr830 | NONE | MEF A&P | chr9 | 18623730 | 18626230 |
| 258075 | Olfr832 | NONE | MEF A&P | chr9 | 18693051 | 18695551 |
| 258074 | Olfr834 | NONE | MEF A&P | chr9 | 18736391 | 18738891 |
| 258557 | Olfr836 | NONE | MEF A&P | chr9 | 18869358 | 18871858 |
| 258558 | Olfr837 | NONE | MEF A&P | chr9 | 18885396 | 18887896 |
| 258249 | Olfr845 | NONE | MEF A&P | chr9 | 19086863 | 19089363 |
| 258279 | Olfr846 | NONE | MEF A&P | chr9 | 19111255 | 19113755 |
| 258518 | Olfr847 | NONE | MEF A&P | chr9 | 19125781 | 19128281 |
| 258520 | Olfr849 | NONE | MEF A&P | chr9 | 19189316 | 19191816 |
| 258516 | Olfr850 | NONE | MEF A&P | chr9 | 19228150 | 19230650 |
| 258907 | Olfr851 | NONE | MEF A&P | chr9 | 19245151 | 19247651 |
| 258908 | Olfr853 | NONE | MEF A&P | chr9 | 19287830 | 19290330 |
| 258515 | Olfr854 | NONE | MEF A&P | chr9 | 19317284 | 19319784 |
| 258517 | Olfr855 | NONE | MEF A&P | chr9 | 19332940 | 19335440 |
| 257963 | Olfr857 | NONE | MEF A&P | chr9 | 19461230 | 19463730 |
| 18358 | Olfr58 | NONE | MEF A&P | chr9 | 19531536 | 19534036 |
| 258519 | Olfr859 | NKLTAG A | MEF A&P | chr9 | 19556721 | 19559221 |
| 258521 | Olfr860 | NONE | MEF A&P | chr9 | 19596519 | 19599019 |
| 258555 | Olfr862 | NONE | MEF A&P | chr9 | 19634205 | 19636705 |
| 258336 | Olfr77 | NONE | MEF A&P | chr9 | 19665630 | 19668130 |
| 258551 | Olfr866 | NONE | MEF A&P | chr9 | 19777838 | 19780338 |
| 257898 | Olfr867 | NONE | MEF A&P | chr9 | 19805363 | 19807863 |
| 258550 | Olfr869 | NONE | MEF A&P | chr9 | 19885603 | 19888103 |
| 57251 | Olfr870 | NONE | MEF A&P | chr9 | 19921471 | 19923971 |
| 258905 | Olfr871 | NONE | MEF A&P | chr9 | 19960752 | 19963252 |
| 258553 | Olfr872 | NONE | MEF A&P | chr9 | 20008327 | 20010827 |
| 258822 | Olfr39 | NONE | MEF A&P | chr9 | 20034102 | 20036602 |
| 258554 | Olfr873 | NONE | MEF A&P | chr9 | 20048612 | 20051112 |
| 75469 | Spata19 | NONE | MEF A&P | chr9 | 27144266 | 27146766 |
| 235106 | Hnt | NONE | MEF A&P | chr9 | 29712075 | 29714575 |
| 68171 | D730048I06Rik | NONE | MEF A&P | chr9 | 35539283 | 35541783 |
| 235973 | A630095E13Rik | NONE | MEF A&P | chr9 | 36387773 | 36390273 |
| 258744 | Olfr875 | NONE | MEF A&P | chr9 | 37520331 | 37522831 |
| 258412 | Olfr877 | NONE | MEF A&P | chr9 | 37602490 | 37604990 |
| 258794 | Olfr878 | NONE | MEF A&P | chr9 | 37666314 | 37668814 |
| 258413 | Olfr881 | NONE | MEF A&P | chr9 | 37741064 | 37743564 |
| 258414 | Olfr883 | NONE | MEF A&P | chr9 | 37774378 | 37776878 |
| 257996 | Olfr884 | NONE | MEF A&P | chr9 | 37795794 | 37798294 |
| 257885 | Olfr885 | NONE | MEF A&P | chr9 | 37809892 | 37812392 |
| 258415 | Olfr887 | NONE | MEF A&P | chr9 | 37833408 | 37835908 |
| 258416 | Olfr888 | NONE | MEF A&P | chr9 | 37857258 | 37859758 |
| 258475 | Olfr889 | NONE | MEF A&P | chr9 | 37864368 | 37866868 |
| 258474 | Olfr890 | NONE | MEF A&P | chr9 | 37891707 | 37894207 |
| 258471 | Olfr891 | NONE | MEF A&P | chr9 | 37930892 | 37933392 |
| 258333 | Olfr893 | NONE | MEF A&P | chr9 | 37957625 | 37960125 |
| 258868 | Olfr894 | NONE | MEF A&P | chr9 | 37967395 | 37969895 |
| 258802 | Olfr143 | NONE | MEF A&P | chr9 | 38001989 | 38004489 |
| 18323 | Olfr25 | NONE | MEF A&P | chr9 | 38078159 | 38080659 |
| 258871 | Olfr898 | NKLTAG A | MEF A&P | chr9 | 38097655 | 38100155 |
| 258869 | Olfr147 | NONE | MEF A&P | chr9 | 38151446 | 38153946 |
| 258028 | Olfr901 | NONE | MEF A&P | chr9 | 38178854 | 38181354 |
| 258798 | Olfr902 | NONE | MEF A&P | chr9 | 38197444 | 38199944 |
| 258797 | Olfr904 | NONE | MEF A&P | chr9 | 38212613 | 38215113 |
| 258800 | Olfr905 | NONE | MEF A&P | chr9 | 38221319 | 38223819 |
| 258799 | Olfr906 | NONE | MEF A&P | chr9 | 38236601 | 38239101 |
| 258801 | Olfr907 | NONE | MEF A&P | chr9 | 38247241 | 38249741 |
| 258872 | Olfr908 | NONE | MEF A&P | chr9 | 38264604 | 38267104 |
| 258807 | Olfr910 | NONE | MEF A&P | chr9 | 38287467 | 38289967 |
| 258225 | Olfr913 | NONE | MEF A&P | chr9 | 38342793 | 38345293 |
| 258782 | Olfr914 | NONE | MEF A&P | chr9 | 38355037 | 38357537 |
| 258781 | Olfr915 | NKLTAG A | MEF A&P | chr9 | 38397593 | 38400093 |
| 258780 | Olfr916 | NONE | MEF A&P | chr9 | 38408461 | 38410961 |
| 258183 | Olfr917 | NONE | MEF A&P | chr9 | 38415913 | 38418413 |
| 258372 | Olfr918 | NONE | MEF A&P | chr9 | 38423552 | 38426052 |
| 258432 | Olfr919 | NONE | MEF A&P | chr9 | 38448447 | 38450947 |
| 258778 | Olfr921 | NONE | MEF A&P | chr9 | 38523827 | 38526327 |
| 258777 | Olfr922 | NONE | MEF A&P | chr9 | 38564075 | 38566575 |
| 258812 | Olfr923 | NONE | MEF A&P | chr9 | 38576263 | 38578763 |
| 258811 | Olfr926 | NONE | MEF A&P | chr9 | 38625748 | 38628248 |
| 258434 | Olfr934 | NONE | MEF A&P | chr9 | 38733113 | 38735613 |
| 258741 | Olfr935 | NONE | MEF A&P | chr9 | 38745504 | 38748004 |
| 258742 | Olfr146 | NONE | MEF A&P | chr9 | 38769610 | 38772110 |
| 258431 | Olfr937 | NONE | MEF A&P | chr9 | 38810735 | 38813235 |
| 258323 | Olfr943 | NONE | MEF A&P | chr9 | 38932750 | 38935250 |
| 258500 | Olfr944 | NONE | MEF A&P | chr9 | 38965929 | 38968429 |
| 257912 | Olfr948 | NONE | MEF A&P | chr9 | 39069683 | 39072183 |
| 235248 | Olfr952 | NONE | MEF A&P | chr9 | 39177140 | 39179640 |
| 258242 | Olfr955 | NONE | MEF A&P | chr9 | 39220795 | 39223295 |
| 258497 | Olfr961 | NONE | MEF A&P | chr9 | 39395298 | 39397798 |
| 258087 | Olfr963 | NONE | MEF A&P | chr9 | 39417629 | 39420129 |
| 235256 | Olfr149 | NONE | MEF A&P | chr9 | 39452838 | 39455338 |
| 258165 | Olfr965 | NONE | MEF A&P | chr9 | 39467799 | 39470299 |
| 258605 | Olfr968 | NONE | MEF A&P | chr9 | 39522869 | 39525369 |
| 258823 | Olfr969 | NONE | MEF A&P | chr9 | 39543947 | 39546447 |
| 258606 | Olfr973 | NONE | MEF A&P | chr9 | 39658375 | 39660875 |
| 258825 | Olfr975 | NKLTAG A | MEF A&P | chr9 | 39700840 | 39703340 |
| 258364 | Olfr976 | NONE | MEF A&P | chr9 | 39707040 | 39709540 |
| 259109 | Olfr978 | NONE | MEF A&P | chr9 | 39742382 | 39744882 |
| 259112 | Olfr979 | NONE | MEF A&P | chr9 | 39751296 | 39753796 |
| 259110 | Olfr980 | NONE | MEF A&P | chr9 | 39757018 | 39759518 |
| 258283 | Olfr981 | NKLTAG A | MEF A&P | chr9 | 39770965 | 39773465 |
| 258853 | Olfr982 | NONE | MEF A&P | chr9 | 39822867 | 39825367 |
| 258824 | Olfr983 | NONE | MEF A&P | chr9 | 39843035 | 39845535 |
| 258854 | Olfr985 | NONE | MEF A&P | chr9 | 39878030 | 39880530 |
| 72821 | Scn2b | NONE | MEF A&P | chr9 | 44866875 | 44869375 |
| 270162 | Elmod1 | NONE | MEF A&P | chr9 | 53772938 | 53775438 |
| 73567 | 1700104A03Rik | NONE | MEF A&P | chr9 | 54099207 | 54101707 |
| 75258 | 4930563M21Rik | NONE | MEF A&P | chr9 | 55778569 | 55781069 |
| 666494 | LOC666494\|Senp6 | NONE | MEF A&P | chr9 | 79850586 | 79853086 |
| 63859 | Impg1 | NONE | MEF A&P | chr9 | 80189991 | 80192491 |
| 20616 | Snap91 | NONE | MEF A&P | chr9 | 86677820 | 86680320 |
| 547109 | LOC547109 | NONE | MEF A&P | chr9 | 88376928 | 88379428 |
| 12044 | Bcl2a1a | NKLTAG A | MEF A&P | chr9 | 88752812 | 88755312 |
| 319213 | 4930579C12Rik | NKLTAG A | MEF A&P | chr9 | 88975280 | 88977780 |
| 12045 | Bcl2a1b\|Bcl2a1a | NKLTAG A | MEF A&P | chr9 | 88995121 | 88997621 |
| 330998 | B230218L05Rik | NONE | MEF A&P | chr9 | 89535868 | 89538368 |
| 22771 | Zic1\|Zic4 | NONE | MEF A&P | chr9 | 91163204 | 91167376 |
| 78911 | Trim42 | NONE | MEF A&P | chr9 | 97178810 | 97181310 |
| 546157 | 7420426K07Rik | NONE | MEF A&P | chr9 | 98710470 | 98712970 |
| 442798 | 9630041A04Rik | NONE | MEF A&P | chr9 | 101792743 | 101795243 |
| 56318 | Acpp | NKLTAG A | MEF A&P | chr9 | 104195567 | 104198067 |
| 320082 | E330009P21Rik | NONE | MEF A&P | chr9 | 109018928 | 109021428 |
| 211305 | Fbxw13 | NKLTAG A | MEF A&P | chr9 | 109052881 | 109055381 |
| 434440 | Fbxw12 | NONE | MEF A&P | chr9 | 109091635 | 109094135 |
| 50757 | Fbxw14 | NKLTAG A | MEF A&P | chr9 | 109144582 | 109147082 |
| 320083 | Fbxw16 | NONE | MEF A&P | chr9 | 109296606 | 109299106 |
| 235612 | Fbxw19 | NONE | MEF A&P | chr9 | 109352760 | 109355260 |
| 382105 | Fbxw15 | NONE | MEF A&P | chr9 | 109425168 | 109427668 |
| 382106 | LOC382106 | NONE | MEF A&P | chr9 | 109482963 | 109485463 |
| 546161 | C85627 | NONE | MEF A&P | chr9 | 109565241 | 109567741 |
| 382109 | LOC382109 | NONE | MEF A&P | chr9 | 109602995 | 109605495 |
| 78709 | C630041L24Rik | NONE | MEF A&P | chr9 | 109672050 | 109674550 |
| 74100 | Arpp21 | NONE | MEF A&P | chr9 | 112032115 | 112034806 |
| 12046 | Bcl2a1c | NONE | MEF A&P | chr9 | 114176853 | 114179353 |
| 24046 | Scn11a | NKLTAG A | MEF A&P | chr9 | 119672058 | 119674558 |
| 17433 | Mobp | NKLTAG A | MEF A&P | chr9 | 119996439 | 119998939 |
| 21922 | Clec3b | NONE | MEF A&P | chr9 | 122997546 | 123000046 |
| 12770 | Ccr1l1 | NKLTAG A | MEF A&P | chr9 | 123826706 | 123829206 |
| 12772 | Ccr2 | NONE | MEF A&P | chr9 | 123948981 | 123951481 |
| 12774 | Ccr5 | NONE | MEF A&P | chr9 | 123968371 | 123970871 |
| 17870 | Mycs | NONE | MEF A&P | chrX | 4644000 | 4646500 |
| 102991 | AU022751 | NONE | MEF A&P | chrX | 5258395 | 5260895 |
| 22232 | Slc35a2 | NONE | MEF A&P | chrX | 7039202 | 7041702 |
| 385312 | Ssxb10 | NONE | MEF A&P | chrX | 7482382 | 7484882 |
| 387131 | Ssxb9 | NONE | MEF A&P | chrX | 7521936 | 7524436 |
| 67985 | Ssxb1 | NKLTAG A | MEF A&P | chrX | 7568263 | 7570763 |
| 387132 | Ssxb2 | NONE | MEF A&P | chrX | 7618185 | 7620685 |
| 278174 | Ssxb3 | NONE | MEF A&P | chrX | 7745705 | 7748205 |
| 382206 | Ssx9 | NONE | MEF A&P | chrX | 7903388 | 7905888 |
| 387586 | Ssxb5 | NONE | MEF A&P | chrX | 7958649 | 7961149 |
| 278255 | BC049702 | NONE | MEF A&P | chrX | 18637778 | 18640278 |
| 236749 | LOC236749 | NONE | MEF A&P | chrX | 22999662 | 23002162 |
| 382275 | MGC118210 | NKLTAG A | MEF A&P | chrX | 24987377 | 24989877 |
| 22526 | Xmr | NKLTAG A | MEF A&P | chrX | 27106997 | 27109497 |
| 245403 | A130007F10Rik | NONE | MEF A&P | chrX | 40612469 | 40614969 |
| 245404 | Wdr40b | NONE | MEF A&P | chrX | 41034278 | 41036778 |
| 73360 | Actrt1 | NONE | MEF A&P | chrX | 42571633 | 42574133 |
| 209268 | Igsf1 | NONE | MEF A&P | chrX | 46039491 | 46044366 |
| 257978 | Olfr1322 | NONE | MEF A&P | chrX | 46130528 | 46133028 |
| 258385 | Olfr1323 | NONE | MEF A&P | chrX | 46254362 | 46256862 |
| 258289 | Olfr1324 | NONE | MEF A&P | chrX | 46670621 | 46673121 |
| 70691 | 3830403N18Rik | NONE | MEF A&P | chrX | 52481355 | 52483855 |
| 245424 | Gpr101 | NONE | MEF A&P | chrX | 53849985 | 53852485 |
| 331424 | C230004F18Rik | NONE | MEF A&P | chrX | 57375062 | 57377562 |
| 434784 | Ldoc1 | NONE | MEF A&P | chrX | 57968278 | 57970778 |
| 70062 | 1700030B21Rik | NONE | MEF A&P | chrX | 61306314 | 61308814 |
| 14266 | Aff2 | NONE | MEF A&P | chrX | 65618995 | 65621495 |
| 619294 | 4930428D18Rik | NONE | MEF A&P | chrX | 72644068 | 72646568 |
| 18249 | Obp1a | NONE | MEF A&P | chrX | 74343593 | 74346093 |
| 17145 | Mageb1 | NONE | MEF A&P | chrX | 88268552 | 88271052 |
| 69357 | 1700003E24Rik | NONE | MEF A&P | chrX | 89433945 | 89436445 |
| 278180 | Vsig4 | NONE | MEF A&P | chrX | 92495657 | 92498157 |
| 26549 | Itgb1bp2 | NONE | MEF A&P | chrX | 97649883 | 97652383 |
| 70887 | 4921520P21Rik\|1700011M02Rik | NONE | MEF A&P | chrX | 99108688 | 99113406 |
| 245566 | Cypt2 | NONE | MEF A&P | chrX | 101700507 | 101703007 |
| 331491 | 5031408O05Rik | NONE | MEF A&P | chrX | 102457591 | 102460091 |
| 245572 | Tbx22 | NONE | MEF A&P | chrX | 103868682 | 103871182 |
| 245572 | Tbx22 | NONE | MEF A&P | chrX | 103879681 | 103882181 |
| 18994 | Pou3f4 | NONE | MEF A&P | chrX | 107015183 | 107017683 |
| 67715 | 2010106E10Rik | NONE | MEF A&P | chrX | 108610217 | 108612717 |
| 54561 | Nap1l3 | NONE | MEF A&P | chrX | 118513829 | 118516329 |
| 83565 | Pramel3\|N/A | NKLTAG A | MEF A&P | chrX | 130646679 | 130649179 |
| 331529 | 4930481M05\|N/A | NKLTAG A | MEF A&P | chrX | 130717647 | 130720147 |
| 245615 | Kir3dl2 | NONE | MEF A&P | chrX | 131814905 | 131817405 |
| 245616 | Kir3dl1 | NONE | MEF A&P | chrX | 131862376 | 131864876 |
| 594844 | Tceal3 | NONE | MEF A&P | chrX | 132010816 | 132013316 |
| 18422 | Ott\|LOC382243 | NKLTAG A | MEF A&P | chrX | 144342319 | 144344819 |
| 436240 | LOC436240 | NONE | MEF A&P | chrX | 148457503 | 148460003 |
| 75185 | 4930542N07Rik | NONE | MEF A&P | chrX | 149820717 | 149823217 |
| 434881 | LOC434881 | NONE | MEF A&P | chrX | 149919546 | 149922046 |
| 17142 | Magea6 | NONE | MEF A&P | chrX | 150276203 | 150278703 |
| 17139 | Magea3 | NONE | MEF A&P | chrX | 150289721 | 150292221 |
| 17141 | Magea5 | NONE | MEF A&P | chrX | 150401769 | 150404269 |
| 17137 | Magea1 | NONE | MEF A&P | chrX | 150429944 | 150432444 |
| 237213 | Glra2 | NONE | MEF A&P | chrX | 160670586 | 160673086 |
| 11856 | Arhgap6 | NKLTAG A | MEF A&P | chrX | 164378733 | 164381233 |
| 16171 | Il17a | NONE | MEF P | chr1 | 20714055 | 20716555 |
| 18986 | Pou2f1 | NONE | MEF P | chr1 | 167771075 | 167773623 |
| 18313 | Olfr16 | NONE | MEF P | chr1 | 174791471 | 174793971 |
| 258717 | Olfr429 | NONE | MEF P | chr1 | 175923716 | 175926216 |
| 16545 | Kera | NONE | MEF P | chr10 | 97034892 | 97037392 |
| 20503 | Slc16a7 | NONE | MEF P | chr10 | 124731095 | 124733595 |
| 116913 | Tpbpb | NONE | MEF P | chr13 | 60913969 | 60916469 |
| 13024 | Ctla2a | NONE | MEF P | chr13 | 60945657 | 60948157 |
| 21984 | Tpbpa | NONE | MEF P | chr13 | 60951057 | 60953557 |
| 432769 | BC038328 | NONE | MEF P | chr13 | 67596573 | 67599073 |
| 105594 | C330003B14Rik | NKLTAG A | MEF P | chr14 | 24773727 | 24776227 |
| 24053 | Sgcg | NKLTAG A | MEF P | chr14 | 60212043 | 60214543 |
| 23888 | Gpc6 | NONE | MEF P | chr14 | 115806386 | 115808886 |
| 223927 | BC048502 | NONE | MEF P | chr15 | 103275993 | 103278493 |
| 224247 | E330017A01Rik | NONE | MEF P | chr16 | 58580446 | 58582946 |
| 224273 | BC043118 | NONE | MEF P | chr16 | 59497508 | 59500008 |
| 224291 | Ckt2 | NONE | MEF P | chr16 | 64420836 | 64423336 |
| 70211 | 2810407A14Rik | NONE | MEF P | chr16 | 87671270 | 87673770 |
| 239931 | Cldn17 | NKLTAG A | MEF P | chr16 | 88395337 | 88397837 |
| 170657 | Krtap16-9 | NKLTAG A | MEF P | chr16 | 88757759 | 88760259 |
| 170654 | Krtap16-4 | NONE | MEF P | chr16 | 88773448 | 88775948 |
| 170939 | AY026312 | NONE | MEF P | chr16 | 88820623 | 88823123 |
| 21461 | Tcp10b\|Tcp10a | NONE | MEF P | chr17 | 7172354 | 7174891 |
| 171227 | V1re4 | NONE | MEF P | chr17 | 20618533 | 20621033 |
| 240041 | A630033E08Rik | NONE | MEF P | chr17 | 22555905 | 22558405 |
| 224754 | H2-M11 | NONE | MEF P | chr17 | 36153127 | 36155627 |
| 14997 | H2-M9 | NONE | MEF P | chr17 | 36250197 | 36252697 |
| 224756 | H2-M1 | NONE | MEF P | chr17 | 36279750 | 36282250 |
| 258505 | Olfr97 | NONE | MEF P | chr17 | 36839921 | 36842421 |
| 17287 | Mep1a | NONE | MEF P | chr17 | 42966656 | 42969156 |
| 13527 | Dtna | NKLTAG A | MEF P | chr18 | 23556451 | 23558951 |
| 433178 | LOC433178 | NONE | MEF P | chr18 | 44151791 | 44154291 |
| 170834 | Oosp1 | NONE | MEF P | chr19 | 11757596 | 11760096 |
| 170738 | Kcnh7 | NONE | MEF P | chr2 | 62989922 | 62992422 |
| 26877 | B3galt1 | NONE | MEF P | chr2 | 67916566 | 67919066 |
| 329436 | A830093M07 | NONE | MEF P | chr2 | 78036385 | 78038885 |
| 21884 | Fabp9 | NKLTAG A | MEF P | chr3 | 10179254 | 10181754 |
| 242100 | Pglyrp3 | NONE | MEF P | chr3 | 92098538 | 92101038 |
| 20758 | Sprr2d | NONE | MEF P | chr3 | 92422560 | 92425060 |
| 14408 | Gabrr1 | NONE | MEF P | chr4 | 33459168 | 33461668 |
| 242384 | Lrrn6c | NONE | MEF P | chr4 | 36033583 | 36036083 |
| 56079 | Astn2 | NONE | MEF P | chr4 | 65890344 | 65892844 |
| 15572 | Elavl4 | NONE | MEF P | chr4 | 109784266 | 109787443 |
| 16826 | Ldb2 | NONE | MEF P | chr5 | 45087367 | 45089867 |
| 435845 | Tmprss11c | NKLTAG A | MEF P | chr5 | 87363321 | 87365821 |
| 71773 | Ugt2b1 | NONE | MEF P | chr5 | 88000564 | 88003064 |
| 17295 | Met | NONE | MEF P | chr6 | 17439240 | 17441740 |
| 12343 | Capza2 | NONE | MEF P | chr6 | 17585097 | 17587597 |
| 67690 | 1700016G05Rik | NONE | MEF P | chr6 | 40448613 | 40451113 |
| 258380 | Olfr461 | NONE | MEF P | chr6 | 40474084 | 40476584 |
| 258381 | Olfr460 | NONE | MEF P | chr6 | 40498994 | 40501494 |
| 23925 | Kel | NONE | MEF P | chr6 | 41633396 | 41635896 |
| 12515 | Cd69 | NONE | MEF P | chr6 | 129240562 | 129243062 |
| 387351 | Tas2r124 | NONE | MEF P | chr6 | 132718422 | 132720922 |
| 18705 | Pik3c2g | NONE | MEF P | chr6 | 139801850 | 139804350 |
| 22310 | V2r4 | NONE | MEF P | chr7 | 8139397 | 8141897 |
| 56640 | Klk4 | NKLTAG A | MEF P | chr7 | 43747213 | 43749713 |
| 259050 | Olfr652 | NONE | MEF P | chr7 | 104436043 | 104438543 |
| 257914 | Olfr663 | NONE | MEF P | chr7 | 104575389 | 104577889 |
| 259100 | Olfr666 | NONE | MEF P | chr7 | 104766947 | 104769447 |
| 259062 | Olfr667 | NKLTAG A | MEF P | chr7 | 104790615 | 104793115 |
| 384703 | Olfr670 | NONE | MEF P | chr7 | 104834051 | 104836551 |
| 244334 | Defb8 | NONE | MEF P | chr8 | 19447083 | 19449583 |
| 13226 | Defcr-rs7 | NONE | MEF P | chr8 | 22684365 | 22686865 |
| 246081 | Defb11 | NONE | MEF P | chr8 | 23371456 | 23373956 |
| 208924 | A730045E13Rik | NONE | MEF P | chr8 | 25637448 | 25639948 |
| 244416 | Ppp1r3b | NONE | MEF P | chr8 | 36842257 | 36844757 |
| 319582 | 6430573F11Rik | NONE | MEF P | chr8 | 37955707 | 37958207 |
| 23793 | Adam25 | NONE | MEF P | chr8 | 42249024 | 42251524 |
| 546055 | Adam39 | NONE | MEF P | chr8 | 42319864 | 42322364 |
| 234203 | Zfp353 | NONE | MEF P | chr8 | 43582696 | 43585196 |
| 244448 | BC050188 | NONE | MEF P | chr8 | 44639803 | 44642303 |
| 382007 | Adam26b | NONE | MEF P | chr8 | 45020280 | 45022780 |
| 13525 | Adam26a | NONE | MEF P | chr8 | 45074983 | 45077483 |
| 319555 | A230063L24Rik | NONE | MEF P | chr8 | 75538414 | 75540914 |
| 234515 | Inpp4b | NONE | MEF P | chr8 | 84608946 | 84611446 |
| 67555 | 4933434I20Rik | NONE | MEF P | chr8 | 86236576 | 86239076 |
| 258267 | Olfr370 | NONE | MEF P | chr8 | 86429250 | 86431750 |
| 624855 | LOC624855 | NONE | MEF P | chr8 | 88040028 | 88042528 |
| 13884 | Es1 | NONE | MEF P | chr8 | 96019899 | 96022399 |
| 72230 | Zfp558\|Mbd3l1 | NONE | MEF P | chr9 | 18226796 | 18229296 |
| 258327 | Olfr958 | NONE | MEF P | chr9 | 39300940 | 39303440 |
| 258498 | Olfr148 | NONE | MEF P | chr9 | 39362139 | 39364639 |
| 258276 | Olfr960 | NONE | MEF P | chr9 | 39371695 | 39374195 |
| 54725 | Igsf4a | NONE | MEF P | chr9 | 47279346 | 47281846 |
| 331374 | Dgkk | NONE | MEF P | chrX | 6028444 | 6030944 |
| 434759 | Rhox4c | NONE | MEF P | chrX | 33784293 | 33786793 |
| 57737 | Rhox4b\|Rhox4d | NONE | MEF P | chrX | 33818491 | 33820991 |
| 385354 | Gm1533 | NONE | MEF P | chrX | 47139749 | 47142249 |
| 78755 | 4632404H22Rik\|4930432H15Rik | NONE | MEF P | chrX | 49516059 | 49518559 |
| 236798 | Gpr112 | NONE | MEF P | chrX | 53308189 | 53310689 |
| 77905 | Fate1 | NONE | MEF P | chrX | 68223705 | 68226205 |
| 71351 | 5430402E10Rik | NONE | MEF P | chrX | 74239009 | 74241509 |
| 272790 | Magee2 | NONE | MEF P | chrX | 101059375 | 101061875 |
| 78826 | P2ry10 | NONE | MEF P | chrX | 103290053 | 103292553 |
| 331535 | Serpina7 | NONE | MEF P | chrX | 134429565 | 134432065 |
| 16165 | Il13ra2 | NONE | MEF P | chrX | 142649704 | 142652204 |
| 80884 | Maged2 | NONE | MEF P | chrX | 146154498 | 146156998 |
| 66106 | Smpx | NONE | MEF P | chrX | 153041222 | 153043722 |
|  |  |  |  |  |  |  |

**Table S2: Transcription factor motif (TFM) analysis of A-type lamin-associated gene promoters**

|  | NKLTAG A Target | | MEF A&P Target | | MEF P Target | |
| --- | --- | --- | --- | --- | --- | --- |
| *TF Family* | *N* | *P-value* | *N* | *P-value* | *N* | *P-value* |
| V$HNF6 | 581 | 8.60E-01 | 2505 | **2.49E-59** | 119 | **1.53E-02** |
| V$RUSH | 909 | 2.83E-01 | 3505 | **5.59E-56** | 175 | **3.48E-03** |
| V$BRN5 | 1246 | 7.68E-01 | 5947 | **1.82E-55** | 297 | **1.51E-04** |
| V$BRNF | 1955 | 9.12E-01 | 9058 | **6.50E-54** | 490 | **4.88E-06** |
| V$DMRT | 1069 | 2.94E-01 | 4244 | **3.36E-49** | 220 | **5.52E-04** |
| V$OCT1 | 2113 | 5.13E-01 | 9527 | **2.46E-45** | 511 | **1.31E-03** |
| V$PDX1 | 568 | 9.78E-01 | 2585 | **3.84E-45** | 161 | **1.10E-04** |
| V$CDXF | 793 | 9.90E-01 | 3215 | **5.90E-44** | 159 | **2.20E-04** |
| V$GATA | 1431 | 5.20E-01 | 5457 | **1.35E-41** | 306 | **5.58E-05** |
| V$ARID | 817 | 9.99E-01 | 4032 | **3.36E-41** | 226 | **7.84E-06** |
| V$CLOX | 1029 | 5.99E-01 | 3994 | **1.19E-39** | 203 | **6.73E-04** |
| V$HOXC | 1025 | 8.52E-01 | 4091 | **1.69E-39** | 198 | **1.58E-02** |
| V$PIT1 | 427 | 1.00E+00 | 2230 | **3.62E-39** | 125 | **3.71E-02** |
| V$FKHD | 1900 | 8.80E-01 | 7601 | **2.17E-38** | 354 | **5.35E-04** |
| V$PARF | 1122 | 9.98E-01 | 5014 | **1.11E-37** | 245 | **3.31E-02** |
| V$LHXF | 1674 | 9.94E-01 | 7880 | **2.00E-37** | 412 | **1.93E-02** |
| V$STEM | 870 | 9.05E-01 | 3206 | **2.39E-37** | 174 | **4.09E-03** |
| V$SRFF | 885 | 5.39E-01 | 2944 | **5.95E-36** | 149 | **8.63E-03** |
| V$FAST | 543 | 2.94E-01 | 1904 | **1.05E-35** | 126 | **9.99E-07** |
| V$HOXF | 2138 | 9.77E-01 | 9245 | **2.21E-33** | 519 | **1.32E-04** |
| V$TALE | 608 | 5.75E-01 | 2149 | **6.91E-30** | 82 | 6.99E-01 |
| V$AIRE | 214 | **7.02E-04** | 689 | **3.06E-29** | 32 | **4.02E-02** |
| V$DLXF | 648 | 9.87E-01 | 2939 | **9.38E-29** | 175 | **2.38E-04** |
| V$EVI1 | 1736 | 8.79E-01 | 6356 | **1.75E-28** | 294 | **1.26E-02** |
| V$CART | 1461 | 9.99E-01 | 6662 | **2.37E-28** | 395 | **8.29E-03** |
| V$NKX6 | 685 | 1.00E+00 | 3392 | **3.84E-28** | 185 | **2.04E-03** |
| V$LEFF | 1129 | 9.62E-01 | 4323 | **2.18E-27** | 230 | **1.80E-03** |
| V$BPTF | 278 | **4.26E-04** | 796 | **1.49E-26** | 38 | 1.38E-01 |
| V$ATBF | 263 | 1.00E+00 | 1424 | **3.22E-26** | 83 | **1.40E-04** |
| V$PBXC | 500 | 5.53E-01 | 1742 | **3.23E-26** | 79 | **2.84E-02** |
| V$ABDB | 1371 | 8.71E-01 | 5410 | **6.49E-26** | 303 | **3.91E-03** |
| V$SORY | 2281 | 1.00E+00 | 9337 | **8.86E-26** | 489 | 3.01E-01 |
| V$HMTB | 343 | 8.80E-01 | 1302 | **2.11E-25** | 63 | 5.56E-02 |
| V$HBOX | 1540 | 1.00E+00 | 6690 | **2.26E-25** | 401 | **7.65E-05** |
| V$HOXH | 485 | 9.36E-01 | 1853 | **2.66E-25** | 90 | **3.56E-02** |
| V$HNF1 | 1339 | 9.94E-01 | 5224 | **1.60E-22** | 277 | **4.20E-02** |
| V$IRXF | 573 | 9.86E-02 | 1855 | **1.74E-22** | 89 | **1.45E-02** |
| V$MEF2 | 540 | 9.99E-01 | 2315 | **6.59E-22** | 133 | **1.71E-04** |
| V$ZF03 | 346 | 7.63E-02 | 1183 | **6.80E-21** | 58 | **2.33E-02** |
| V$OVOL | 417 | 6.37E-01 | 1516 | **3.30E-20** | 72 | 2.97E-01 |
| V$SATB | 193 | 1.00E+00 | 1101 | **2.09E-19** | 52 | 2.35E-01 |
| V$GFI1 | 377 | 6.21E-01 | 1303 | **1.74E-18** | 59 | 6.95E-01 |
| V$CEBP | 578 | 1.00E+00 | 2289 | **1.76E-18** | 130 | **1.86E-02** |
| V$PAX2 | 317 | 9.99E-01 | 1307 | **8.01E-17** | 62 | 1.91E-01 |
| V$NFAT | 945 | 1.00E+00 | 3327 | **8.11E-17** | 173 | 8.00E-02 |
| V$AP1F | 443 | 9.86E-01 | 1551 | **1.46E-16** | 110 | **1.25E-04** |
| V$MYT1 | 966 | 1.00E+00 | 3989 | **6.16E-16** | 222 | **3.33E-02** |
| V$NKX1 | 305 | 9.99E-01 | 1337 | **1.06E-15** | 92 | **2.55E-03** |
| V$SNAP | 467 | 9.98E-01 | 1907 | **2.31E-15** | 109 | **5.37E-03** |
| V$PLZF | 273 | 9.98E-01 | 1050 | **4.03E-15** | 53 | 1.38E-01 |
| V$PAXH | 302 | 1.00E+00 | 1474 | **5.50E-13** | 94 | 8.53E-02 |
| V$BCDF | 642 | 9.97E-01 | 2402 | **5.72E-12** | 137 | **7.90E-03** |
| V$YY1F | 747 | 9.98E-01 | 2622 | **1.34E-11** | 154 | **3.48E-03** |
| V$RP58 | 186 | 6.58E-02 | 599 | **2.40E-11** | 26 | 2.53E-01 |
| V$PCBE | 157 | 9.39E-01 | 583 | **2.84E-11** | 39 | **2.03E-02** |
| V$NKXH | 1323 | 1.00E+00 | 5490 | **4.43E-11** | 317 | 5.70E-02 |
| V$CIZF | 232 | 9.58E-01 | 843 | **6.55E-11** | 45 | **1.62E-02** |
| V$TCFF | 68 | 8.34E-01 | 305 | **1.42E-10** | 9 | 7.94E-01 |
| V$PRDF | 417 | 9.87E-01 | 1437 | **3.46E-10** | 90 | **9.82E-04** |
| V$BCL6 | 545 | 1.00E+00 | 2250 | **4.62E-10** | 105 | 2.48E-01 |
| V$CAAT | 831 | 8.75E-01 | 2643 | **6.65E-10** | 136 | 2.27E-01 |
| V$HOMF | 2636 | 1.00E+00 | 10767 | **1.37E-09** | 634 | 4.03E-01 |
| V$TEAF | 352 | 6.92E-01 | 1117 | **2.98E-09** | 61 | **3.46E-02** |
| V$SALL | 68 | 1.00E+00 | 344 | **3.24E-08** | 8 | 9.47E-01 |
| V$GREF | 1056 | 7.56E-01 | 3077 | **5.23E-08** | 150 | 1.80E-01 |
| V$RBPF | 387 | 7.03E-01 | 1099 | **3.80E-07** | 62 | 1.98E-01 |
| V$CHRF | 318 | 9.71E-01 | 1096 | **4.26E-07** | 64 | **1.17E-02** |
| V$NBRE | 194 | 6.66E-01 | 614 | **1.15E-06** | 30 | 1.62E-01 |
| V$IRFF | 994 | 1.00E+00 | 3335 | **1.61E-06** | 165 | **9.41E-03** |
| V$SIXF | 334 | 6.85E-01 | 1014 | **1.94E-06** | 55 | **1.57E-02** |
| V$RORA | 624 | 8.73E-01 | 2005 | **2.44E-06** | 99 | **6.51E-03** |
| V$STAT | 1145 | 1.00E+00 | 4360 | **4.91E-06** | 186 | 4.54E-01 |
| V$GZF1 | 192 | 4.66E-01 | 603 | **9.53E-06** | 34 | **3.25E-02** |
| V$PERO | 814 | 9.97E-01 | 2477 | **1.49E-05** | 122 | 8.04E-01 |
| V$BARB | 266 | 5.20E-01 | 814 | **2.04E-05** | 50 | 2.89E-01 |
| V$PAX3 | 490 | 9.63E-01 | 1365 | **1.67E-04** | 76 | **1.68E-02** |
| V$HZIP | 81 | 7.73E-01 | 267 | **5.89E-04** | 16 | 2.60E-01 |
| V$HAML | 436 | **3.15E-04** | 1075 | **5.91E-04** | 56 | 4.80E-01 |
| V$PPAR | 144 | 8.49E-01 | 459 | **7.38E-04** | 17 | 7.38E-01 |
| V$AARF | 55 | 9.99E-01 | 258 | **7.40E-04** | 13 | 2.28E-01 |
| V$THAP | 102 | 9.52E-01 | 356 | **7.78E-04** | 13 | 7.16E-01 |
| V$ZF05 | 257 | 5.44E-02 | 666 | **1.72E-03** | 35 | 1.42E-01 |
| V$CHOP | 65 | 9.02E-01 | 228 | **1.83E-03** | 17 | **2.03E-02** |
| V$NEUR | 637 | 9.92E-01 | 1905 | **2.60E-03** | 82 | 8.70E-01 |
| V$EREF | 567 | 9.84E-01 | 1670 | **2.85E-03** | 81 | 4.84E-01 |
| V$ZF10 | 273 | **3.32E-02** | 761 | **3.39E-03** | 48 | **2.85E-02** |
| V$CABL | 72 | 9.94E-01 | 298 | **3.76E-03** | 10 | 7.45E-01 |
| V$MITF | 241 | 2.43E-01 | 573 | **5.36E-03** | 29 | 3.42E-01 |
| V$E4FF | 196 | 9.80E-01 | 612 | **5.36E-03** | 38 | **1.96E-02** |
| V$IKRS | 397 | 1.00E+00 | 1330 | **5.41E-03** | 64 | 7.64E-01 |
| V$NR2F | 1494 | 1.00E+00 | 4712 | **1.07E-02** | 210 | 8.31E-01 |
| V$HEAT | 1279 | 1.00E+00 | 4532 | **4.31E-02** | 247 | 3.00E-01 |
| V$AP1R | 1115 | 1.00E+00 | 3778 | 5.18E-02 | 193 | 1.15E-01 |
| V$DICE | 227 | 8.75E-01 | 632 | 7.94E-02 | 24 | 8.23E-01 |
| V$ZF09 | 6 | 6.47E-01 | 33 | 8.07E-02 | 11 | 6.20E-02 |
| V$BTBF | 143 | 8.00E-02 | 349 | 9.06E-02 | 15 | 8.30E-01 |
| V$RU49 | 199 | 9.46E-01 | 635 | 1.03E-01 | 43 | 1.66E-01 |
| V$FXRE | 132 | 9.91E-01 | 453 | 1.13E-01 | 24 | 3.30E-01 |
| V$OSRF | 110 | 9.96E-01 | 383 | 1.68E-01 | 17 | 6.36E-01 |
| V$BRAC | 515 | 9.11E-01 | 1357 | 2.48E-01 | 63 | 7.58E-01 |
| V$GRHL | 318 | 1.00E+00 | 981 | 3.42E-01 | 48 | 9.03E-01 |
| V$PTF1 | 174 | 7.96E-01 | 516 | 3.49E-01 | 24 | 8.73E-01 |
| V$RXRF | 1521 | 9.80E-01 | 4307 | 4.34E-01 | 203 | 8.71E-01 |
| V$SF1F | 365 | 5.98E-01 | 918 | 5.05E-01 | 57 | **4.62E-02** |
| V$PAX1 | 88 | 3.80E-01 | 204 | 5.44E-01 | 11 | 4.30E-01 |
| V$GUCE | 24 | 2.10E-01 | 47 | 5.87E-01 | 1 | **2.41E-02** |
| V$HAND | 1531 | 1.00E+00 | 4394 | 6.21E-01 | 184 | 8.27E-01 |
| V$NF1F | 452 | 9.88E-01 | 1337 | 6.77E-01 | 59 | 9.52E-01 |
| V$GCNR | 36 | 9.28E-01 | 115 | 7.19E-01 | 5 | 6.95E-01 |
| V$GTBX | 62 | 9.59E-01 | 183 | 7.37E-01 | 5 | 9.71E-01 |
| V$ZFHX | 534 | 9.83E-01 | 1547 | 8.32E-01 | 74 | 6.41E-01 |
| V$ZF06 | 100 | 9.98E-01 | 297 | 8.60E-01 | 12 | 8.89E-01 |
| V$XBBF | 970 | 1.00E+00 | 2575 | 9.01E-01 | 126 | 8.24E-01 |
| V$MYOD | 713 | 1.00E+00 | 1971 | 9.57E-01 | 97 | 8.74E-01 |
| V$ZF04 | 129 | 6.34E-01 | 276 | 9.95E-01 | 14 | 7.29E-01 |
| V$CSEN | 141 | 7.67E-01 | 343 | 9.97E-01 | 15 | 9.50E-01 |
| V$PAX6 | 842 | 9.99E-01 | 2411 | 9.97E-01 | 154 | 4.62E-01 |
| V$P53F | 572 | 9.88E-01 | 1548 | 9.98E-01 | 70 | 9.68E-01 |
| V$MEF3 | 216 | 9.97E-01 | 585 | 9.98E-01 | 18 | 9.99E-01 |
| V$ZF35 | 148 | 9.95E-01 | 389 | 9.99E-01 | 30 | 2.60E-01 |
| V$MYBL | 993 | 1.00E+00 | 3024 | 9.99E-01 | 166 | 8.73E-01 |
| V$YBXF | 186 | 7.75E-01 | 437 | 1.00E+00 | 26 | 6.84E-01 |
| V$SMAD | 497 | 6.52E-01 | 1016 | 1.00E+00 | 49 | 9.62E-01 |
| V$CREB | 1221 | 1.00E+00 | 4102 | 1.00E+00 | 222 | 8.74E-01 |
| V$HICF | 260 | 7.64E-01 | 601 | 1.00E+00 | 36 | 5.38E-01 |
| V$ZF08 | 67 | 9.90E-01 | 170 | 1.00E+00 | 19 | 5.85E-01 |
| V$DMTF | 123 | 9.96E-01 | 317 | 1.00E+00 | 12 | 9.90E-01 |
| V$SIX3 | 17 | 1.00E+00 | 60 | 1.00E+00 | 6 | 4.02E-01 |
| V$BNCF | 173 | 2.45E-01 | 305 | 1.00E+00 | 14 | 9.38E-01 |
| V$NFKB | 477 | 1.00E+00 | 1397 | 1.00E+00 | 71 | 9.25E-01 |
| V$HDBP | 15 | 1.00E+00 | 11 | 1.00E+00 | 2 | 1.62E-01 |
| V$GABF | 568 | 7.83E-01 | 1236 | 1.00E+00 | 51 | 5.52E-01 |
| V$ZICF | 249 | 1.00E+00 | 506 | 1.00E+00 | 33 | 7.33E-01 |
| V$LTFM | 185 | 9.03E-01 | 412 | 1.00E+00 | 26 | 7.61E-01 |
| V$PRDM | 182 | 1.00E+00 | 381 | 1.00E+00 | 24 | 7.92E-01 |
| V$ETSF | 2025 | 1.00E+00 | 5875 | 1.00E+00 | 280 | 8.54E-01 |
| V$HASF | 101 | 1.00E+00 | 198 | 1.00E+00 | 16 | 8.61E-01 |
| V$INSM | 314 | 8.84E-01 | 565 | 1.00E+00 | 40 | 8.74E-01 |
| V$MOKF | 414 | 1.00E+00 | 1103 | 1.00E+00 | 63 | 9.07E-01 |
| V$CP2F | 431 | 1.00E+00 | 960 | 1.00E+00 | 55 | 9.29E-01 |
| V$NOLF | 345 | 1.00E+00 | 720 | 1.00E+00 | 47 | 9.37E-01 |
| V$GCMF | 704 | 9.88E-01 | 1758 | 1.00E+00 | 83 | 9.37E-01 |
| V$SREB | 181 | 9.96E-01 | 431 | 1.00E+00 | 18 | 9.73E-01 |
| V$AP4R | 206 | 9.97E-01 | 491 | 1.00E+00 | 24 | 9.76E-01 |
| V$PAX5 | 666 | 1.00E+00 | 1884 | 1.00E+00 | 93 | 9.86E-01 |
| V$OAZF | 161 | 1.00E+00 | 316 | 1.00E+00 | 19 | 9.87E-01 |
| V$STAF | 314 | 1.00E+00 | 717 | 1.00E+00 | 39 | 9.92E-01 |
| V$RBP2 | 149 | 6.66E-01 | 271 | 1.00E+00 | 9 | 9.94E-01 |
| V$SP1F | 815 | 9.95E-01 | 1276 | 1.00E+00 | 80 | 9.94E-01 |
| V$NRSF | 373 | 1.00E+00 | 853 | 1.00E+00 | 44 | 9.96E-01 |
| V$PURA | 33 | 1.00E+00 | 54 | 1.00E+00 | 4 | 9.97E-01 |
| V$SPZ1 | 239 | 9.98E-01 | 462 | 1.00E+00 | 20 | 9.98E-01 |
| V$MZF1 | 531 | 1.00E+00 | 1150 | 1.00E+00 | 62 | 9.98E-01 |
| V$KLFS | 1550 | 1.00E+00 | 3271 | 1.00E+00 | 185 | 9.99E-01 |
| V$DEAF | 86 | 1.00E+00 | 173 | 1.00E+00 | 11 | 9.99E-01 |
| V$ZF07 | 222 | 1.00E+00 | 358 | 1.00E+00 | 27 | 9.99E-01 |
| V$MAZF | 405 | 1.00E+00 | 616 | 1.00E+00 | 48 | 9.99E-01 |
| V$ZF01 | 150 | 1.00E+00 | 332 | 1.00E+00 | 13 | 1.00E+00 |
| V$HIFF | 246 | 1.00E+00 | 510 | 1.00E+00 | 26 | 1.00E+00 |
| V$NGRE | 268 | 1.00E+00 | 747 | 1.00E+00 | 25 | 1.00E+00 |
| V$PAX9 | 49 | 1.00E+00 | 80 | 1.00E+00 | 5 | 1.00E+00 |
| V$HNFP | 35 | 1.00E+00 | 61 | 1.00E+00 | 2 | 1.00E+00 |
| V$RREB | 413 | 9.02E-02 | 641 | 1.00E+00 | 27 | 1.00E+00 |
| V$ZFXY | 88 | 1.00E+00 | 135 | 1.00E+00 | 9 | 1.00E+00 |
| V$CARE | 133 | 1.00E+00 | 333 | 1.00E+00 | 9 | 1.00E+00 |
| V$MTF1 | 190 | 8.52E-01 | 370 | 1.00E+00 | 9 | 1.00E+00 |
| V$EBOX | 585 | 1.00E+00 | 1148 | 1.00E+00 | 57 | 1.00E+00 |
| V$ZF02 | 1062 | 9.92E-01 | 1586 | 1.00E+00 | 81 | 1.00E+00 |
| V$E2FF | 688 | 1.00E+00 | 1438 | 1.00E+00 | 73 | 1.00E+00 |
| V$WHNF | 82 | 1.00E+00 | 151 | 1.00E+00 | 7 | 1.00E+00 |
| V$AHRR | 197 | 1.00E+00 | 333 | 1.00E+00 | 26 | 1.00E+00 |
| V$AP2F | 344 | 1.00E+00 | 623 | 1.00E+00 | 33 | 1.00E+00 |
| V$HESF | 478 | 1.00E+00 | 868 | 1.00E+00 | 49 | 1.00E+00 |
| V$PLAG | 632 | 1.00E+00 | 1099 | 1.00E+00 | 60 | 1.00E+00 |
| V$NRF1 | 58 | 1.00E+00 | 78 | 1.00E+00 | 6 | 1.00E+00 |
| V$GLIF | 602 | 9.99E-01 | 1048 | 1.00E+00 | 48 | 1.00E+00 |
| V$CDEF | 35 | 1.00E+00 | 36 | 1.00E+00 | 2 | 1.00E+00 |
| V$CHRE | 61 | 1.00E+00 | 103 | 1.00E+00 | 2 | 1.00E+00 |
| V$EGRF | 793 | 1.00E+00 | 1110 | 1.00E+00 | 71 | 1.00E+00 |
| V$CTCF | 290 | 1.00E+00 | 483 | 1.00E+00 | 20 | 1.00E+00 |
| V$ZF5F | 104 | 1.00E+00 | 125 | 1.00E+00 | 17 | 1.00E+00 |
|  |  |  |  |  |  |  |
| Legend: TFM analysis as described in material and methods shows the amount of TFMs (N) identified in NKLTAG Lamin A-associated targets, MEF A&P Targets and MEF P targets. Significant enrichment for TFM are indicated in bold (p<0.05). | | | | | | |

**Table S3: Highly expressed lamin A-associated genes**

| Entrez Gene ID | Gene Abbreviation | Target Type | Expression WT | Expression LMNA^GT-/-^ | FC LMNA^GT-/-^  vs. WT | P-value |
| --- | --- | --- | --- | --- | --- | --- |
| 19091 | Tfrc | NKLtag OSTA target | 6.77 | 5.55 | 0.82 | >0.05 |
| 194655 | Klf11 | NKLtag OSTA target | 6.78 | 6.98 | 1.03 | >0.05 |
| 13171 | Dbt | NKLtag OSTA target | 6.83 | 7.35 | 1.08 | >0.05 |
| 67203 | Nde1 | NKLtag OSTA target | 6.83 | 6.77 | 0.99 | >0.05 |
| 103425 | Ncln | NKLtag OSTA target | 6.84 | 6.70 | 0.98 | >0.05 |
| 268417 | Zkscan17 | NKLtag OSTA target | 6.86 | 6.67 | 0.97 | >0.05 |
| 252972 | Tpcn1 | NKLtag OSTA target | 6.87 | 6.90 | 1.00 | >0.05 |
| 74094 | Tjap1 | NKLtag OSTA target | 6.89 | 6.81 | 0.99 | >0.05 |
| 18738 | Pitpna | NKLtag OSTA target | 7.02 | 6.91 | 0.99 | >0.05 |
| 23897 | Hax1 | NKLtag OSTA target | 7.06 | 6.95 | 0.99 | >0.05 |
| 14755 | Pigq | NKLtag OSTA target | 7.13 | 7.13 | 1.00 | >0.05 |
| 224129 | Adcy5 | NKLtag OSTA target | 7.19 | 7.28 | 1.01 | >0.05 |
| 67912 | 1600012H06Rik | NKLtag OSTA target | 7.26 | 7.33 | 1.01 | >0.05 |
| 227648 | Sec16a | NKLtag OSTA target | 7.27 | 7.42 | 1.02 | >0.05 |
| 58887 | Repin1 | NKLtag OSTA target | 7.31 | 6.82 | 0.93 | **<0.05** |
| 71699 | Slc41a3 | NKLtag OSTA target | 7.39 | 6.66 | 0.90 | >0.05 |
| 545428 | 2610301F02Rik | NKLtag OSTA target | 7.39 | 7.44 | 1.01 | >0.05 |
| 384382 | NuGO_emt086506_x_at | NKLtag OSTA target | 7.49 | 7.79 | 1.04 | >0.05 |
| 50766 | Crim1 | NKLtag OSTA target | 7.51 | 8.72 | 1.16 | >0.05 |
| 13195 | Ddc | NKLtag OSTA target | 7.60 | 7.79 | 1.02 | >0.05 |
| 105245 | Txndc5 | NKLtag OSTA target | 7.64 | 7.01 | 0.92 | >0.05 |
| 381236 | AI747699 | NKLtag OSTA target | 7.65 | 7.44 | 0.97 | >0.05 |
| 56744 | Pf4 | NKLtag OSTA target | 7.71 | 7.32 | 0.95 | >0.05 |
| 24053 | Sgcg | NKLtag OSTA target | 7.73 | 7.59 | 0.98 | >0.05 |
| 545007 | ENSMUSG00000068790 | NKLtag OSTA target | 7.76 | 8.71 | 1.12 | >0.05 |
| 26357 | Abcg2 | NKLtag OSTA target | 7.80 | 7.88 | 1.01 | >0.05 |
| 20755 | Plxnb2 | NKLtag OSTA target | 7.90 | 7.87 | 1.00 | >0.05 |
| 79059 | Nme3 | NKLtag OSTA target | 8.01 | 6.67 | 0.83 | >0.05 |
| 67881 | 1810034K20Rik | NKLtag OSTA target | 8.01 | 8.04 | 1.00 | >0.05 |
| 11350 | Abl1 | NKLtag OSTA target | 8.02 | 7.98 | 0.99 | >0.05 |
| 11745 | Anxa3 | NKLtag OSTA target | 8.06 | 8.26 | 1.02 | >0.05 |
| 17533 | Mrc1 | NKLtag OSTA target | 8.09 | 7.58 | 0.94 | >0.05 |
| 11632 | Aip | NKLtag OSTA target | 8.10 | 7.69 | 0.95 | >0.05 |
| 68760 | Synpo2l | NKLtag OSTA target | 8.11 | 8.74 | 1.08 | >0.05 |
| 54197 | Rnf5 | NKLtag OSTA target | 8.13 | 7.63 | 0.94 | >0.05 |
| 71323 | Rassf8 | NKLtag OSTA target | 8.13 | 8.00 | 0.98 | >0.05 |
| 56453 | Mbtps1 | NKLtag OSTA target | 8.14 | 8.35 | 1.03 | >0.05 |
| 545007 | ENSMUSG00000068790 | NKLtag OSTA target | 8.15 | 9.26 | 1.14 | >0.05 |
| 66990 | Tmem134 | NKLtag OSTA target | 8.17 | 7.60 | 0.93 | >0.05 |
| 66462 | 2810428I15Rik | NKLtag OSTA target | 8.24 | 7.73 | 0.94 | >0.05 |
| 76299 | Txndc4 | NKLtag OSTA target | 8.38 | 7.71 | 0.92 | >0.05 |
| 280408 | Rilp | NKLtag OSTA target | 8.40 | 8.18 | 0.97 | >0.05 |
| 381598 | 6820431F20Rik | NKLtag OSTA target | 8.58 | 8.54 | 1.00 | >0.05 |
| 77106 | Tmem181 | NKLtag OSTA target | 8.72 | 8.93 | 1.02 | >0.05 |
| 18798 | Plcb4 | NKLtag OSTA target | 8.85 | 9.16 | 1.04 | >0.05 |
| 12391 | Cav3 | NKLtag OSTA target | 8.88 | 8.58 | 0.97 | >0.05 |
| 21687 | Tek | NKLtag OSTA target | 9.08 | 9.17 | 1.01 | >0.05 |
| 19179 | Psmc1 | NKLtag OSTA target | 9.50 | 9.22 | 0.97 | >0.05 |
| 110454 | Ly6a | NKLtag OSTA target | 9.79 | 10.16 | 1.04 | >0.05 |
| 384382 | 384382 | NKLtag OSTA target | 9.90 | 9.93 | 1.00 | >0.05 |
| 77106 | Tmem181 | NKLtag OSTA target | 9.93 | 9.68 | 0.97 | >0.05 |
| 57296 | Psmd8 | NKLtag OSTA target | 9.95 | 9.32 | 0.94 | >0.05 |
| 66066 | Gng11 | NKLtag OSTA target | 10.21 | 9.86 | 0.97 | >0.05 |
| 68460 | Dhrs7c | NKLtag OSTA target | 10.24 | 10.17 | 0.99 | >0.05 |
| 16562 | Kif1c | NKLtag OSTA target | 10.30 | 10.54 | 1.02 | >0.05 |
| 54198 | Snx3 | NKLtag OSTA target | 10.53 | 10.14 | 0.96 | >0.05 |
| 56486 | Gabarap | NKLtag OSTA target | 10.61 | 9.92 | 0.94 | >0.05 |
| 14790 | Grcc10 | NKLtag OSTA target | 10.66 | 10.07 | 0.94 | >0.05 |
| 14958 | H1f0 | NKLtag OSTA target | 10.79 | 10.27 | 0.95 | >0.05 |
| 68045 | 2700060E02Rik | NKLtag OSTA target | 10.79 | 10.60 | 0.98 | >0.05 |
| 67130 | Ndufa6 | NKLtag OSTA target | 10.87 | 10.60 | 0.98 | >0.05 |
| 64082 | Popdc2 | NKLtag OSTA target | 11.05 | 10.60 | 0.96 | >0.05 |
| 77864 | Ypel2 | NKLtag OSTA target | 11.69 | 11.70 | 1.00 | >0.05 |
| 17929 | Myom1 | NKLtag OSTA target | 11.77 | 11.79 | 1.00 | >0.05 |
| 259302 | Srgap3 | NKLtag OSTA target | 11.89 | 12.68 | 1.07 | >0.05 |
| 18642 | Pfkm | NKLtag OSTA target | 11.93 | 11.44 | 0.96 | >0.05 |
| 13602 | Sparcl1 | NKLtag OSTA target | 12.15 | 11.89 | 0.98 | >0.05 |
| 14194 | Fh1 | NKLtag OSTA target | 12.23 | 12.11 | 0.99 | >0.05 |
| 22186 | Uba52 | NKLtag OSTA target | 12.71 | 12.53 | 0.99 | >0.05 |
| 15122 | Hba-a1 | NKLtag OSTA target | 13.15 | 12.99 | 0.99 | >0.05 |
| 22186 | Uba52 | NKLtag OSTA target | 14.24 | 14.16 | 0.99 | >0.05 |
|  |  |  |  |  |  |  |
|  | | | | | | |

**Table S4: Lamin A-associated genes that change expression levels upon loss of lamin A/C**

| Entrez Gene ID | Gene Abbreviation | Target Type | Expression WT | Expression LMNA^GT-/-^ | FC LMNA^GT-/-^ vs. WT  WT WT | P-value |
| --- | --- | --- | --- | --- | --- | --- |
| 258321 | Olfr809 | NKLTAG A | 2.22 | 2.17 | 0.96 | **<0.05** |
| 58179 | Klrc3 | NKLTAG A | 2.64 | 2.57 | 0.96 | **<0.05** |
| 215900 | A630077B13Rik | NKLTAG A | 2.34 | 2.27 | 0.95 | **<0.05** |
| 385138 | BC061237 | NKLTAG A | 3.22 | 3.14 | 0.95 | **<0.05** |
| 109676 | Ank2 | NKLTAG A | 3.21 | 3.11 | 0.93 | **<0.05** |
| 58887 | Repin1 | NKLTAG A | 7.31 | 6.82 | 0.93 | **<0.05** |
| 258366 | Olfr434 | NKLTAG A | 2.38 | 2.26 | 0.92 | **<0.05** |
| 71826 | 1700001F09Rik | NKLTAG A | 3.16 | 3.02 | 0.90 | **<0.05** |
| 59031 | Chst12 | NKLTAG A | 3.84 | 3.69 | 0.90 | **<0.05** |
| 66322 | 1700011A15Rik | NKLTAG A | 2.87 | 2.68 | 0.88 | **<0.05** |
| 73318 | 1700013N18Rik | NKLTAG A | 4.23 | 3.55 | 0.62 | **<0.05** |
| 231633 | Tmem119 | NKLTAG A | 5.50 | 4.38 | 0.47 | **<0.05** |
|  | | | | | | |

| Table S5: Preferential lamin A interacting genes | | | | | | | | | |  |  |  |
| --- | --- | --- | --- | --- | --- | --- | --- | --- | --- | --- | --- | --- |
| Entrez Gene ID | Gene Abbreviation | Target Type | Expression WT+Ctrl | Expression WT+OST-A | Expression WT+OST-P | FC A/Ctrl | FC P/Ctrl | FC  P/A | Expression change | | | |
| 75801 | Six6os1 | MEF A | 2.41 | 2.41 | 2.41 | 1.00 | 1.00 | 1.00 | A- | | P- |  |
| 77397 | 9530003J23Rik | MEF A | 2.53 | 2.64 | 2.53 | 1.04 | 1.00 | 0.96 | A- | | P- |  |
| 13618 | Ednrb | MEF A | 3.28 | 3.22 | 3.21 | 0.98 | 0.98 | 0.99 | A- | | P- |  |
| 619332 | 4933416C03Rik | MEF A | 3.38 | 3.42 | 3.45 | **1.01*** | 1.02 | 1.01 | A↑ | | P- |  |
| 209776 | Gpr139 | MEF A | 3.76 | 3.79 | 3.83 | 1.01 | 1.02 | 1.01 | A- | | P- |  |
| 70069 | H1fnt | MEF A | 4.34 | 4.40 | 4.30 | 1.02 | 0.99 | 0.98 | A- | | P- |  |
| 66816 | Thap2\|Psrc2 | MEF A | 8.27 | 8.02 | 8.25 | 0.97 | 1.00 | 1.03 | A- | | P- |  |
| 16475 | Jub | MEF A | 8.40 | 8.45 | 8.34 | 1.01 | 0.99 | 0.99 | A- | | P- |  |

Legend: * mRNA expression level linear fold changes differ significantly (p<0.05, no fold-change criterion; Anova) in MEF OST-A vs. empty control cells, in MEF OST-P vs. empty control cells, or in MEF OST-P vs. MEF OST-A.

| Table S6: Preferential progerin interacting genes | | | | | | | | | | |  |
| --- | --- | --- | --- | --- | --- | --- | --- | --- | --- | --- | --- |
| Entrez Gene ID | Gene Abbreviation | Target Type | Expression WT+Ctrl | Expression WT+OST-A | Expression WT+OST-P | FC A/Ctrl | FC P/Ctrl | FC  P/A | Expression change | | |
| 116913 | Tpbpb | MEF P | 2.25 | 2.19 | 2.17 | 0.97 | **0.96*** | 0.99 | A- | P↓ |  |
| 382007 | Adam26b | MEF P | 2.27 | 2.24 | 2.24 | 0.99 | 0.99 | 1.00 | A- | P- |  |
| 223927 | BC048502 | MEF P | 2.37 | 2.46 | 2.43 | 1.04 | 1.03 | 0.99 | A- | P- |  |
| 23793 | Adam25 | MEF P | 2.37 | 2.42 | 2.34 | 1.02 | 0.98 | 0.97 | A- | P- |  |
| 170657 | Krtap16-9 | MEF P | 2.41 | 2.61 | 2.37 | 1.08 | 0.98 | 0.91 | A- | P- |  |
| 78826 | P2ry10 | MEF P | 2.43 | 2.44 | 2.40 | 1.01 | 0.99 | 0.98 | A- | P- |  |
| 387351 | Tas2r124 | MEF P | 2.46 | 2.54 | 2.56 | 1.03 | 1.04 | **1.01*** | A- | P- |  |
| 246081 | Defb11 | MEF P | 2.53 | 2.58 | 2.58 | 1.02 | 1.02 | 1.00 | A- | P- |  |
| 21884 | Fabp9 | MEF P | 2.56 | 2.63 | 2.65 | 1.03 | 1.03 | 1.01 | A- | P- |  |
| 433178 | LOC433178 | MEF P | 2.57 | 2.57 | 2.57 | 1.00 | 1.00 | 1.00 | A- | P- |  |
| 12515 | Cd69 | MEF P | 2.62 | 2.72 | 2.70 | 1.04 | 1.03 | 0.99 | A- | P- |  |
| 171227 | V1re4 | MEF P | 2.62 | 2.85 | 2.76 | 1.09 | 1.05 | 0.97 | A- | P- |  |
| 546055 | Adam39 | MEF P | 2.63 | 2.57 | 2.57 | 0.98 | 0.98 | 1.00 | A- | P- |  |
| 234515 | Inpp4b | MEF P | 2.65 | 2.73 | 2.64 | 1.03 | 1.00 | 0.97 | A- | P- |  |
| 16545 | Kera | MEF P | 2.66 | 2.71 | 2.79 | 1.02 | 1.05 | 1.03 | A- | P- |  |
| 71773 | Ugt2b1 | MEF P | 2.68 | 2.72 | 2.65 | 1.02 | 0.99 | **0.97*** | A- | P- |  |
| 258380 | Olfr461 | MEF P | 2.71 | 2.70 | 2.64 | 1.00 | 0.97 | 0.98 | A- | P- |  |
| 170834 | Oosp1 | MEF P | 2.72 | 2.66 | 2.60 | 0.98 | 0.95 | 0.98 | A- | P- |  |
| 66106 | Smpx | MEF P | 2.74 | 2.76 | 2.72 | 1.01 | 0.99 | 0.99 | A- | P- |  |
| 17287 | Mep1a | MEF P | 2.74 | 2.71 | 2.67 | 0.99 | 0.97 | 0.99 | A- | P- |  |
| 257914 | Olfr663 | MEF P | 2.77 | 2.82 | 2.87 | 1.02 | 1.04 | 1.02 | A- | P- |  |
| 258498 | Olfr148 | MEF P | 2.77 | 2.81 | 2.89 | **1.01*** | 1.04 | 1.03 | A↑ | P- |  |
| 16165 | Il13ra2 | MEF P | 2.78 | 2.95 | 2.66 | 1.06 | 0.96 | 0.90 | A- | P- |  |
| 258276 | Olfr960 | MEF P | 2.78 | 2.85 | 2.57 | 1.03 | 0.92 | 0.90 | A- | P- |  |
| 236798 | Gpr112 | MEF P | 2.78 | 2.76 | 2.77 | 0.99 | 0.99 | 1.00 | A- | P- |  |
| 258381 | Olfr460 | MEF P | 2.79 | 2.80 | 2.70 | 1.01 | 0.97 | 0.96 | A- | P- |  |
| 13525 | Adam26a | MEF P | 2.80 | 3.01 | 2.78 | 1.08 | 1.00 | 0.93 | A- | P- |  |
| 258505 | Olfr97 | MEF P | 2.80 | 3.00 | 2.82 | **1.07*** | 1.01 | 0.94 | A↑ | P- |  |
| 18705 | Pik3c2g | MEF P | 2.82 | 2.77 | 2.83 | 0.98 | 1.00 | 1.02 | A- | P- |  |
| 21984 | Tpbpa | MEF P | 2.83 | 2.87 | 2.78 | 1.02 | 0.98 | 0.97 | A- | P- |  |
| 67690 | 1700016G05Rik | MEF P | 2.86 | 2.77 | 2.86 | 0.97 | 1.00 | 1.03 | A- | P- |  |
| 331535 | Serpina7 | MEF P | 2.87 | 2.75 | 2.73 | 0.96 | 0.95 | 0.99 | A- | P- |  |
| 77905 | Fate1 | MEF P | 2.88 | 2.82 | 2.87 | 0.98 | 1.00 | 1.02 | A- | P- |  |
| 259050 | Olfr652 | MEF P | 2.94 | 2.90 | 2.83 | 0.99 | 0.96 | 0.98 | A- | P- |  |
| 170738 | Kcnh7 | MEF P | 2.97 | 3.12 | 3.01 | 1.05 | 1.01 | 0.96 | A- | P- |  |
| 384703 | Olfr670 | MEF P | 3.02 | 3.02 | 2.92 | 1.00 | 0.97 | **0.97*** | A- | P- |  |
| 224291 | Ckt2 | MEF P | 3.03 | 2.96 | 2.97 | 0.98 | 0.98 | 1.00 | A- | P- |  |
| 329436 | A830093M07 | MEF P | 3.04 | 3.13 | 2.91 | 1.03 | 0.95 | 0.93 | A- | P- |  |
| 258717 | Olfr429 | MEF P | 3.05 | 3.13 | 3.18 | 1.03 | 1.04 | 1.01 | A- | P- |  |
| 224247 | E330017A01Rik | MEF P | 3.07 | 3.08 | 3.09 | 1.00 | 1.00 | 1.00 | A- | P- |  |
| 258327 | Olfr958 | MEF P | 3.07 | 3.02 | 3.13 | 0.98 | 1.02 | 1.04 | A- | P- |  |
| 170939 | AY026312 | MEF P | 3.10 | 3.13 | 3.07 | 1.01 | 0.99 | 0.98 | A- | P- |  |
| 234203 | Zfp353 | MEF P | 3.10 | 3.11 | 3.10 | 1.00 | 1.00 | 1.00 | A- | P- |  |
| 15572 | Elavl4 | MEF P | 3.11 | 3.15 | 3.18 | 1.01 | 1.02 | 1.01 | A- | P- |  |
| 13884 | Es1 | MEF P | 3.14 | 3.27 | 3.04 | 1.04 | 0.97 | 0.93 | A- | P- |  |
| 259062 | Olfr667 | MEF P | 3.14 | 3.22 | 3.22 | 1.03 | 1.03 | 1.00 | A- | P- |  |
| 239931 | Cldn17 | MEF P | 3.17 | 3.13 | 3.19 | 0.99 | 1.01 | 1.02 | A- | P- |  |
| 319582 | 6430573F11Rik | MEF P | 3.21 | 3.14 | 3.12 | 0.98 | 0.97 | 0.99 | A- | P- |  |
| 24053 | Sgcg | MEF P | 3.21 | 3.35 | 3.20 | **1.04*** | 1.00 | 0.95 | A↑ | P- |  |
| 224756 | H2-M1 | MEF P | 3.22 | 3.52 | 3.19 | **1.09*** | 0.99 | 0.91 | A↑ | P- |  |
| 23925 | Kel | MEF P | 3.24 | 3.15 | 3.20 | 0.97 | 0.99 | 1.02 | A- | P- |  |
| 20758 | Sprr2d | MEF P | 3.26 | 3.07 | 3.10 | 0.94 | 0.95 | 1.01 | A- | P- |  |
| 18313 | Olfr16 | MEF P | 3.26 | 3.40 | 3.46 | 1.04 | 1.06 | 1.02 | A- | P- |  |
| 224754 | H2-M11 | MEF P | 3.27 | 3.28 | 3.19 | 1.00 | 0.97 | 0.97 | A- | P- |  |
| 242100 | Pglyrp3 | MEF P | 3.29 | 3.15 | 3.07 | 0.96 | 0.93 | 0.98 | A- | P- |  |
| 319555 | A230063L24Rik | MEF P | 3.31 | 3.21 | 3.25 | 0.97 | 0.98 | 1.01 | A- | P- |  |
| 67555 | 4933434I20Rik | MEF P | 3.38 | 3.34 | 3.33 | 0.99 | 0.98 | 1.00 | A- | P- |  |
| 331374 | Dgkk | MEF P | 3.39 | 3.41 | 3.62 | 1.01 | 1.07 | 1.06 | A- | P- |  |
| 244448 | BC050188 | MEF P | 3.40 | 3.31 | 3.29 | 0.98 | 0.97 | 0.99 | A- | P- |  |
| 244334 | Defb8 | MEF P | 3.45 | 3.57 | 3.39 | 1.03 | 0.98 | 0.95 | A- | P- |  |
| 624855 | LOC624855 | MEF P | 3.50 | 3.41 | 3.70 | 0.97 | 1.05 | **1.08*** | A- | P- |  |
| 259100 | Olfr666 | MEF P | 3.53 | 3.24 | 3.52 | 0.92 | 1.00 | 1.09 | A- | P- |  |
| 72230 | Zfp558\|Mbd3l1 | MEF P | 3.56 | 3.70 | 3.69 | 1.04 | 1.04 | 1.00 | A- | P- |  |
| 242384 | Lrrn6c | MEF P | 3.83 | 3.76 | 3.83 | 0.98 | 1.00 | 1.02 | A- | P- |  |
| 14408 | Gabrr1 | MEF P | 3.84 | 3.79 | 3.85 | 0.99 | 1.00 | 1.01 | A- | P- |  |
| 432769 | BC038328 | MEF P | 3.88 | 3.93 | 3.90 | 1.01 | 1.00 | 0.99 | A- | P- |  |
| 258267 | Olfr370 | MEF P | 3.95 | 3.93 | 4.03 | 1.00 | 1.02 | 1.03 | A- | P- |  |
| 272790 | Magee2 | MEF P | 3.97 | 4.01 | 3.92 | 1.01 | 0.99 | 0.98 | A- | P- |  |
| 16171 | Il17a | MEF P | 4.04 | 4.18 | 4.11 | 1.03 | 1.02 | 0.99 | A- | P- |  |
| 435845 | Tmprss11c | MEF P | 4.26 | 4.23 | 4.23 | 0.99 | 0.99 | 1.00 | A- | P- |  |
| 56079 | Astn2 | MEF P | 4.29 | 4.31 | 4.44 | 1.00 | 1.04 | 1.03 | A- | P- |  |
| 26877 | B3galt1 | MEF P | 4.43 | 4.23 | 4.14 | 0.96 | 0.93 | 0.98 | A- | P- |  |
| 244416 | Ppp1r3b | MEF P | 4.60 | 4.65 | 4.73 | 1.01 | 1.03 | 1.02 | A- | P- |  |
| 78755 | 4632404H22Rik\|4930432H15Rik | MEF P | 4.86 | 4.96 | 4.85 | 1.02 | 1.00 | 0.98 | A- | P- |  |
| 56640 | Klk4 | MEF P | 5.04 | 4.87 | 5.01 | 0.97 | 1.00 | 1.03 | A- | P- |  |
| 240041 | A630033E08Rik | MEF P | 5.04 | 4.88 | 5.22 | 0.97 | 1.04 | 1.07 | A- | P- |  |
| 16826 | Ldb2 | MEF P | 5.28 | 5.28 | 5.98 | 1.00 | **1.13*** | 1.13 | A- | P↑ |  |
| 170654 | Krtap16-4 | MEF P | 5.49 | 5.56 | 5.56 | 1.01 | 1.01 | 1.00 | A- | P- |  |
| 20503 | Slc16a7 | MEF P | 5.81 | 5.73 | 5.80 | 0.99 | 1.00 | 1.01 | A- | P- |  |
| 18986 | Pou2f1 | MEF P | 5.86 | 5.74 | 5.74 | 0.98 | 0.98 | 1.00 | A- | P- |  |
| 54725 | Igsf4a | MEF P | 6.16 | 6.12 | 5.98 | 0.99 | 0.97 | 0.98 | A- | P- |  |
| 14997 | H2-M9 | MEF P | 6.27 | 6.25 | 6.50 | 1.00 | **1.04*** | 1.04 | A- | P↑ |  |
| 13527 | Dtna | MEF P | 6.86 | 6.83 | 6.76 | 1.00 | 0.99 | 0.99 | A- | P- |  |
| 17295 | Met | MEF P | 7.24 | 7.02 | 6.53 | 0.97 | **0.90*** | 0.93 | A- | P↑ |  |
| 224273 | BC043118 | MEF P | 7.50 | 7.37 | 7.27 | 0.98 | 0.97 | 0.99 | A- | P- |  |
| 13024 | Ctla2a | MEF P | 7.52 | 7.54 | 7.59 | 1.00 | 1.01 | 1.01 | A- | P- |  |
| 80884 | Maged2 | MEF P | 8.00 | 8.07 | 8.17 | 1.01 | 1.02 | 1.01 | A- | P- |  |
| 23888 | Gpc6 | MEF P | 8.76 | 8.83 | 9.08 | 1.01 | **1.04*** | 1.03 | A- | P↑ |  |
| 12343 | Capza2 | MEF P | 10.30 | 10.06 | 10.14 | **0.98*** | 0.98 | 1.01 | A↓ | P- |  |
| 57737 | Rhox4b\|Rhox4d | MEF P |  |  |  |  |  |  |  |  |  |
| 13226 | Defcr-rs7 | MEF P |  |  |  |  |  |  |  |  |  |
| 21461 | Tcp10b\|Tcp10a | MEF P |  |  |  |  |  |  |  |  |  |
| 22310 | V2r4 | MEF P |  |  |  |  |  |  |  |  |  |
| 70211 | 2810407A14Rik | MEF P |  |  |  |  |  |  |  |  |  |
| 71351 | 5430402E10Rik | MEF P |  |  |  |  |  |  |  |  |  |
| 105594 | C330003B14Rik | MEF P |  |  |  |  |  |  |  |  |  |
| 208924 | A730045E13Rik | MEF P |  |  |  |  |  |  |  |  |  |
| 385354 | Gm1533 | MEF P |  |  |  |  |  |  |  |  |  |
| 434759 | Rhox4c | MEF P |  |  |  |  |  |  |  |  |  |

Legend: * mRNA expression level linear fold changes differ significantly (p<0.05, no fold-change criterion; Anova) in MEF OST-A vs. empty control cells, in MEF OST-P vs. empty control cells, or in MEF OST-P vs. MEF OST-A.
